# Neonatal gut *Bifidobacterium* associates with indole-3-lactic acid levels in blood and risk of ADHD at age 10

**DOI:** 10.1038/s41380-026-03480-z

**Published:** 2026-02-11

**Authors:** Michael Widdowson, Shiraz Shah, Jonathan Thorsen, Casper Sahl Poulsen, Julie B. Rosenberg, Parisa Mohammadzadeh, Jens Richardt Moellegaard Jepsen, Rebecca Vinding, Christina Egeø Poulsen, Cristina Leal Rodriguez, Casper-Emil T. Pedersen, Tingting Wang, Trine Zachariasen, Filip Ottosson, Thomas Mears Werge, Madeleine Ernst, Søren Johannes Sørensen, Bo Chawes, Klaus Bønnelykke, Urvish Trivedi, Bjørn H. Ebdrup, Jakob Stokholm

**Affiliations:** 1 COPSAC, Copenhagen Prospective Studies on Asthma in Childhood, Copenhagen University Hospital - Herlev and Gentofte, Gentofte, Denmark; 2 Department of Food Science, University of Copenhagen, Copenhagen, Denmark; 3 Center for Neuropsychiatric Schizophrenia Research (CNSR) & Center for Clinical Intervention and Neuropsychiatric Schizophrenia Research (CINS), Mental Health Center, Glostrup, Copenhagen University Hospital – Mental Health Services CPH, Copenhagen, Denmark; 4 Child and Adolescent Mental Health Center, Copenhagen University Hospital – Mental Health Services CPH, Copenhagen, Denmark; 5 Department of Clinical Medicine, Faculty of Health and Medical Sciences, University of Copenhagen, Copenhagen, Denmark; 6 Section of Microbiology, University of Copenhagen, Copenhagen, Denmark; 7 Section for Clinical Mass Spectrometry, Danish Center for Neonatal Screening, Department of Congenital Disorders, Statens Serum Institut, Copenhagen, Denmark; 8 Institute of Biological Psychiatry, Mental Health Services, Copenhagen University Hospital, DK-2100 Copenhagen Ø, Denmark; 9 Department of Clinical Medicine, University of Copenhagen, DK-2100 Copenhagen Ø, Denmark; 10 iPSYCH - The Lundbeck Foundation Initiative for Psychiatric Research, DK-2100 Copenhagen Ø, Denmark; 11 Department of Pediatrics, Slagelse Hospital, Slagelse, Denmark

**Keywords:** ADHD, Neuroscience, Molecular biology

## Abstract

The gut microbiome has been associated with brain health. The neuromodulatory effects of microbial-derived metabolites is supported by experimental evidence, and alterations of gut microbiota have been associated with the pathophysiology of certain neuropsychiatric disorders.

Research on the gut microbiome’s role in attention-deficit/hyperactivity disorder (ADHD) have been predominantly cross-sectional and seldomly within the neonatal period, missing its potential impact on critical periods shaping long-term neurodevelopmental outcomes. This study addresses how initial colonization and timing of specific gut bacteria, and their metabolic byproducts, may influence the risk of future ADHD. Using the highly-phenotyped COPSAC_2010_ birth cohort, we show that a higher level of *Bifidobacterium* in the child’s one-week gut microbiome, after extensively adjusting for genetic and early-life factors, is associated with ADHD at age 10. Our analyses also reveal that the tryptophan-derived metabolite indole-3-lactic acid (ILA) in the neonatal dried blood spot (DBS) mediates the relationship between *Bifidobacterium* and ADHD risk. The association between neonatal DBS ILA and ADHD was replicated in two independent cohorts. *Bifidobacterium* is known to promote healthy neurodevelopmental outcomes; however, these findings suggest that the initial temporal colonization pattern of *Bifidobacterium* may be particularly important for neurodevelopment. In particular, elevated *Bifidobacterium*-derived metabolite ILA levels may have adverse consequences on the child’s neurodevelopment during the first week of life. This suggests that by promoting a suitable temporal colonization pattern for *Bifidobacterium* and its production of ILA in the newborn may represent potential strategies for clinical interventions for supporting adequate neurodevelopment, and mitigating the risk of future ADHD.

## Introduction

The microbiota-gut-brain axis is a bidirectional communication system involving the interplay between the gastrointestinal tract and the central nervous system, with the gut microbiota modulating interactions from initial colonization throughout life [1–3]. Having a diverse gut microbiome seems essential for overall human health [4], including brain health [5]. At the same time, some microbial species that may confer benefits at certain periods in life might pose disadvantages during others [6, 7]. Additionally, adequate colonization and maturation of the gut microbiome in early life may be particularly important in establishing stable physiological systems [8, 9]. This period in early life, which overlaps with intense and dynamic neurodevelopment [10], represents a window of opportunity to potentially imprint lasting effects on an individual’s future psychological well-being [11–13].

Emerging and mainly preclinical evidence has suggested the gut microbiome to be directly involved in the pathophysiology of certain neuropsychiatric and neurological disorders [14–16], yet human studies remain few. It is becoming increasingly recognized that the gut microbiota produces various metabolites that can influence emotional and cognitive processes as well as social behaviors [17, 18]. A significant group of bioactive metabolites produced by gut bacteria are tryptophan derivatives, of which perturbations by microbial-tryptophan metabolism have been associated with neuropsychiatric traits [16]. Dietary tryptophan in the gut can be converted into kynurenine and indole derivatives, which are important for immune regulation and neurobiological functions [16, 19–21]. Commensal infant gut microbiota can modulate tryptophan metabolism and thereby influence the available precursor pool of tryptophan metabolites [22], which suggests that they might have a meaningful impact during neurodevelopment.

Although the gut microbiome has been implicated in a range of neuropathological and psychopathological conditions [23–26], its potential role in shaping early-life predisposition of attention-deficit/hyperactivity disorder (ADHD) remains uncertain. ADHD, a childhood onset disorder affecting approximately 5-10% of children and adolescents worldwide, is the most prevalent neurodevelopmental disorder among individuals aged 18 and younger [27–29]. It is defined by pervasive and impairing symptoms in inattention and hyperactivity-impulsivity [30], situating affected children at a higher risk for negative impacts on their quality of life [31]. The heritability of ADHD is estimated to be upwards of 80%, indicating a genetic risk comparable to that of autism spectrum disorder and schizophrenia [32, 33]. Over the last few decades there has been an observed increase in ADHD incidences, which may be explained by several factors. While these do include changes in diagnostic criteria and heightened awareness of disparities among marginalized populations, such factors alone cannot explain the increase, which points towards effects of altered environmental factors in early life [34–37]. Even so, ADHD is a complex neurodevelopmental disorder and highly heritable, but seems to be influenced by a variety of early-life factors [38] of which differences in the initial gut microbiome might contribute.

Most human research on the gut microbiome’s role in ADHD has been cross-sectional, focusing on toddler, adolescent, and adult populations [39]. Significant knowledge gaps remain in the earliest of life’s course due to a lack of deeply-phenotyped prospective birth cohort studies that incorporate additional multi-omic layers, coupled with the need for replication. Since ADHD is a neurodevelopmental disorder that originates in early life, it is highly relevant to consider whether microbial exposures and the modulatory effects of their metabolic byproducts during the neonatal period may contribute to its etiology [40].

In this study, we used the extensively phenotyped Copenhagen Prospective Studies on Asthma in Childhood-2010 (COPSAC_2010_) mother-child birth cohort of 700 Danish children to examine the early life gut microbiome in relation to ADHD at age 10. Fecal and blood metabolome samples within the first year of life were analyzed to investigate how early-life gut microbiota colonization and timing might impact long-term neurodevelopment and future ADHD risk. We report that a higher relative abundance of *Bifidobacterium* in the neonatal gut at one week, mediated by the bioactive tryptophan-derived metabolite indole-3-lactic acid, was associated with ADHD at age 10–independent of genetic risk and early-life environmental factors. These findings were replicated in two independent cohorts.

## Results

### Baseline characteristics and clinical follow-up

The COPSAC_2010_ cohort [41] characteristics are displayed in Table 1.

**Table 1:** Baseline characteristics by completion of 10-year COPSYCH visit including clinical assessment for psychopathology.

Feature	Completed 10-year visit	Did not complete 10-year visit	*p*
*n*	593	107	
Sex, female (%)	288 (48.6)	52 (48.6)	1.000
Race, caucasian (%)	567 (95.6)	103 (96.3)	0.965
Mother age, mean (SD)	32.35 (4.33)	31.88 (4.54)	0.306
Father age, mean (SD)	34.63 (5.21)	33.83 (5.56)	0.154
Season of birth			0.541
Spring	163 (27.5)	23 (21.5)	•
Summer	125 (21.1)	24 (22.4)	•
Autumn	123 (20.7)	27 (25.2)	•
Winter	182 (30.7)	33 (30.8)	•
Gestational age, mean (SD), weeks	39.88 (1.68)	39.73 (1.59)	0.384
Birth weight, mean (SD), kg	3.54 (0.55)	3.49 (0.56)	0.396
Birth length, mean (SD), cm	51.94 (2.56)	51.51 (2.42)	0.113
Mode of delivery, C-section (%)	123 (20.7)	28 (26.2)	0.259
Preeclampsia, any (%)	28 (4.7)	4 (3.7)	0.841
Mothers pre-pregnancy BMI, mean (SD), kg/m^2^	24.62 (4.44)	24.13 (4.08)	0.291
Preterm birth, yes (%)	21 (3.5)	7 (6.5)	0.234
Hospitalized at birth, yes (%)	69 (11.6)	12 (11.2)	1.000
Older sibling, yes (%)	345 (58.2)	59 (55.1)	0.632
Mother smoking during pregnancy, yes (%)	19 (3.2)	6 (5.6)	0.342
Antibiotics at birth			0.402
No	401 (68.0)	65 (60.7)	•
Mother	174 (29.5)	39 (36.4)	•
Both	9 (1.5)	1 (0.9)	•
Proband	6 (1.0)	2 (1.9)	•
Antibiotics at birth, both mother and child (%)	15 (2.5)	3 (2.8)	1.000
Furred animals (any) during pregnancy (%)	222 (37.4)	45 (42.1)	0.425
Exclusive breastfeeding duration, mean (SD)	104.74 (58.92)	94.06 (63.42)	0.098
Breastfed during 1st week, yes (%)	541 (91.2)	84 (84.0)	**0.039**
Address at birth, urban (%)	515 (86.8)	93 (86.9)	1.000
Family income at 1 week			0.429
Below 100,000	55 (9.3)	12 (11.2)	•
100,000 – 150,000	137 (23.1)	33 (30.8)	•
150,000 – 200,000	173 (29.2)	27 (25.2)	•
200,000 – 250,000	136 (23.0)	21 (19.6)	•
Above 250,000	91 (15.4)	14 (13.1)	•
Maternal education at 1 week			0.165
Low	50 (8.4)	5 (4.7)	•
Medium	374 (63.1)	77 (72.0)	•
High	169 (28.5)	25 (23.4)	•
Paternal education at 1 week			0.833
Low	58 (10.0)	12 (11.8)	•
Medium	356 (61.6)	63 (61.8)	•
High	164 (28.4)	27 (26.5)	•
Dried blood spot samples (%)	576 (97.1)	102 (95.3)	0.494
Gut microbiome samples, 1 week (%)	473 (79.8)	79 (73.8)	0.210
Gut microbiome samples, 1 month (%)	524 (88.4)	91 (85.0)	0.420
Gut microbiome samples, 1 year (%)	545 (91.9)	82 (76.6)	**<0.001**

At the 10-year follow-up, 604 participants (86.3%) underwent the COPSYCH visit [42], with 593 (85%) successfully completing a clinical assessment for psychopathology. The children who completed the visit had a higher rate of being breastfed during the first week of life, and more fecal samples collected at year one, compared with those who did not. 65 individuals (10.9%) received a research diagnosis of ADHD. A detailed comparison of those diagnosed with ADHD and those without is shown in Supplementary Table 1. In the ADHD group, there were significantly more boys, higher maternal pre-pregnancy BMI, more with older siblings and furred pets, and lower maternal education when compared with the group without ADHD.

### Community-level diversity of the early-life gut microbiota in ADHD

Of the 593 participants who successfully completed the COPSYCH psychopathology assessment at age 10, 473 children had fecal samples available at age 1 week, 524 at 1 month, and 545 at 1 year (Supplementary Figure 1) [9]. In overall alpha diversity of the early-life gut microbiota at the 1-week, 1-month, and 1-year time points, we found no significant differences in observed richness, Shannon diversity, or Faith’s phylogenetic diversity within individuals who developed ADHD by age 10 and those who did not (Supplementary Figure 2). Furthermore, no significant differences in overall beta diversity between samples were observed among individuals who developed ADHD at age 10 compared to those who did not (Supplementary Figure 3). At the 1-week time point, there was a significant difference using weighted UniFrac (*R*^*2*^ = 0.006, *p* = 0.02); however, this significance did not persist after adjusting for sex, age, maternal pre-pregnancy BMI, gestational age, birth weight, mode of delivery, older sibling, antibiotics at birth, maternal smoking during pregnancy, breastfed during the first week of life, any household furred animal during pregnancy, and maternal education.

### Early-life gut microbiota relative abundance in ADHD

**Fig. 1:**
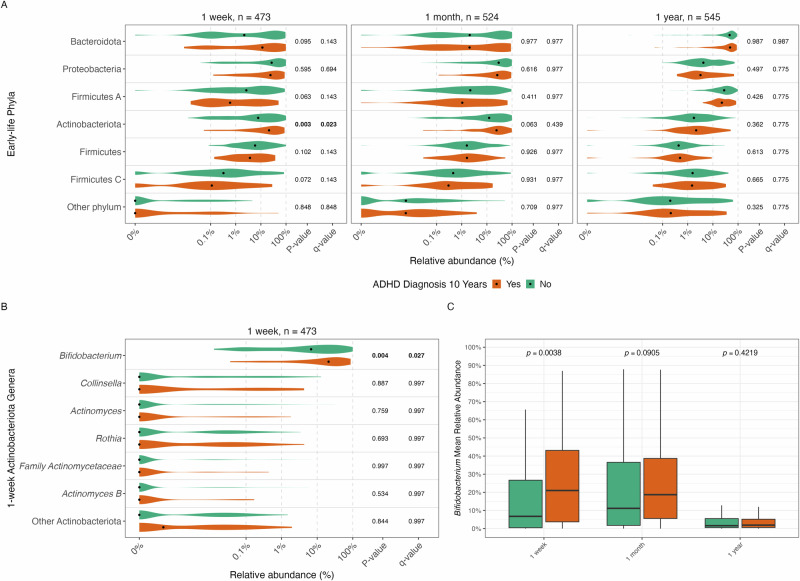
Relative abundances of gut microbiota and associations with ADHD diagnosis at 10 years. **A**) Relative abundance across three early-life time points colored according to those who developed ADHD (orange) and those who did not (green) by age 10. Differential relative abundance plots, utilizing non-parametric Wilcoxon Rank Sum Tests, are shown at the phylum level with multiple testing correction (FDR). **B**) Genus level associations at 1 week for the genera under the phylum Actinobacteriota, again with FDR correction. Along the x-axis of these plots is the relative abundance percentage on a log_10_ scale, with a black dot representing the median of the group’s relative abundance and the variance represented by the probability density. A pseudocount of 0.0001% was added to preserve zeroes on the logarithmic scale. The respective taxonomic names are displayed along the y-axis and are in descending order from most abundant to least abundant. **C**) Longitudinal *Bifidobacterium* mean relative abundance across the three early-life time points for the two groups as boxplots.

At the phylum level, we found that children, who fulfilled a diagnosis of ADHD at age 10, had a significantly higher relative abundance of *Actinobacteriota* at age 1 week (47.6% higher levels; *p*_*FDR*_ = 0.023) compared to those who did not, whereas there were no associations at 1 month or 1 year (Fig. 1A). Within *Actinobacteriota* at age 1 week, we discovered the genus *Bifidobacterium* was the driver of this signal (*p*_*FDR*_ = 0.027) (Fig. 1B). *Bifidobacterium* mean relative abundance differed significantly between the ADHD vs no ADHD group only at age 1 week, converging by age 1 year (Fig. 1C). In a crude logistic regression model, for every 10-fold increase in *Bifidobacterium* relative abundance in the one-week samples there was a 59% higher odds of developing ADHD by age 10 (OR 1.59 [1.15, 2.28], *p* = 0.007; *n* = 473). In the covariate adjusted model, the estimate remained consistent (OR 1.54 [1.08, 2.29], *p* = 0.024; *n* = 468). Upon deeper examination, *Bifidobacterium* appeared to be more strongly associated with the predominantly inattentive presentation of ADHD (crude: OR 1.76 [1.11, 3.03], *p* = 0.026; adjusted: OR 1.65 [0.98, 3.08], *p* = 0.082), compared with the predominantly hyperactive/impulsive presentation (crude: OR 1.46 [0.96, 2.38], *p* = 0.098; adjusted: OR 1.48 [0.92, 2.55], *p* = 0.128) (Supplementary Figure 4).

In a sub-analysis, an ADHD polygenic risk score (PRS) derived from genotyping data was added as a covariate to the crude and adjusted logistic regression models above to assess the potential dependence of the association between *Bifidobacterium* in the one-week gut and ADHD at age 10 from genetic risk. In a crude model with mutual adjustment of *Bifidobacterium* and the ADHD PRS, both were significant (*Bifidobacterium*: OR log_10_ increase 1.58 [1.13, 2.32], *p* = 0.012; ADHD PRS: OR 1.62 [1.17, 2.27], *p* = 0.004; *n* = 484). Both *Bifidobacterium* (OR 1.52 [1.03, 2.03], *p* = 0.042) and ADHD genetic risk (OR 1.60 [1.13, 2.31], *p* = 0.010) remained significant when further adjusting for covariates (*n* = 416), suggesting that the effects on ADHD are independent.

### *Bifidobacterium* associated blood metabolites and their relation to ADHD

**Fig. 2:**
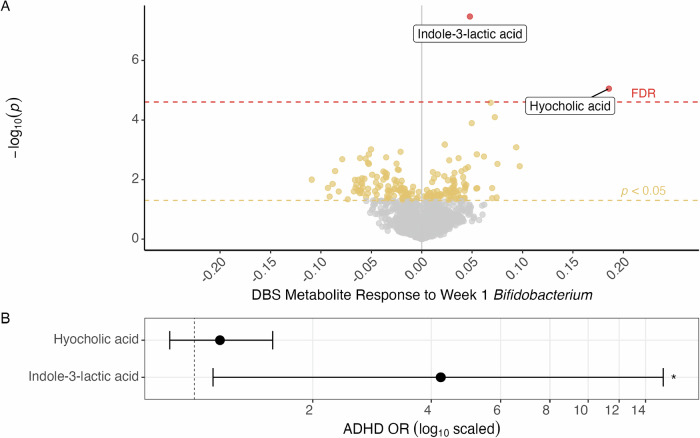
Bifidobacterium-associated metabolites from DBS metabolome and associations with ADHD. **A**) Volcano plot showing the adjusted linear estimates of metabolite measurements from the DBS (log10) taken approximately 3 days after birth and Bifidobacterium relative abundance (log10) from the 1 week fecal samples. Metabolites with nominally significant estimates (*p* < 0.05) in yellow while metabolites surviving FDR-correction are shown in red. **B**) The odds ratio of each FDR-significant metabolite (log10) tested against ADHD in an adjusted logistic regression, showing confidence intervals and “*” to symbolize *p* < 0.05. Both figures are adjusted for sex, mother’s pre-pregnancy BMI, gestational age in days, birth weight, maternal education at 1 week, having an older sibling, mode of delivery, antibiotics at birth, any household furred animals during pregnancy, maternal smoking during pregnancy, and breastfed within the first week of life, with the addition of age at the time of the 10-year COPSYCH visit in (B).

678 neonatal (1-3 days after birth) dried blood spot (DBS) heel-prick samples collected from the children had previously undergone metabolomics profiling [43]. We compared these metabolomics profiles to the 1-week gut microbiome in order to identify potential metabolites that could help explain the 1-week *Bifidobacterium* ADHD link. *Bifidobacterium* relative abundance at one-week was significantly associated with two metabolites from the DBS metabolome after covariate adjustment and correction for multiple testing (Fig. 2A), namely the tryptophan metabolite indole-3-lactic acid (ILA; β-estimate per log_10_ increase 0.048 [0.03, 0.06], *p*_*FDR*_ = 0.00007; *n* = 535) and hyocholic acid (β 0.19 [0.10, 0.27], *p*_*FDR*_ = 0.018). Of these two DBS metabolites, ILA was associated with an increased risk of ADHD at age 10 (OR 4.23 per log_10_ increase [1.12, 15.51], *p* = 0.031; *n* = 571), while hyocholic acid was not (Fig. 2B). The association between ILA and ADHD at age 10 did not persist in the metabolome from blood samples collected at 6 and 18 months (Supplementary Table 2).

Mutually adjusting for *Bifidobacterium* and ILA in a crude analysis, *Bifidobacterium* remained significant (*Bifidobacterium*: OR 1.48 [1.06, 2.15], *p* = 0.028; ILA: OR 2.91 [0.72, 11.15], *p* = 0.123; *n* = 461), whereas ILA remained significant when also adjusting the model for covariates (*Bifidobacterium*: OR 1.42 [0.98, 2.14], *p* = 0.077; ILA: OR 5.28 [1.06, 25.45], *p* = 0.039; *n* = 456), indicating partially shared and partially distinct signals. Furthermore, DBS ILA levels were not significantly associated with other bacterial genera at 1 week (Supplementary Figure 5).

### Replication in COPSAC_2000_ cohort

**Fig. 3:**
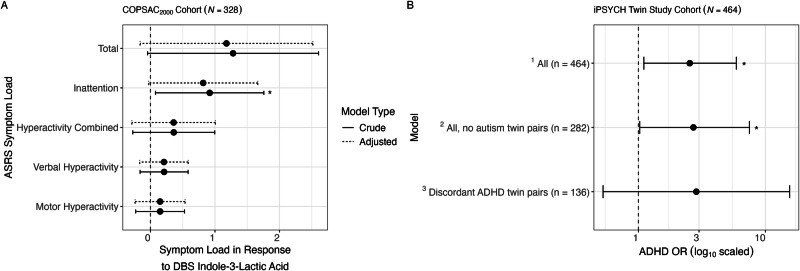
Replication of indole-3-lactic acid’s association with ADHD in two independent cohorts. **A**) Forest plot showing the linear relationship (crude; adjusted for sex, age at 18 years, gestational age, and birthweight) between indole-3-lactic acid (ILA; log_10_) in the neonate dried blood spot (DBS) and ADHD Self Report Scale (ASRS) symptom load taken at 18 years of age in the COPSAC_2000_ cohort. **B**) Forest plot showing the odds ratio (OR) for ADHD for every 10-fold of ILA in the neonate DBS in the iPSYCH twin study cohort using three different models. ^a^A logistic regression performed on the entire cohort for having a diagnosis of ADHD. ^b^A logistic regression performed omitting autism twin pairs. ^c^A conditional logistic regression performed on only discordant ADHD twin pairs. Both ^a^ and ^b^ were adjusted for sex, birth weight, gestational age, zygosity and birth order, whereas ^c^ was adjusted for birth weight. “*” denotes *p* < 0.05.

To validate our findings we used the COPSAC_2000_ cohort [44]. Here, we were able to conduct a simple linear regression to assess the relationship between DBS ILA levels and self-reported ADHD responses from the ADHD Self Report Scale (ASRS) questionnaire taken at age 18 years. 328 had DBS samples and ASRS responses. Although there was a borderline non-significant association with total ADHD symptom load (β per log_10_ increase 1.28 [−0.04, 2.60], *p* = 0.058), we did observe a significant association with the inattentive symptom load of the ASRS (β 0.92 [0.08, 1.76], *p* = 0.032) in crude analyses. After correcting for sex, age at the time of the ASRS response, gestational age and birth weight, total ADHD symptom load remained non-significant but suggestive (β 1.29 [−0.04, 2.63], *p* = 0.058), whereas inattention symptom load became non-significant (β 0.81 [-0.03, 1.55], *p* = 0.059). There were no significant findings for motor, verbal, or combined hyperactivity symptom load, though all estimates were consistently positive (Fig. 3A).

### Replication in iPSYCH twin study cohort

DBS data, processed using the same protocols as the COPSAC_2000_ and COPSAC_2010_ cohorts, was used to assess postnatal levels of ILA in the DBS in relation to ADHD within the iPSYCH twin study cohort (*N* = 464) (Fig. 3B). In examining the entire cohort for those who have a diagnosis of ADHD and those who did not, ILA was found to be significantly associated with ADHD (OR 2.54 per log_10_ increase [1.11, 5.89], *p* = 0.029; *N* = 464) after adjusting for sex, birth year, gestational age, birth weight, and zygosity in a logistic regression model. Omitting the autism twin pairs, ILA remained significantly associated with ADHD (OR 2.71 [1.03, 7.49], *p* = 0.049; *n* = 282). Lastly, using a conditional logistic regression model only within the discordant ADHD twin pairs and adjusting for birth weight, ILA kept a similar effect size although not significant in this smaller group (2.86 [0.53, 15.50], *p* = 0.223; *n* = 136).

### Mediation of *Bifidobacterium*’s association with ADHD by ILA

To clarify these partially shared associations, we explored their relatedness in a causal mediation analysis between the three involved factors, *Bifidobacterium* (predictor), ILA (mediator), and ADHD (outcome) while adjusting for covariates. The combined association of *Bifidobacterium* and ILA on ADHD was significant (β_Total_ = 0.05, [0.008, 0.14], *p* = 0.010). Additionally, after accounting for ILA levels, the direct association of *Bifidobacterium* on ADHD remained significant (Average Direct Effect, β_ADE_ 0.04 [0.001, 0.12], *p* = 0.040). The indirect (mediated) association through ILA was also significant (Average Causal Mediation Effect, β_ACME_ 0.01 [0.001, 0.02], *p* = 0.026). The average proportion of the total effect mediated through ILA was estimated to be 21% (*p* = 0.036).

### Associations of *Bifidobacterium* and ILA with ADHD dimensional psychopathology

In examining the dimensional psychopathology of ADHD with the K-SADS-PL and parental rated ADHD-RS at age 10, *Bifidobacterium* relative abundance in the 1 week gut was found to be significantly associated with sum of present ADHD inattentive symptoms (β 0.23 [0.04, 0.42], *p* = 0.019) and for the sum of present total symptoms (β 0.31 [0.005, 0.61], *p* = 0.047) from K-SADS-PL in a crude analysis. Adjusting for sex and age, both present ADHD inattentive symptoms from K-SADS-PL (β 0.22 [0.04, 0.41], *p* = 0.018) and the sum of present total symptoms from K-SADS-PL (β 0.30 [0.009, 0.58], *p* = 0.044) remained significant. Fully adjusting the model for covariates, only the association of the sum of present ADHD inattentive symptoms from KSADS-PL remained significant (β 0.20 [0.008, 0.40], *p* = 0.042). Though none of the ADHD-RS symptom loads were significant, most had a positive trend except the inattention symptom load when fully adjusted (Supplementary Figure 6).

There were no significant associations between DBS ILA levels and any of the dimensional psychopathology for ADHD though there appeared to be a positive trend with symptom loads from the ADHD-RS (Supplementary Figure 7).

### Disentangling the 1-week gut *Bifidobacterium* specie identity

**Fig. 4:**
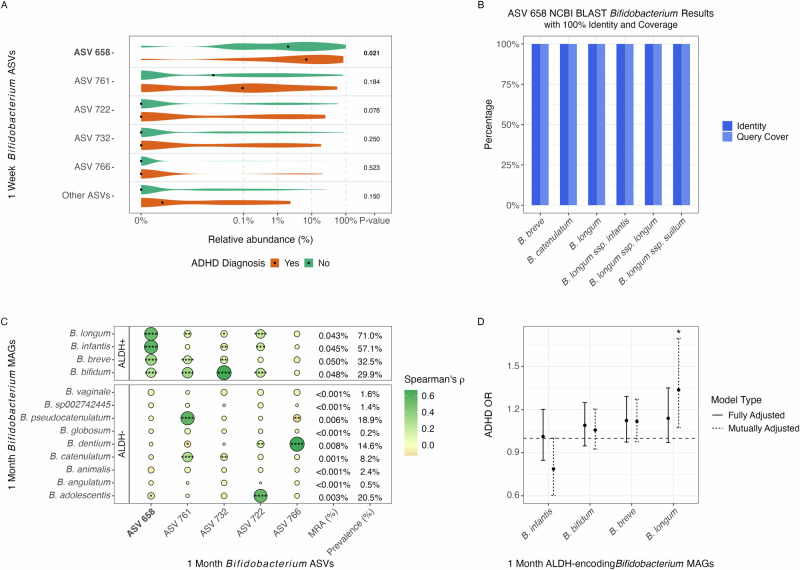
Identification of the 1 week ADHD-associated *Bifidobacterium* ASV 658 with ALDH-encoding *Bifidobacterium.* **A**) Differential relative abundance of *Bifidobacterium* ASVs between ADHD (orange) and no ADHD (green) at 1 week, utilizing non-parametric Wilcoxon Rank Sum Tests. **B**) *Bifidobacterium* species and subspecies with 100% identity and coverage from a BLAST search of the ASV 658 nucleotide sequence against NCBI’s database. **C**) Correlation plot using Spearman’s correlation of the relative abundances between the one-month *Bifidobacterium* ASVs and metagenome-assembled genome (MAG) abundances of *Bifidobacterium* species, stratified by ALDH carriage positive(+)/negative(-), with mean relative abundance (MRA) and prevalence shown for each species (“*” = *p* < 0.05; “**” = *p* < 0.01; “***” = *p* < 0.001; “****” = *p* < 0.0001). **D**) The association of the ALDH-encoding, ASV-658-associated *Bifidobacterium* MAGs at 1 month with ADHD at age 10 using fully adjusted and mutually adjust logistic regression models (“*” = *p* < 0.05).

We identified one *Bifidobacterium* 16S amplicon sequence variant (ASV 658) at 1 week that was mainly responsible for this association with ADHD at age 10 (Fig. 4A). An NCBI BLAST [45] search (Supplementary Table 3) of this ASV 16S rRNA gene sequence showed 59 distinct species and subspecies of *Bifidobacterium* with greater than 97% identity, 19 with greater than 99% identity, and 6 at 100% identity (Fig. 4B). Among these 6 were *Bifidobacterium breve*, *B. catenulatum*, *B. longum*, *B. longum subsp. infantis*, *B. longum subsp. longum*, and *B. longum subsp. suillum*. To further explore the identity of this ASV, we leveraged the closest available shotgun metagenomics data, taken at age 1 month, when residual signal of the same ASV was still present (Fig. 1C). We found significant correlations between the ASV and 1-month *Bifidobacterium* metagenome-assembled genomes (MAGs) carrying the aromatic lactate dehydrogenase (ALDH) gene [20] (Fig. 4C). These MAGs belonged to four of the six above species with 100% identity. The ASV was not associated with species not encoding ALDH. This corroborates the ILA finding as ILA is produced by the ALDH gene [20].

Similar to our observations for Actinobacteriota at the 1-month 16S level, at the MAG level there was a non-significant positive trend of association with ADHD for the relative abundances of three of the four associated ALDH-encoding *Bifidobacterium* species (*B. bifidum*, *B. breve*, *B. longum*) in relation to ADHD at age 10. However, *B. infantis* did not appear to have an association (Fig. 4D). Taking into account the confounding environmental factors that are conducive to the growth of these functionally similar *Bifidobacterium*, our mutual adjustment of these four species showed that *B. longum* was significantly associated with ADHD (OR 1.34 [1.07, 1.70], *p* = 0.012). Therefore, it appears that the ADHD-associated Bifidobacterium ASV represents a mix of these ALDH-encoding species, of which an imbalance in B. longum might be particularly important for *Bifidobacterium*’s association with ADHD.

## Discussion

### Primary findings

Here, we report an association between the relative abundance of *Bifidobacterium* in the 1-week gut microbiota and indole-3-lactic acid (ILA) in neonatal dried blood spots with the subsequent clinical assessment of ADHD at age 10. These associations remained significant despite adjustment for important covariates, including genetic risk, and multiple test corrections. In a mediation analysis, we found that part of the association between *Bifidobacterium* and risk of ADHD was mediated via ILA, but that a distinct direct signal between *Bifidobacterium* and ADHD remained as well. Furthermore, the association between neonatal ILA and ADHD was partially replicated in two independent cohorts.

### Strengths and limitations

Here, we collected fecal and blood samples in early life, coupled with the extensive recordings of early-life factors and exposures, which enabled the generation of a multi-omic dataset, including ADHD genetic risk, that aligns with critical periods of neurodevelopment. In addition, while most cohorts rely on questionnaires to assess psychopathology phenotypes, the COPSYCH 10-year visit provided a thorough characterization of ADHD psychopathology and diagnosis as an endpoint within the COPSAC_2010_ cohort [42]. The consensus diagnosis of ADHD at age 10 was confirmed externally by a professor of child and adolescent psychiatry demonstrating validity of the assessment. Another major strength of our study is the validation of our dry blood spot ILA findings in the separate COPSAC_2000_ cohort and iPSYCH twin study cohort using the same metabolomics profiling platform. While the twin study serves as an independent replication, its insights, provided by its design, mitigates the impact of unknown maternal phenotypes, such as high liability or simply undiagnosed ADHD, as well as environmental correlates thereof - which might otherwise confound a case-control design. Moreover, ADHD diagnoses of the twin study cohort are registry-based, lending greater validity to our findings by this replication. Despite these strengths, our study also exhibits several limitations. The temporal resolution of our early-life fecal and blood samples is limited, especially between the first month and first year of life, so microbiomic and metabolomic trajectories during this time interval are not able to be entirely compared to reports from other studies. However, given that *Bifidobacterium* in children later developing ADHD remains elevated at all measured time points, albeit non-significant after 1 week, it doesn’t seem likely that its relative abundance would be reduced between them, likewise for ILA.

Due to the limitation of 16S rRNA sequencing of the fecal samples, the one-week *Bifidobacterium* ASV found to be primarily driving the association with ADHD (*Bifidobacterium* ASV 658) remains unclear as we are not able to clearly distinguish between the species that it matches to: *B. longum*, *B. breve*, *B. bifidum*, including the important subspecies *B. longum ssp. infantis* which are common in infants due to its capacity for degrading human milk oligosaccharides [46]. Since these are all common in infants, it is likely that this ASV represents a mix of species, which cannot be definitively disentangled for each child. Moreover, the ambiguity of this ASV may harbor differences in functional and metabolic properties [20]. Until further metagenomic sequencing can be done at 1 week, or elucidation through experimental models, the certainty of identity remains unknown. However, this limitation is partially mitigated by our analysis of the 1-month metagenomes indicating that the association could involve ALDH-encoding, ILA-producing *Bifidobacterium* species including *B. longum*.

There is a difference in time between the 1-week fecal samples and the DBS taken 1-3 days after birth, which may have affected the results. In the very early gut microbiome, there may be day-to-day dynamics we were not able to capture that could be addressed directly by future cohort studies. The proposed biological mechanisms by which *Bifidobacterium* associates with ADHD at age 10 remains speculative until findings can be verified in experimental models as these findings are based on observational data. Early abundant *Bifidobacterium* colonization could also be a marker of other ecological phenomena, such as a cross-feeding interaction with other bacteria, or an atypical maturity or colonization patterns. Such patterns could be driven by external exposures; however, our design permitted adjustment for many such important exposures and the associations studied remained significant despite this adjustment.

Though ILA’s association with ADHD was replicated in two independent cohorts, it is important to see the direct association between 1-week gut *Bifidobacterium* and ADHD replicated as well. Adding to this, it is also important to replicate these findings within multi-ethnic cohorts to assess the generalizability of these findings to diverse populations as COPSAC_2010_ lacks heterogeneity within its demographics (i.e., primarily Caucasian).

### Interpretation

The differences observed in the early-life gut microbiome of individuals who developed ADHD do not appear from an overall shift in microbial diversity, as evidenced by the community-level diversity analyses across all three early life time points. Instead, higher *Bifidobacterium* relative abundance in a narrow time window, the first week of life, seems to carry a specific association with later ADHD.

*Bifidobacterium* species, especially the types found in infancy, have been widely regarded as beneficial for human health and development [20, 47], and have been suggested to be protective (at 5 months) against neurodevelopmental disorders [48]. Furthermore, a higher *Bifidobacterium* abundance during early life has been associated with a lower risk of developing asthma, obesity, diabetes, metabolic disorders, and all-cause mortality [49–52], highlighting the importance of its presence and abundance in early developmental phases. Investigations into healthy gut microbiome trajectories show that the genus *Bifidobacterium* commonly peaks in abundance around four months after birth [20, 53, 54], the same age at which its benefits are frequently reported. This might be attributed to the fact that exclusive breastfeeding commonly occurs during this phase of infancy, which, in itself, is one of the most influential drivers in shaping the composition of the early-life gut microbiota [55]. In particular, the species of *Bifidobacterium* found in infancy utilize human milk oligosaccharides as a nutritional source and engage in enzymatic transformation of amino acids, producing bioactive and neuroactive molecules that modulate host systems, often inducing beneficial effects [56]. Studies showing advantages of *Bifidobacterium* often report such benefits associated with abundance levels in the age range about 4 to 6 months after birth. However, our findings suggest an important new aspect of the current paradigm of early-life *Bifidobacterium*-host dynamics: a greater proportion of *Bifidobacterium* relative abundance shortly after the initial colonization of the neonatal gut might steer atypical neurodevelopment. Our findings show that the risk of ADHD by the increased proportion of gut *Bifidobacterium* is present during the first week of life, but wanes by 1 month of age. Equivalently, there is no association between ILA and ADHD beyond the DBS sampling, pointing to potential very early neuromodulatory effects on neural networks likely associated with ADHD.

Previously, *Bifidobacterium* abundance in two separate prospective cohorts was found to be associated with infant temperament (subset of the Pregnancy Experiences and Infant Development Study, *n* = 67; FinnBrain Birth Cohort Study, *n* = 301). The studies showed a positive association between relative abundances in the gut at 1-3 weeks and 2.5 months with surgency/extraversion scores at subsequent time points (12 months and 6 months, respectively) [57, 58]. Surgency/extraversion entails high activity level, high-intensity pleasure seeking, low shyness, and impulsivity [59]. Notably, surgency and extraversion have been associated with and implicated in being an early marker of ADHD [60, 61]. These findings are consistent with our study’s results.

In a recent study it was shown that certain breastmilk-promoted *Bifidobacterium* species in the infant gut, encoding for aromatic lactate dehydrogenase (ALDH), produce substantially greater quantities of ILA [20]. Consistent with our findings, these types of human milk oligosaccharide-degrading *Bifidobacterium* species harboring the ALDH gene were identified to be *Bifidobacterium longum*, *B*. *breve, B*. *bifidum*, and subspecies *B*. *longum ssp. infantis*, thereby establishing a bio-metabolic link between particular *Bifidobacterium* species found in infancy and ILA [20]. Tryptophan, an amino acid found in human breast milk, is enzymatically transformed into indolepyruvic acid by aromatic amino acid transaminase, and is further metabolized by bifidobacterial ALDH into the bioactive compound ILA [62]. Furthermore, infant *Bifidobacterium* species demonstrate a greater propensity for ILA production than other lactate-dehydrogenase-encoding bacteria (such as *Lactobacillus* species [63]) and those species found within the adult human gut [20, 63, 64], corroborating our findings of ILA primarily associating with *Bifidobacterium* in the one-week gut (Supplementary Figure 5).

ILA interfaces with the host through agonistic interactions via the aryl hydrocarbon receptor (AhR), a highly-conserved cytosolic transcription factor governing gene expression by the binding of exogenous ligands, exhibiting a response that varies with concentration [65]. Gene expression outcomes resulting from AhR activation or inhibition vary considerably [66]. For instance, indole derivatives such as ILA, mediated by AhR, may improve intestinal barrier function [67], regulate gut mucosal immune activity [68], and modulate inflammatory responses [69, 70]. Extending beyond their local gastrointestinal effects, indoles, such as ILA, can enter the circulatory system [71] and successfully traverse the blood-brain barrier [72], thereby potentially exerting direct neuromodulatory effects on the brain, where AhR is also expressed.

Indoles have gained recognition as key mediators of the microbiota-gut-brain axis exhibiting neuroprotective and anti-inflammatory properties in a number of diseases and disorders of the nervous system [72–75]. Nevertheless, it is unexpected that bifidobacterial-derived ILA in our study, in first week of life, shows an association with increased risk of developing ADHD as it has also been demonstrated to promote neuritogenesis and neuronal differentiation in an experimental model [65], which can be regarded as beneficial for neurodevelopment. Nerve growth factor(NGF)-induced PC12 cells treated with ILA show increased expression of the NGF receptor tropomyosin receptor kinase (TrkA) and acetylcholinesterase (AchE). These cells also exhibit enhanced neuritogenesis and outgrowth, mediated by AhR binding and activation of the Ras/Extracellular Signal-Regulated Kinase (ERK) pathway, demonstrating a morphogenic effect on neuronal cells. [65]. AChE, commonly recognized for its role in hydrolyzing acetylcholine, has also been shown to play a crucial morphogenic role in axonal growth and differentiation in neural development [76, 77]. Furthermore, cells overexpressing (or upregulating) TrkA differentiate more rapidly in response to lower concentrations of NGF [78], meaning ILA may hypersensitize the target cell’s affinity for NGF, thus promoting differentiation. It could be possible that exceptional levels of circulating ILA in the one-week-old neonate may alter neurodevelopmental differentiation and connectivity patterns of certain neuronal populations in the brain, specifically those associated with ADHD. In contrast, it is possible that different early-life neuromodulatory mechanisms or other unknown neuronal populations that express AhR could be affected. Framing this within a highly sensitive and dynamic period of neurodevelopment, when embryonic neurodevelopmental progressions are waning and postnatal neurodevelopment begins, the potential for perturbations is possible. Considering the context of our findings, this phenomenon may suggest an unusual neuronal maturation pattern, one that would normally rely on a gradual increase in exposure parallel with the sensitive developing brain, such as the incremental rise in *Bifidobacterium* and ILA during the first few months of life [20, 53, 54].

Additionally, a relatively strong body of evidence substantiates the existence of bidirectional intercommunication between the underlying AhR and ERK pathways–functioning in a spatiotemporal manner in a multitude of important developmental cascades [79–83]. Dysregulation, or perturbation, during early critical stages of neurodevelopment has been associated with a myriad of neurodevelopmental disorders [79]. In a nuanced comparison, germline mutations of the Ras/ERK pathway, resulting in increased activation as observed in RASopathies, with the majority of these genetic mutations contributing to such alterations, exhibit higher rates of ADHD diagnoses and ADHD-related phenotypes compared to the general population [84]. While speculative, it is conceivable that a greater *Bifidobacterium* relative abundance in the gut at 1 week of age, along with a corresponding increase in its byproduct ILA which interfaces agonistically with these pathways, can act as a perturber of this developmental cascade in a way similarly seen in RASopathies.

Lastly, given *Bifidobacterium*’s distinct direct signal remained in mediation, there could be other ways in which *Bifidobacterium* interacts with ADHD. For example, there may be other DBS metabolites associated with ADHD but not FDR-significant, such as tryptophan derivatives that have the same direction of association, which could contribute to *Bifidobacterium*’s association with ADHD, influence other gut-brain pathways (e.g., immune response to ILA’s anti-inflammatory properties), or involve other mechanisms not yet understood. It is important to note that most children showing high relative abundances of *Bifidobacterium* in their one-week gut microbiome did not develop ADHD. In exploratory interaction analyses, selected common AhR variants implicated in mental health disorders did not modify the neonatal *Bifidobacterium* or ILA associations with ADHD (Supplementary Table 4 and Supplementary Table 5), suggesting that any AhR-related influence may involve other pathway genes or rare or regulatory variants not tested [85, 86]. This suggests that perhaps only some children may be susceptible to this specific encounter in relation to later development of ADHD, of which distinct and particular genetic differences, presence or absence of other commensal bacteria, or unaccounted environmental exposures may play an additional role. Until our findings can be better understood through experimental models, such as murine or brain organoid models, and intervention studies, the proposed mechanisms and potential causality of perturbed early-life neurodevelopment are limited. Nevertheless, promoting a suitable colonization pattern of *Bifidobacterium* in the neonatal gut microbiome, or directly mediating levels of the metabolite ILA shortly after birth, could represent potential strategies for clinical interventions that support adequate neurodevelopment.

## Conclusion

Here, we found that *Bifidobacterium* relative abundance in the gut microbiome and its production of ILA within the first week of life, but not later in childhood, are associated with risk of developing ADHD by age 10. ILA’s association with ADHD was partially replicated in two independent cohorts. This suggests that promoting a suitable temporal colonization pattern for *Bifidobacterium* in early life could aid the prevention of ADHD.

## Materials / subjects and methods

### Ethics

The study was conducted in accordance with the guiding principles of the Declaration of Helsinki and was approved by the Local Ethics Committee (H-B-2008-093) and the Danish Data Protection Agency (2015-41-3696). Both parents gave oral and written informed consent before enrolment.

### Study population

The COPSAC_2010_ cohort [41] is a population-based mother-child cohort of 700 children. The pregnant women were recruited in pregnancy week 24. The children were prospectively followed by COPSAC study pediatricians, who collected all biosamples, clinical measurements and diagnoses. Clinical visits were scheduled at 1 week, 1, 3, 6, 12, 18, 24, 30, and 36 months, yearly thereafter to age 6 and again at age 8 and 10 years at the COPSAC clinic. Data collected within the first year of life and at age 10 years was considered relevant for analysis. For baseline characteristics of the cohort, see Table 1.

### Fecal sample processing and microbiome data generation

Fecal samples were collected 1 week, 1 month, and 1 year after birth, either at the research clinic or by the parents at home using detailed instructions. Each sample was mixed on arrival with 1 mL of 10% vol/vol glycerol broth (Statens Serum Institut, Copenhagen, Denmark) and frozen at −80 °C.

Genomic DNA from the infants’ samples were extracted using the PowerMag® Soil DNA Isolation Kit and the epMotion® robotic platform model 5075 according to the manufacturer’s protocol with some alterations, including centrifugation and elution steps. DNA concentrations were determined using the Quant-iT™ PicoGreen® quantification system. Extracted DNA was stored at −20 °C.

The 16S rRNA gene amplification procedure was divided into two PCR steps. In the first PCR reaction, the hypervariable V4 region of the 16S rRNA gene was amplified with modified broad range primers 515 F (5′-GTGCCAGCMGCCGCGGTAA-3′) and 806 R (5′-GGACTACHVGGGTWTCTAAT-3′) and a reaction mixture. PCR products were quantified using the Quant-iT™ PicoGreen® quantification system (Life Technologies) and samples above a concentration of 6 ng/µL were diluted to ~3–6 ng/µL prior to further analysis.

In the second step, sequencing primers and adaptors were added before purification and quantification of the amplicons. Equimolar amounts of the amplification products were pooled and sequenced on the Illumina MiSeq System (Illumina Inc., CA, USA), with a 1.0% PhiX internal control included for each run. The sequencing output was generated as demultiplexed fastq-files for downstream analysis with up to 192 samples sequenced per run. All procedures for fecal sample processing and microbiome data generation have been previously described in detail [9].

### Bioinformatics processing

The 16 s rRNA sequencing data was analyzed by removing primer sequences with Cutadapt v1.15 [87]. Amplicon Sequence Variants (ASVs) and their relative abundances were inferred with DADA2 [88] through QIIME 2 v2018.2.0 [89]. Forward and reverse reads were trimmed 8 bp at the 5’ end. Forward and reverse reads were trimmed to 180 bp and 160 bp, respectively. Other DADA2 parameters were kept at their defaults. Taxonomy was assigned with the *assignTaxnomy* function from DADA2 using the GTDB ssu-r86 database (10.5281/zenodo.3188334) [90]. ASVs which could not be annotated at the Phylum level were discarded. Furthermore, only samples with at least 2000 reads were included. The phylogenetic tree produced by QIIME was rooted by the archaeal clade.

### Metagenome-assembled genomes data generation

To investigate the metagenome-assembled genomes (MAGs) we used 611 deeply sequenced metagenomics samples taken at 1 month. The samples consist of paired-end short reads of 150 bp. Prior to analysis the reads was preprocessed by removing adapters with BBDuk v38.96 [91] run with the settings ‘ktrim=r k = 23 mink=11 hdist=1 hdist2 = 0 ptpe tbo’, removal of reads of low-quality (including reads < 75 bp) with Sickle v1.33 [92] and removal of human contamination with BBmap v38.96 [91], leaving an average number of 33.5 million (SD: 土 24 million) reads per sample. The reads were assembled with Spades v3.15.5 [93] run with the meta-option and kmer-sizes of 21,33,55,77,99 and 127, whereafter contigs less than 1500 bp were discarded with biopython v1.79 [94]. Read-mappings to the assemblies were done using BWA-mem2 v2.2.1 [95] and sorted with Samtools v1.10 [96]. The depth was found using MetaBAT2 v2.12 [97] and contigs were binned into MAGs with VAMB v3.0.8 [98], leaving 2436 MAG clusters. Annotation, refinement and abundance estimations of the MAGs were done using MAGinator [99].

### Nucleotide BLAST search

The bacterial species identification for potential ASV candidates was performed using Nucleotide BLAST search [45] against NCBI’s database [100], focusing on its hypervariable V4 region within the genus *Bifidobacterium* for highly similar sequences (megablast) using default parameters. The top 5,000 query matches were selected and downloaded in CSV format (Supplementary Table 3). Query matches with 100% coverage and >97% identity were kept for analysis. *Bifidobacterium* gene and chromosome matches were filtered out, leaving a resulting 128 *Bifidobacterium* species and subspecies.

### Blood sample processing for metabolome data generation

Dried Blood Spot samples obtained 1-3 days after birth, and blood samples from 6 months and 18 months from the child were examined. Metabolome collection, storage, preparation, profiling, and annotation in the COPSAC_2010_ cohort have been described in detail in previous work [101–103]. Briefly, data preprocessing was conducted using XCMS and MZmine, and data quality was assessed based on the distribution of pooled samples. Metabolites exhibiting more than 30% missingness were removed from the dataset. Subsequently, missing values were imputed using the minimum value found for each feature and log_10_ transformed for all time points for statistical analyses such as linear and logistic regressions. A total of 2,313 features were extracted in the COPSAC_2010_ DBS metabolome, out of which 279 were annotated at identification levels 2 or 3 [104]. Compound annotation was facilitated by the application of the Global Natural Products Social Molecular Networking (GNPS) platform [105], the MolNetEnhancer Workflow [106], and “metID” R package version 1.2.24 [107], utilizing preprocessed tandem mass spectrometry (MS2) profiles.

### The COPSYCH 10-year visit: neuropsychiatric assessment

At the 10-year COPSYCH visit children underwent a comprehensive psychopathological assessment at the COPSAC research clinic, carried out by medical doctors and research assistants with child and adolescent psychiatric training. Diagnostic interviews using the Kiddie-Schedule for Affective Disorders and Schizophrenia for School-Age Children-Present and Lifetime Version (K-SADS-PL) [108], were separately administered with parents and child.

Categorical diagnoses (i.e., diagnosis yes/no) were assigned according to both ICD-10 [109] and DSM-5 [30]. Consensus diagnoses were established at weekly sessions by a senior researcher and psychologist in child and adolescent psychiatry and at least two examiners, with monthly external validation conducted by a professor of child and adolescent psychiatry. Inter-rater reliability assessment was estimated based on 10 K-SADS-PL screening interview video recordings during the first half of the data collection period.

To further assess the extent of psychopathology, including symptoms that might not meet the threshold for a psychiatric diagnosis, dimensional evaluations of psychiatric symptoms and traits were conducted for all children. To thoroughly assess inattentive and impulsive-hyperactive behaviors across various settings, the ADHD Rating Scale (ADHD-RS) questionnaire was administered, filled out by the parent(s) [110]. A detailed description of the COPSYCH visit has been described previously [42].

### ADHD polygenic risk

The ADHD polygenic risk score was constructed using the PRS continuous shrinkage method (PRS-CS) [111] with an automatically determined phi parameter. We restricted the analyses to approximately 1 million SNPs identified in the 1000 genomes reference panel. The input ADHD summary statistic dataset is from the most recently published ADHD GWAS [112].

### Covariate and confounder selection

Early-life exposures and other risk factors that associate with ADHD were included in the adjusted analyses: sex; maternal smoking during pregnancy [113]; gestational age (days); birth weight (kg); and maternal pre-pregnancy BMI (kg/m^2^). Early-life exposures that have an impact on the initial gut microbiome composition (i.e, the first week of life) were used in the adjusted analyses: mode of delivery, intrapartum antibiotics (child), and breastfed in the first week of life. Significant group differences between ADHD cases and non-ADHD in baseline characteristics (Supplementary Table 1) were also included: household furred animals during pregnancy (as it is significantly greater in ADHD cases in our cohort and an early-life factor influencing gut microbiome composition and diversity) [114, 115], maternal education, and having an older sibling (any) to reduce any confounding group differences and unaccounted influences. ADHD PRS was used in secondary analyses due to the reduced number with this information. Lastly, age in years at the 10-year COPSYCH visit was used for adjusting in analyses involving ADHD diagnoses.

All information was obtained by personal interview at clinical visits by medical doctors and research assistants both with child and pediatric training as previously detailed41. Medical, familial, environmental, and socio-economic factors were recorded from clinical interviews according to predefined questions and closed response categories. Maternal BMI was measured at the COPSAC clinic and calculated using mother pre-pregnancy weight and height. Weight was measured at every visit using calibrated digital weight scales. Gestational age was determined by an ultrasound nuchal translucency scan in week 13 of pregnancy.

### Replication cohorts

#### COPSAC_2000_ birth cohort

COPSAC_2000_ is an independent birth cohort consisting of 411 infants of asthmatic mothers enrolled at the age of 1 month between the years 1998 and 2001 in the greater Copenhagen area of Denmark (Supplementary Table 6) [44]. Although there was no early-life microbiome information available, there were dried blood spot samples 1-12 days after birth identically analyzed for metabolomics [101] using the same protocol described for COPSAC_2010_ above. At 18 years of age the Adult ADHD Self-Report Screening Scale v1.1 (ASRS) [116] was administered via mail. The validated ASRS questionnaire was used to assess the frequency of specific ADHD-related behaviors over the past six months and to provide insight into the severity of ADHD-like phenotypes including subscales (inattention, motor and verbal and hyperactivity) [117, 118]. Both combination and sub-scale, inattention and hyperactivity-impulsivity, scores were analyzed. Age at the time of the ASRS, sex, gestational age, and birthweight were used in the adjusted analyses. These covariates were selected to maintain consistency in adjustments across the cohorts (COPSAC_2010_ and iPSYCH twin study cohort).

#### iPSYCH twin study cohort

iPSYCH twin study cohort is a cohort of same sex twins were selected from The Lundbeck Foundation Integrative Psychiatric Research (iPSYCH) 2012 cohort [119], who were born after 1995, with a gestational age of 35-40 weeks and alive and resident in Denmark on their 1-year birthday. Twin pairs where either twin had been diagnosed with either ADHD or autism were included in the cohort, meaning that discordant pairs (where only one twin is diagnosed) were also part of the study. A control group of twins not diagnosed with ADHD (F90.0), autistic disorder (F84.0/F84.1/F84.5/F84.8/F84.9), anorexia (F50.0), bipolar disorder (F30-F31), affective/depressive mood disorder (F32-F39) or schizophrenia (F20) was also included, where codes in parentheses are ICD-10 classification codes [109]. In total, the cohort consisted of 464 twins, including 100 neonates later diagnosed with ADHD, 103 with autism and 261 not diagnosed with either (Supplementary Table 7). The ADHD cases consisted of 68 twins from pairs discordant for ADHD and 32 twins from concordant pairs (16 pairs). It is important to note that this cohort sample does not overlap with the COPSAC_2010_ cohort.

All DBS samples were submitted to untargeted metabolomic profiling using liquid chromatography tandem mass spectrometry (LC-MS/MS), using the identical procedure as COPSAC_2000_ [101], and has been described in depth previously [120]. Briefly, DBS samples were extracted in 80% methanol and reconstituted in 95% water before injection of X ul extract into a Thermo Scientific Q-Exactive Orbitrap mass spectrometer. The chromatographic separation was performed on an Acquity UPLC BEH C18 column, with the mobile phases consisting of Solvent A (0.1% formic acid in water) and solvent B (0.1% formic acid in methanol).

Preprocessing of raw data files was performed using MZmine (version 3.9.0) [121]. A detailed description of the preprocessing protocol can be found in Supplementary Materials. Annotation was performed by submitting MS/MS fragmentation spectra to feature-based molecular networking using the Global Natural Products Social Molecular Networking Platform (GNPS) [105].

### Statistical analysis

All statistical analyses were performed in R v.4.1.2 [122], and figures prepared using {ggplot2} v.3.4.3 [123] and arranged using {patchwork} v1.2.0 [124]. Microbiome data was handled with {phyloseq} v.1.40.0 [125]. Baseline variables were tested using Fisher’s exact test for categorical data, and Wilcoxon rank-sum tests for continuous data. Alpha diversity (within-sample), calculated at the ASV level, was quantified using observed richness, Shannon diversity index, and Faith’s phylogenetic diversity from {picante} v.1.8.2 [126]. Beta diversity (between-sample), calculated at the ASV level, was quantified using the Bray-Curtis, unweighted and weighted UniFrac metric [127] and inference was calculated with the adonis2 PERMANOVA, assessing marginal effects of the variables, from the package {vegan} v.2.6.4 [128]. Differential abundance at the phylum, genus, and ASV levels used Wilcoxon Rank Sum Test and {DESeq2} v.1.34.0 [129] to assess percentage change (for interpretability) between groups from the log_2_ fold change. Linear regression was used to quantify the relationship between the microbiome and blood metabolome, adjusting for potential confounding variables, to identify microbial-associated metabolites. Logistic regression was used to explore the relationship between microbial taxa selected from differential abundance analyses and bacterial-associated metabolites against ADHD. The odds ratio was used to clarify whether certain characteristics of the microbiome or metabolome are associated with an increased or decreased probability of having an ADHD diagnosis at age 10. A nonparametric bootstrap (1,000 simulations) mediation analysis was performed with cofactors of early exposures using {mediation} v4.5.0 [130] to assess the mediating effect of the metabolome between the microbiome and ADHD. A linear regression model was used for investigating the relationship between the microbiome and metabolome against the K-SADS-PL present symptoms and the ADHD-RS symptom load (dimensional psychopathology), adjusting for confounding variables mentioned within the cohort description above. The association between ILA and ADHD in the iPSYCH twin study cohort was investigated using logistic regression models. In the primary model, all neonates without ADHD were considered as controls. In the secondary model, all twins that either were diagnosed with autism or were a part of a discordant autism twin pair were excluded from the control group. The logistic regression models were adjusted for sex, birth weight, gestational age, zygosity and birth order. Additionally, we also used a conditional logistic regression model, implemented in the R package {survival*}* (3.3-1) [131], to investigate the association between ILA and ADHD within the discordant twin pairs. The conditional logistic regression models were adjusted for birth weight. All analyses for the iPSYCH twin study cohort were performed in R 4.1.1 [122]. Log_10_ transformation was applied to the relative abundance of the taxa and metabolites prior to executing linear and logistic regression models. Both linear and logistic regressions were tested in univariate and multivariable models adjusting for cofactors addressed in the cofactor selection methods. A two-sided *p*-value below 0.05 was considered statistically significant throughout all analyses. In the differential abundance analysis, multiple comparisons were controlled using the Benjamini-Hochberg False Discovery Rate (FDR) correction, expressed as q-values, as well as implemented for linear regression *p*-values from the microbiome and metabolome.

## Supplementary information

Supplementary Figures and Tables

Supplementary Table 3

Supplementary Methods
