# Supplementary material for: Neonatal gut Bifidobacterium associates with indole-3-lactic acid levels in blood and risk of ADHD at age 10
Source: Mol Psychiatry. 2026 Feb 11;31(6):3544–57. doi: 10.1038/s41380-026-03480-z (PMC13190319; doi:10.1038/s41380-026-03480-z)
Supplement: Supplementary file 1 — Supplementary Figures and Tables [file 41380_2026_3480_MOESM1_ESM.pdf]

Supplementary Figure 1

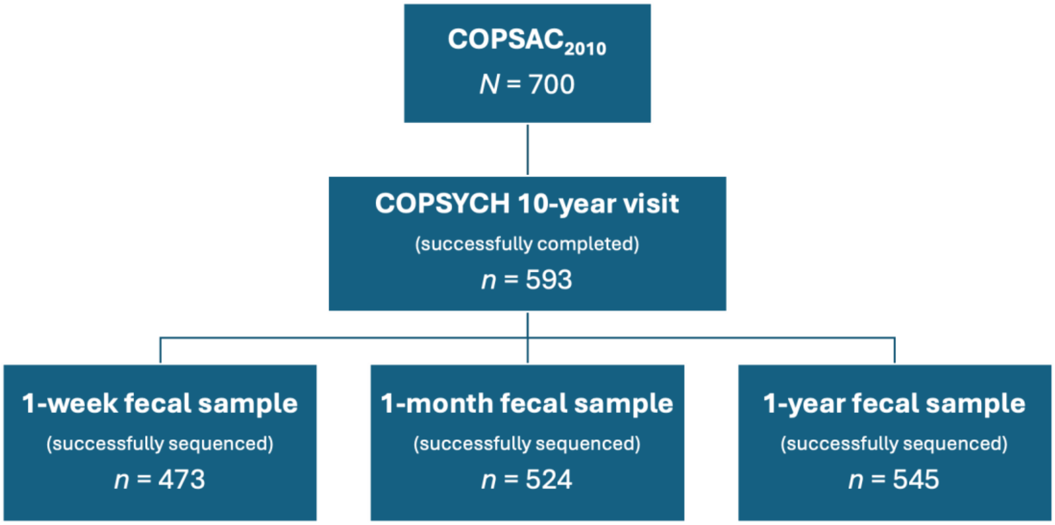

Supplementary Figure 1. Flowchart of $COPSAC_{2010}$ samples. The number of successfully sequenced gut microbiome fecal samples per early-life time point for those who also successfully completed the COPSYCH 10-year followup visit.

Supplementary Figure 2

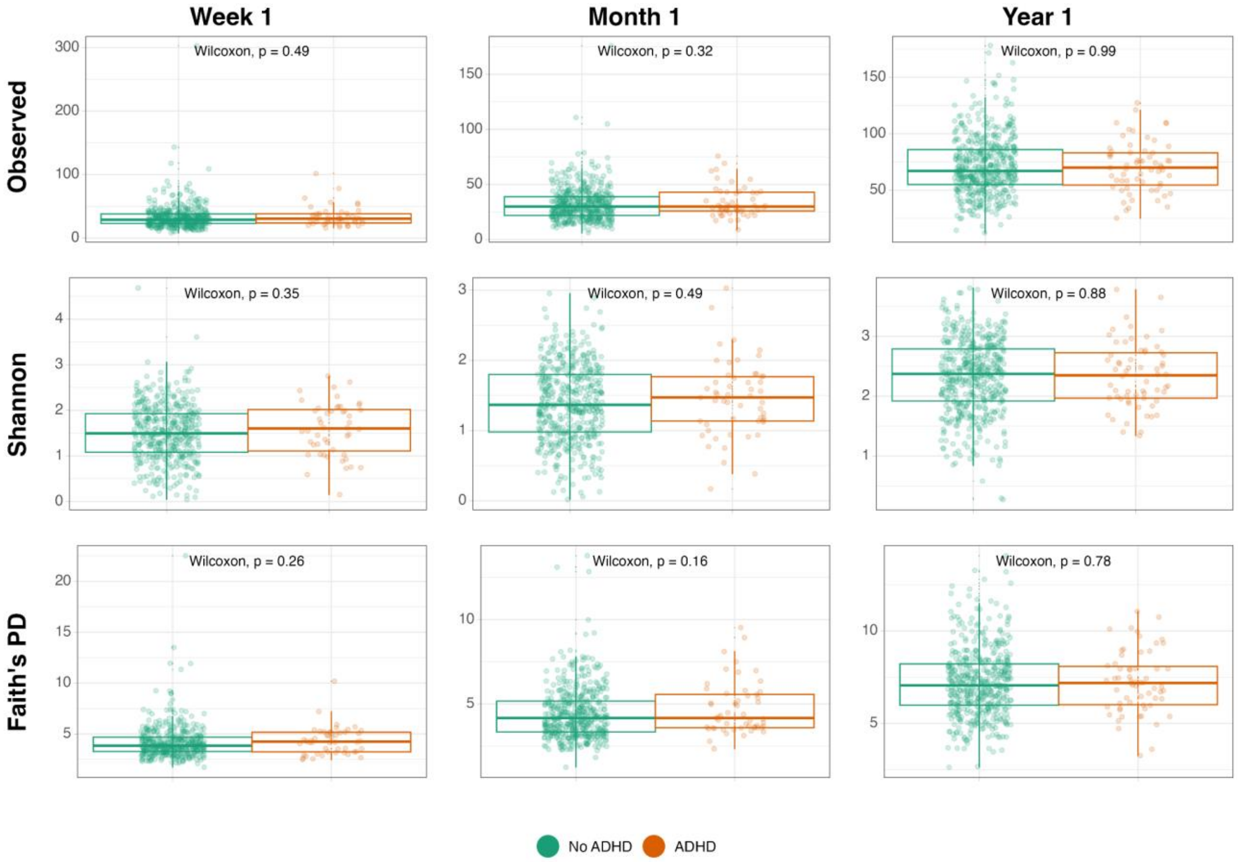

Supplementary Figure 2. Alpha diversity across early-life time points. Box plots of alpha diversity using observed richness, Shannon's Index, and Faith's phylogenetic diversity are displayed across the three time points (1 week, 1 month, and 1 year) at the genus level. Those who developed ADHD at age 10 are shown in orange and those who did not are shown in blue.

Supplementary Figure 3

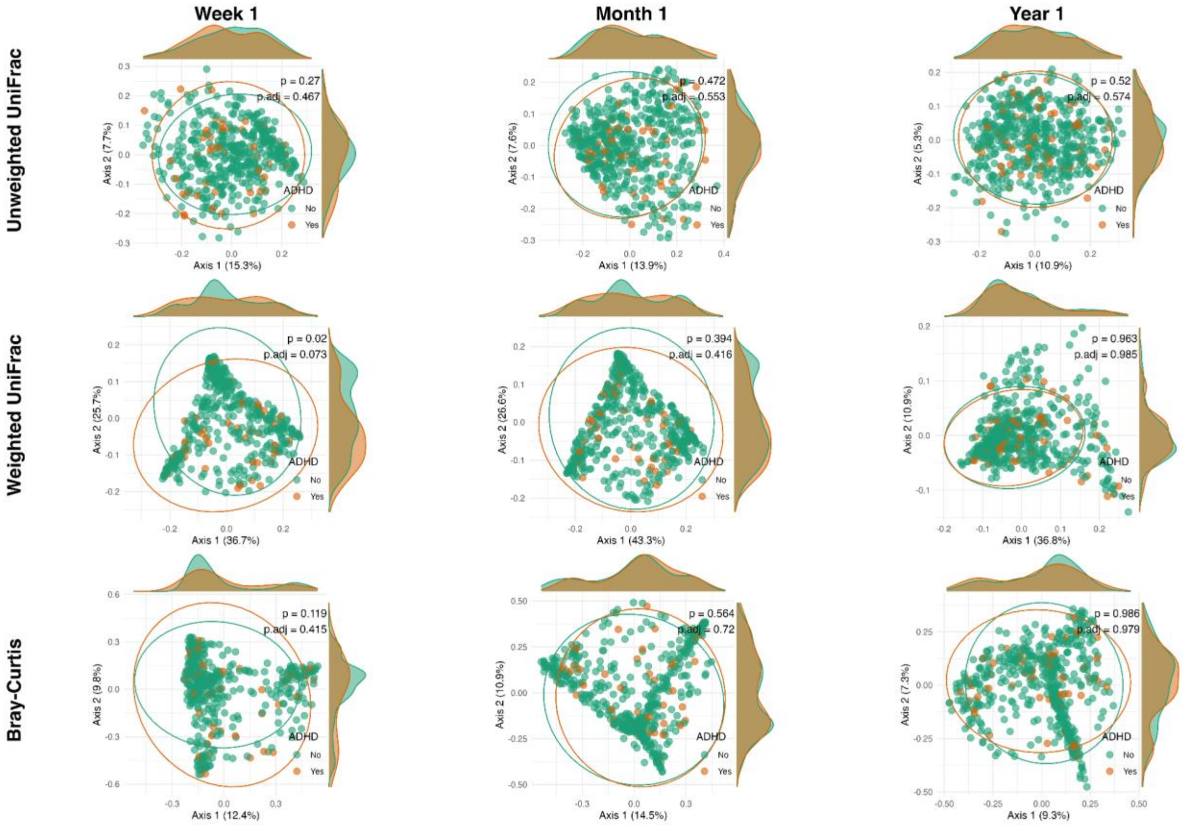

Supplementary Figure 3. Beta diversity across early-life time points. PCoA plots from three different beta diversity measures (unweighted and weighted UniFrac, and Bray-Curtis) across 1-week, 1-month, and 1-year time points. Displayed are the unadjusted and adjusted p-values from a Permutational Analysis of Variance (PERMANOVA), grouping those who developed ADHD at age 10 (orange) and those who did not (green). Co-factors for the adjusted p-values included sex, age at the time of the 10-year COPSYCH visit, maternal pre-pregnancy BMI, maternal smoking during pregnancy, household furred animal during pregnancy, maternal education at 1 week after birth, gestational age in days, weight at birth, having an older sibling, mode of delivery, antibiotics at birth (child), breastfed during the first week of life, and batch number the sample was sequenced in. The densities in the margins above and to the right of each plot show the distributions of the points on each axis for the two groups.

Supplementary Figure 4

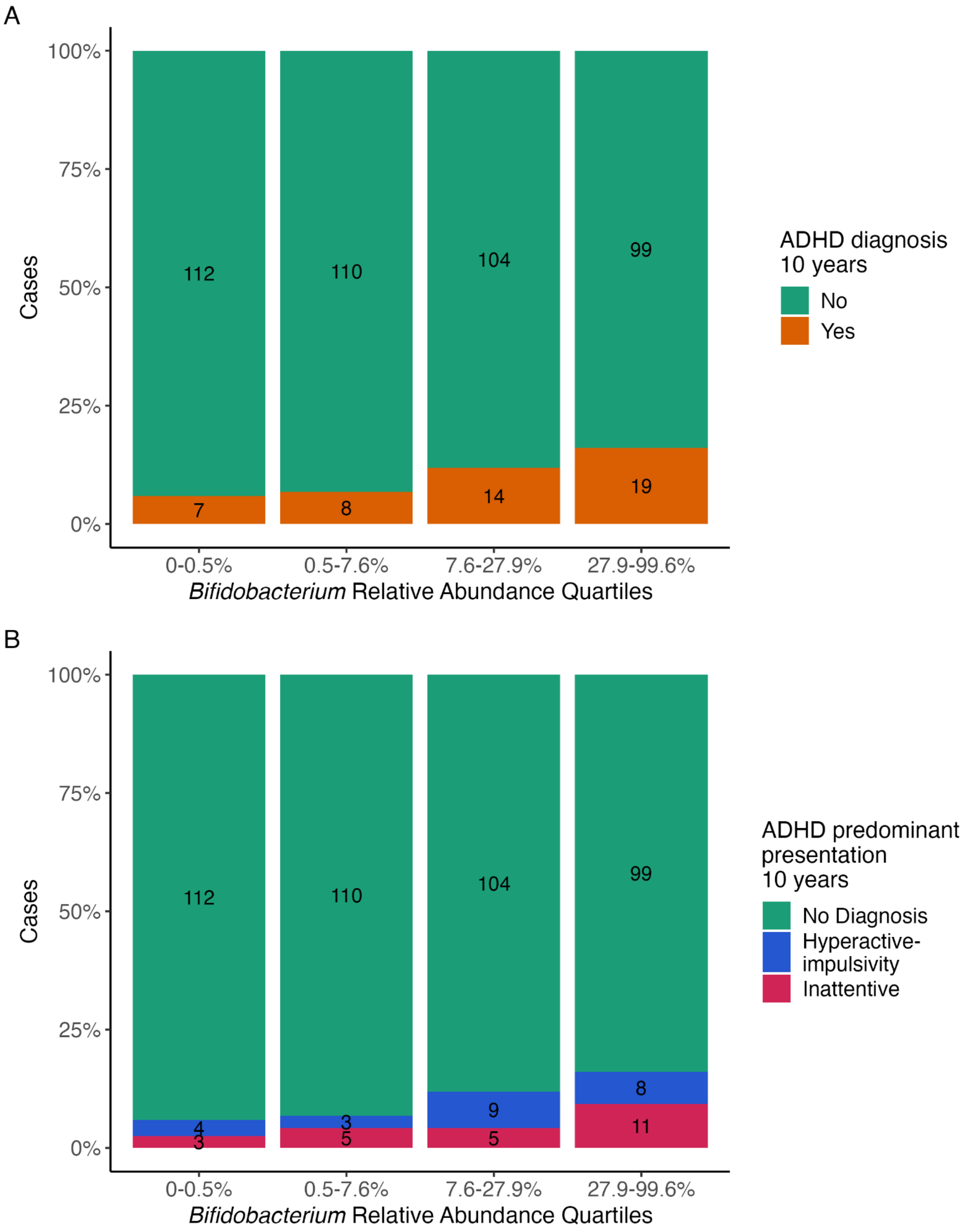

Supplementary Figure 4. Distribution of *Bifidobacterium* abundance quartiles in the one-week gut and ADHD-to-Non-ADHD ratio. Bar plots showing the quartiles of *Bifidobacterium* relative abundance within the one-week gut

microbiome. The x-axis shows the relative abundance range for each of the four quartiles. The figure displays the proportion of those diagnosed with ADHD at 10 years and those who were not (A) per quartile, and the predominant presentations (inattention and hyperactivity/impulsivity) of ADHD (B).

Supplementary Figure 5

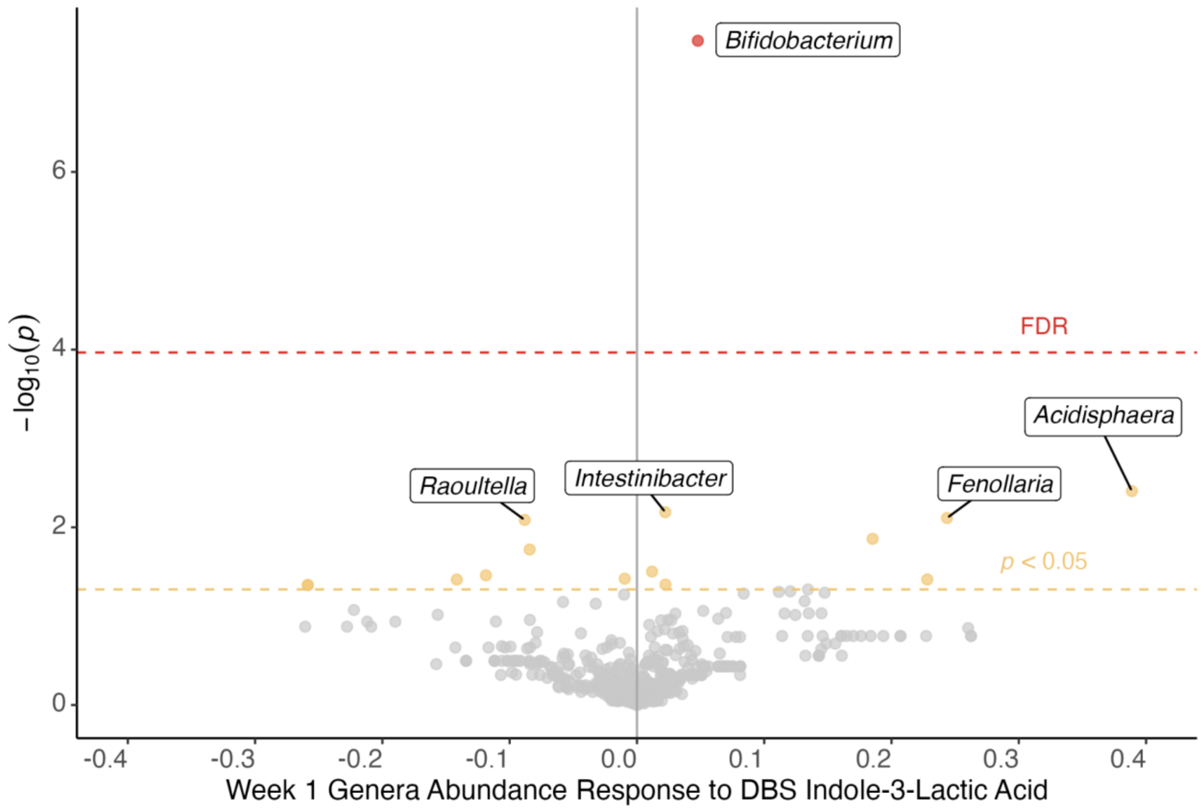

Supplementary Figure 5. Dried blood spot indole-3-lactic acid associated 1-week gut bacterial genera. A volcano plot showing the adjusted linear estimates of the non-zero 1-week gut bacterial genera relative abundance ($\log_{10}$) in response to DBS indole-3-lactic acid ($\log_{10}$). The figure displays genera with nominally significant estimates ($p < 0.05$) in yellow while genus surviving FDR-correction are shown in red. Adjusted for sex, mother's pre-pregnancy BMI, gestational age in days, birth weight, maternal education at 1 week, having an older sibling, mode of delivery, antibiotics at birth, any household furred animals during pregnancy, maternal smoking during pregnancy, and breastfed within the first week of life.

Supplementary Figure 6

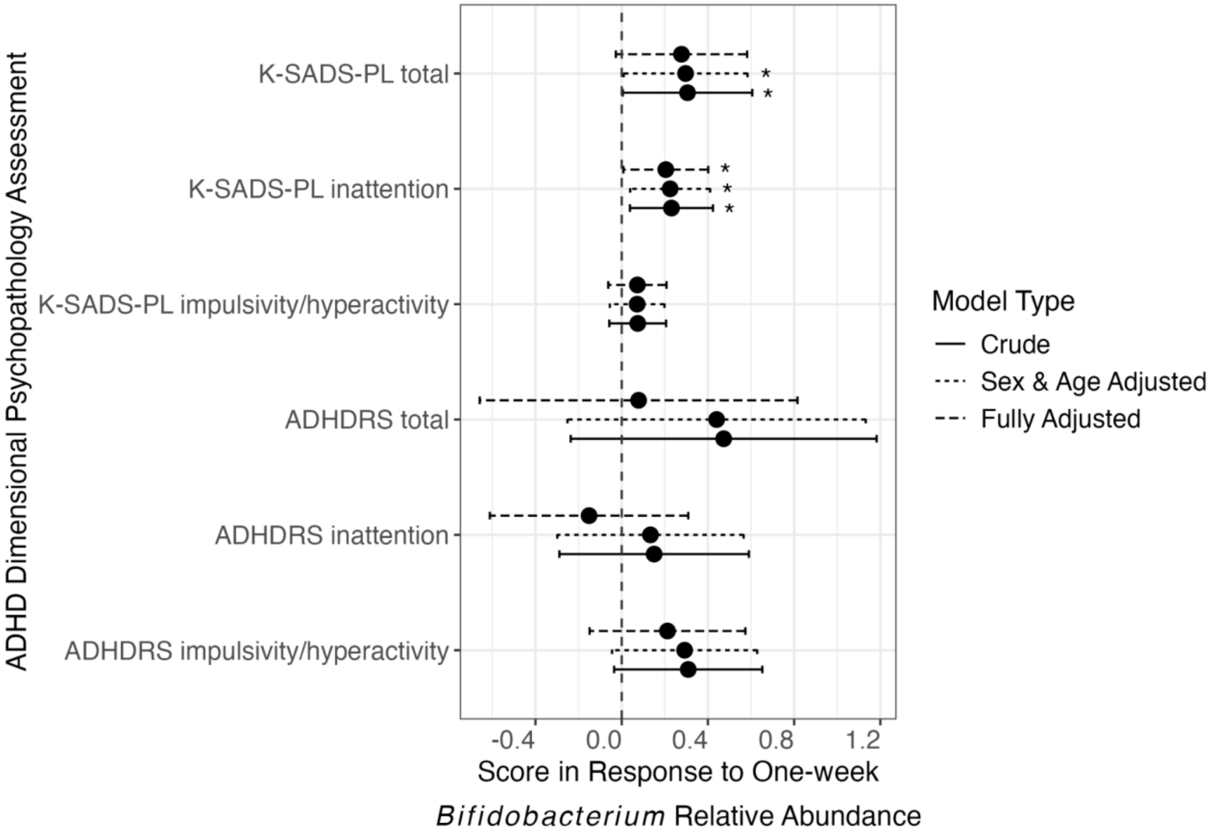

Supplementary Figure 6. Dimensional ADHD psychopathology in response to one-week gut *Bifidobacterium* relative abundance. A forest plot displaying the linear estimates of the K-SADS-PL and ADHDRS symptom loads from the COPSYCH visit in response to *Bifidobacterium* relative abundance (log_{10}) in the one-week gut microbiome using three different models: crude; sex and age adjusted; and fully adjusted for sex, age, mother's pre-pregnancy BMI, gestational age in days, birth weight, maternal education at 1 week, having an older sibling, mode of delivery, antibiotics at birth, any household furred animals during pregnancy, maternal smoking during pregnancy, and breastfed within the first week of life. "*" denotes $p<0.05$.

Supplementary Figure 7

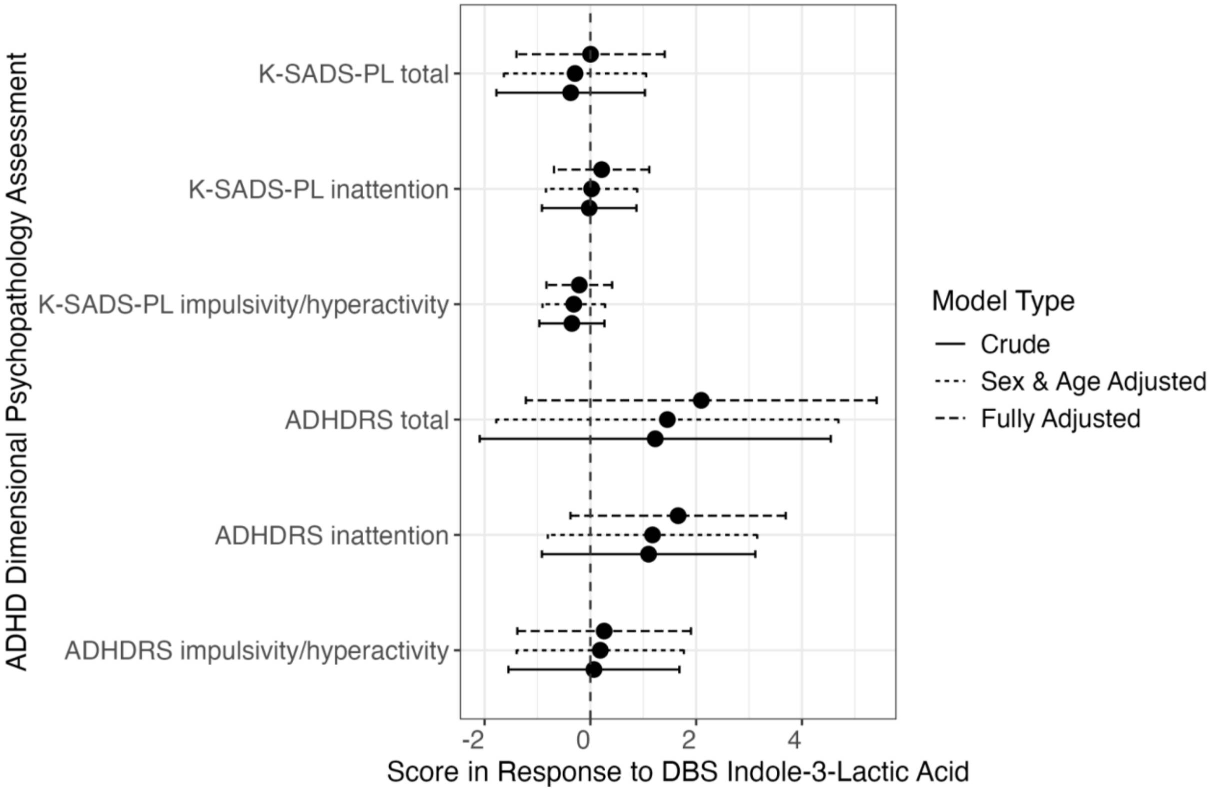

Supplementary Figure 7. Dimensional ADHD psychopathology in response to DBS indole-3-lactic acid levels. A forest plot displaying the linear estimates of the K-SADS-PL and ADHDRS symptom loads from the COPSYCH visit in response to indole-3-lactic (log_{10}) in the neonate dried blood spot using three different models: crude; sex and age adjusted; and fully adjusted for sex, age, mother's pre-pregnancy BMI, gestational age in days, birth weight, maternal education at 1 week, having an older sibling, mode of delivery, antibiotics at birth, any household furred animals during pregnancy, maternal smoking during pregnancy, and breastfed within the first week of life.

Supplementary Table 1

Supplementary Table 1. ADHD sample characteristics

Feature	No ADHD	ADHD	*p*
n	528	65	
Sex, female (%)	272 (51.5)	16 (24.6)	**<0.001**
Race, caucasian (%)	502 (95.1)	65 (100.0)	0.131
Mother age, mean (SD)	32.37 (4.29)	32.19 (4.68)	0.745
Father age, mean (SD)	34.60 (5.15)	34.83 (5.67)	0.747
Season of birth			0.968
Spring	146 (27.7)	17 (26.2)	•
Summer	110 (20.8)	15 (23.1)	•
Autumn	109 (20.6)	14 (21.5)	•
Winter	163 (30.9)	19 (29.2)	•
Gestational age, mean (SD), weeks	39.86 (1.73)	40.06 (1.23)	0.365
Birth weight, mean (SD), kg	3.54 (0.55)	3.61 (0.49)	0.284
Birth length, mean (SD), cm	51.90 (2.54)	52.23 (2.67)	0.325
Mode of delivery, C-section (%)	111 (21.0)	12 (18.5)	0.750
Preeclampsia, any (%)	26 (4.9)	2 (3.1)	0.722
Mothers pre-pregnancy BMI, mean (SD), kg/m^2	24.46 (4.24)	25.89 (5.73)	**0.015**
Preterm birth, yes (%)	21 (4.0)	0 (0.0)	0.200
Hospitalized at birth, yes (%)	64 (12.1)	5 (7.7)	0.398

Older sibling, yes (%)	299 (56.6)	46 (70.8)	**0.041**
Mother smoking during pregnancy, yes (%)	14 (2.7)	5 (7.7)	0.071
Antibiotics at birth			0.688
No	355 (67.6)	46 (70.8)	•
Mother	156 (29.7)	18 (27.7)	•
Both	9 (1.7)	0 (0.0)	•
Proband	5 (1.0)	1 (1.5)	•
Antibiotics at birth, both mother and child (%)	14 (2.7)	1 (1.5)	0.899
Furred animals (any) during pregnancy (%)	188 (35.6)	34 (52.3)	**0.013**
Exclusive breastfeeding duration, mean (SD)	105.46 (58.16)	98.83 (64.88)	0.392
Breastfed during 1st week, yes (%)	483 (91.5)	58 (89.2)	0.710
Address at birth, urban (%)	457 (86.6)	58 (89.2)	0.683
Family income at 1 week			0.217
Below 100,000	49 (9.3)	6 (9.2)	•
100,000 – 150,000	116 (22.0)	21 (32.3)	•
150,000 – 200,000	156 (29.6)	17 (26.2)	•
200,000 – 250,000	120 (22.8)	16 (24.6)	•
Above 250,000	86 (16.3)	5 (7.7)	•
Maternal education at 1 week			**0.006**
Low	40 (7.6)	10 (15.4)	•

Medium	328 (62.1)	46 (70.8)	•
High	160 (30.3)	9 (13.8)	•
Paternal education at 1 week			0.545
Low	52 (10.1)	6 (9.2)	•
Medium	312 (60.8)	44 (67.7)	•
High	149 (29.0)	15 (23.1)	•
Dried blood spot samples (%)	511 (96.8)	65 (100.0)	0.283
Gut microbiome samples, 1 week (%)	425 (80.5)	48 (73.8)	0.274
Gut microbiome samples, 1 month (%)	471 (89.2)	53 (81.5)	0.107
Gut microbiome samples, 1 year (%)	483 (91.5)	62 (95.4)	0.396

Supplementary Table 2

Supplementary table 2. $COPSAC_{2010}$ ILA associations at 6 months and 18 months.

Metabolome Blood Samples of ILA (log_{10})	OR [95% CI]	*p*
6 months	2.58 [0.70, 9.55]	0.153
18 months	4.42 [0.34, 56.94]	0.254

Supplementary Table 3

Supplementary Table 3. NCBI BLAST search results of the *Bifidobacterium* amplicon sequence variant 658 hypervariable V4 region nucleotide sequence.Table is sorted by the percent identity.

Please see ASV_658_NCBI_BLAST_hits_5000.csv for **Supplementary Table 3**.

Supplementary Table 4

Supplementary Table 4. 1-week gut Bifidobacterium relative abundance (log10) and AHR SNP interactions predicting ADHD at age 10 (sex and age adjusted)

SNP	Genotype	$OR_{Interaction}$	95% CI	*p*
**rs2228099**	CC (reference)	-	-	-
	CG (heterozygous)	1.30	[0.49, 3.30]	0.578
	GG (homozygous)	1.47	[0.52, 4.21]	0.460
	CG + GG (combined)	1.21	[0.57, 2.84]	0.636
**rs2066853**	CC (reference)	-	-	-
	CG (heterozygous)	2.43	[0.71, 16.76]	0.259
	GG (homozygous)	2.24	[0.20, Inf]	0.815
	CG + GG (combined)	0.39	[0.06, 1.31]	0.222
**rs17137566**	AA (reference)	-	-	-
	AT (heterozygous)	0.93	[0.45, 2.08]	0.850
	TT (homozygous)	6712.00	[0.17, 8.59e+31]	0.586
	AT + TT (combined)	1.05	[0.47, 2.17]	0.903

Supplementary Table 5

Supplementary Table 5. DBS indole-3-lactic acid levels (log10) and AHR SNP interactions predicting ADHD at age 10 (sex and age adjusted)

SNP	Genotype	$OR_{Interaction}$	95% CI	*p*
**rs2228099**	CC (reference)	-	-	-
	CG (heterozygous)	0.39	[0.01, 14.48]	0.606
	GG (homozygous)	0.16	[0.00, 7.44]	0.347
	CG + GG (combined)	0.34	[0.02, 4.50]	0.420
**rs2066853**	CC (reference)	-	-	-
	CG (heterozygous)	1.06	[0.02, 40.84]	0.974
	GG (homozygous)	0.02	[0.00, 2.54e+07]	0.631
	CG + GG (combined)	1.40	[0.04, 59.57]	0.856
**rs17137566**	AA (reference)	-	-	-
	AT (heterozygous)	0.31	[0.01, 5.85]	0.446
	TT (homozygous)	0.08	[0.00, 1.91e+06]	0.717
	AT + TT (combined)	3.75	[0.22, 74.91]	0.372

Supplementary Table 6

Supplementary Table 6. $COPSAC_{2000}$ birth cohort characteristics

Feature	
n	328
Age at 18-year ASRS questionnaire, mean (SD), years	17.7 (0.55)
Sex, female (%)	166 (50.6)
Gestational age, mean (SD), weeks	40.1 (1.4)
Birth weight, mean (SD), kg	3.52 (0.52)

Supplementary Table 7

Supplementary Table 7. iPSYCH Twin Study Cohort Characteristics

Feature	Controls	ADHD
n	364	100
Sex, female (%)	17 (17)	16 (24.6)
Gestational age, mean (SD), weeks	37.0 (1.2)	36.9 (1.6)
Birth weight, mean (SD), kg	2.68 (0.49)	2.68 (0.48)
Zygosity, monozygosity (%)	31 (31)	91 (25)
