# Supplementary material for: Neonatal gut Bifidobacterium associates with indole-3-lactic acid levels in blood and risk of ADHD at age 10
Source: Mol Psychiatry. 2026 Feb 11;31(6):3544–57. doi: 10.1038/s41380-026-03480-z (PMC13190319; doi:10.1038/s41380-026-03480-z)
Supplement: Supplementary file 2 — Supplementary Table 3 [file 41380_2026_3480_MOESM2_ESM.pdf]

Description	Scientific Name	Query Cover	E value	Per. ident
Bifidobacterium	Bifidobacterium	100%	9.00E-122	100
Bifidobacterium	Bifidobacterium	100%	9.00E-122	100
Bifidobacterium	Bifidobacterium	100%	9.00E-122	100
Bifidobacterium	Bifidobacterium	100%	9.00E-122	100
Bifidobacterium	Bifidobacterium	100%	9.00E-122	100
Bifidobacterium	Bifidobacterium	100%	9.00E-122	100
Bifidobacterium	Bifidobacterium	100%	9.00E-122	100
Bifidobacterium	Bifidobacterium	100%	9.00E-122	100
Bifidobacterium	Bifidobacterium	100%	9.00E-122	100
Bifidobacterium	Bifidobacterium	100%	9.00E-122	100
Bifidobacterium	Bifidobacterium	100%	9.00E-122	100
Bifidobacterium	Bifidobacterium	100%	9.00E-122	100
Bifidobacterium	Bifidobacterium	100%	9.00E-122	100
Bifidobacterium	Bifidobacterium	100%	9.00E-122	100
Bifidobacterium	Bifidobacterium	100%	9.00E-122	100
Bifidobacterium	Bifidobacterium	100%	9.00E-122	100
Bifidobacterium	Bifidobacterium	100%	9.00E-122	100
Bifidobacterium	Bifidobacterium	100%	9.00E-122	100
Bifidobacterium	Bifidobacterium	100%	9.00E-122	100
Bifidobacterium	Bifidobacterium	100%	9.00E-122	100
Bifidobacterium	Bifidobacterium	100%	9.00E-122	100
Bifidobacterium	Bifidobacterium	100%	9.00E-122	100
Bifidobacterium	Bifidobacterium	100%	9.00E-122	100
Bifidobacterium	Bifidobacterium	100%	9.00E-122	100
Bifidobacterium	Bifidobacterium	100%	9.00E-122	100
Bifidobacterium	Bifidobacterium	100%	9.00E-122	100
Bifidobacterium	Bifidobacterium	100%	9.00E-122	100
Bifidobacterium	Bifidobacterium	100%	9.00E-122	100
Bifidobacterium	Bifidobacterium	100%	9.00E-122	100
Bifidobacterium	Bifidobacterium	100%	9.00E-122	100
Bifidobacterium	Bifidobacterium	100%	9.00E-122	100
Bifidobacterium	Bifidobacterium	100%	9.00E-122	100
Bifidobacterium	Bifidobacterium	100%	9.00E-122	100
Bifidobacterium	Bifidobacterium	100%	9.00E-122	100
Bifidobacterium	Bifidobacterium	100%	9.00E-122	100
Bifidobacterium	Bifidobacterium	100%	9.00E-122	100
Bifidobacterium	Bifidobacterium	100%	9.00E-122	100
Bifidobacterium	Bifidobacterium	100%	9.00E-122	100
Bifidobacterium	Bifidobacterium	100%	9.00E-122	100
Bifidobacterium	Bifidobacterium	100%	9.00E-122	100
Bifidobacterium	Bifidobacterium	100%	9.00E-122	100
Bifidobacterium	Bifidobacterium	100%	9.00E-122	100
Bifidobacterium	Bifidobacterium	100%	9.00E-122	100

Bifidobacterium	Bifidobacterium	100%	9.00E-122	100
Bifidobacterium	Bifidobacterium	100%	9.00E-122	100
Bifidobacterium	Bifidobacterium	100%	9.00E-122	100
Bifidobacterium	Bifidobacterium	100%	9.00E-122	100
Bifidobacterium	Bifidobacterium	100%	9.00E-122	100
Bifidobacterium	Bifidobacterium	100%	9.00E-122	100
Bifidobacterium	Bifidobacterium	100%	9.00E-122	100
Bifidobacterium	Bifidobacterium	100%	9.00E-122	100
Bifidobacterium	Bifidobacterium	100%	9.00E-122	100
Bifidobacterium	Bifidobacterium	100%	9.00E-122	100
Bifidobacterium	Bifidobacterium	100%	9.00E-122	100
Bifidobacterium	Bifidobacterium	100%	9.00E-122	100
Bifidobacterium	Bifidobacterium	100%	9.00E-122	100
Bifidobacterium	Bifidobacterium	100%	9.00E-122	100
Bifidobacterium	Bifidobacterium	100%	9.00E-122	100
Bifidobacterium	Bifidobacterium	100%	9.00E-122	100
Bifidobacterium	Bifidobacterium	100%	9.00E-122	100
Bifidobacterium	Bifidobacterium	100%	9.00E-122	100
Bifidobacterium	Bifidobacterium	100%	9.00E-122	100
Bifidobacterium	Bifidobacterium	100%	9.00E-122	100
Bifidobacterium	Bifidobacterium	100%	9.00E-122	100
Bifidobacterium	Bifidobacterium	100%	9.00E-122	100
Bifidobacterium	Bifidobacterium	100%	9.00E-122	100
Bifidobacterium	Bifidobacterium	100%	9.00E-122	100
Bifidobacterium	Bifidobacterium	100%	9.00E-122	100
Bifidobacterium	Bifidobacterium	100%	9.00E-122	100
Bifidobacterium	Bifidobacterium	100%	9.00E-122	100
Bifidobacterium	Bifidobacterium	100%	9.00E-122	100
Bifidobacterium	Bifidobacterium	100%	9.00E-122	100
Bifidobacterium	Bifidobacterium	100%	9.00E-122	100
Bifidobacterium	Bifidobacterium	100%	9.00E-122	100
Bifidobacterium	Bifidobacterium	100%	9.00E-122	100
Bifidobacterium	Bifidobacterium	100%	9.00E-122	100
Bifidobacterium	Bifidobacterium	100%	9.00E-122	100
Bifidobacterium	Bifidobacterium	100%	9.00E-122	100
Bifidobacterium	Bifidobacterium	100%	9.00E-122	100
Bifidobacterium	Bifidobacterium	100%	9.00E-122	100
Bifidobacterium	Bifidobacterium	100%	9.00E-122	100
Bifidobacterium	Bifidobacterium	100%	9.00E-122	100
Bifidobacterium	Bifidobacterium	100%	9.00E-122	100
Bifidobacterium	Bifidobacterium	100%	9.00E-122	100
Bifidobacterium	Bifidobacterium	100%	9.00E-122	100
Bifidobacterium	Bifidobacterium	100%	9.00E-122	100
Bifidobacterium	Bifidobacterium	100%	9.00E-122	100

Bifidobacterium	Bifidobacterium	100%	9.00E-122	100
Bifidobacterium	Bifidobacterium	100%	9.00E-122	100
Bifidobacterium	Bifidobacterium	100%	9.00E-122	100
Bifidobacterium	Bifidobacterium	100%	9.00E-122	100
Bifidobacterium	Bifidobacterium	100%	9.00E-122	100
Bifidobacterium	Bifidobacterium	100%	9.00E-122	100
Bifidobacterium	Bifidobacterium	100%	9.00E-122	100
Bifidobacterium	Bifidobacterium	100%	9.00E-122	100
Bifidobacterium	Bifidobacterium	100%	9.00E-122	100
Bifidobacterium	Bifidobacterium	100%	9.00E-122	100
Bifidobacterium	Bifidobacterium	100%	9.00E-122	100
Bifidobacterium	Bifidobacterium	100%	9.00E-122	100
Bifidobacterium	Bifidobacterium	100%	9.00E-122	100
Bifidobacterium	Bifidobacterium	100%	9.00E-122	100
Bifidobacterium	Bifidobacterium	100%	9.00E-122	100
Bifidobacterium	Bifidobacterium	100%	9.00E-122	100
Bifidobacterium	Bifidobacterium	100%	9.00E-122	100
Bifidobacterium	Bifidobacterium	100%	9.00E-122	100
Bifidobacterium	Bifidobacterium	100%	9.00E-122	100
Bifidobacterium	Bifidobacterium	100%	9.00E-122	100
Bifidobacterium	Bifidobacterium	100%	9.00E-122	100
Bifidobacterium	Bifidobacterium	100%	9.00E-122	100
Bifidobacterium	Bifidobacterium	100%	9.00E-122	100
Bifidobacterium	Bifidobacterium	100%	9.00E-122	100
Bifidobacterium	Bifidobacterium	100%	9.00E-122	100
Bifidobacterium	Bifidobacterium	100%	9.00E-122	100
Bifidobacterium	Bifidobacterium	100%	9.00E-122	100
Bifidobacterium	Bifidobacterium	100%	9.00E-122	100
Bifidobacterium	Bifidobacterium	100%	9.00E-122	100
Bifidobacterium	Bifidobacterium	100%	9.00E-122	100
Bifidobacterium	Bifidobacterium	100%	9.00E-122	100
Bifidobacterium	Bifidobacterium	100%	9.00E-122	100
Bifidobacterium	Bifidobacterium	100%	9.00E-122	100
Bifidobacterium	Bifidobacterium	100%	9.00E-122	100
Bifidobacterium	Bifidobacterium	100%	9.00E-122	100
Bifidobacterium	Bifidobacterium	100%	9.00E-122	100
Bifidobacterium	Bifidobacterium	100%	9.00E-122	100
Bifidobacterium	Bifidobacterium	100%	9.00E-122	100
Bifidobacterium	Bifidobacterium	100%	9.00E-122	100
Bifidobacterium	Bifidobacterium	100%	9.00E-122	100
Bifidobacterium	Bifidobacterium	100%	9.00E-122	100
Bifidobacterium	Bifidobacterium	100%	9.00E-122	100
Bifidobacterium	Bifidobacterium	100%	9.00E-122	100
Bifidobacterium	Bifidobacterium	100%	9.00E-122	100

Bifidobacterium	Bifidobacterium	100%	9.00E-122	100
Bifidobacterium	Bifidobacterium	100%	9.00E-122	100
Bifidobacterium	Bifidobacterium	100%	9.00E-122	100
Bifidobacterium	Bifidobacterium	100%	9.00E-122	100
Bifidobacterium	Bifidobacterium	100%	9.00E-122	100
Bifidobacterium	Bifidobacterium	100%	9.00E-122	100
Bifidobacterium	Bifidobacterium	100%	9.00E-122	100
Bifidobacterium	Bifidobacterium	100%	9.00E-122	100
Bifidobacterium	Bifidobacterium	100%	9.00E-122	100
Bifidobacterium	Bifidobacterium	100%	9.00E-122	100
Bifidobacterium	Bifidobacterium	100%	9.00E-122	100
Bifidobacterium	Bifidobacterium	100%	9.00E-122	100
Bifidobacterium	Bifidobacterium	100%	9.00E-122	100
Bifidobacterium	Bifidobacterium	100%	9.00E-122	100
Bifidobacterium	Bifidobacterium	100%	9.00E-122	100
Bifidobacterium	Bifidobacterium	100%	9.00E-122	100
Bifidobacterium	Bifidobacterium	100%	9.00E-122	100
Bifidobacterium	Bifidobacterium	100%	9.00E-122	100
Bifidobacterium	Bifidobacterium	100%	9.00E-122	100
Bifidobacterium	Bifidobacterium	100%	9.00E-122	100
Bifidobacterium	Bifidobacterium	100%	9.00E-122	100
Bifidobacterium	Bifidobacterium	100%	9.00E-122	100
Bifidobacterium	Bifidobacterium	100%	9.00E-122	100
Bifidobacterium	Bifidobacterium	100%	9.00E-122	100
Bifidobacterium	Bifidobacterium	100%	9.00E-122	100
Bifidobacterium	Bifidobacterium	100%	9.00E-122	100
Bifidobacterium	Bifidobacterium	100%	9.00E-122	100
Bifidobacterium	Bifidobacterium	100%	9.00E-122	100
Bifidobacterium	Bifidobacterium	100%	9.00E-122	100
Bifidobacterium	Bifidobacterium	100%	9.00E-122	100
Bifidobacterium	Bifidobacterium	100%	9.00E-122	100
Bifidobacterium	Bifidobacterium	100%	9.00E-122	100
Bifidobacterium	Bifidobacterium	100%	9.00E-122	100
Bifidobacterium	Bifidobacterium	100%	9.00E-122	100
Bifidobacterium	Bifidobacterium	100%	9.00E-122	100
Bifidobacterium	Bifidobacterium	100%	9.00E-122	100
Bifidobacterium	Bifidobacterium	100%	9.00E-122	100
Bifidobacterium	Bifidobacterium	100%	9.00E-122	100
Bifidobacterium	Bifidobacterium	100%	9.00E-122	100
Bifidobacterium	Bifidobacterium	100%	9.00E-122	100
Bifidobacterium	Bifidobacterium	100%	9.00E-122	100
Bifidobacterium	Bifidobacterium	100%	9.00E-122	100
Bifidobacterium	Bifidobacterium	100%	9.00E-122	100
Bifidobacterium	Bifidobacterium	100%	9.00E-122	100

Bifidobacterium	Bifidobacterium	100%	9.00E-122	100
Bifidobacterium	Bifidobacterium	100%	9.00E-122	100
Bifidobacterium	Bifidobacterium	100%	9.00E-122	100
Bifidobacterium	Bifidobacterium	100%	9.00E-122	100
Bifidobacterium	Bifidobacterium	100%	9.00E-122	100
Bifidobacterium	Bifidobacterium	100%	9.00E-122	100
Bifidobacterium	Bifidobacterium	100%	9.00E-122	100
Bifidobacterium	Bifidobacterium	100%	9.00E-122	100
Bifidobacterium	Bifidobacterium	100%	9.00E-122	100
Bifidobacterium	Bifidobacterium	100%	9.00E-122	100
Bifidobacterium	Bifidobacterium	100%	9.00E-122	100
Bifidobacterium	Bifidobacterium	100%	9.00E-122	100
Bifidobacterium	Bifidobacterium	100%	9.00E-122	100
Bifidobacterium	Bifidobacterium	100%	9.00E-122	100
Bifidobacterium	Bifidobacterium	100%	9.00E-122	100
Bifidobacterium	Bifidobacterium	100%	9.00E-122	100
Bifidobacterium	Bifidobacterium	100%	9.00E-122	100
Bifidobacterium	Bifidobacterium	100%	9.00E-122	100
Bifidobacterium	Bifidobacterium	100%	9.00E-122	100
Bifidobacterium	Bifidobacterium	100%	9.00E-122	100
Bifidobacterium	Bifidobacterium	100%	9.00E-122	100
Bifidobacterium	Bifidobacterium	100%	9.00E-122	100
Bifidobacterium	Bifidobacterium	100%	9.00E-122	100
Bifidobacterium	Bifidobacterium	100%	9.00E-122	100
Bifidobacterium	Bifidobacterium	100%	9.00E-122	100
Bifidobacterium	Bifidobacterium	100%	9.00E-122	100
Bifidobacterium	Bifidobacterium	100%	9.00E-122	100
Bifidobacterium	Bifidobacterium	100%	9.00E-122	100
Bifidobacterium	Bifidobacterium	100%	9.00E-122	100
Bifidobacterium	Bifidobacterium	100%	9.00E-122	100
Bifidobacterium	Bifidobacterium	100%	9.00E-122	100
Bifidobacterium	Bifidobacterium	100%	9.00E-122	100
Bifidobacterium	Bifidobacterium	100%	9.00E-122	100
Bifidobacterium	Bifidobacterium	100%	9.00E-122	100
Bifidobacterium	Bifidobacterium	100%	9.00E-122	100
Bifidobacterium	Bifidobacterium	100%	9.00E-122	100
Bifidobacterium	Bifidobacterium	100%	9.00E-122	100
Bifidobacterium	Bifidobacterium	100%	9.00E-122	100
Bifidobacterium	Bifidobacterium	100%	9.00E-122	100
Bifidobacterium	Bifidobacterium	100%	9.00E-122	100
Bifidobacterium	Bifidobacterium	100%	9.00E-122	100
Bifidobacterium	Bifidobacterium	100%	9.00E-122	100
Bifidobacterium	Bifidobacterium	100%	9.00E-122	100
Bifidobacterium	Bifidobacterium	100%	9.00E-122	100

Bifidobacterium	Bifidobacterium	100%	9.00E-122	100
Bifidobacterium	Bifidobacterium	100%	9.00E-122	100
Bifidobacterium	Bifidobacterium	100%	9.00E-122	100
Bifidobacterium	Bifidobacterium	100%	9.00E-122	100
Bifidobacterium	Bifidobacterium	100%	9.00E-122	100
Bifidobacterium	Bifidobacterium	100%	9.00E-122	100
Bifidobacterium	Bifidobacterium	100%	9.00E-122	100
Bifidobacterium	Bifidobacterium	100%	9.00E-122	100
Bifidobacterium	Bifidobacterium	100%	9.00E-122	100
Bifidobacterium	Bifidobacterium	100%	9.00E-122	100
Bifidobacterium	Bifidobacterium	100%	9.00E-122	100
Bifidobacterium	Bifidobacterium	100%	9.00E-122	100
Bifidobacterium	Bifidobacterium	100%	9.00E-122	100
Bifidobacterium	Bifidobacterium	100%	9.00E-122	100
Bifidobacterium	Bifidobacterium	100%	9.00E-122	100
Bifidobacterium	Bifidobacterium	100%	9.00E-122	100
Bifidobacterium	Bifidobacterium	100%	9.00E-122	100
Bifidobacterium	Bifidobacterium	100%	9.00E-122	100
Bifidobacterium	Bifidobacterium	100%	9.00E-122	100
Bifidobacterium	Bifidobacterium	100%	9.00E-122	100
Bifidobacterium	Bifidobacterium	100%	9.00E-122	100
Bifidobacterium	Bifidobacterium	100%	9.00E-122	100
Bifidobacterium	Bifidobacterium	100%	9.00E-122	100
Bifidobacterium	Bifidobacterium	100%	9.00E-122	100
Bifidobacterium	Bifidobacterium	100%	9.00E-122	100
Bifidobacterium	Bifidobacterium	100%	9.00E-122	100
Bifidobacterium	Bifidobacterium	100%	9.00E-122	100
Bifidobacterium	Bifidobacterium	100%	9.00E-122	100
Bifidobacterium	Bifidobacterium	100%	9.00E-122	100
Bifidobacterium	Bifidobacterium	100%	9.00E-122	100
Bifidobacterium	Bifidobacterium	100%	9.00E-122	100
Bifidobacterium	Bifidobacterium	100%	9.00E-122	100
Bifidobacterium	Bifidobacterium	100%	9.00E-122	100
Bifidobacterium	Bifidobacterium	100%	9.00E-122	100
Bifidobacterium	Bifidobacterium	100%	9.00E-122	100
Bifidobacterium	Bifidobacterium	100%	9.00E-122	100
Bifidobacterium	Bifidobacterium	100%	9.00E-122	100
Bifidobacterium	Bifidobacterium	100%	9.00E-122	100
Bifidobacterium	Bifidobacterium	100%	9.00E-122	100
Bifidobacterium	Bifidobacterium	100%	9.00E-122	100
Bifidobacterium	Bifidobacterium	100%	9.00E-122	100
Bifidobacterium	Bifidobacterium	100%	9.00E-122	100
Bifidobacterium	Bifidobacterium	100%	9.00E-122	100
Bifidobacterium	Bifidobacterium	100%	9.00E-122	100

Bifidobacterium	Bifidobacterium	100%	9.00E-122	100
Bifidobacterium	Bifidobacterium	100%	9.00E-122	100
Bifidobacterium	Bifidobacterium	100%	9.00E-122	100
Bifidobacterium	Bifidobacterium	100%	9.00E-122	100
Bifidobacterium	Bifidobacterium	100%	9.00E-122	100
Bifidobacterium	Bifidobacterium	100%	9.00E-122	100
Bifidobacterium	Bifidobacterium	100%	9.00E-122	100
Bifidobacterium	Bifidobacterium	100%	9.00E-122	100
Bifidobacterium	Bifidobacterium	100%	9.00E-122	100
Bifidobacterium	Bifidobacterium	100%	9.00E-122	100
Bifidobacterium	Bifidobacterium	100%	9.00E-122	100
Bifidobacterium	Bifidobacterium	100%	9.00E-122	100
Bifidobacterium	Bifidobacterium	100%	9.00E-122	100
Bifidobacterium	Bifidobacterium	100%	9.00E-122	100
Bifidobacterium	Bifidobacterium	100%	9.00E-122	100
Bifidobacterium	Bifidobacterium	100%	9.00E-122	100
Bifidobacterium	Bifidobacterium	100%	9.00E-122	100
Bifidobacterium	Bifidobacterium	100%	9.00E-122	100
Bifidobacterium	Bifidobacterium	100%	9.00E-122	100
Bifidobacterium	Bifidobacterium	100%	9.00E-122	100
Bifidobacterium	Bifidobacterium	100%	9.00E-122	100
Bifidobacterium	Bifidobacterium	100%	9.00E-122	100
Bifidobacterium	Bifidobacterium	100%	9.00E-122	100
Bifidobacterium	Bifidobacterium	100%	9.00E-122	100
Bifidobacterium	Bifidobacterium	100%	9.00E-122	100
Bifidobacterium	Bifidobacterium	100%	9.00E-122	100
Bifidobacterium	Bifidobacterium	100%	9.00E-122	100
Bifidobacterium	Bifidobacterium	100%	9.00E-122	100
Bifidobacterium	Bifidobacterium	100%	9.00E-122	100
Bifidobacterium	Bifidobacterium	100%	9.00E-122	100
Bifidobacterium	Bifidobacterium	100%	9.00E-122	100
Bifidobacterium	Bifidobacterium	100%	9.00E-122	100
Bifidobacterium	Bifidobacterium	100%	9.00E-122	100
Bifidobacterium	Bifidobacterium	100%	9.00E-122	100
Bifidobacterium	Bifidobacterium	100%	9.00E-122	100
Bifidobacterium	Bifidobacterium	100%	9.00E-122	100
Bifidobacterium	Bifidobacterium	100%	9.00E-122	100
Bifidobacterium	Bifidobacterium	100%	9.00E-122	100
Bifidobacterium	Bifidobacterium	100%	9.00E-122	100
Bifidobacterium	Bifidobacterium	100%	9.00E-122	100
Bifidobacterium	Bifidobacterium	100%	9.00E-122	100
Bifidobacterium	Bifidobacterium	100%	9.00E-122	100
Bifidobacterium	Bifidobacterium	100%	9.00E-122	100
Bifidobacterium	Bifidobacterium	100%	9.00E-122	100

Bifidobacterium	Bifidobacterium	100%	9.00E-122	100
Bifidobacterium	Bifidobacterium	100%	9.00E-122	100
Bifidobacterium	Bifidobacterium	100%	9.00E-122	100
Bifidobacterium	Bifidobacterium	100%	9.00E-122	100
Bifidobacterium	Bifidobacterium	100%	9.00E-122	100
Bifidobacterium	Bifidobacterium	100%	9.00E-122	100
Bifidobacterium	Bifidobacterium	100%	9.00E-122	100
Bifidobacterium	Bifidobacterium	100%	9.00E-122	100
Bifidobacterium	Bifidobacterium	100%	9.00E-122	100
Bifidobacterium	Bifidobacterium	100%	9.00E-122	100
Bifidobacterium	Bifidobacterium	100%	9.00E-122	100
Bifidobacterium	Bifidobacterium	100%	9.00E-122	100
Bifidobacterium	Bifidobacterium	100%	9.00E-122	100
Bifidobacterium	Bifidobacterium	100%	9.00E-122	100
Bifidobacterium	Bifidobacterium	100%	9.00E-122	100
Bifidobacterium	Bifidobacterium	100%	9.00E-122	100
Bifidobacterium	Bifidobacterium	100%	9.00E-122	100
Bifidobacterium	Bifidobacterium	100%	9.00E-122	100
Bifidobacterium	Bifidobacterium	100%	9.00E-122	100
Bifidobacterium	Bifidobacterium	100%	9.00E-122	100
Bifidobacterium	Bifidobacterium	100%	9.00E-122	100
Bifidobacterium	Bifidobacterium	100%	9.00E-122	100
Bifidobacterium	Bifidobacterium	100%	9.00E-122	100
Bifidobacterium	Bifidobacterium	100%	9.00E-122	100
Bifidobacterium	Bifidobacterium	100%	9.00E-122	100
Bifidobacterium	Bifidobacterium	100%	9.00E-122	100
Bifidobacterium	Bifidobacterium	100%	9.00E-122	100
Bifidobacterium	Bifidobacterium	100%	9.00E-122	100
Bifidobacterium	Bifidobacterium	100%	9.00E-122	100
Bifidobacterium	Bifidobacterium	100%	9.00E-122	100
Bifidobacterium	Bifidobacterium	100%	9.00E-122	100
Bifidobacterium	Bifidobacterium	100%	9.00E-122	100
Bifidobacterium	Bifidobacterium	100%	9.00E-122	100
Bifidobacterium	Bifidobacterium	100%	9.00E-122	100
Bifidobacterium	Bifidobacterium	100%	9.00E-122	100
Bifidobacterium	Bifidobacterium	100%	9.00E-122	100
Bifidobacterium	Bifidobacterium	100%	9.00E-122	100
Bifidobacterium	Bifidobacterium	100%	9.00E-122	100
Bifidobacterium	Bifidobacterium	100%	9.00E-122	100
Bifidobacterium	Bifidobacterium	100%	9.00E-122	100
Bifidobacterium	Bifidobacterium	100%	9.00E-122	100
Bifidobacterium	Bifidobacterium	100%	9.00E-122	100
Bifidobacterium	Bifidobacterium	100%	9.00E-122	100
Bifidobacterium	Bifidobacterium	100%	9.00E-122	100

Bifidobacterium	Bifidobacterium	100%	9.00E-122	100
Bifidobacterium	Bifidobacterium	100%	9.00E-122	100
Bifidobacterium	Bifidobacterium	100%	9.00E-122	100
Bifidobacterium	Bifidobacterium	100%	9.00E-122	100
Bifidobacterium	Bifidobacterium	100%	9.00E-122	100
Bifidobacterium	Bifidobacterium	100%	9.00E-122	100
Bifidobacterium	Bifidobacterium	100%	9.00E-122	100
Bifidobacterium	Bifidobacterium	100%	9.00E-122	100
Bifidobacterium	Bifidobacterium	100%	9.00E-122	100
Bifidobacterium	Bifidobacterium	100%	9.00E-122	100
Bifidobacterium	Bifidobacterium	100%	9.00E-122	100
Bifidobacterium	Bifidobacterium	100%	9.00E-122	100
Bifidobacterium	Bifidobacterium	100%	9.00E-122	100
Bifidobacterium	Bifidobacterium	100%	9.00E-122	100
Bifidobacterium	Bifidobacterium	100%	9.00E-122	100
Bifidobacterium	Bifidobacterium	100%	9.00E-122	100
Bifidobacterium	Bifidobacterium	100%	9.00E-122	100
Bifidobacterium	Bifidobacterium	100%	9.00E-122	100
Bifidobacterium	Bifidobacterium	100%	9.00E-122	100
Bifidobacterium	Bifidobacterium	100%	9.00E-122	100
Bifidobacterium	Bifidobacterium	100%	9.00E-122	100
Bifidobacterium	Bifidobacterium	100%	9.00E-122	100
Bifidobacterium	Bifidobacterium	100%	9.00E-122	100
Bifidobacterium	Bifidobacterium	100%	9.00E-122	100
Bifidobacterium	Bifidobacterium	100%	9.00E-122	100
Bifidobacterium	Bifidobacterium	100%	9.00E-122	100
Bifidobacterium	Bifidobacterium	100%	9.00E-122	100
Bifidobacterium	Bifidobacterium	100%	9.00E-122	100
Bifidobacterium	Bifidobacterium	100%	9.00E-122	100
Bifidobacterium	Bifidobacterium	100%	9.00E-122	100
Bifidobacterium	Bifidobacterium	100%	9.00E-122	100
Bifidobacterium	Bifidobacterium	100%	9.00E-122	100
Bifidobacterium	Bifidobacterium	100%	9.00E-122	100
Bifidobacterium	Bifidobacterium	100%	9.00E-122	100
Bifidobacterium	Bifidobacterium	100%	9.00E-122	100
Bifidobacterium	Bifidobacterium	100%	9.00E-122	100
Bifidobacterium	Bifidobacterium	100%	9.00E-122	100
Bifidobacterium	Bifidobacterium	100%	9.00E-122	100
Bifidobacterium	Bifidobacterium	100%	9.00E-122	100
Bifidobacterium	Bifidobacterium	100%	9.00E-122	100
Bifidobacterium	Bifidobacterium	100%	9.00E-122	100
Bifidobacterium	Bifidobacterium	100%	9.00E-122	100
Bifidobacterium	Bifidobacterium	100%	9.00E-122	100
Bifidobacterium	Bifidobacterium	100%	9.00E-122	100

Bifidobacterium	Bifidobacterium	100%	9.00E-122	100
Bifidobacterium	Bifidobacterium	100%	9.00E-122	100
Bifidobacterium	Bifidobacterium	100%	9.00E-122	100
Bifidobacterium	Bifidobacterium	100%	9.00E-122	100
Bifidobacterium	Bifidobacterium	100%	9.00E-122	100
Bifidobacterium	Bifidobacterium	100%	9.00E-122	100
Bifidobacterium	Bifidobacterium	100%	9.00E-122	100
Bifidobacterium	Bifidobacterium	100%	9.00E-122	100
Bifidobacterium	Bifidobacterium	100%	9.00E-122	100
Bifidobacterium	Bifidobacterium	100%	9.00E-122	100
Bifidobacterium	Bifidobacterium	100%	9.00E-122	100
Bifidobacterium	Bifidobacterium	100%	9.00E-122	100
Bifidobacterium	Bifidobacterium	100%	9.00E-122	100
Bifidobacterium	Bifidobacterium	100%	9.00E-122	100
Bifidobacterium	Bifidobacterium	100%	9.00E-122	100
Bifidobacterium	Bifidobacterium	100%	9.00E-122	100
Bifidobacterium	Bifidobacterium	100%	9.00E-122	100
Bifidobacterium	Bifidobacterium	100%	9.00E-122	100
Bifidobacterium	Bifidobacterium	100%	9.00E-122	100
Bifidobacterium	Bifidobacterium	100%	9.00E-122	100
Bifidobacterium	Bifidobacterium	100%	9.00E-122	100
Bifidobacterium	Bifidobacterium	100%	9.00E-122	100
Bifidobacterium	Bifidobacterium	100%	9.00E-122	100
Bifidobacterium	Bifidobacterium	100%	9.00E-122	100
Bifidobacterium	Bifidobacterium	100%	9.00E-122	100
Bifidobacterium	Bifidobacterium	100%	9.00E-122	100
Bifidobacterium	Bifidobacterium	100%	9.00E-122	100
Bifidobacterium	Bifidobacterium	100%	9.00E-122	100
Bifidobacterium	Bifidobacterium	100%	9.00E-122	100
Bifidobacterium	Bifidobacterium	100%	9.00E-122	100
Bifidobacterium	Bifidobacterium	100%	9.00E-122	100
Bifidobacterium	Bifidobacterium	100%	9.00E-122	100
Bifidobacterium	Bifidobacterium	100%	9.00E-122	100
Bifidobacterium	Bifidobacterium	100%	9.00E-122	100
Bifidobacterium	Bifidobacterium	100%	9.00E-122	100
Bifidobacterium	Bifidobacterium	100%	9.00E-122	100
Bifidobacterium	Bifidobacterium	100%	9.00E-122	100
Bifidobacterium	Bifidobacterium	100%	9.00E-122	100
Bifidobacterium	Bifidobacterium	100%	9.00E-122	100
Bifidobacterium	Bifidobacterium	100%	9.00E-122	100
Bifidobacterium	Bifidobacterium	100%	9.00E-122	100
Bifidobacterium	Bifidobacterium	100%	9.00E-122	100
Bifidobacterium	Bifidobacterium	100%	9.00E-122	100
Bifidobacterium	Bifidobacterium	100%	9.00E-122	100

Bifidobacterium	Bifidobacterium	100%	9.00E-122	100
Bifidobacterium	Bifidobacterium	100%	9.00E-122	100
Bifidobacterium	Bifidobacterium	100%	9.00E-122	100
Bifidobacterium	Bifidobacterium	100%	9.00E-122	100
Bifidobacterium	Bifidobacterium	100%	9.00E-122	100
Bifidobacterium	Bifidobacterium	100%	9.00E-122	100
Bifidobacterium	Bifidobacterium	100%	9.00E-122	100
Bifidobacterium	Bifidobacterium	100%	9.00E-122	100
Bifidobacterium	Bifidobacterium	100%	9.00E-122	100
Bifidobacterium	Bifidobacterium	100%	9.00E-122	100
Bifidobacterium	Bifidobacterium	100%	9.00E-122	100
Bifidobacterium	Bifidobacterium	100%	9.00E-122	100
Bifidobacterium	Bifidobacterium	100%	9.00E-122	100
Bifidobacterium	Bifidobacterium	100%	9.00E-122	100
Bifidobacterium	Bifidobacterium	100%	9.00E-122	100
Bifidobacterium	Bifidobacterium	100%	9.00E-122	100
Bifidobacterium	Bifidobacterium	100%	9.00E-122	100
Bifidobacterium	Bifidobacterium	100%	9.00E-122	100
Bifidobacterium	Bifidobacterium	100%	9.00E-122	100
Bifidobacterium	Bifidobacterium	100%	9.00E-122	100
Bifidobacterium	Bifidobacterium	100%	9.00E-122	100
Bifidobacterium	Bifidobacterium	100%	9.00E-122	100
Bifidobacterium	Bifidobacterium	100%	9.00E-122	100
Bifidobacterium	Bifidobacterium	100%	9.00E-122	100
Bifidobacterium	Bifidobacterium	100%	9.00E-122	100
Bifidobacterium	Bifidobacterium	100%	9.00E-122	100
Bifidobacterium	Bifidobacterium	100%	9.00E-122	100
Bifidobacterium	Bifidobacterium	100%	9.00E-122	100
Bifidobacterium	Bifidobacterium	100%	9.00E-122	100
Bifidobacterium	Bifidobacterium	100%	9.00E-122	100
Bifidobacterium	Bifidobacterium	100%	9.00E-122	100
Bifidobacterium	Bifidobacterium	100%	9.00E-122	100
Bifidobacterium	Bifidobacterium	100%	9.00E-122	100
Bifidobacterium	Bifidobacterium	100%	9.00E-122	100
Bifidobacterium	Bifidobacterium	100%	9.00E-122	100
Bifidobacterium	Bifidobacterium	100%	9.00E-122	100
Uncultured Bific	uncultured Bifid	100%	9.00E-122	100
Bifidobacterium	Bifidobacterium	100%	9.00E-122	100
Bifidobacterium	Bifidobacterium	100%	9.00E-122	100
Bifidobacterium	Bifidobacterium	100%	9.00E-122	100
Bifidobacterium	Bifidobacterium	100%	9.00E-122	100
Uncultured Bific	uncultured Bifid	100%	9.00E-122	100
Bifidobacterium	Bifidobacterium	100%	9.00E-122	100
Bifidobacterium	Bifidobacterium	100%	9.00E-122	100

Bifidobacterium	Bifidobacterium	100%	9.00E-122	100
Bifidobacterium	Bifidobacterium	100%	9.00E-122	100
Bifidobacterium	Bifidobacterium	100%	9.00E-122	100
Bifidobacterium	Bifidobacterium	100%	9.00E-122	100
Bifidobacterium	Bifidobacterium	100%	9.00E-122	100
Bifidobacterium	Bifidobacterium	100%	9.00E-122	100
Bifidobacterium	Bifidobacterium	100%	9.00E-122	100
Bifidobacterium	Bifidobacterium	100%	9.00E-122	100
Bifidobacterium	Bifidobacterium	100%	9.00E-122	100
Bifidobacterium	Bifidobacterium	100%	9.00E-122	100
Bifidobacterium	Bifidobacterium	100%	9.00E-122	100
Bifidobacterium	Bifidobacterium	100%	9.00E-122	100
Bifidobacterium	Bifidobacterium	100%	9.00E-122	100
Bifidobacterium	Bifidobacterium	100%	9.00E-122	100
Bifidobacterium	Bifidobacterium	100%	9.00E-122	100
Bifidobacterium	Bifidobacterium	100%	9.00E-122	100
Bifidobacterium	Bifidobacterium	100%	9.00E-122	100
Bifidobacterium	Bifidobacterium	100%	9.00E-122	100
Bifidobacterium	Bifidobacterium	100%	9.00E-122	100
Bifidobacterium	Bifidobacterium	100%	9.00E-122	100
Bifidobacterium	Bifidobacterium	100%	9.00E-122	100
Bifidobacterium	Bifidobacterium	100%	9.00E-122	100
Bifidobacterium	Bifidobacterium	100%	9.00E-122	100
Bifidobacterium	Bifidobacterium	100%	9.00E-122	100
Bifidobacterium	Bifidobacterium	100%	9.00E-122	100
Bifidobacterium	Bifidobacterium	100%	9.00E-122	100
Bifidobacterium	Bifidobacterium	100%	9.00E-122	100
Bifidobacterium	Bifidobacterium	100%	9.00E-122	100
Bifidobacterium	Bifidobacterium	100%	9.00E-122	100
Bifidobacterium	Bifidobacterium	100%	9.00E-122	100
Bifidobacterium	Bifidobacterium	100%	9.00E-122	100
Bifidobacterium	Bifidobacterium	100%	9.00E-122	100
Bifidobacterium	Bifidobacterium	100%	9.00E-122	100
Bifidobacterium	Bifidobacterium	100%	9.00E-122	100
Bifidobacterium	Bifidobacterium	100%	9.00E-122	100
Bifidobacterium	Bifidobacterium	100%	9.00E-122	100
Bifidobacterium	Bifidobacterium	100%	9.00E-122	100
Bifidobacterium	Bifidobacterium	100%	9.00E-122	100
Bifidobacterium	Bifidobacterium	100%	9.00E-122	100
Bifidobacterium	Bifidobacterium	100%	9.00E-122	100
Uncultured Bific	uncultured Bifid	100%	9.00E-122	100
Uncultured Bific	uncultured Bifid	100%	9.00E-122	100
Bifidobacterium	Bifidobacterium	100%	9.00E-122	100
Bifidobacterium	Bifidobacterium	100%	9.00E-122	100
Bifidobacterium	Bifidobacterium	100%	9.00E-122	100
Bifidobacterium	Bifidobacterium	100%	9.00E-122	100

Bifidobacterium	Bifidobacterium	100%	9.00E-122	100
Bifidobacterium	Bifidobacterium	100%	9.00E-122	100
Bifidobacterium	Bifidobacterium	100%	9.00E-122	100
Bifidobacterium	Bifidobacterium	100%	9.00E-122	100
Bifidobacterium	Bifidobacterium	100%	9.00E-122	100
Bifidobacterium	Bifidobacterium	100%	9.00E-122	100
Bifidobacterium	Bifidobacterium	100%	9.00E-122	100
Bifidobacterium	Bifidobacterium	100%	9.00E-122	100
Bifidobacterium	Bifidobacterium	100%	9.00E-122	100
Bifidobacterium	Bifidobacterium	100%	9.00E-122	100
Bifidobacterium	Bifidobacterium	100%	9.00E-122	100
Bifidobacterium	Bifidobacterium	100%	9.00E-122	100
Bifidobacterium	Bifidobacterium	100%	9.00E-122	100
Bifidobacterium	Bifidobacterium	100%	9.00E-122	100
Uncultured Bifid	uncultured Bifid	100%	9.00E-122	100
Bifidobacterium	Bifidobacterium	100%	9.00E-122	100
Bifidobacterium	Bifidobacterium	100%	9.00E-122	100
Bifidobacterium	Bifidobacterium	100%	9.00E-122	100
Bifidobacterium	Bifidobacterium	100%	9.00E-122	100
Bifidobacterium	Bifidobacterium	100%	9.00E-122	100
Bifidobacterium	Bifidobacterium	100%	9.00E-122	100
Bifidobacterium	Bifidobacterium	100%	9.00E-122	100
Bifidobacterium	Bifidobacterium	100%	9.00E-122	100
Bifidobacterium	Bifidobacterium	100%	9.00E-122	100
Bifidobacterium	Bifidobacterium	100%	9.00E-122	100
Bifidobacterium	Bifidobacterium	100%	9.00E-122	100
Bifidobacterium	Bifidobacterium	100%	9.00E-122	100
Bifidobacterium	Bifidobacterium	100%	9.00E-122	100
Bifidobacterium	Bifidobacterium	100%	9.00E-122	100
Bifidobacterium	Bifidobacterium	100%	9.00E-122	100
Bifidobacterium	Bifidobacterium	100%	9.00E-122	100
Bifidobacterium	Bifidobacterium	100%	9.00E-122	100
Bifidobacterium	Bifidobacterium	100%	9.00E-122	100
Bifidobacterium	Bifidobacterium	100%	9.00E-122	100
Bifidobacterium	Bifidobacterium	100%	9.00E-122	100
Bifidobacterium	Bifidobacterium	100%	9.00E-122	100
Bifidobacterium	Bifidobacterium	100%	9.00E-122	100
Bifidobacterium	Bifidobacterium	100%	9.00E-122	100
Bifidobacterium	Bifidobacterium	100%	9.00E-122	100
Bifidobacterium	Bifidobacterium	100%	9.00E-122	100
Bifidobacterium	Bifidobacterium	100%	9.00E-122	100
Bifidobacterium	Bifidobacterium	100%	9.00E-122	100
Bifidobacterium	Bifidobacterium	100%	9.00E-122	100
Bifidobacterium	Bifidobacterium	100%	9.00E-122	100

Bifidobacterium	Bifidobacterium	100%	9.00E-122	100
Bifidobacterium	Bifidobacterium	100%	9.00E-122	100
Bifidobacterium	Bifidobacterium	100%	9.00E-122	100
Bifidobacterium	Bifidobacterium	100%	9.00E-122	100
Bifidobacterium	Bifidobacterium	100%	9.00E-122	100
Bifidobacterium	Bifidobacterium	100%	9.00E-122	100
Bifidobacterium	Bifidobacterium	100%	9.00E-122	100
Bifidobacterium	Bifidobacterium	100%	9.00E-122	100
Bifidobacterium	Bifidobacterium	100%	9.00E-122	100
Bifidobacterium	Bifidobacterium	100%	9.00E-122	100
Bifidobacterium	Bifidobacterium	100%	9.00E-122	100
Bifidobacterium	Bifidobacterium	100%	9.00E-122	100
Bifidobacterium	Bifidobacterium	100%	9.00E-122	100
Bifidobacterium	Bifidobacterium	100%	9.00E-122	100
Bifidobacterium	Bifidobacterium	100%	9.00E-122	100
Bifidobacterium	Bifidobacterium	100%	9.00E-122	100
Bifidobacterium	Bifidobacterium	100%	9.00E-122	100
Bifidobacterium	Bifidobacterium	100%	9.00E-122	100
Bifidobacterium	Bifidobacterium	100%	9.00E-122	100
Bifidobacterium	Bifidobacterium	100%	9.00E-122	100
Bifidobacterium	Bifidobacterium	100%	9.00E-122	100
Bifidobacterium	Bifidobacterium	100%	9.00E-122	100
Bifidobacterium	Bifidobacterium	100%	9.00E-122	100
Bifidobacterium	Bifidobacterium	100%	9.00E-122	100
Bifidobacterium	Bifidobacterium	100%	9.00E-122	100
Bifidobacterium	Bifidobacterium	100%	9.00E-122	100
Bifidobacterium	Bifidobacterium	100%	9.00E-122	100
Bifidobacterium	Bifidobacterium	100%	9.00E-122	100
Bifidobacterium	Bifidobacterium	100%	9.00E-122	100
Bifidobacterium	Bifidobacterium	100%	9.00E-122	100
Bifidobacterium	Bifidobacterium	100%	9.00E-122	100
Bifidobacterium	Bifidobacterium	100%	9.00E-122	100
Bifidobacterium	Bifidobacterium	100%	9.00E-122	100
Bifidobacterium	Bifidobacterium	100%	9.00E-122	100
Bifidobacterium	Bifidobacterium	100%	9.00E-122	100
Bifidobacterium	Bifidobacterium	100%	9.00E-122	100
Bifidobacterium	Bifidobacterium	100%	9.00E-122	100
Bifidobacterium	Bifidobacterium	100%	9.00E-122	100
Bifidobacterium	Bifidobacterium	100%	9.00E-122	100
Bifidobacterium	Bifidobacterium	100%	9.00E-122	100
Bifidobacterium	Bifidobacterium	100%	9.00E-122	100
Bifidobacterium	Bifidobacterium	100%	9.00E-122	100
Bifidobacterium	Bifidobacterium	100%	9.00E-122	100
Bifidobacterium	Bifidobacterium	100%	9.00E-122	100

Bifidobacterium	Bifidobacterium	100%	9.00E-122	100
Bifidobacterium	Bifidobacterium	100%	9.00E-122	100
Bifidobacterium	Bifidobacterium	100%	9.00E-122	100
Bifidobacterium	Bifidobacterium	100%	9.00E-122	100
Bifidobacterium	Bifidobacterium	100%	9.00E-122	100
Bifidobacterium	Bifidobacterium	100%	9.00E-122	100
Bifidobacterium	Bifidobacterium	100%	9.00E-122	100
Bifidobacterium	Bifidobacterium	100%	9.00E-122	100
Bifidobacterium	Bifidobacterium	100%	9.00E-122	100
Bifidobacterium	Bifidobacterium	100%	9.00E-122	100
Bifidobacterium	Bifidobacterium	100%	9.00E-122	100
Bifidobacterium	Bifidobacterium	100%	9.00E-122	100
Bifidobacterium	Bifidobacterium	100%	9.00E-122	100
Bifidobacterium	Bifidobacterium	100%	9.00E-122	100
Bifidobacterium	Bifidobacterium	100%	9.00E-122	100
Bifidobacterium	Bifidobacterium	100%	9.00E-122	100
Bifidobacterium	Bifidobacterium	100%	9.00E-122	100
Bifidobacterium	Bifidobacterium	100%	9.00E-122	100
Bifidobacterium	Bifidobacterium	100%	9.00E-122	100
Bifidobacterium	Bifidobacterium	100%	9.00E-122	100
Bifidobacterium	Bifidobacterium	100%	9.00E-122	100
Bifidobacterium	Bifidobacterium	100%	9.00E-122	100
Bifidobacterium	Bifidobacterium	100%	9.00E-122	100
Bifidobacterium	Bifidobacterium	100%	9.00E-122	100
Bifidobacterium	Bifidobacterium	100%	9.00E-122	100
Bifidobacterium	Bifidobacterium	100%	9.00E-122	100
Uncultured Bifid	uncultured Bifid	100%	9.00E-122	100
Bifidobacterium	Bifidobacterium	100%	9.00E-122	100
Bifidobacterium	Bifidobacterium	100%	9.00E-122	100
Bifidobacterium	Bifidobacterium	100%	9.00E-122	100
Bifidobacterium	Bifidobacterium	100%	9.00E-122	100
Bifidobacterium	Bifidobacterium	100%	9.00E-122	100
Bifidobacterium	Bifidobacterium	100%	9.00E-122	100
Bifidobacterium	Bifidobacterium	100%	9.00E-122	100
Bifidobacterium	Bifidobacterium	100%	9.00E-122	100
Bifidobacterium	Bifidobacterium	100%	9.00E-122	100
Bifidobacterium	Bifidobacterium	100%	9.00E-122	100
Bifidobacterium	Bifidobacterium	100%	9.00E-122	100
Bifidobacterium	Bifidobacterium	100%	9.00E-122	100
Bifidobacterium	Bifidobacterium	100%	9.00E-122	100
Bifidobacterium	Bifidobacterium	100%	9.00E-122	100
Bifidobacterium	Bifidobacterium	100%	9.00E-122	100
Bifidobacterium	Bifidobacterium	100%	9.00E-122	100
Bifidobacterium	Bifidobacterium	100%	9.00E-122	100

Bifidobacterium	Bifidobacterium	100%	9.00E-122	100
Bifidobacterium	Bifidobacterium	100%	9.00E-122	100
Bifidobacterium	Bifidobacterium	100%	9.00E-122	100
Bifidobacterium	Bifidobacterium	100%	9.00E-122	100
Bifidobacterium	Bifidobacterium	100%	9.00E-122	100
Bifidobacterium	Bifidobacterium	100%	9.00E-122	100
Bifidobacterium	Bifidobacterium	100%	9.00E-122	100
Bifidobacterium	Bifidobacterium	100%	9.00E-122	100
Bifidobacterium	Bifidobacterium	100%	9.00E-122	100
Bifidobacterium	Bifidobacterium	100%	9.00E-122	100
Bifidobacterium	Bifidobacterium	100%	9.00E-122	100
Bifidobacterium	Bifidobacterium	100%	9.00E-122	100
Bifidobacterium	Bifidobacterium	100%	9.00E-122	100
Bifidobacterium	Bifidobacterium	100%	9.00E-122	100
Bifidobacterium	Bifidobacterium	100%	9.00E-122	100
Bifidobacterium	Bifidobacterium	100%	9.00E-122	100
Bifidobacterium	Bifidobacterium	100%	9.00E-122	100
Bifidobacterium	Bifidobacterium	100%	9.00E-122	100
Bifidobacterium	Bifidobacterium	100%	9.00E-122	100
Bifidobacterium	Bifidobacterium	100%	9.00E-122	100
Bifidobacterium	Bifidobacterium	100%	9.00E-122	100
Bifidobacterium	Bifidobacterium	100%	9.00E-122	100
Bifidobacterium	Bifidobacterium	100%	9.00E-122	100
Bifidobacterium	Bifidobacterium	100%	9.00E-122	100
Bifidobacterium	Bifidobacterium	100%	9.00E-122	100
Bifidobacterium	Bifidobacterium	100%	9.00E-122	100
Bifidobacterium	Bifidobacterium	100%	9.00E-122	100
Uncultured Bific	uncultured Bifid	100%	9.00E-122	100
Bifidobacterium	Bifidobacterium	100%	9.00E-122	100
Bifidobacterium	Bifidobacterium	100%	9.00E-122	100
Bifidobacterium	Bifidobacterium	100%	9.00E-122	100
Bifidobacterium	Bifidobacterium	100%	9.00E-122	100
Bifidobacterium	Bifidobacterium	100%	9.00E-122	100
Bifidobacterium	Bifidobacterium	100%	9.00E-122	100
Bifidobacterium	Bifidobacterium	100%	9.00E-122	100
Bifidobacterium	Bifidobacterium	100%	9.00E-122	100
Bifidobacterium	Bifidobacterium	100%	9.00E-122	100
Uncultured Bific	uncultured Bifid	100%	9.00E-122	100
Uncultured Bific	uncultured Bifid	100%	9.00E-122	100
Bifidobacterium	Bifidobacterium	100%	9.00E-122	100
Bifidobacterium	Bifidobacterium	100%	9.00E-122	100
Bifidobacterium	Bifidobacterium	100%	9.00E-122	100
Uncultured Bific	uncultured Bifid	100%	9.00E-122	100
Uncultured Bific	uncultured Bifid	100%	9.00E-122	100

Uncultured Bific	uncultured Bifid	100%	9.00E-122	100
Uncultured Bific	uncultured Bifid	100%	9.00E-122	100
Uncultured Bific	uncultured Bifid	100%	9.00E-122	100
Bifidobacterium	Bifidobacterium	100%	9.00E-122	100
Bifidobacterium	Bifidobacterium	100%	9.00E-122	100
Uncultured Bific	uncultured Bifid	100%	9.00E-122	100
Bifidobacterium	Bifidobacterium	100%	9.00E-122	100
Bifidobacterium	Bifidobacterium	100%	9.00E-122	100
Bifidobacterium	Bifidobacterium	100%	9.00E-122	100
Bifidobacterium	Bifidobacterium	100%	9.00E-122	100
Bifidobacterium	Bifidobacterium	100%	9.00E-122	100
Bifidobacterium	Bifidobacterium	100%	9.00E-122	100
Bifidobacterium	Bifidobacterium	100%	9.00E-122	100
Bifidobacterium	Bifidobacterium	100%	9.00E-122	100
Bifidobacterium	Bifidobacterium	100%	9.00E-122	100
Bifidobacterium	Bifidobacterium	100%	9.00E-122	100
Bifidobacterium	Bifidobacterium	100%	9.00E-122	100
Bifidobacterium	Bifidobacterium	100%	9.00E-122	100
Bifidobacterium	Bifidobacterium	100%	9.00E-122	100
Bifidobacterium	Bifidobacterium	100%	9.00E-122	100
Bifidobacterium	Bifidobacterium	100%	9.00E-122	100
Bifidobacterium	Bifidobacterium	100%	9.00E-122	100
Bifidobacterium	Bifidobacterium	100%	9.00E-122	100
Bifidobacterium	Bifidobacterium	100%	9.00E-122	100
Bifidobacterium	Bifidobacterium	100%	9.00E-122	100
Bifidobacterium	Bifidobacterium	100%	9.00E-122	100
Bifidobacterium	Bifidobacterium	100%	9.00E-122	100
Bifidobacterium	Bifidobacterium	100%	9.00E-122	100
Bifidobacterium	Bifidobacterium	100%	9.00E-122	100
Bifidobacterium	Bifidobacterium	100%	9.00E-122	100
Bifidobacterium	Bifidobacterium	100%	9.00E-122	100
Bifidobacterium	Bifidobacterium	100%	9.00E-122	100
Bifidobacterium	Bifidobacterium	100%	9.00E-122	100
Bifidobacterium	Bifidobacterium	100%	9.00E-122	100
Bifidobacterium	Bifidobacterium	100%	9.00E-122	100
Bifidobacterium	Bifidobacterium	100%	9.00E-122	100
Bifidobacterium	Bifidobacterium	100%	9.00E-122	100
Bifidobacterium	Bifidobacterium	100%	9.00E-122	100
Bifidobacterium	Bifidobacterium	100%	9.00E-122	100
Bifidobacterium	Bifidobacterium	100%	9.00E-122	100
Bifidobacterium	Bifidobacterium	100%	9.00E-122	100
Bifidobacterium	Bifidobacterium	100%	9.00E-122	100
Bifidobacterium	Bifidobacterium	100%	9.00E-122	100
Bifidobacterium	Bifidobacterium	100%	9.00E-122	100

Bifidobacterium	Bifidobacterium	100%	9.00E-122	100
Bifidobacterium	Bifidobacterium	100%	9.00E-122	100
Bifidobacterium	Bifidobacterium	100%	9.00E-122	100
Bifidobacterium	Bifidobacterium	100%	9.00E-122	100
Bifidobacterium	Bifidobacterium	100%	9.00E-122	100
Bifidobacterium	Bifidobacterium	100%	9.00E-122	100
Bifidobacterium	Bifidobacterium	100%	9.00E-122	100
Bifidobacterium	Bifidobacterium	100%	9.00E-122	100
Bifidobacterium	Bifidobacterium	100%	9.00E-122	100
Bifidobacterium	Bifidobacterium	100%	9.00E-122	100
Bifidobacterium	Bifidobacterium	100%	9.00E-122	100
Bifidobacterium	Bifidobacterium	100%	9.00E-122	100
Bifidobacterium	Bifidobacterium	100%	9.00E-122	100
Bifidobacterium	Bifidobacterium	100%	9.00E-122	100
Bifidobacterium	Bifidobacterium	100%	9.00E-122	100
Bifidobacterium	Bifidobacterium	100%	9.00E-122	100
Bifidobacterium	Bifidobacterium	100%	9.00E-122	100
Bifidobacterium	Bifidobacterium	100%	9.00E-122	100
Bifidobacterium	Bifidobacterium	100%	9.00E-122	100
Bifidobacterium	Bifidobacterium	100%	9.00E-122	100
Bifidobacterium	Bifidobacterium	100%	9.00E-122	100
Bifidobacterium	Bifidobacterium	100%	9.00E-122	100
Bifidobacterium	Bifidobacterium	100%	9.00E-122	100
Bifidobacterium	Bifidobacterium	100%	9.00E-122	100
Bifidobacterium	Bifidobacterium	100%	9.00E-122	100
Bifidobacterium	Bifidobacterium	100%	9.00E-122	100
Bifidobacterium	Bifidobacterium	100%	9.00E-122	100
Bifidobacterium	Bifidobacterium	100%	9.00E-122	100
Bifidobacterium	Bifidobacterium	100%	9.00E-122	100
Bifidobacterium	Bifidobacterium	100%	9.00E-122	100
Bifidobacterium	Bifidobacterium	100%	9.00E-122	100
Bifidobacterium	Bifidobacterium	100%	9.00E-122	100
Bifidobacterium	Bifidobacterium	100%	9.00E-122	100
Bifidobacterium	Bifidobacterium	100%	9.00E-122	100
Bifidobacterium	Bifidobacterium	100%	9.00E-122	100
Bifidobacterium	Bifidobacterium	100%	9.00E-122	100
Bifidobacterium	Bifidobacterium	100%	9.00E-122	100
Bifidobacterium	Bifidobacterium	100%	9.00E-122	100
Bifidobacterium	Bifidobacterium	100%	9.00E-122	100
Bifidobacterium	Bifidobacterium	100%	9.00E-122	100
Bifidobacterium	Bifidobacterium	100%	9.00E-122	100
Bifidobacterium	Bifidobacterium	100%	9.00E-122	100
Bifidobacterium	Bifidobacterium	100%	9.00E-122	100
Bifidobacterium	Bifidobacterium	100%	9.00E-122	100

Bifidobacterium	Bifidobacterium	100%	9.00E-122	100
Bifidobacterium	Bifidobacterium	100%	9.00E-122	100
Bifidobacterium	Bifidobacterium	100%	9.00E-122	100
Bifidobacterium	Bifidobacterium	100%	9.00E-122	100
Bifidobacterium	Bifidobacterium	100%	9.00E-122	100
Bifidobacterium	Bifidobacterium	100%	9.00E-122	100
Bifidobacterium	Bifidobacterium	100%	9.00E-122	100
Bifidobacterium	Bifidobacterium	100%	9.00E-122	100
Bifidobacterium	Bifidobacterium	100%	9.00E-122	100
Bifidobacterium	Bifidobacterium	100%	9.00E-122	100
Bifidobacterium	Bifidobacterium	100%	9.00E-122	100
Bifidobacterium	Bifidobacterium	100%	9.00E-122	100
Bifidobacterium	Bifidobacterium	100%	9.00E-122	100
Bifidobacterium	Bifidobacterium	100%	9.00E-122	100
Bifidobacterium	Bifidobacterium	100%	9.00E-122	100
Bifidobacterium	Bifidobacterium	100%	9.00E-122	100
Bifidobacterium	Bifidobacterium	100%	9.00E-122	100
Bifidobacterium	Bifidobacterium	100%	9.00E-122	100
Bifidobacterium	Bifidobacterium	100%	9.00E-122	100
Bifidobacterium	Bifidobacterium	100%	9.00E-122	100
Bifidobacterium	Bifidobacterium	100%	9.00E-122	100
Bifidobacterium	Bifidobacterium	100%	9.00E-122	100
Bifidobacterium	Bifidobacterium	100%	9.00E-122	100
Bifidobacterium	Bifidobacterium	100%	9.00E-122	100
Bifidobacterium	Bifidobacterium	100%	9.00E-122	100
Bifidobacterium	Bifidobacterium	100%	9.00E-122	100
Bifidobacterium	Bifidobacterium	100%	9.00E-122	100
Bifidobacterium	Bifidobacterium	100%	9.00E-122	100
Bifidobacterium	Bifidobacterium	100%	9.00E-122	100
Bifidobacterium	Bifidobacterium	100%	9.00E-122	100
Bifidobacterium	Bifidobacterium	100%	9.00E-122	100
Bifidobacterium	Bifidobacterium	100%	9.00E-122	100
Bifidobacterium	Bifidobacterium	100%	9.00E-122	100
Bifidobacterium	Bifidobacterium	100%	9.00E-122	100
Bifidobacterium	Bifidobacterium	100%	9.00E-122	100
Bifidobacterium	Bifidobacterium	100%	9.00E-122	100
Bifidobacterium	Bifidobacterium	100%	9.00E-122	100
Bifidobacterium	Bifidobacterium	100%	9.00E-122	100
Bifidobacterium	Bifidobacterium	100%	9.00E-122	100
Bifidobacterium	Bifidobacterium	100%	9.00E-122	100
Bifidobacterium	Bifidobacterium	100%	9.00E-122	100
Bifidobacterium	Bifidobacterium	100%	9.00E-122	100
Bifidobacterium	Bifidobacterium	100%	9.00E-122	100
Bifidobacterium	Bifidobacterium	100%	9.00E-122	100

Bifidobacterium	Bifidobacterium	100%	9.00E-122	100
Bifidobacterium	Bifidobacterium	100%	9.00E-122	100
Bifidobacterium	Bifidobacterium	100%	9.00E-122	100
Bifidobacterium	Bifidobacterium	100%	9.00E-122	100
Bifidobacterium	Bifidobacterium	100%	9.00E-122	100
Bifidobacterium	Bifidobacterium	100%	9.00E-122	100
Bifidobacterium	Bifidobacterium	100%	9.00E-122	100
Bifidobacterium	Bifidobacterium	100%	9.00E-122	100
Bifidobacterium	Bifidobacterium	100%	9.00E-122	100
Bifidobacterium	Bifidobacterium	100%	9.00E-122	100
Bifidobacterium	Bifidobacterium	100%	9.00E-122	100
Bifidobacterium	Bifidobacterium	100%	9.00E-122	100
Bifidobacterium	Bifidobacterium	100%	9.00E-122	100
Bifidobacterium	Bifidobacterium	100%	9.00E-122	100
Bifidobacterium	Bifidobacterium	100%	9.00E-122	100
Bifidobacterium	Bifidobacterium	100%	9.00E-122	100
Bifidobacterium	Bifidobacterium	100%	9.00E-122	100
Bifidobacterium	Bifidobacterium	100%	9.00E-122	100
Bifidobacterium	Bifidobacterium	100%	9.00E-122	100
Bifidobacterium	Bifidobacterium	100%	9.00E-122	100
Bifidobacterium	Bifidobacterium	100%	9.00E-122	100
Bifidobacterium	Bifidobacterium	100%	9.00E-122	100
Bifidobacterium	Bifidobacterium	100%	9.00E-122	100
Bifidobacterium	Bifidobacterium	100%	9.00E-122	100
Bifidobacterium	Bifidobacterium	100%	9.00E-122	100
Bifidobacterium	Bifidobacterium	100%	9.00E-122	100
Bifidobacterium	Bifidobacterium	100%	9.00E-122	100
Bifidobacterium	Bifidobacterium	100%	9.00E-122	100
Bifidobacterium	Bifidobacterium	100%	9.00E-122	100
Uncultured Bifid	uncultured Bifid	100%	9.00E-122	100
Bifidobacterium	Bifidobacterium	100%	9.00E-122	100
Bifidobacterium	Bifidobacterium	100%	9.00E-122	100
Bifidobacterium	Bifidobacterium	100%	9.00E-122	100
Bifidobacterium	Bifidobacterium	100%	9.00E-122	100
Bifidobacterium	Bifidobacterium	100%	9.00E-122	100
Bifidobacterium	Bifidobacterium	100%	9.00E-122	100
Bifidobacterium	Bifidobacterium	100%	9.00E-122	100
Bifidobacterium	Bifidobacterium	100%	9.00E-122	100
Bifidobacterium	Bifidobacterium	100%	9.00E-122	100
Bifidobacterium	Bifidobacterium	100%	9.00E-122	100
Bifidobacterium	Bifidobacterium	100%	9.00E-122	100
Bifidobacterium	Bifidobacterium	100%	9.00E-122	100
Uncultured Bifid	uncultured Bifid	100%	9.00E-122	100
Bifidobacterium	Bifidobacterium	100%	9.00E-122	100

Bifidobacterium	Bifidobacterium	100%	9.00E-122	100
Bifidobacterium	Bifidobacterium	100%	9.00E-122	100
Bifidobacterium	Bifidobacterium	100%	9.00E-122	100
Bifidobacterium	Bifidobacterium	100%	9.00E-122	100
Bifidobacterium	Bifidobacterium	100%	9.00E-122	100
Bifidobacterium	Bifidobacterium	100%	9.00E-122	100
Bifidobacterium	Bifidobacterium	100%	9.00E-122	100
Bifidobacterium	Bifidobacterium	100%	9.00E-122	100
Bifidobacterium	Bifidobacterium	100%	9.00E-122	100
Bifidobacterium	Bifidobacterium	100%	9.00E-122	100
Bifidobacterium	Bifidobacterium	100%	9.00E-122	100
Bifidobacterium	Bifidobacterium	100%	9.00E-122	100
Bifidobacterium	Bifidobacterium	100%	9.00E-122	100
Bifidobacterium	Bifidobacterium	100%	9.00E-122	100
Bifidobacterium	Bifidobacterium	100%	9.00E-122	100
Bifidobacterium	Bifidobacterium	100%	9.00E-122	100
Bifidobacterium	Bifidobacterium	100%	9.00E-122	100
Bifidobacterium	Bifidobacterium	100%	9.00E-122	100
Bifidobacterium	Bifidobacterium	100%	9.00E-122	100
Bifidobacterium	Bifidobacterium	100%	9.00E-122	100
Bifidobacterium	Bifidobacterium	100%	9.00E-122	100
Bifidobacterium	Bifidobacterium	100%	9.00E-122	100
Bifidobacterium	Bifidobacterium	100%	9.00E-122	100
Bifidobacterium	Bifidobacterium	100%	9.00E-122	100
Bifidobacterium	Bifidobacterium	100%	9.00E-122	100
Bifidobacterium	Bifidobacterium	100%	9.00E-122	100
Bifidobacterium	Bifidobacterium	100%	9.00E-122	100
Bifidobacterium	Bifidobacterium	100%	9.00E-122	100
Uncultured Bific	uncultured Bifid	100%	9.00E-122	100
Bifidobacterium	Bifidobacterium	100%	9.00E-122	100
Bifidobacterium	Bifidobacterium	100%	9.00E-122	100
Bifidobacterium	Bifidobacterium	100%	9.00E-122	100
Bifidobacterium	Bifidobacterium	100%	9.00E-122	100
Bifidobacterium	Bifidobacterium	100%	9.00E-122	100
Bifidobacterium	Bifidobacterium	100%	9.00E-122	100
Bifidobacterium	Bifidobacterium	100%	9.00E-122	100
Bifidobacterium	Bifidobacterium	100%	9.00E-122	100
Bifidobacterium	Bifidobacterium	100%	9.00E-122	100
Bifidobacterium	Bifidobacterium	100%	9.00E-122	100
Bifidobacterium	Bifidobacterium	100%	9.00E-122	100
Bifidobacterium	Bifidobacterium	100%	9.00E-122	100
Bifidobacterium	Bifidobacterium	100%	9.00E-122	100
Bifidobacterium	Bifidobacterium	100%	9.00E-122	100
Bifidobacterium	Bifidobacterium	100%	9.00E-122	100

Bifidobacterium	Bifidobacterium	100%	9.00E-122	100
Bifidobacterium	Bifidobacterium	100%	9.00E-122	100
Bifidobacterium	Bifidobacterium	100%	9.00E-122	100
Bifidobacterium	Bifidobacterium	100%	9.00E-122	100
Bifidobacterium	Bifidobacterium	100%	9.00E-122	100
Bifidobacterium	Bifidobacterium	100%	9.00E-122	100
Bifidobacterium	Bifidobacterium	100%	9.00E-122	100
Bifidobacterium	Bifidobacterium	100%	9.00E-122	100
Bifidobacterium	Bifidobacterium	100%	9.00E-122	100
Bifidobacterium	Bifidobacterium	100%	9.00E-122	100
Bifidobacterium	Bifidobacterium	100%	9.00E-122	100
Bifidobacterium	Bifidobacterium	100%	9.00E-122	100
Bifidobacterium	Bifidobacterium	100%	9.00E-122	100
Bifidobacterium	Bifidobacterium	100%	9.00E-122	100
Bifidobacterium	Bifidobacterium	100%	9.00E-122	100
Uncultured Bific	uncultured Bifid	100%	9.00E-122	100
Bifidobacterium	Bifidobacterium	100%	9.00E-122	100
Bifidobacterium	Bifidobacterium	100%	9.00E-122	100
Bifidobacterium	Bifidobacterium	100%	9.00E-122	100
Bifidobacterium	Bifidobacterium	100%	9.00E-122	100
Bifidobacterium	Bifidobacterium	100%	9.00E-122	100
Bifidobacterium	Bifidobacterium	100%	9.00E-122	100
Bifidobacterium	Bifidobacterium	100%	9.00E-122	100
Bifidobacterium	Bifidobacterium	100%	9.00E-122	100
Bifidobacterium	Bifidobacterium	100%	9.00E-122	100
Bifidobacterium	Bifidobacterium	100%	9.00E-122	100
Bifidobacterium	Bifidobacterium	100%	9.00E-122	100
Bifidobacterium	Bifidobacterium	100%	9.00E-122	100
Bifidobacterium	Bifidobacterium	100%	9.00E-122	100
Bifidobacterium	Bifidobacterium	100%	9.00E-122	100
Bifidobacterium	Bifidobacterium	100%	9.00E-122	100
Bifidobacterium	Bifidobacterium	100%	9.00E-122	100
Bifidobacterium	Bifidobacterium	100%	9.00E-122	100
Bifidobacterium	Bifidobacterium	100%	9.00E-122	100
Bifidobacterium	Bifidobacterium	100%	9.00E-122	100
Bifidobacterium	Bifidobacterium	100%	9.00E-122	100
Bifidobacterium	Bifidobacterium	100%	9.00E-122	100
Bifidobacterium	Bifidobacterium	100%	9.00E-122	100
Bifidobacterium	Bifidobacterium	100%	9.00E-122	100
Bifidobacterium	Bifidobacterium	100%	9.00E-122	100
Bifidobacterium	Bifidobacterium	100%	9.00E-122	100
Bifidobacterium	Bifidobacterium	100%	9.00E-122	100
Bifidobacterium	Bifidobacterium	100%	9.00E-122	100
Uncultured Bific	uncultured Bifid	100%	9.00E-122	100

Bifidobacterium	Bifidobacterium	100%	9.00E-122	100
Bifidobacterium	Bifidobacterium	100%	9.00E-122	100
Bifidobacterium	Bifidobacterium	100%	9.00E-122	100
Bifidobacterium	Bifidobacterium	100%	9.00E-122	100
Bifidobacterium	Bifidobacterium	100%	9.00E-122	100
Bifidobacterium	Bifidobacterium	100%	9.00E-122	100
Bifidobacterium	Bifidobacterium	100%	9.00E-122	100
Bifidobacterium	Bifidobacterium	100%	9.00E-122	100
Bifidobacterium	Bifidobacterium	100%	9.00E-122	100
Uncultured Bific	uncultured Bifid	100%	9.00E-122	100
Uncultured Bific	uncultured Bifid	100%	9.00E-122	100
Uncultured Bific	uncultured Bifid	100%	9.00E-122	100
Uncultured Bific	uncultured Bifid	100%	9.00E-122	100
Uncultured Bific	uncultured Bifid	100%	9.00E-122	100
Uncultured Bific	uncultured Bifid	100%	9.00E-122	100
Uncultured Bific	uncultured Bifid	100%	9.00E-122	100
Uncultured Bific	uncultured Bifid	100%	9.00E-122	100
Uncultured Bific	uncultured Bifid	100%	9.00E-122	100
Uncultured Bific	uncultured Bifid	100%	9.00E-122	100
Uncultured Bific	uncultured Bifid	100%	9.00E-122	100
Bifidobacterium	Bifidobacterium	100%	9.00E-122	100
Uncultured Bific	uncultured Bifid	100%	9.00E-122	100
Uncultured Bific	uncultured Bifid	100%	9.00E-122	100
Uncultured Bific	uncultured Bifid	100%	9.00E-122	100
Uncultured Bific	uncultured Bifid	100%	9.00E-122	100
Uncultured Bific	uncultured Bifid	100%	9.00E-122	100
Bifidobacterium	Bifidobacterium	100%	9.00E-122	100
Bifidobacterium	Bifidobacterium	100%	9.00E-122	100
Bifidobacterium	Bifidobacterium	100%	9.00E-122	100
Bifidobacterium	Bifidobacterium	100%	9.00E-122	100
Bifidobacterium	Bifidobacterium	100%	9.00E-122	100
Bifidobacterium	Bifidobacterium	100%	9.00E-122	100
Bifidobacterium	Bifidobacterium	100%	9.00E-122	100
Bifidobacterium	Bifidobacterium	100%	9.00E-122	100
Bifidobacterium	Bifidobacterium	100%	9.00E-122	100
Bifidobacterium	Bifidobacterium	100%	9.00E-122	100
Bifidobacterium	Bifidobacterium	100%	9.00E-122	100
Bifidobacterium	Bifidobacterium	100%	9.00E-122	100
Bifidobacterium	Bifidobacterium	100%	9.00E-122	100
Bifidobacterium	Bifidobacterium	100%	9.00E-122	100
Bifidobacterium	Bifidobacterium	100%	9.00E-122	100
Bifidobacterium	Bifidobacterium	100%	9.00E-122	100
Bifidobacterium	Bifidobacterium	100%	9.00E-122	100
Bifidobacterium	Bifidobacterium	100%	9.00E-122	100

Bifidobacterium	Bifidobacterium	100%	9.00E-122	100
Bifidobacterium	Bifidobacterium	100%	9.00E-122	100
Uncultured Bifid	uncultured Bifid	100%	9.00E-122	100
Bifidobacterium	Bifidobacterium	100%	9.00E-122	100
Bifidobacterium	Bifidobacterium	100%	9.00E-122	100
Bifidobacterium	Bifidobacterium	100%	9.00E-122	100
Bifidobacterium	Bifidobacterium	100%	9.00E-122	100
Bifidobacterium	Bifidobacterium	100%	9.00E-122	100
Bifidobacterium	Bifidobacterium	100%	9.00E-122	100
Bifidobacterium	Bifidobacterium	100%	9.00E-122	100
Bifidobacterium	Bifidobacterium	100%	9.00E-122	100
Bifidobacterium	Bifidobacterium	100%	9.00E-122	100
Bifidobacterium	Bifidobacterium	100%	9.00E-122	100
Bifidobacterium	Bifidobacterium	100%	9.00E-122	100
Bifidobacterium	Bifidobacterium	99%	1.00E-120	100
Bifidobacterium	Bifidobacterium	99%	1.00E-120	100
Bifidobacterium	Bifidobacterium	99%	1.00E-120	100
Bifidobacterium	Bifidobacterium	99%	1.00E-120	100
Bifidobacterium	Bifidobacterium	99%	1.00E-120	100
Bifidobacterium	Bifidobacterium	99%	1.00E-120	100
Bifidobacterium	Bifidobacterium	99%	1.00E-120	100
Bifidobacterium	Bifidobacterium	99%	1.00E-120	100
Bifidobacterium	Bifidobacterium	99%	1.00E-120	100
Bifidobacterium	Bifidobacterium	99%	1.00E-120	100
Bifidobacterium	Bifidobacterium	99%	1.00E-120	100
Bifidobacterium	Bifidobacterium	100%	1.00E-120	99.58
Bifidobacterium	Bifidobacterium	100%	1.00E-120	99.58
Bifidobacterium	Bifidobacterium	99%	1.00E-120	100
Bifidobacterium	Bifidobacterium	99%	1.00E-120	100
Bifidobacterium	Bifidobacterium	99%	1.00E-120	100
Uncultured Bifid	uncultured Bifid	99%	1.00E-120	100
Bifidobacterium	Bifidobacterium	99%	1.00E-120	100
Bifidobacterium	Bifidobacterium	100%	1.00E-120	99.58
Bifidobacterium	Bifidobacterium	99%	1.00E-120	100
Bifidobacterium	Bifidobacterium	99%	1.00E-120	100
Bifidobacterium	Bifidobacterium	100%	1.00E-120	99.58
Bifidobacterium	Bifidobacterium	100%	4.00E-120	99.58
Bifidobacterium	Bifidobacterium	100%	4.00E-120	99.58
Bifidobacterium	Bifidobacterium	100%	4.00E-120	99.58
Bifidobacterium	Bifidobacterium	100%	4.00E-120	99.58
Bifidobacterium	Bifidobacterium	100%	4.00E-120	99.58
Bifidobacterium	Bifidobacterium	100%	4.00E-120	99.58
Bifidobacterium	Bifidobacterium	100%	4.00E-120	99.58
Bifidobacterium	Bifidobacterium	100%	4.00E-120	99.58

Bifidobacterium	Bifidobacterium	100%	4.00E-120	99.58
Bifidobacterium	Bifidobacterium	100%	4.00E-120	99.58
Bifidobacterium	Bifidobacterium	100%	4.00E-120	99.58
Bifidobacterium	Bifidobacterium	100%	4.00E-120	99.58
Bifidobacterium	Bifidobacterium	100%	4.00E-120	99.58
Bifidobacterium	Bifidobacterium	100%	4.00E-120	99.58
Bifidobacterium	Bifidobacterium	100%	4.00E-120	99.58
Bifidobacterium	Bifidobacterium	100%	4.00E-120	99.58
Bifidobacterium	Bifidobacterium	100%	4.00E-120	99.58
Bifidobacterium	Bifidobacterium	100%	4.00E-120	99.58
Bifidobacterium	Bifidobacterium	100%	4.00E-120	99.58
Bifidobacterium	Bifidobacterium	100%	4.00E-120	99.58
Bifidobacterium	Bifidobacterium	100%	4.00E-120	99.58
Bifidobacterium	Bifidobacterium	100%	4.00E-120	99.58
Bifidobacterium	Bifidobacterium	100%	4.00E-120	99.58
Bifidobacterium	Bifidobacterium	100%	4.00E-120	99.58
Bifidobacterium	Bifidobacterium	100%	4.00E-120	99.58
Bifidobacterium	Bifidobacterium	100%	4.00E-120	99.58
Bifidobacterium	Bifidobacterium	100%	4.00E-120	99.58
Bifidobacterium	Bifidobacterium	100%	4.00E-120	99.58
Bifidobacterium	Bifidobacterium	100%	4.00E-120	99.58
Bifidobacterium	Bifidobacterium	100%	4.00E-120	99.58
Bifidobacterium	Bifidobacterium	100%	4.00E-120	99.58
Bifidobacterium	Bifidobacterium	100%	4.00E-120	99.58
Bifidobacterium	Bifidobacterium	100%	4.00E-120	99.58
Bifidobacterium	Bifidobacterium	100%	4.00E-120	99.58
Bifidobacterium	Bifidobacterium	100%	4.00E-120	99.58
Bifidobacterium	Bifidobacterium	100%	4.00E-120	99.58
Bifidobacterium	Bifidobacterium	100%	4.00E-120	99.58
Bifidobacterium	Bifidobacterium	100%	4.00E-120	99.58
Bifidobacterium	Bifidobacterium	100%	4.00E-120	99.58
Bifidobacterium	Bifidobacterium	100%	4.00E-120	99.58
Bifidobacterium	Bifidobacterium	100%	4.00E-120	99.58
Bifidobacterium	Bifidobacterium	100%	4.00E-120	99.58
Bifidobacterium	Bifidobacterium	100%	4.00E-120	99.58
Bifidobacterium	Bifidobacterium	100%	4.00E-120	99.58
Bifidobacterium	Bifidobacterium	100%	4.00E-120	99.58
Bifidobacterium	Bifidobacterium	100%	4.00E-120	99.58
Bifidobacterium	Bifidobacterium	100%	4.00E-120	99.58
Bifidobacterium	Bifidobacterium	100%	4.00E-120	99.58
Bifidobacterium	Bifidobacterium	100%	4.00E-120	99.58
Bifidobacterium	Bifidobacterium	100%	4.00E-120	99.58
Bifidobacterium	Bifidobacterium	100%	4.00E-120	99.58
Bifidobacterium	Bifidobacterium	100%	4.00E-120	99.58

Bifidobacterium	Bifidobacterium	100%	4.00E-120	99.58
Bifidobacterium	Bifidobacterium	100%	4.00E-120	99.58
Bifidobacterium	Bifidobacterium	100%	4.00E-120	99.58
Bifidobacterium	Bifidobacterium	100%	4.00E-120	99.58
Uncultured Bifid	uncultured Bifid	100%	4.00E-120	99.58
Bifidobacterium	Bifidobacterium	100%	4.00E-120	99.58
Bifidobacterium	Bifidobacterium	100%	4.00E-120	99.58
Bifidobacterium	Bifidobacterium	100%	4.00E-120	99.58
Bifidobacterium	Bifidobacterium	100%	4.00E-120	99.58
Bifidobacterium	Bifidobacterium	100%	4.00E-120	99.58
Bifidobacterium	Bifidobacterium	100%	4.00E-120	99.58
Bifidobacterium	Bifidobacterium	100%	4.00E-120	99.58
Uncultured Bifid	uncultured Bifid	100%	4.00E-120	99.58
Uncultured Bifid	uncultured Bifid	100%	4.00E-120	99.58
Bifidobacterium	Bifidobacterium	100%	4.00E-120	99.58
Bifidobacterium	Bifidobacterium	100%	4.00E-120	99.58
Bifidobacterium	Bifidobacterium	100%	4.00E-120	99.58
Bifidobacterium	Bifidobacterium	100%	4.00E-120	99.58
Bifidobacterium	Bifidobacterium	100%	4.00E-120	99.58
Bifidobacterium	Bifidobacterium	100%	4.00E-120	99.58
Bifidobacterium	Bifidobacterium	100%	4.00E-120	99.58
Bifidobacterium	Bifidobacterium	100%	4.00E-120	99.58
Bifidobacterium	Bifidobacterium	100%	4.00E-120	99.58
Bifidobacterium	Bifidobacterium	100%	4.00E-120	99.58
Bifidobacterium	Bifidobacterium	100%	4.00E-120	99.58
Bifidobacterium	Bifidobacterium	100%	4.00E-120	99.58
Bifidobacterium	Bifidobacterium	100%	4.00E-120	99.58
Bifidobacterium	Bifidobacterium	100%	4.00E-120	99.58
Bifidobacterium	Bifidobacterium	100%	4.00E-120	99.58
Bifidobacterium	Bifidobacterium	100%	4.00E-120	99.58
Bifidobacterium	Bifidobacterium	100%	4.00E-120	99.58
Bifidobacterium	Bifidobacterium	100%	4.00E-120	99.58
Bifidobacterium	Bifidobacterium	100%	4.00E-120	99.58
Bifidobacterium	Bifidobacterium	100%	4.00E-120	99.58
Bifidobacterium	Bifidobacterium	100%	4.00E-120	99.58
Bifidobacterium	Bifidobacterium	100%	4.00E-120	99.58
Bifidobacterium	Bifidobacterium	100%	4.00E-120	99.58
Bifidobacterium	Bifidobacterium	100%	4.00E-120	99.58
Bifidobacterium	Bifidobacterium	100%	4.00E-120	99.58
Bifidobacterium	Bifidobacterium	100%	4.00E-120	99.58
Bifidobacterium	Bifidobacterium	100%	4.00E-120	99.58
Bifidobacterium	Bifidobacterium	100%	4.00E-120	99.58
Bifidobacterium	Bifidobacterium	100%	4.00E-120	99.58
Bifidobacterium	Bifidobacterium	100%	4.00E-120	99.58

Bifidobacterium	Bifidobacterium	100%	4.00E-120	99.58
Bifidobacterium	Bifidobacterium	100%	4.00E-120	99.58
Bifidobacterium	Bifidobacterium	100%	4.00E-120	99.58
Bifidobacterium	Bifidobacterium	100%	4.00E-120	99.58
Bifidobacterium	Bifidobacterium	100%	4.00E-120	99.58
Bifidobacterium	Bifidobacterium	100%	4.00E-120	99.58
Bifidobacterium	Bifidobacterium	100%	4.00E-120	99.58
Bifidobacterium	Bifidobacterium	100%	4.00E-120	99.58
Bifidobacterium	Bifidobacterium	100%	4.00E-120	99.58
Bifidobacterium	Bifidobacterium	100%	4.00E-120	99.58
Bifidobacterium	Bifidobacterium	100%	4.00E-120	99.58
Uncultured Bific	uncultured Bifid	100%	4.00E-120	99.58
Bifidobacterium	Bifidobacterium	100%	4.00E-120	99.58
Bifidobacterium	Bifidobacterium	100%	4.00E-120	99.58
Bifidobacterium	Bifidobacterium	100%	4.00E-120	99.58
Bifidobacterium	Bifidobacterium	100%	4.00E-120	99.58
Bifidobacterium	Bifidobacterium	100%	4.00E-120	99.58
Bifidobacterium	Bifidobacterium	100%	4.00E-120	99.58
Bifidobacterium	Bifidobacterium	100%	4.00E-120	99.58
Bifidobacterium	Bifidobacterium	100%	4.00E-120	99.58
Bifidobacterium	Bifidobacterium	100%	4.00E-120	99.58
Bifidobacterium	Bifidobacterium	100%	4.00E-120	99.58
Bifidobacterium	Bifidobacterium	100%	4.00E-120	99.58
Bifidobacterium	Bifidobacterium	100%	4.00E-120	99.58
Bifidobacterium	Bifidobacterium	100%	4.00E-120	99.58
Bifidobacterium	Bifidobacterium	100%	4.00E-120	99.58
Bifidobacterium	Bifidobacterium	100%	4.00E-120	99.58
Bifidobacterium	Bifidobacterium	100%	4.00E-120	99.58
Bifidobacterium	Bifidobacterium	100%	4.00E-120	99.58
Bifidobacterium	Bifidobacterium	100%	4.00E-120	99.58
Bifidobacterium	Bifidobacterium	100%	4.00E-120	99.58
Bifidobacterium	Bifidobacterium	100%	4.00E-120	99.58
Bifidobacterium	Bifidobacterium	100%	4.00E-120	99.58
Uncultured Bific	uncultured Bifid	100%	4.00E-120	99.58
Bifidobacterium	Bifidobacterium	100%	4.00E-120	99.58
Bifidobacterium	Bifidobacterium	100%	4.00E-120	99.58
Bifidobacterium	Bifidobacterium	100%	4.00E-120	99.58
Bifidobacterium	Bifidobacterium	100%	4.00E-120	99.58
Bifidobacterium	Bifidobacterium	100%	4.00E-120	99.58
Uncultured Bific	uncultured Bifid	100%	4.00E-120	99.58
Bifidobacterium	Bifidobacterium	100%	4.00E-120	99.58
Bifidobacterium	Bifidobacterium	100%	4.00E-120	99.58
Bifidobacterium	Bifidobacterium	100%	4.00E-120	99.58
Bifidobacterium	Bifidobacterium	100%	4.00E-120	99.58

Bifidobacterium	Bifidobacterium	100%	4.00E-120	99.58
Uncultured Bific	uncultured Bifid	100%	4.00E-120	99.58
Uncultured Bific	uncultured Bifid	100%	4.00E-120	99.58
Uncultured Bific	uncultured Bifid	100%	4.00E-120	99.58
Bifidobacterium	Bifidobacterium	100%	4.00E-120	99.58
Bifidobacterium	Bifidobacterium	100%	4.00E-120	99.58
Bifidobacterium	Bifidobacterium	100%	4.00E-120	99.58
Bifidobacterium	Bifidobacterium	100%	4.00E-120	99.58
Bifidobacterium	Bifidobacterium	100%	4.00E-120	99.58
Bifidobacterium	Bifidobacterium	100%	4.00E-120	99.58
Bifidobacterium	Bifidobacterium	100%	4.00E-120	99.58
Bifidobacterium	Bifidobacterium	100%	4.00E-120	99.58
Bifidobacterium	Bifidobacterium	100%	4.00E-120	99.58
Bifidobacterium	Bifidobacterium	100%	4.00E-120	99.58
Bifidobacterium	Bifidobacterium	100%	4.00E-120	99.58
Bifidobacterium	Bifidobacterium	100%	4.00E-120	99.58
Bifidobacterium	Bifidobacterium	100%	4.00E-120	99.58
Bifidobacterium	Bifidobacterium	100%	4.00E-120	99.58
Bifidobacterium	Bifidobacterium	100%	4.00E-120	99.58
Bifidobacterium	Bifidobacterium	100%	4.00E-120	99.58
Bifidobacterium	Bifidobacterium	100%	4.00E-120	99.58
Bifidobacterium	Bifidobacterium	100%	4.00E-120	99.58
Bifidobacterium	Bifidobacterium	100%	4.00E-120	99.58
Bifidobacterium	Bifidobacterium	100%	4.00E-120	99.58
Bifidobacterium	Bifidobacterium	100%	4.00E-120	99.58
Bifidobacterium	Bifidobacterium	100%	4.00E-120	99.58
Bifidobacterium	Bifidobacterium	100%	4.00E-120	99.58
Bifidobacterium	Bifidobacterium	100%	4.00E-120	99.58
Bifidobacterium	Bifidobacterium	100%	4.00E-120	99.58
Bifidobacterium	Bifidobacterium	100%	4.00E-120	99.58
Bifidobacterium	Bifidobacterium	100%	4.00E-120	99.58
Bifidobacterium	Bifidobacterium	100%	4.00E-120	99.58
Bifidobacterium	Bifidobacterium	100%	4.00E-120	99.58
Bifidobacterium	Bifidobacterium	100%	4.00E-120	99.58
Bifidobacterium	Bifidobacterium	100%	4.00E-120	99.58
Bifidobacterium	Bifidobacterium	100%	4.00E-120	99.58
Bifidobacterium	Bifidobacterium	100%	4.00E-120	99.58
Bifidobacterium	Bifidobacterium	100%	4.00E-120	99.58
Bifidobacterium	Bifidobacterium	100%	4.00E-120	99.58
Bifidobacterium	Bifidobacterium	100%	4.00E-120	99.58
Bifidobacterium	Bifidobacterium	100%	4.00E-120	99.58
Uncultured Bific	uncultured Bifid	100%	4.00E-120	99.58
Bifidobacterium	Bifidobacterium	100%	4.00E-120	99.58
Bifidobacterium	Bifidobacterium	100%	4.00E-120	99.58

Bifidobacterium	Bifidobacterium	100%	4.00E-120	99.58
Bifidobacterium	Bifidobacterium	100%	4.00E-120	99.58
Bifidobacterium	Bifidobacterium	100%	4.00E-120	99.58
Bifidobacterium	Bifidobacterium	100%	4.00E-120	99.58
Bifidobacterium	Bifidobacterium	100%	4.00E-120	99.58
Bifidobacterium	Bifidobacterium	100%	4.00E-120	99.58
Uncultured Bific	uncultured Bifid	100%	4.00E-120	99.58
Bifidobacterium	Bifidobacterium	100%	4.00E-120	99.58
Bifidobacterium	Bifidobacterium	100%	4.00E-120	99.58
Bifidobacterium	Bifidobacterium	100%	4.00E-120	99.58
Bifidobacterium	Bifidobacterium	100%	4.00E-120	99.58
Bifidobacterium	Bifidobacterium	100%	4.00E-120	99.58
Bifidobacterium	Bifidobacterium	100%	4.00E-120	99.58
Bifidobacterium	Bifidobacterium	100%	4.00E-120	99.58
Bifidobacterium	Bifidobacterium	100%	4.00E-120	99.58
Bifidobacterium	Bifidobacterium	100%	4.00E-120	99.58
Bifidobacterium	Bifidobacterium	100%	4.00E-120	99.58
Bifidobacterium	Bifidobacterium	100%	4.00E-120	99.58
Bifidobacterium	Bifidobacterium	100%	4.00E-120	99.58
Bifidobacterium	Bifidobacterium	100%	4.00E-120	99.58
Bifidobacterium	Bifidobacterium	100%	4.00E-120	99.58
Bifidobacterium	Bifidobacterium	100%	4.00E-120	99.58
Bifidobacterium	Bifidobacterium	100%	4.00E-120	99.58
Bifidobacterium	Bifidobacterium	100%	4.00E-120	99.58
Bifidobacterium	Bifidobacterium	100%	4.00E-120	99.58
Bifidobacterium	Bifidobacterium	100%	4.00E-120	99.58
Bifidobacterium	Bifidobacterium	100%	4.00E-120	99.58
Bifidobacterium	Bifidobacterium	100%	4.00E-120	99.58
Bifidobacterium	Bifidobacterium	100%	4.00E-120	99.58
Bifidobacterium	Bifidobacterium	100%	4.00E-120	99.58
Bifidobacterium	Bifidobacterium	100%	4.00E-120	99.58
Bifidobacterium	Bifidobacterium	100%	4.00E-120	99.58
Bifidobacterium	Bifidobacterium	100%	4.00E-120	99.58
Bifidobacterium	Bifidobacterium	100%	4.00E-120	99.58
Bifidobacterium	Bifidobacterium	100%	4.00E-120	99.58
Bifidobacterium	Bifidobacterium	100%	4.00E-120	99.58
Bifidobacterium	Bifidobacterium	100%	4.00E-120	99.58
Bifidobacterium	Bifidobacterium	100%	4.00E-120	99.58
Bifidobacterium	Bifidobacterium	100%	4.00E-120	99.58
Bifidobacterium	Bifidobacterium	100%	4.00E-120	99.58
Bifidobacterium	Bifidobacterium	100%	4.00E-120	99.58
Bifidobacterium	Bifidobacterium	100%	4.00E-120	99.58
Bifidobacterium	Bifidobacterium	100%	4.00E-120	99.58
Bifidobacterium	Bifidobacterium	100%	4.00E-120	99.58
Bifidobacterium	Bifidobacterium	98%	4.00E-120	100

Bifidobacterium	Bifidobacterium	100%	4.00E-120	99.58
Bifidobacterium	Bifidobacterium	100%	4.00E-120	99.58
Bifidobacterium	Bifidobacterium	100%	4.00E-120	99.58
Bifidobacterium	Bifidobacterium	100%	4.00E-120	99.58
Bifidobacterium	Bifidobacterium	100%	4.00E-120	99.58
Uncultured Bifid	uncultured Bifid	100%	4.00E-120	99.58
Bifidobacterium	Bifidobacterium	98%	4.00E-120	100
Bifidobacterium	Bifidobacterium	100%	4.00E-120	99.58
Uncultured Bifid	uncultured Bifid	100%	4.00E-120	99.58
Uncultured Bifid	uncultured Bifid	100%	4.00E-120	99.58
Uncultured Bifid	uncultured Bifid	100%	4.00E-120	99.58
Uncultured Bifid	uncultured Bifid	100%	4.00E-120	99.58
Uncultured Bifid	uncultured Bifid	100%	4.00E-120	99.58
Bifidobacterium	Bifidobacterium	100%	4.00E-120	99.58
Bifidobacterium	Bifidobacterium	100%	4.00E-120	99.58
Bifidobacterium	Bifidobacterium	100%	4.00E-120	99.58
Bifidobacterium	Bifidobacterium	100%	4.00E-120	99.58
Bifidobacterium	Bifidobacterium	100%	4.00E-120	99.58
Bifidobacterium	Bifidobacterium	100%	4.00E-120	99.58
Bifidobacterium	Bifidobacterium	100%	4.00E-120	99.58
Bifidobacterium	Bifidobacterium	100%	4.00E-120	99.58
Bifidobacterium	Bifidobacterium	100%	4.00E-120	99.58
Bifidobacterium	Bifidobacterium	100%	4.00E-120	99.58
Bifidobacterium	Bifidobacterium	100%	4.00E-120	99.58
Bifidobacterium	Bifidobacterium	100%	4.00E-120	99.58
Bifidobacterium	Bifidobacterium	100%	4.00E-120	99.58
Bifidobacterium	Bifidobacterium	100%	4.00E-120	99.58
Bifidobacterium	Bifidobacterium	100%	4.00E-120	99.58
Bifidobacterium	Bifidobacterium	100%	4.00E-120	99.58
Bifidobacterium	Bifidobacterium	100%	4.00E-120	99.58
Bifidobacterium	Bifidobacterium	100%	4.00E-120	99.58
Bifidobacterium	Bifidobacterium	100%	4.00E-120	99.58
Uncultured Bifid	uncultured Bifid	100%	4.00E-120	99.58
Uncultured Bifid	uncultured Bifid	100%	4.00E-120	99.58
Uncultured Bifid	uncultured Bifid	100%	4.00E-120	99.58
Uncultured Bifid	uncultured Bifid	100%	4.00E-120	99.58
Uncultured Bifid	uncultured Bifid	100%	4.00E-120	99.58
Uncultured Bifid	uncultured Bifid	100%	4.00E-120	99.58
Bifidobacterium	Bifidobacterium	100%	4.00E-120	99.58
Bifidobacterium	Bifidobacterium	100%	4.00E-120	99.58
Bifidobacterium	Bifidobacterium	100%	4.00E-120	99.58
Bifidobacterium	Bifidobacterium	100%	4.00E-120	99.58
Uncultured Bifid	uncultured Bifid	100%	4.00E-120	99.58
Bifidobacterium	Bifidobacterium	100%	4.00E-120	99.58

Bifidobacterium	Bifidobacterium	100%	4.00E-120	99.58
Bifidobacterium	Bifidobacterium	100%	4.00E-120	99.58
Bifidobacterium	Bifidobacterium	100%	4.00E-120	99.58
Bifidobacterium	Bifidobacterium	100%	4.00E-120	99.58
Bifidobacterium	Bifidobacterium	100%	4.00E-120	99.58
Bifidobacterium	Bifidobacterium	100%	4.00E-120	99.58
Bifidobacterium	Bifidobacterium	100%	4.00E-120	99.58
Bifidobacterium	Bifidobacterium	100%	4.00E-120	99.58
Bifidobacterium	Bifidobacterium	100%	4.00E-120	99.58
Bifidobacterium	Bifidobacterium	100%	4.00E-120	99.58
Bifidobacterium	Bifidobacterium	100%	4.00E-120	99.58
Bifidobacterium	Bifidobacterium	100%	4.00E-120	99.58
Bifidobacterium	Bifidobacterium	100%	4.00E-120	99.58
Bifidobacterium	Bifidobacterium	100%	4.00E-120	99.58
Bifidobacterium	Bifidobacterium	100%	4.00E-120	99.58
Bifidobacterium	Bifidobacterium	100%	4.00E-120	99.58
Bifidobacterium	Bifidobacterium	100%	4.00E-120	99.58
Bifidobacterium	Bifidobacterium	100%	4.00E-120	99.58
Bifidobacterium	Bifidobacterium	100%	4.00E-120	99.58
Bifidobacterium	Bifidobacterium	100%	4.00E-120	99.58
Bifidobacterium	Bifidobacterium	100%	4.00E-120	99.58
Bifidobacterium	Bifidobacterium	100%	4.00E-120	99.58
Bifidobacterium	Bifidobacterium	100%	4.00E-120	99.58
Bifidobacterium	Bifidobacterium	100%	4.00E-120	99.58
Uncultured Bific	uncultured Bifid	100%	4.00E-120	99.58
Bifidobacterium	Bifidobacterium	100%	4.00E-120	99.58
Bifidobacterium	Bifidobacterium	100%	4.00E-120	99.58
Bifidobacterium	Bifidobacterium	100%	4.00E-120	99.58
Uncultured Bific	uncultured Bifid	100%	4.00E-120	99.58
Bifidobacterium	Bifidobacterium	100%	4.00E-120	99.58
Bifidobacterium	Bifidobacterium	100%	4.00E-120	99.58
Bifidobacterium	Bifidobacterium	100%	4.00E-120	99.58
Bifidobacterium	Bifidobacterium	100%	4.00E-120	99.58
Bifidobacterium	Bifidobacterium	100%	4.00E-120	99.58
Bifidobacterium	Bifidobacterium	100%	4.00E-120	99.58
Bifidobacterium	Bifidobacterium	100%	4.00E-120	99.58
Bifidobacterium	Bifidobacterium	100%	4.00E-120	99.58
Bifidobacterium	Bifidobacterium	100%	4.00E-120	99.58
Bifidobacterium	Bifidobacterium	100%	4.00E-120	99.58
Bifidobacterium	Bifidobacterium	100%	4.00E-120	99.58
Bifidobacterium	Bifidobacterium	100%	4.00E-120	99.58
Bifidobacterium	Bifidobacterium	100%	4.00E-120	99.58
Bifidobacterium	Bifidobacterium	100%	4.00E-120	99.58
Bifidobacterium	Bifidobacterium	100%	4.00E-120	99.58

Bifidobacterium	Bifidobacterium	100%	4.00E-120	99.58
Uncultured Bific	uncultured Bifid	100%	4.00E-120	99.58
Bifidobacterium	Bifidobacterium	100%	4.00E-120	99.58
Bifidobacterium	Bifidobacterium	100%	4.00E-120	99.58
Bifidobacterium	Bifidobacterium	100%	4.00E-120	99.58
Bifidobacterium	Bifidobacterium	100%	4.00E-120	99.58
Bifidobacterium	Bifidobacterium	100%	4.00E-120	99.58
Bifidobacterium	Bifidobacterium	100%	4.00E-120	99.58
Bifidobacterium	Bifidobacterium	100%	4.00E-120	99.58
Bifidobacterium	Bifidobacterium	100%	4.00E-120	99.58
Bifidobacterium	Bifidobacterium	100%	4.00E-120	99.58
Bifidobacterium	Bifidobacterium	100%	4.00E-120	99.58
Bifidobacterium	Bifidobacterium	100%	4.00E-120	99.58
Bifidobacterium	Bifidobacterium	100%	4.00E-120	99.58
Bifidobacterium	Bifidobacterium	100%	4.00E-120	99.58
Bifidobacterium	Bifidobacterium	100%	4.00E-120	99.58
Bifidobacterium	Bifidobacterium	100%	4.00E-120	99.58
Bifidobacterium	Bifidobacterium	100%	4.00E-120	99.58
Bifidobacterium	Bifidobacterium	100%	4.00E-120	99.58
Bifidobacterium	Bifidobacterium	100%	4.00E-120	99.58
Uncultured Bific	uncultured Bifid	100%	4.00E-120	99.58
Bifidobacterium	Bifidobacterium	100%	4.00E-120	99.58
Bifidobacterium	Bifidobacterium	100%	4.00E-120	99.58
Bifidobacterium	Bifidobacterium	100%	4.00E-120	99.58
Bifidobacterium	Bifidobacterium	100%	4.00E-120	99.58
Bifidobacterium	Bifidobacterium	100%	4.00E-120	99.58
Bifidobacterium	Bifidobacterium	100%	4.00E-120	99.58
Bifidobacterium	Bifidobacterium	100%	4.00E-120	99.58
Bifidobacterium	Bifidobacterium	100%	4.00E-120	99.58
Bifidobacterium	Bifidobacterium	100%	4.00E-120	99.58
Uncultured Bific	uncultured Bifid	100%	4.00E-120	99.58
Bifidobacterium	Bifidobacterium	100%	4.00E-120	99.58
Bifidobacterium	Bifidobacterium	100%	4.00E-120	99.58
Bifidobacterium	Bifidobacterium	100%	4.00E-120	99.58
Uncultured Bific	uncultured Bifid	100%	4.00E-120	99.58
Uncultured Bific	uncultured Bifid	100%	4.00E-120	99.58
Uncultured Bific	uncultured Bifid	100%	4.00E-120	99.58
Uncultured Bific	uncultured Bifid	100%	4.00E-120	99.58
Uncultured Bific	uncultured Bifid	100%	4.00E-120	99.58
Uncultured Bific	uncultured Bifid	100%	4.00E-120	99.58
Uncultured Bific	uncultured Bifid	100%	4.00E-120	99.58
Bifidobacterium	Bifidobacterium	100%	4.00E-120	99.58
Uncultured Bific	uncultured Bifid	100%	4.00E-120	99.58
Uncultured Bific	uncultured Bifid	100%	4.00E-120	99.58

Uncultured Bifid	uncultured Bifid	100%	4.00E-120	99.58
Uncultured Bifid	uncultured Bifid	100%	4.00E-120	99.58
Uncultured Bifid	uncultured Bifid	100%	4.00E-120	99.58
Uncultured Bifid	uncultured Bifid	100%	4.00E-120	99.58
Uncultured Bifid	uncultured Bifid	100%	4.00E-120	99.58
Bifidobacterium	Bifidobacterium	100%	4.00E-120	99.58
Bifidobacterium	Bifidobacterium	100%	4.00E-120	99.58
Bifidobacterium	Bifidobacterium	100%	4.00E-120	99.58
Bifidobacterium	Bifidobacterium	100%	4.00E-120	99.58
Bifidobacterium	Bifidobacterium	100%	4.00E-120	99.58
Bifidobacterium	Bifidobacterium	100%	4.00E-120	99.58
Uncultured Bifid	uncultured Bifid	100%	4.00E-120	99.58
Uncultured Bifid	uncultured Bifid	100%	4.00E-120	99.58
Bifidobacterium	Bifidobacterium	100%	4.00E-120	99.58
Bifidobacterium	Bifidobacterium	100%	4.00E-120	99.58
Bifidobacterium	Bifidobacterium	100%	4.00E-120	99.58
Bifidobacterium	Bifidobacterium	100%	4.00E-120	99.58
Bifidobacterium	Bifidobacterium	100%	4.00E-120	99.58
Uncultured Bifid	uncultured Bifid	100%	4.00E-120	99.58
Bifidobacterium	Bifidobacterium	100%	4.00E-120	99.58
Bifidobacterium	Bifidobacterium	100%	4.00E-120	99.58
Uncultured Bifid	uncultured Bifid	100%	4.00E-120	99.58
Bifidobacterium	Bifidobacterium	100%	4.00E-120	99.58
Bifidobacterium	Bifidobacterium	100%	4.00E-120	99.58
Bifidobacterium	Bifidobacterium	100%	4.00E-120	99.58
Bifidobacterium	Bifidobacterium	100%	4.00E-120	99.58
Bifidobacterium	Bifidobacterium	100%	4.00E-120	99.58
Bifidobacterium	Bifidobacterium	100%	4.00E-120	99.58
Bifidobacterium	Bifidobacterium	100%	4.00E-120	99.58
Bifidobacterium	Bifidobacterium	100%	4.00E-120	99.58
Bifidobacterium	Bifidobacterium	100%	4.00E-120	99.58
Bifidobacterium	Bifidobacterium	100%	4.00E-120	99.58
Bifidobacterium	Bifidobacterium	100%	4.00E-120	99.58
Bifidobacterium	Bifidobacterium	100%	4.00E-120	99.58
Bifidobacterium	Bifidobacterium	100%	4.00E-120	99.58
Bifidobacterium	Bifidobacterium	100%	2.00E-119	99.58
Bifidobacterium	Bifidobacterium	100%	2.00E-119	99.58
Bifidobacterium	Bifidobacterium	100%	2.00E-119	99.58
Uncultured Bifid	uncultured Bifid	99%	2.00E-119	99.57
Bifidobacterium	Bifidobacterium	100%	2.00E-119	99.58
Bifidobacterium	Bifidobacterium	100%	2.00E-119	99.58
Bifidobacterium	Bifidobacterium	100%	2.00E-119	99.16
Bifidobacterium	Bifidobacterium	100%	2.00E-119	99.58
Bifidobacterium	Bifidobacterium	100%	2.00E-119	99.58

Bifidobacterium	Bifidobacterium	99%	2.00E-119	99.57
Bifidobacterium	Bifidobacterium	100%	2.00E-119	99.58
Bifidobacterium	Bifidobacterium	100%	2.00E-119	99.58
Bifidobacterium	Bifidobacterium	100%	2.00E-119	99.58
Bifidobacterium	Bifidobacterium	99%	2.00E-119	99.58
Bifidobacterium	Bifidobacterium	98%	2.00E-119	100
B.breve (CIP 64	Bifidobacterium	100%	2.00E-119	99.16
Bifidobacterium	Bifidobacterium	99%	6.00E-119	99.57
Bifidobacterium	Bifidobacterium	100%	6.00E-119	99.16
Uncultured Bific	uncultured Bifid	97%	6.00E-119	100
Uncultured Bific	uncultured Bifid	97%	6.00E-119	100
Bifidobacterium	Bifidobacterium	100%	6.00E-119	99.16
Bifidobacterium	Bifidobacterium	100%	6.00E-119	99.16
Bifidobacterium	Bifidobacterium	100%	6.00E-119	99.16
Bifidobacterium	Bifidobacterium	100%	6.00E-119	99.16
Bifidobacterium	Bifidobacterium	99%	6.00E-119	99.57
Bifidobacterium	Bifidobacterium	100%	6.00E-119	99.16
Bifidobacterium	Bifidobacterium	100%	6.00E-119	99.16
Bifidobacterium	Bifidobacterium	100%	6.00E-119	99.16
Bifidobacterium	Bifidobacterium	100%	6.00E-119	99.16
Bifidobacterium	Bifidobacterium	100%	6.00E-119	99.16
Bifidobacterium	Bifidobacterium	100%	6.00E-119	99.16
Uncultured Bific	uncultured Bifid	99%	6.00E-119	99.57
Uncultured Bific	uncultured Bifid	100%	6.00E-119	99.16
Uncultured Bific	uncultured Bifid	100%	6.00E-119	99.16
Uncultured Bific	uncultured Bifid	100%	6.00E-119	99.16
Bifidobacterium	Bifidobacterium	100%	2.00E-118	99.16
Bifidobacterium	Bifidobacterium	100%	2.00E-118	99.16
Bifidobacterium	Bifidobacterium	100%	2.00E-118	99.16
Bifidobacterium	Bifidobacterium	100%	2.00E-118	99.16
Bifidobacterium	Bifidobacterium	100%	2.00E-118	99.16
Bifidobacterium	Bifidobacterium	100%	2.00E-118	99.16
Bifidobacterium	Bifidobacterium	100%	2.00E-118	99.16
Bifidobacterium	Bifidobacterium	100%	2.00E-118	99.16
Bifidobacterium	Bifidobacterium	100%	2.00E-118	99.16
Bifidobacterium	Bifidobacterium	100%	2.00E-118	99.16
Bifidobacterium	Bifidobacterium	100%	2.00E-118	99.16
Bifidobacterium	Bifidobacterium	100%	2.00E-118	99.16
Bifidobacterium	Bifidobacterium	100%	2.00E-118	99.16
Bifidobacterium	Bifidobacterium	100%	2.00E-118	99.16
Bifidobacterium	Bifidobacterium	100%	2.00E-118	99.16
Bifidobacterium	Bifidobacterium	100%	2.00E-118	99.16
Bifidobacterium	Bifidobacterium	100%	2.00E-118	99.16
Bifidobacterium	Bifidobacterium	100%	2.00E-118	99.16

Bifidobacterium	Bifidobacterium	100%	2.00E-118	99.16
Bifidobacterium	Bifidobacterium	100%	2.00E-118	99.16
Bifidobacterium	Bifidobacterium	100%	2.00E-118	99.16
Bifidobacterium	Bifidobacterium	100%	2.00E-118	99.16
Bifidobacterium	Bifidobacterium	100%	2.00E-118	99.16
Bifidobacterium	Bifidobacterium	100%	2.00E-118	99.16
Bifidobacterium	Bifidobacterium	100%	2.00E-118	99.16
Bifidobacterium	Bifidobacterium	100%	2.00E-118	99.16
Bifidobacterium	Bifidobacterium	100%	2.00E-118	99.16
Bifidobacterium	Bifidobacterium	100%	2.00E-118	99.16
Bifidobacterium	Bifidobacterium	100%	2.00E-118	99.16
Bifidobacterium	Bifidobacterium	100%	2.00E-118	99.16
Bifidobacterium	Bifidobacterium	100%	2.00E-118	99.16
Bifidobacterium	Bifidobacterium	100%	2.00E-118	99.16
Bifidobacterium	Bifidobacterium	100%	2.00E-118	99.16
Bifidobacterium	Bifidobacterium	100%	2.00E-118	99.16
Bifidobacterium	Bifidobacterium	100%	2.00E-118	99.16
Bifidobacterium	Bifidobacterium	100%	2.00E-118	99.16
Bifidobacterium	Bifidobacterium	100%	2.00E-118	99.16
Bifidobacterium	Bifidobacterium	100%	2.00E-118	99.16
Bifidobacterium	Bifidobacterium	100%	2.00E-118	99.16
Bifidobacterium	Bifidobacterium	100%	2.00E-118	99.16
Bifidobacterium	Bifidobacterium	100%	2.00E-118	99.16
Bifidobacterium	Bifidobacterium	100%	2.00E-118	99.16
Bifidobacterium	Bifidobacterium	100%	2.00E-118	99.16
Bifidobacterium	Bifidobacterium	100%	2.00E-118	99.16
Bifidobacterium	Bifidobacterium	100%	2.00E-118	99.16
Bifidobacterium	Bifidobacterium	100%	2.00E-118	99.16
Bifidobacterium	Bifidobacterium	100%	2.00E-118	99.16
Bifidobacterium	Bifidobacterium	100%	2.00E-118	99.16
Bifidobacterium	Bifidobacterium	100%	2.00E-118	99.16
Bifidobacterium	Bifidobacterium	100%	2.00E-118	99.16
Bifidobacterium	Bifidobacterium	100%	2.00E-118	99.16
Bifidobacterium	Bifidobacterium	100%	2.00E-118	99.16
Bifidobacterium	Bifidobacterium	100%	2.00E-118	99.16
Bifidobacterium	Bifidobacterium	100%	2.00E-118	99.16
Bifidobacterium	Bifidobacterium	100%	2.00E-118	99.16
Bifidobacterium	Bifidobacterium	100%	2.00E-118	99.16
Bifidobacterium	Bifidobacterium	100%	2.00E-118	99.16
Bifidobacterium	Bifidobacterium	100%	2.00E-118	99.16
Bifidobacterium	Bifidobacterium	100%	2.00E-118	99.16
Bifidobacterium	Bifidobacterium	100%	2.00E-118	99.16
Bifidobacterium	Bifidobacterium	100%	2.00E-118	99.16
Bifidobacterium	Bifidobacterium	100%	2.00E-118	99.16

Bifidobacterium	Bifidobacterium	100%	2.00E-118	99.16
Bifidobacterium	Bifidobacterium	100%	2.00E-118	99.16
Bifidobacterium	Bifidobacterium	100%	2.00E-118	99.16
Bifidobacterium	Bifidobacterium	100%	2.00E-118	99.16
Bifidobacterium	Bifidobacterium	100%	2.00E-118	99.16
Bifidobacterium	Bifidobacterium	100%	2.00E-118	99.16
Bifidobacterium	Bifidobacterium	100%	2.00E-118	99.16
Bifidobacterium	Bifidobacterium	100%	2.00E-118	99.16
Bifidobacterium	Bifidobacterium	100%	2.00E-118	99.16
Bifidobacterium	Bifidobacterium	100%	2.00E-118	99.16
Bifidobacterium	Bifidobacterium	100%	2.00E-118	99.16
Bifidobacterium	Bifidobacterium	100%	2.00E-118	99.16
Bifidobacterium	Bifidobacterium	100%	2.00E-118	99.16
Bifidobacterium	Bifidobacterium	100%	2.00E-118	99.16
Bifidobacterium	Bifidobacterium	100%	2.00E-118	99.16
Bifidobacterium	Bifidobacterium	100%	2.00E-118	99.16
Bifidobacterium	Bifidobacterium	100%	2.00E-118	99.16
Bifidobacterium	Bifidobacterium	100%	2.00E-118	99.16
Bifidobacterium	Bifidobacterium	100%	2.00E-118	99.16
Bifidobacterium	Bifidobacterium	100%	2.00E-118	99.16
Bifidobacterium	Bifidobacterium	100%	2.00E-118	99.16
Bifidobacterium	Bifidobacterium	100%	2.00E-118	99.16
Bifidobacterium	Bifidobacterium	100%	2.00E-118	99.16
Bifidobacterium	Bifidobacterium	100%	2.00E-118	99.16
Bifidobacterium	Bifidobacterium	100%	2.00E-118	99.16
Bifidobacterium	Bifidobacterium	100%	2.00E-118	99.16
Bifidobacterium	Bifidobacterium	100%	2.00E-118	99.16
Bifidobacterium	Bifidobacterium	100%	2.00E-118	99.16
Bifidobacterium	Bifidobacterium	100%	2.00E-118	99.16
Bifidobacterium	Bifidobacterium	100%	2.00E-118	99.16
Bifidobacterium	Bifidobacterium	100%	2.00E-118	99.16
Bifidobacterium	Bifidobacterium	100%	2.00E-118	99.16
Bifidobacterium	Bifidobacterium	100%	2.00E-118	99.16
Bifidobacterium	Bifidobacterium	100%	2.00E-118	99.16
Bifidobacterium	Bifidobacterium	100%	2.00E-118	99.16
Bifidobacterium	Bifidobacterium	100%	2.00E-118	99.16
Bifidobacterium	Bifidobacterium	100%	2.00E-118	99.16
Bifidobacterium	Bifidobacterium	100%	2.00E-118	99.16
Bifidobacterium	Bifidobacterium	100%	2.00E-118	99.16
Bifidobacterium	Bifidobacterium	100%	2.00E-118	99.16
Bifidobacterium	Bifidobacterium	100%	2.00E-118	99.16
Bifidobacterium	Bifidobacterium	100%	2.00E-118	99.16
Bifidobacterium	Bifidobacterium	100%	2.00E-118	99.16
Bifidobacterium	Bifidobacterium	100%	2.00E-118	99.16

Bifidobacterium	Bifidobacterium	100%	2.00E-118	99.16
Bifidobacterium	Bifidobacterium	100%	2.00E-118	99.16
Bifidobacterium	Bifidobacterium	100%	2.00E-118	99.16
Bifidobacterium	Bifidobacterium	100%	2.00E-118	99.16
Bifidobacterium	Bifidobacterium	100%	2.00E-118	99.16
Bifidobacterium	Bifidobacterium	100%	2.00E-118	99.16
Bifidobacterium	Bifidobacterium	100%	2.00E-118	99.16
Bifidobacterium	Bifidobacterium	100%	2.00E-118	99.16
Bifidobacterium	Bifidobacterium	100%	2.00E-118	99.16
Bifidobacterium	Bifidobacterium	100%	2.00E-118	99.16
Bifidobacterium	Bifidobacterium	100%	2.00E-118	99.16
Bifidobacterium	Bifidobacterium	100%	2.00E-118	99.16
Bifidobacterium	Bifidobacterium	100%	2.00E-118	99.16
Bifidobacterium	Bifidobacterium	100%	2.00E-118	99.16
Bifidobacterium	Bifidobacterium	100%	2.00E-118	99.16
Bifidobacterium	Bifidobacterium	100%	2.00E-118	99.16
Bifidobacterium	Bifidobacterium	100%	2.00E-118	99.16
Bifidobacterium	Bifidobacterium	100%	2.00E-118	99.16
Bifidobacterium	Bifidobacterium	100%	2.00E-118	99.16
Bifidobacterium	Bifidobacterium	100%	2.00E-118	99.16
Bifidobacterium	Bifidobacterium	100%	2.00E-118	99.16
Bifidobacterium	Bifidobacterium	100%	2.00E-118	99.16
Bifidobacterium	Bifidobacterium	100%	2.00E-118	99.16
Bifidobacterium	Bifidobacterium	100%	2.00E-118	99.16
Bifidobacterium	Bifidobacterium	100%	2.00E-118	99.16
Bifidobacterium	Bifidobacterium	100%	2.00E-118	99.16
Bifidobacterium	Bifidobacterium	100%	2.00E-118	99.16
Bifidobacterium	Bifidobacterium	100%	2.00E-118	99.16
Bifidobacterium	Bifidobacterium	100%	2.00E-118	99.16
Bifidobacterium	Bifidobacterium	100%	2.00E-118	99.16
Bifidobacterium	Bifidobacterium	100%	2.00E-118	99.16
Bifidobacterium	Bifidobacterium	100%	2.00E-118	99.16
Bifidobacterium	Bifidobacterium	100%	2.00E-118	99.16
Bifidobacterium	Bifidobacterium	100%	2.00E-118	99.16
Bifidobacterium	Bifidobacterium	100%	2.00E-118	99.16
Bifidobacterium	Bifidobacterium	100%	2.00E-118	99.16
Bifidobacterium	Bifidobacterium	100%	2.00E-118	99.16
Bifidobacterium	Bifidobacterium	100%	2.00E-118	99.16
Bifidobacterium	Bifidobacterium	100%	2.00E-118	99.16
Bifidobacterium	Bifidobacterium	100%	2.00E-118	99.16
Uncultured Bific	uncultured Bifid	100%	2.00E-118	99.16
Bifidobacterium	Bifidobacterium	100%	2.00E-118	99.16
Bifidobacterium	Bifidobacterium	100%	2.00E-118	99.16
Bifidobacterium	Bifidobacterium	100%	2.00E-118	99.16

Bifidobacterium	Bifidobacterium	100%	2.00E-118	99.16
Bifidobacterium	Bifidobacterium	100%	2.00E-118	99.16
Bifidobacterium	Bifidobacterium	100%	2.00E-118	99.16
Bifidobacterium	Bifidobacterium	100%	2.00E-118	99.16
Bifidobacterium	Bifidobacterium	100%	2.00E-118	99.16
Bifidobacterium	Bifidobacterium	100%	2.00E-118	99.16
Bifidobacterium	Bifidobacterium	100%	2.00E-118	99.16
Bifidobacterium	Bifidobacterium	100%	2.00E-118	99.16
Bifidobacterium	Bifidobacterium	100%	2.00E-118	99.16
Bifidobacterium	Bifidobacterium	100%	2.00E-118	99.16
Bifidobacterium	Bifidobacterium	100%	2.00E-118	99.16
Bifidobacterium	Bifidobacterium	100%	2.00E-118	99.16
Bifidobacterium	Bifidobacterium	100%	2.00E-118	99.16
Bifidobacterium	Bifidobacterium	100%	2.00E-118	99.16
Bifidobacterium	Bifidobacterium	100%	2.00E-118	99.16
Bifidobacterium	Bifidobacterium	100%	2.00E-118	99.16
Bifidobacterium	Bifidobacterium	100%	2.00E-118	99.16
Bifidobacterium	Bifidobacterium	100%	2.00E-118	99.16
Bifidobacterium	Bifidobacterium	100%	2.00E-118	99.16
Bifidobacterium	Bifidobacterium	100%	2.00E-118	99.16
Bifidobacterium	Bifidobacterium	100%	2.00E-118	99.16
Bifidobacterium	Bifidobacterium	100%	2.00E-118	99.16
Bifidobacterium	Bifidobacterium	98%	2.00E-118	99.57
Bifidobacterium	Bifidobacterium	100%	2.00E-118	99.16
Bifidobacterium	Bifidobacterium	100%	2.00E-118	99.16
Uncultured Bifid	uncultured Bifid	100%	2.00E-118	99.16
Uncultured Bifid	uncultured Bifid	100%	2.00E-118	99.16
Bifidobacterium	Bifidobacterium	100%	2.00E-118	99.16
Bifidobacterium	Bifidobacterium	100%	2.00E-118	99.16
Bifidobacterium	Bifidobacterium	100%	2.00E-118	99.16
Bifidobacterium	Bifidobacterium	100%	2.00E-118	99.16
Bifidobacterium	Bifidobacterium	100%	2.00E-118	99.16
Bifidobacterium	Bifidobacterium	100%	2.00E-118	99.16
Bifidobacterium	Bifidobacterium	100%	2.00E-118	99.16
Bifidobacterium	Bifidobacterium	100%	2.00E-118	99.16
Bifidobacterium	Bifidobacterium	100%	2.00E-118	99.16
Bifidobacterium	Bifidobacterium	100%	2.00E-118	99.16
Bifidobacterium	Bifidobacterium	100%	2.00E-118	99.16
Uncultured Bifid	uncultured Bifid	100%	2.00E-118	99.16
Uncultured Bifid	uncultured Bifid	100%	2.00E-118	99.16
Uncultured Bifid	uncultured Bifid	100%	2.00E-118	99.16
Bifidobacterium	Bifidobacterium	100%	2.00E-118	99.16
Bifidobacterium	Bifidobacterium	100%	2.00E-118	99.16
Bifidobacterium	Bifidobacterium	100%	2.00E-118	99.16

Bifidobacterium	Bifidobacterium	100%	2.00E-118	99.16
Bifidobacterium	Bifidobacterium	100%	2.00E-118	99.16
Bifidobacterium	Bifidobacterium	100%	2.00E-118	99.16
Bifidobacterium	Bifidobacterium	100%	2.00E-118	99.16
Bifidobacterium	Bifidobacterium	100%	2.00E-118	99.16
Bifidobacterium	Bifidobacterium	100%	2.00E-118	99.16
Bifidobacterium	Bifidobacterium	100%	2.00E-118	99.16
Bifidobacterium	Bifidobacterium	100%	2.00E-118	99.16
Bifidobacterium	Bifidobacterium	100%	2.00E-118	99.16
Bifidobacterium	Bifidobacterium	100%	2.00E-118	99.16
Bifidobacterium	Bifidobacterium	100%	2.00E-118	99.16
Bifidobacterium	Bifidobacterium	100%	2.00E-118	99.16
Bifidobacterium	Bifidobacterium	100%	2.00E-118	99.16
Bifidobacterium	Bifidobacterium	100%	2.00E-118	99.16
Bifidobacterium	Bifidobacterium	100%	2.00E-118	99.16
Bifidobacterium	Bifidobacterium	100%	2.00E-118	99.16
Bifidobacterium	Bifidobacterium	100%	2.00E-118	99.16
Bifidobacterium	Bifidobacterium	100%	2.00E-118	99.16
Bifidobacterium	Bifidobacterium	100%	2.00E-118	99.16
Bifidobacterium	Bifidobacterium	100%	2.00E-118	99.16
Uncultured Bifid	uncultured Bifid	100%	2.00E-118	99.16
Uncultured Bifid	uncultured Bifid	100%	2.00E-118	99.16
Bifidobacterium	Bifidobacterium	100%	2.00E-118	99.16
Bifidobacterium	Bifidobacterium	100%	2.00E-118	99.16
Bifidobacterium	Bifidobacterium	100%	2.00E-118	99.16
Bifidobacterium	Bifidobacterium	100%	2.00E-118	99.16
Bifidobacterium	Bifidobacterium	100%	2.00E-118	99.16
Bifidobacterium	Bifidobacterium	100%	2.00E-118	99.16
Bifidobacterium	Bifidobacterium	100%	2.00E-118	99.16
Bifidobacterium	Bifidobacterium	100%	2.00E-118	99.16
Bifidobacterium	Bifidobacterium	100%	2.00E-118	99.16
Bifidobacterium	Bifidobacterium	100%	2.00E-118	99.16
Bifidobacterium	Bifidobacterium	100%	2.00E-118	99.16
Bifidobacterium	Bifidobacterium	100%	2.00E-118	99.16
Bifidobacterium	Bifidobacterium	100%	2.00E-118	99.16
Bifidobacterium	Bifidobacterium	100%	2.00E-118	99.16
Uncultured Bifid	uncultured Bifid	100%	2.00E-118	99.16
Bifidobacterium	Bifidobacterium	100%	2.00E-118	99.16
Bifidobacterium	Bifidobacterium	100%	2.00E-118	99.16
Bifidobacterium	Bifidobacterium	100%	2.00E-118	99.16
Bifidobacterium	Bifidobacterium	100%	2.00E-118	99.16
Bifidobacterium	Bifidobacterium	100%	2.00E-118	99.16
Bifidobacterium	Bifidobacterium	100%	2.00E-118	99.16
Bifidobacterium	Bifidobacterium	100%	2.00E-118	99.16

Bifidobacterium	Bifidobacterium	100%	2.00E-118	99.16
Bifidobacterium	Bifidobacterium	100%	2.00E-118	99.16
Bifidobacterium	Bifidobacterium	100%	2.00E-118	99.16
Bifidobacterium	Bifidobacterium	100%	2.00E-118	99.16
Bifidobacterium	Bifidobacterium	100%	2.00E-118	99.16
Bifidobacterium	Bifidobacterium	100%	2.00E-118	99.16
Bifidobacterium	Bifidobacterium	100%	2.00E-118	99.16
Bifidobacterium	Bifidobacterium	100%	2.00E-118	99.16
Bifidobacterium	Bifidobacterium	100%	2.00E-118	99.16
Bifidobacterium	Bifidobacterium	100%	2.00E-118	99.16
Bifidobacterium	Bifidobacterium	100%	2.00E-118	99.16
Bifidobacterium	Bifidobacterium	100%	2.00E-118	99.16
Uncultured Bifid	uncultured Bifid	100%	2.00E-118	99.16
Bifidobacterium	Bifidobacterium	100%	2.00E-118	99.16
Bifidobacterium	Bifidobacterium	100%	2.00E-118	99.16
Bifidobacterium	Bifidobacterium	100%	2.00E-118	99.16
Bifidobacterium	Bifidobacterium	100%	2.00E-118	99.16
Bifidobacterium	Bifidobacterium	100%	2.00E-118	99.16
Uncultured Bifid	uncultured Bifid	100%	2.00E-118	99.16
Uncultured Bifid	uncultured Bifid	100%	2.00E-118	99.16
Bifidobacterium	Bifidobacterium	100%	2.00E-118	99.16
Uncultured Bifid	uncultured Bifid	100%	2.00E-118	99.16
Uncultured Bifid	uncultured Bifid	100%	2.00E-118	99.16
Bifidobacterium	Bifidobacterium	100%	2.00E-118	99.16
Bifidobacterium	Bifidobacterium	100%	2.00E-118	99.16
Bifidobacterium	Bifidobacterium	100%	2.00E-118	99.16
Bifidobacterium	Bifidobacterium	100%	2.00E-118	99.16
Bifidobacterium	Bifidobacterium	100%	2.00E-118	99.16
Bifidobacterium	Bifidobacterium	100%	2.00E-118	99.16
Bifidobacterium	Bifidobacterium	100%	2.00E-118	99.16
Bifidobacterium	Bifidobacterium	100%	2.00E-118	99.16
Bifidobacterium	Bifidobacterium	100%	2.00E-118	99.16
Bifidobacterium	Bifidobacterium	100%	2.00E-118	99.16
Bifidobacterium	Bifidobacterium	100%	2.00E-118	99.16
Bifidobacterium	Bifidobacterium	100%	2.00E-118	99.16
Bifidobacterium	Bifidobacterium	100%	2.00E-118	99.16
Bifidobacterium	Bifidobacterium	100%	2.00E-118	99.16
Bifidobacterium	Bifidobacterium	100%	2.00E-118	99.16
Bifidobacterium	Bifidobacterium	100%	2.00E-118	99.16
Bifidobacterium	Bifidobacterium	100%	2.00E-118	99.16
Bifidobacterium	Bifidobacterium	100%	2.00E-118	99.16
Bifidobacterium	Bifidobacterium	100%	2.00E-118	99.16
Bifidobacterium	Bifidobacterium	100%	2.00E-118	99.16
Bifidobacterium	Bifidobacterium	100%	2.00E-118	99.16

Bifidobacterium	Bifidobacterium	100%	2.00E-118	99.16
Bifidobacterium	Bifidobacterium	100%	2.00E-118	99.16
Bifidobacterium	Bifidobacterium	100%	2.00E-118	99.16
Bifidobacterium	Bifidobacterium	100%	2.00E-118	99.16
Bifidobacterium	Bifidobacterium	100%	2.00E-118	99.16
Bifidobacterium	Bifidobacterium	100%	2.00E-118	99.16
Bifidobacterium	Bifidobacterium	100%	2.00E-118	99.16
Bifidobacterium	Bifidobacterium	100%	2.00E-118	99.16
Bifidobacterium	Bifidobacterium	100%	2.00E-118	99.16
Bifidobacterium	Bifidobacterium	100%	2.00E-118	99.16
Bifidobacterium	Bifidobacterium	100%	2.00E-118	99.16
Bifidobacterium	Bifidobacterium	100%	2.00E-118	99.16
Bifidobacterium	Bifidobacterium	100%	2.00E-118	99.16
Bifidobacterium	Bifidobacterium	100%	2.00E-118	99.16
Bifidobacterium	Bifidobacterium	100%	2.00E-118	99.16
Bifidobacterium	Bifidobacterium	100%	2.00E-118	99.16
Bifidobacterium	Bifidobacterium	100%	2.00E-118	99.16
Bifidobacterium	Bifidobacterium	100%	2.00E-118	99.16
Bifidobacterium	Bifidobacterium	100%	2.00E-118	99.16
Bifidobacterium	Bifidobacterium	100%	2.00E-118	99.16
Bifidobacterium	Bifidobacterium	100%	2.00E-118	99.16
Bifidobacterium	Bifidobacterium	100%	2.00E-118	99.16
Bifidobacterium	Bifidobacterium	100%	2.00E-118	99.16
Bifidobacterium	Bifidobacterium	100%	2.00E-118	99.16
Bifidobacterium	Bifidobacterium	100%	2.00E-118	99.16
Bifidobacterium	Bifidobacterium	100%	2.00E-118	99.16
Bifidobacterium	Bifidobacterium	100%	2.00E-118	99.16
Bifidobacterium	Bifidobacterium	100%	2.00E-118	99.16
Bifidobacterium	Bifidobacterium	100%	2.00E-118	99.16
Bifidobacterium	Bifidobacterium	100%	2.00E-118	99.16
Bifidobacterium	Bifidobacterium	100%	2.00E-118	99.16
Bifidobacterium	Bifidobacterium	100%	2.00E-118	99.16
Bifidobacterium	Bifidobacterium	100%	2.00E-118	99.16
Bifidobacterium	Bifidobacterium	100%	2.00E-118	99.16
Bifidobacterium	Bifidobacterium	100%	2.00E-118	99.16
Bifidobacterium	Bifidobacterium	100%	2.00E-118	99.16
Bifidobacterium	Bifidobacterium	100%	2.00E-118	99.16
Bifidobacterium	Bifidobacterium	100%	2.00E-118	99.16
Bifidobacterium	Bifidobacterium	100%	2.00E-118	99.16
Bifidobacterium	Bifidobacterium	100%	2.00E-118	99.16
Bifidobacterium	Bifidobacterium	100%	2.00E-118	99.16
Bifidobacterium	Bifidobacterium	100%	2.00E-118	99.16
Bifidobacterium	Bifidobacterium	99%	2.00E-118	99.57
Bifidobacterium	Bifidobacterium	100%	2.00E-118	99.16

Bifidobacterium	Bifidobacterium	100%	2.00E-118	99.16
Bifidobacterium	Bifidobacterium	100%	2.00E-118	99.16
Bifidobacterium	Bifidobacterium	100%	2.00E-118	99.16
Bifidobacterium	Bifidobacterium	100%	2.00E-118	99.16
Bifidobacterium	Bifidobacterium	100%	2.00E-118	99.16
Uncultured Bifid	uncultured Bifid	100%	2.00E-118	99.16
Bifidobacterium	Bifidobacterium	100%	2.00E-118	99.16
Bifidobacterium	Bifidobacterium	100%	2.00E-118	99.16
Bifidobacterium	Bifidobacterium	100%	2.00E-118	99.16
Bifidobacterium	Bifidobacterium	100%	2.00E-118	99.16
Bifidobacterium	Bifidobacterium	100%	2.00E-118	99.16
Bifidobacterium	Bifidobacterium	100%	2.00E-118	99.16
Bifidobacterium	Bifidobacterium	100%	2.00E-118	99.16
Bifidobacterium	Bifidobacterium	100%	2.00E-118	99.16
Bifidobacterium	Bifidobacterium	100%	2.00E-118	99.16
Bifidobacterium	Bifidobacterium	100%	2.00E-118	99.16
Bifidobacterium	Bifidobacterium	100%	2.00E-118	99.16
Bifidobacterium	Bifidobacterium	100%	2.00E-118	99.16
Bifidobacterium	Bifidobacterium	100%	2.00E-118	99.16
Bifidobacterium	Bifidobacterium	100%	2.00E-118	99.16
Bifidobacterium	Bifidobacterium	100%	2.00E-118	99.16
Bifidobacterium	Bifidobacterium	100%	2.00E-118	99.16
Bifidobacterium	Bifidobacterium	100%	2.00E-118	99.16
Bifidobacterium	Bifidobacterium	100%	2.00E-118	99.16
Bifidobacterium	Bifidobacterium	100%	2.00E-118	99.16
Uncultured Bifid	uncultured Bifid	100%	2.00E-118	99.16
Bifidobacterium	Bifidobacterium	100%	2.00E-118	99.16
Bifidobacterium	Bifidobacterium	100%	2.00E-118	99.16
Bifidobacterium	Bifidobacterium	98%	2.00E-118	99.57
Bifidobacterium	Bifidobacterium	100%	2.00E-118	99.16
Bifidobacterium	Bifidobacterium	100%	2.00E-118	99.16
Bifidobacterium	Bifidobacterium	100%	2.00E-118	99.16
Bifidobacterium	Bifidobacterium	100%	2.00E-118	99.16
Bifidobacterium	Bifidobacterium	100%	2.00E-118	99.16
Bifidobacterium	Bifidobacterium	100%	2.00E-118	99.16
Bifidobacterium	Bifidobacterium	100%	2.00E-118	99.16
Bifidobacterium	Bifidobacterium	100%	2.00E-118	99.16
Bifidobacterium	Bifidobacterium	100%	2.00E-118	99.16
Bifidobacterium	Bifidobacterium	100%	2.00E-118	99.16
Bifidobacterium	Bifidobacterium	100%	2.00E-118	99.16
Bifidobacterium	Bifidobacterium	100%	2.00E-118	99.16
Bifidobacterium	Bifidobacterium	100%	2.00E-118	99.16
Bifidobacterium	Bifidobacterium	100%	2.00E-118	99.16
Bifidobacterium	Bifidobacterium	100%	2.00E-118	99.16

Bifidobacterium	Bifidobacterium	100%	2.00E-118	99.16
Bifidobacterium	Bifidobacterium	100%	2.00E-118	99.16
Bifidobacterium	Bifidobacterium	98%	2.00E-118	99.57
Bifidobacterium	Bifidobacterium	100%	2.00E-118	99.16
Bifidobacterium	Bifidobacterium	100%	2.00E-118	99.16
Bifidobacterium	Bifidobacterium	100%	2.00E-118	99.16
Bifidobacterium	Bifidobacterium	100%	2.00E-118	99.16
Bifidobacterium	Bifidobacterium	100%	2.00E-118	99.16
Bifidobacterium	Bifidobacterium	100%	2.00E-118	99.16
Bifidobacterium	Bifidobacterium	100%	2.00E-118	99.16
Bifidobacterium	Bifidobacterium	100%	2.00E-118	99.16
Bifidobacterium	Bifidobacterium	100%	2.00E-118	99.16
Bifidobacterium	Bifidobacterium	100%	2.00E-118	99.16
Bifidobacterium	Bifidobacterium	100%	2.00E-118	99.16
Bifidobacterium	Bifidobacterium	100%	2.00E-118	99.16
Uncultured Bifid	uncultured Bifid	100%	2.00E-118	99.16
Uncultured Bifid	uncultured Bifid	100%	2.00E-118	99.16
Uncultured Bifid	uncultured Bifid	100%	2.00E-118	99.16
Uncultured Bifid	uncultured Bifid	100%	2.00E-118	99.16
Uncultured Bifid	uncultured Bifid	100%	2.00E-118	99.16
Uncultured Bifid	uncultured Bifid	100%	2.00E-118	99.16
Uncultured Bifid	uncultured Bifid	100%	2.00E-118	99.16
Bifidobacterium	Bifidobacterium	100%	2.00E-118	99.16
Bifidobacterium	Bifidobacterium	100%	2.00E-118	99.16
Bifidobacterium	Bifidobacterium	100%	2.00E-118	99.16
Bifidobacterium	Bifidobacterium	100%	2.00E-118	99.16
Bifidobacterium	Bifidobacterium	100%	2.00E-118	99.16
Bifidobacterium	Bifidobacterium	100%	2.00E-118	99.16
Bifidobacterium	Bifidobacterium	100%	2.00E-118	99.16
Bifidobacterium	Bifidobacterium	100%	2.00E-118	98.73
Bifidobacterium	Bifidobacterium	100%	7.00E-118	98.74
Bifidobacterium	Bifidobacterium	99%	7.00E-118	99.16
Bifidobacterium	Bifidobacterium	100%	7.00E-118	99.16
Bifidobacterium	Bifidobacterium	100%	7.00E-118	98.73
Bifidobacterium	Bifidobacterium	100%	7.00E-118	98.73
Bifidobacterium	Bifidobacterium	100%	7.00E-118	99.16
Bifidobacterium	Bifidobacterium	100%	7.00E-118	99.16
Bifidobacterium	Bifidobacterium	100%	7.00E-118	99.16
Uncultured Bifid	uncultured Bifid	100%	7.00E-118	98.73
Bifidobacterium	Bifidobacterium	100%	3.00E-117	98.73
Bifidobacterium	Bifidobacterium	99%	3.00E-117	99.15
Bifidobacterium	Bifidobacterium	99%	3.00E-117	99.15
Uncultured Bifid	uncultured Bifid	97%	3.00E-117	99.57
Uncultured Bifid	uncultured Bifid	97%	3.00E-117	99.57

Uncultured Bific	uncultured Bifid	97%	3.00E-117	99.57
Bifidobacterium	Bifidobacterium	99%	3.00E-117	99.15
Bifidobacterium	Bifidobacterium	100%	3.00E-117	98.75
Bifidobacterium	Bifidobacterium	100%	3.00E-117	98.31
Bifidobacterium	Bifidobacterium	100%	3.00E-117	98.31
Bifidobacterium	Bifidobacterium	96%	3.00E-117	100
Bifidobacterium	Bifidobacterium	99%	3.00E-117	99.15
Bifidobacterium	Bifidobacterium	100%	3.00E-117	98.31
Bifidobacterium	Bifidobacterium	100%	3.00E-117	98.73
Bifidobacterium	Bifidobacterium	100%	3.00E-117	98.74
Uncultured Bific	uncultured Bifid	100%	3.00E-117	98.74
Uncultured Bific	uncultured Bifid	100%	3.00E-117	98.74
Uncultured Bific	uncultured Bifid	100%	3.00E-117	98.73
Bifidobacterium	Bifidobacterium	100%	9.00E-117	98.73
Bifidobacterium	Bifidobacterium	100%	9.00E-117	98.73
Bifidobacterium	Bifidobacterium	100%	9.00E-117	98.73
Bifidobacterium	Bifidobacterium	100%	9.00E-117	98.73
Bifidobacterium	Bifidobacterium	100%	9.00E-117	98.73
Bifidobacterium	Bifidobacterium	100%	9.00E-117	98.73
Bifidobacterium	Bifidobacterium	100%	9.00E-117	98.73
Bifidobacterium	Bifidobacterium	100%	9.00E-117	98.73
Bifidobacterium	Bifidobacterium	100%	9.00E-117	98.73
Uncultured Bific	uncultured Bifid	100%	9.00E-117	98.73
Bifidobacterium	Bifidobacterium	100%	9.00E-117	98.73
Bifidobacterium	Bifidobacterium	100%	9.00E-117	98.73
Bifidobacterium	Bifidobacterium	100%	9.00E-117	98.73
Bifidobacterium	Bifidobacterium	100%	9.00E-117	98.73
Bifidobacterium	Bifidobacterium	100%	9.00E-117	98.73
Bifidobacterium	Bifidobacterium	100%	9.00E-117	98.73
Bifidobacterium	Bifidobacterium	100%	9.00E-117	98.73
Bifidobacterium	Bifidobacterium	100%	9.00E-117	98.73
Bifidobacterium	Bifidobacterium	100%	9.00E-117	98.73
Bifidobacterium	Bifidobacterium	100%	9.00E-117	98.73
Bifidobacterium	Bifidobacterium	100%	9.00E-117	98.73
Bifidobacterium	Bifidobacterium	100%	9.00E-117	98.73
Bifidobacterium	Bifidobacterium	100%	9.00E-117	98.73
Bifidobacterium	Bifidobacterium	100%	9.00E-117	98.73
Bifidobacterium	Bifidobacterium	100%	9.00E-117	98.73
Bifidobacterium	Bifidobacterium	100%	9.00E-117	98.73
Bifidobacterium	Bifidobacterium	100%	9.00E-117	98.73
Bifidobacterium	Bifidobacterium	100%	9.00E-117	98.73
Bifidobacterium	Bifidobacterium	100%	9.00E-117	98.73
Bifidobacterium	Bifidobacterium	100%	9.00E-117	98.73
Bifidobacterium	Bifidobacterium	100%	9.00E-117	98.73

Bifidobacterium	Bifidobacterium	100%	9.00E-117	98.73
Bifidobacterium	Bifidobacterium	100%	9.00E-117	98.32
Bifidobacterium	Bifidobacterium	100%	9.00E-117	98.73
Bifidobacterium	Bifidobacterium	100%	9.00E-117	98.73
Uncultured Bifid	uncultured Bifid	100%	9.00E-117	98.73
Bifidobacterium	Bifidobacterium	100%	9.00E-117	98.73
Bifidobacterium	Bifidobacterium	100%	9.00E-117	98.31
Bifidobacterium	Bifidobacterium	100%	9.00E-117	98.73
Bifidobacterium	Bifidobacterium	100%	9.00E-117	98.74
Bifidobacterium	Bifidobacterium	100%	9.00E-117	98.73
Bifidobacterium	Bifidobacterium	100%	9.00E-117	98.73
Bifidobacterium	Bifidobacterium	100%	9.00E-117	98.73
Bifidobacterium	Bifidobacterium	100%	9.00E-117	98.73
Bifidobacterium	Bifidobacterium	100%	9.00E-117	98.73
Bifidobacterium	Bifidobacterium	100%	9.00E-117	98.73
Bifidobacterium	Bifidobacterium	100%	9.00E-117	98.73
Bifidobacterium	Bifidobacterium	100%	9.00E-117	98.73
Bifidobacterium	Bifidobacterium	100%	9.00E-117	98.73
Bifidobacterium	Bifidobacterium	100%	9.00E-117	98.73
Bifidobacterium	Bifidobacterium	100%	9.00E-117	98.73
Bifidobacterium	Bifidobacterium	100%	9.00E-117	98.73
Uncultured Bifid	uncultured Bifid	100%	9.00E-117	98.73
Bifidobacterium	Bifidobacterium	97%	9.00E-117	99.14
Bifidobacterium	Bifidobacterium	100%	9.00E-117	98.73
Bifidobacterium	Bifidobacterium	100%	9.00E-117	98.73
Bifidobacterium	Bifidobacterium	100%	9.00E-117	98.73
Bifidobacterium	Bifidobacterium	100%	9.00E-117	98.73
Bifidobacterium	Bifidobacterium	100%	9.00E-117	98.74
Bifidobacterium	Bifidobacterium	100%	9.00E-117	98.73
Bifidobacterium	Bifidobacterium	100%	9.00E-117	98.73
Bifidobacterium	Bifidobacterium	100%	9.00E-117	98.73
Bifidobacterium	Bifidobacterium	100%	9.00E-117	98.73
Bifidobacterium	Bifidobacterium	100%	9.00E-117	98.73
Bifidobacterium	Bifidobacterium	100%	9.00E-117	98.73
Bifidobacterium	Bifidobacterium	100%	9.00E-117	98.73
Bifidobacterium	Bifidobacterium	100%	9.00E-117	98.73
Bifidobacterium	Bifidobacterium	100%	9.00E-117	98.73
Bifidobacterium	Bifidobacterium	100%	9.00E-117	98.73
Bifidobacterium	Bifidobacterium	100%	9.00E-117	98.73
Uncultured Bifid	uncultured Bifid	100%	9.00E-117	98.73
Uncultured Bifid	uncultured Bifid	100%	9.00E-117	98.73
Uncultured Bifid	uncultured Bifid	100%	9.00E-117	98.73
Bifidobacterium	Bifidobacterium	100%	9.00E-117	98.73
Bifidobacterium	Bifidobacterium	100%	9.00E-117	98.73

Bifidobacterium	Bifidobacterium	100%	9.00E-117	98.73
Uncultured Bific	uncultured Bifid	100%	9.00E-117	98.74
Bifidobacterium	Bifidobacterium	100%	9.00E-117	98.73
Bifidobacterium	Bifidobacterium	100%	9.00E-117	98.73
Bifidobacterium	Bifidobacterium	100%	9.00E-117	98.73
Bifidobacterium	Bifidobacterium	100%	9.00E-117	98.73
Bifidobacterium	Bifidobacterium	100%	9.00E-117	98.73
Bifidobacterium	Bifidobacterium	100%	9.00E-117	98.73
Bifidobacterium	Bifidobacterium	100%	9.00E-117	98.73
Bifidobacterium	Bifidobacterium	100%	9.00E-117	98.73
Bifidobacterium	Bifidobacterium	97%	9.00E-117	99.14
Bifidobacterium	Bifidobacterium	100%	3.00E-116	98.73
Bifidobacterium	Bifidobacterium	99%	3.00E-116	98.72
Bifidobacterium	Bifidobacterium	98%	3.00E-116	99.14
Uncultured Bific	uncultured Bifid	100%	3.00E-116	98.33
Uncultured Bific	uncultured Bifid	97%	1.00E-115	99.14
Uncultured Bific	uncultured Bifid	97%	1.00E-115	99.14
Bifidobacterium	Bifidobacterium	100%	1.00E-115	98.73
Bifidobacterium	Bifidobacterium	100%	1.00E-115	97.91
Bifidobacterium	Bifidobacterium	97%	1.00E-115	99.14
Bifidobacterium	Bifidobacterium	100%	4.00E-115	98.32
Bifidobacterium	Bifidobacterium	100%	4.00E-115	98.31
Bifidobacterium	Bifidobacterium	100%	4.00E-115	98.31
Bifidobacterium	Bifidobacterium	100%	4.00E-115	98.31
Bifidobacterium	Bifidobacterium	100%	4.00E-115	98.31
Bifidobacterium	Bifidobacterium	100%	4.00E-115	98.31
Bifidobacterium	Bifidobacterium	100%	4.00E-115	98.31
Bifidobacterium	Bifidobacterium	100%	4.00E-115	98.31
Bifidobacterium	Bifidobacterium	100%	4.00E-115	98.31
Bifidobacterium	Bifidobacterium	100%	4.00E-115	98.31
Bifidobacterium	Bifidobacterium	100%	4.00E-115	98.31
Bifidobacterium	Bifidobacterium	100%	4.00E-115	98.31
Bifidobacterium	Bifidobacterium	100%	4.00E-115	98.31
Bifidobacterium	Bifidobacterium	100%	4.00E-115	98.31
Bifidobacterium	Bifidobacterium	100%	4.00E-115	98.31
Bifidobacterium	Bifidobacterium	100%	4.00E-115	98.31
Bifidobacterium	Bifidobacterium	100%	4.00E-115	98.31
Bifidobacterium	Bifidobacterium	100%	4.00E-115	98.31
Bifidobacterium	Bifidobacterium	100%	4.00E-115	98.31
Bifidobacterium	Bifidobacterium	100%	4.00E-115	98.31
Bifidobacterium	Bifidobacterium	100%	4.00E-115	98.31
Bifidobacterium	Bifidobacterium	100%	4.00E-115	98.31
Bifidobacterium	Bifidobacterium	100%	4.00E-115	98.31
Bifidobacterium	Bifidobacterium	100%	4.00E-115	98.31

Bifidobacterium	Bifidobacterium	99%	4.00E-115	98.31
Bifidobacterium	Bifidobacterium	100%	4.00E-115	98.31
Bifidobacterium	Bifidobacterium	100%	4.00E-115	98.31
Bifidobacterium	Bifidobacterium	100%	4.00E-115	98.31
Bifidobacterium	Bifidobacterium	100%	4.00E-115	98.31
Bifidobacterium	Bifidobacterium	100%	4.00E-115	98.31
Bifidobacterium	Bifidobacterium	100%	4.00E-115	98.31
Bifidobacterium	Bifidobacterium	100%	4.00E-115	98.31
Bifidobacterium	Bifidobacterium	100%	4.00E-115	98.31
Bifidobacterium	Bifidobacterium	100%	4.00E-115	98.31
Bifidobacterium	Bifidobacterium	100%	4.00E-115	98.31
Bifidobacterium	Bifidobacterium	100%	4.00E-115	98.31
Uncultured Bifid	uncultured Bifid	100%	4.00E-115	98.31
Uncultured Bifid	uncultured Bifid	100%	4.00E-115	98.31
Bifidobacterium	Bifidobacterium	100%	4.00E-115	98.31
Bifidobacterium	Bifidobacterium	100%	4.00E-115	98.32
Bifidobacterium	Bifidobacterium	100%	4.00E-115	98.31
Bifidobacterium	Bifidobacterium	100%	4.00E-115	98.31
Bifidobacterium	Bifidobacterium	100%	4.00E-115	98.31
Bifidobacterium	Bifidobacterium	100%	4.00E-115	98.31
Bifidobacterium	Bifidobacterium	100%	4.00E-115	98.31
Bifidobacterium	Bifidobacterium	100%	4.00E-115	98.31
Bifidobacterium	Bifidobacterium	100%	4.00E-115	98.31
Bifidobacterium	Bifidobacterium	100%	4.00E-115	98.31
Bifidobacterium	Bifidobacterium	100%	4.00E-115	98.31
Uncultured Bifid	uncultured Bifid	100%	4.00E-115	98.31
Bifidobacterium	Bifidobacterium	100%	4.00E-115	98.31
Bifidobacterium	Bifidobacterium	100%	4.00E-115	98.31
Bifidobacterium	Bifidobacterium	100%	4.00E-115	98.31
Bifidobacterium	Bifidobacterium	100%	4.00E-115	98.31
Bifidobacterium	Bifidobacterium	99%	4.00E-115	98.72
Bifidobacterium	Bifidobacterium	100%	4.00E-115	98.31
Bifidobacterium	Bifidobacterium	100%	4.00E-115	98.31
Bifidobacterium	Bifidobacterium	100%	4.00E-115	98.31
Bifidobacterium	Bifidobacterium	100%	4.00E-115	98.31
Bifidobacterium	Bifidobacterium	100%	4.00E-115	98.31
Bifidobacterium	Bifidobacterium	100%	4.00E-115	98.31
Bifidobacterium	Bifidobacterium	100%	4.00E-115	98.31
Uncultured Bifid	uncultured Bifid	100%	4.00E-115	98.31
Uncultured Bifid	uncultured Bifid	100%	4.00E-115	98.31
Uncultured Bifid	uncultured Bifid	100%	4.00E-115	98.31
Bifidobacterium	Bifidobacterium	98%	4.00E-115	98.72
Bifidobacterium	Bifidobacterium	100%	4.00E-115	98.31
Bifidobacterium	Bifidobacterium	100%	4.00E-115	98.31

Bifidobacterium	Bifidobacterium	95%	2.00E-114	99.56
Bifidobacterium	Bifidobacterium	100%	2.00E-114	97.89
Bifidobacterium	Bifidobacterium	100%	2.00E-114	97.47
Bifidobacterium	Bifidobacterium	95%	2.00E-114	99.56
Bifidobacterium	Bifidobacterium	100%	2.00E-114	98.31
Bifidobacterium	Bifidobacterium	98%	2.00E-114	98.72
Bifidobacterium	Bifidobacterium	98%	2.00E-114	98.72
Bifidobacterium	Bifidobacterium	97%	2.00E-114	98.28
Bifidobacterium	Bifidobacterium	100%	2.00E-114	97.47
Uncultured Bific	uncultured Bifid	97%	6.00E-114	98.71
Bifidobacterium	Bifidobacterium	100%	6.00E-114	97.89
Bifidobacterium	Bifidobacterium	100%	6.00E-114	97.05
Uncultured Bific	uncultured Bifid	100%	6.00E-114	97.9
B.breve (ATCC	Bifidobacterium	97%	6.00E-114	98.28
Bifidobacterium	Bifidobacterium	100%	6.00E-114	97.05
Bifidobacterium	Bifidobacterium	100%	2.00E-113	97.89
Bifidobacterium	Bifidobacterium	100%	2.00E-113	97.89
Bifidobacterium	Bifidobacterium	100%	2.00E-113	97.89
Bifidobacterium	Bifidobacterium	100%	2.00E-113	97.89
Bifidobacterium	Bifidobacterium	100%	2.00E-113	97.89
Bifidobacterium	Bifidobacterium	100%	2.00E-113	97.89
Bifidobacterium	Bifidobacterium	100%	2.00E-113	97.89
Bifidobacterium	Bifidobacterium	100%	2.00E-113	97.89
Bifidobacterium	Bifidobacterium	100%	2.00E-113	97.89
Bifidobacterium	Bifidobacterium	100%	2.00E-113	97.89
Bifidobacterium	Bifidobacterium	100%	2.00E-113	97.89
Bifidobacterium	Bifidobacterium	100%	2.00E-113	97.89
Bifidobacterium	Bifidobacterium	100%	2.00E-113	97.89
Bifidobacterium	Bifidobacterium	100%	2.00E-113	97.89
Bifidobacterium	Bifidobacterium	100%	2.00E-113	97.89
Bifidobacterium	Bifidobacterium	100%	2.00E-113	97.89
Bifidobacterium	Bifidobacterium	100%	2.00E-113	97.89
Bifidobacterium	Bifidobacterium	100%	2.00E-113	97.89
Bifidobacterium	Bifidobacterium	100%	2.00E-113	97.89
Bifidobacterium	Bifidobacterium	100%	2.00E-113	97.89
Bifidobacterium	Bifidobacterium	100%	2.00E-113	97.89
Bifidobacterium	Bifidobacterium	100%	2.00E-113	97.89
Bifidobacterium	Bifidobacterium	100%	2.00E-113	97.89
Bifidobacterium	Bifidobacterium	100%	2.00E-113	97.89
Bifidobacterium	Bifidobacterium	100%	2.00E-113	97.89
Bifidobacterium	Bifidobacterium	100%	2.00E-113	97.89
Bifidobacterium	Bifidobacterium	100%	2.00E-113	97.89
Bifidobacterium	Bifidobacterium	100%	2.00E-113	97.89

Bifidobacterium	Bifidobacterium	100%	2.00E-113	97.89
Bifidobacterium	Bifidobacterium	100%	2.00E-113	97.89
Bifidobacterium	Bifidobacterium	100%	2.00E-113	97.89
Bifidobacterium	Bifidobacterium	100%	2.00E-113	97.89
Bifidobacterium	Bifidobacterium	100%	2.00E-113	97.89
Bifidobacterium	Bifidobacterium	100%	2.00E-113	97.89
Bifidobacterium	Bifidobacterium	100%	2.00E-113	97.89
Bifidobacterium	Bifidobacterium	100%	2.00E-113	97.89
Bifidobacterium	Bifidobacterium	100%	2.00E-113	97.89
Bifidobacterium	Bifidobacterium	100%	2.00E-113	97.89
Bifidobacterium	Bifidobacterium	100%	2.00E-113	97.89
Bifidobacterium	Bifidobacterium	100%	2.00E-113	97.89
Bifidobacterium	Bifidobacterium	100%	2.00E-113	97.89
Uncultured Bific	uncultured Bifid	100%	2.00E-113	97.89
Bifidobacterium	Bifidobacterium	100%	2.00E-113	97.89
Bifidobacterium	Bifidobacterium	100%	2.00E-113	97.89
Bifidobacterium	Bifidobacterium	100%	2.00E-113	97.89
Bifidobacterium	Bifidobacterium	100%	2.00E-113	97.89
Bifidobacterium	Bifidobacterium	100%	2.00E-113	97.89
Bifidobacterium	Bifidobacterium	100%	2.00E-113	97.89
Bifidobacterium	Bifidobacterium	100%	2.00E-113	97.89
Bifidobacterium	Bifidobacterium	100%	2.00E-113	97.89
Bifidobacterium	Bifidobacterium	100%	2.00E-113	97.89
Bifidobacterium	Bifidobacterium	100%	2.00E-113	97.89
Bifidobacterium	Bifidobacterium	100%	2.00E-113	97.89
Bifidobacterium	Bifidobacterium	100%	2.00E-113	97.89
Bifidobacterium	Bifidobacterium	100%	2.00E-113	97.89
Bifidobacterium	Bifidobacterium	100%	2.00E-113	97.89
Bifidobacterium	Bifidobacterium	100%	2.00E-113	97.89
Bifidobacterium	Bifidobacterium	100%	2.00E-113	97.89
Bifidobacterium	Bifidobacterium	100%	2.00E-113	97.89
Bifidobacterium	Bifidobacterium	100%	2.00E-113	97.89
Bifidobacterium	Bifidobacterium	100%	2.00E-113	97.89
Bifidobacterium	Bifidobacterium	100%	2.00E-113	97.89
Bifidobacterium	Bifidobacterium	100%	2.00E-113	97.89
Bifidobacterium	Bifidobacterium	100%	2.00E-113	97.89
Bifidobacterium	Bifidobacterium	100%	2.00E-113	97.89
Bifidobacterium	Bifidobacterium	100%	2.00E-113	97.89
Bifidobacterium	Bifidobacterium	100%	2.00E-113	97.89
Bifidobacterium	Bifidobacterium	100%	2.00E-113	97.89
Bifidobacterium	Bifidobacterium	100%	2.00E-113	97.89
Bifidobacterium	Bifidobacterium	100%	2.00E-113	97.89
Bifidobacterium	Bifidobacterium	100%	2.00E-113	97.89
Bifidobacterium	Bifidobacterium	100%	2.00E-113	97.89

Bifidobacterium	Bifidobacterium	100%	2.00E-113	97.89
Bifidobacterium	Bifidobacterium	100%	2.00E-113	97.89
Bifidobacterium	Bifidobacterium	100%	2.00E-113	97.89
Bifidobacterium	Bifidobacterium	100%	2.00E-113	97.89
Bifidobacterium	Bifidobacterium	100%	2.00E-113	97.89
Bifidobacterium	Bifidobacterium	100%	2.00E-113	97.89
Bifidobacterium	Bifidobacterium	100%	2.00E-113	97.89
Bifidobacterium	Bifidobacterium	100%	2.00E-113	97.89
Bifidobacterium	Bifidobacterium	100%	2.00E-113	97.89
Uncultured Bifi	uncultured Bifid	100%	2.00E-113	97.89
Uncultured Bifi	uncultured Bifid	100%	2.00E-113	97.89
Uncultured Bifi	uncultured Bifid	100%	2.00E-113	97.89
Bifidobacterium	Bifidobacterium	100%	2.00E-113	97.89
Bifidobacterium	Bifidobacterium	100%	2.00E-113	97.89
Bifidobacterium	Bifidobacterium	100%	2.00E-113	97.89
Bifidobacterium	Bifidobacterium	100%	2.00E-113	97.89
Bifidobacterium	Bifidobacterium	100%	2.00E-113	97.89
Bifidobacterium	Bifidobacterium	100%	2.00E-113	97.89
Bifidobacterium	Bifidobacterium	100%	2.00E-113	97.89
Bifidobacterium	Bifidobacterium	100%	2.00E-113	97.89
Bifidobacterium	Bifidobacterium	100%	2.00E-113	97.89
Bifidobacterium	Bifidobacterium	100%	2.00E-113	97.89
Bifidobacterium	Bifidobacterium	100%	2.00E-113	97.89
Bifidobacterium	Bifidobacterium	100%	2.00E-113	97.89
Bifidobacterium	Bifidobacterium	100%	2.00E-113	97.89
Bifidobacterium	Bifidobacterium	100%	2.00E-113	97.89
Bifidobacterium	Bifidobacterium	100%	2.00E-113	97.89
Bifidobacterium	Bifidobacterium	100%	2.00E-113	97.89
Bifidobacterium	Bifidobacterium	100%	2.00E-113	97.89
Bifidobacterium	Bifidobacterium	100%	2.00E-113	97.89
Uncultured Bifi	uncultured Bifid	100%	2.00E-113	97.89
Uncultured Bifi	uncultured Bifid	100%	2.00E-113	97.89
Bifidobacterium	Bifidobacterium	100%	2.00E-113	97.89
Bifidobacterium	Bifidobacterium	100%	2.00E-113	97.89
Bifidobacterium	Bifidobacterium	100%	2.00E-113	97.89
Uncultured Bifi	uncultured Bifid	98%	2.00E-113	98.29
Bifidobacterium	Bifidobacterium	100%	2.00E-113	97.89
Bifidobacterium	Bifidobacterium	100%	2.00E-113	97.89
Bifidobacterium	Bifidobacterium	100%	2.00E-113	97.89
Bifidobacterium	Bifidobacterium	100%	2.00E-113	97.89
Bifidobacterium	Bifidobacterium	100%	2.00E-113	97.89
Uncultured Bifi	uncultured Bifid	100%	2.00E-113	97.89
Uncultured Bifi	uncultured Bifid	100%	2.00E-113	97.89
Uncultured Bifi	uncultured Bifid	100%	2.00E-113	97.89

Bifidobacterium	Bifidobacterium	100%	2.00E-113	97.89
Bifidobacterium	Bifidobacterium	100%	2.00E-113	97.89
Bifidobacterium	Bifidobacterium	100%	2.00E-113	97.89
Bifidobacterium	Bifidobacterium	100%	2.00E-113	97.89
Bifidobacterium	Bifidobacterium	100%	2.00E-113	97.89
Uncultured Bific	uncultured Bifid	100%	2.00E-113	97.89
Bifidobacterium	Bifidobacterium	100%	2.00E-113	97.89
Bifidobacterium	Bifidobacterium	100%	2.00E-113	97.89
Bifidobacterium	Bifidobacterium	100%	2.00E-113	97.89
Bifidobacterium	Bifidobacterium	100%	2.00E-113	97.89
Bifidobacterium	Bifidobacterium	100%	2.00E-113	97.89
Bifidobacterium	Bifidobacterium	100%	2.00E-113	97.89
Bifidobacterium	Bifidobacterium	100%	2.00E-113	97.89
Bifidobacterium	Bifidobacterium	100%	2.00E-113	97.89
Bifidobacterium	Bifidobacterium	100%	2.00E-113	97.89
Bifidobacterium	Bifidobacterium	100%	2.00E-113	97.89
Bifidobacterium	Bifidobacterium	100%	2.00E-113	97.89
Bifidobacterium	Bifidobacterium	100%	2.00E-113	97.89
Bifidobacterium	Bifidobacterium	100%	2.00E-113	97.89
Uncultured Bific	uncultured Bifid	100%	2.00E-113	97.89
Bifidobacterium	Bifidobacterium	100%	2.00E-113	97.89
Bifidobacterium	Bifidobacterium	100%	2.00E-113	97.89
Bifidobacterium	Bifidobacterium	100%	2.00E-113	97.89
uncultured Bifid	uncultured Bifid	100%	2.00E-113	97.89
Bifidobacterium	Bifidobacterium	100%	2.00E-113	97.89
Bifidobacterium	Bifidobacterium	100%	2.00E-113	97.89
Bifidobacterium	Bifidobacterium	100%	2.00E-113	97.89
Bifidobacterium	Bifidobacterium	100%	2.00E-113	97.89
Bifidobacterium	Bifidobacterium	100%	2.00E-113	97.89
Bifidobacterium	Bifidobacterium	100%	2.00E-113	97.89
Bifidobacterium	Bifidobacterium	100%	2.00E-113	97.89
Bifidobacterium	Bifidobacterium	100%	2.00E-113	97.89
Bifidobacterium	Bifidobacterium	100%	2.00E-113	97.89
Bifidobacterium	Bifidobacterium	100%	2.00E-113	97.89
Bifidobacterium	Bifidobacterium	100%	2.00E-113	97.89
Bifidobacterium	Bifidobacterium	100%	2.00E-113	97.89
Bifidobacterium	Bifidobacterium	100%	2.00E-113	97.89
Bifidobacterium	Bifidobacterium	100%	2.00E-113	97.89
Bifidobacterium	Bifidobacterium	100%	2.00E-113	97.89
Bifidobacterium	Bifidobacterium	100%	2.00E-113	97.89
Bifidobacterium	Bifidobacterium	100%	2.00E-113	97.89
Bifidobacterium	Bifidobacterium	100%	2.00E-113	97.89
Bifidobacterium	Bifidobacterium	100%	2.00E-113	97.89
Bifidobacterium	Bifidobacterium	100%	2.00E-113	97.89

Bifidobacterium	Bifidobacterium	100%	2.00E-113	97.89
Bifidobacterium	Bifidobacterium	100%	2.00E-113	97.89
Bifidobacterium	Bifidobacterium	100%	2.00E-113	97.89
Bifidobacterium	Bifidobacterium	100%	2.00E-113	97.89
Bifidobacterium	Bifidobacterium	100%	2.00E-113	97.89
Bifidobacterium	Bifidobacterium	100%	2.00E-113	97.89
Uncultured Bific	uncultured Bifid	100%	2.00E-113	97.89
Uncultured Bific	uncultured Bifid	100%	2.00E-113	97.89
Uncultured Bific	uncultured Bifid	100%	2.00E-113	97.89
Bifidobacterium	Bifidobacterium	100%	2.00E-113	97.89
Bifidobacterium	Bifidobacterium	100%	2.00E-113	97.89
Bifidobacterium	Bifidobacterium	100%	2.00E-113	97.89
Bifidobacterium	Bifidobacterium	100%	2.00E-113	97.89
Bifidobacterium	Bifidobacterium	100%	2.00E-113	97.89
Bifidobacterium	Bifidobacterium	100%	2.00E-113	97.89
Bifidobacterium	Bifidobacterium	100%	2.00E-113	97.89
Bifidobacterium	Bifidobacterium	100%	2.00E-113	97.89
Bifidobacterium	Bifidobacterium	100%	2.00E-113	97.89
Bifidobacterium	Bifidobacterium	100%	2.00E-113	97.89
Bifidobacterium	Bifidobacterium	100%	2.00E-113	97.89
Bifidobacterium	Bifidobacterium	100%	2.00E-113	97.89
Bifidobacterium	Bifidobacterium	100%	2.00E-113	97.89
Bifidobacterium	Bifidobacterium	100%	2.00E-113	97.89
Bifidobacterium	Bifidobacterium	100%	2.00E-113	97.89
Bifidobacterium	Bifidobacterium	100%	2.00E-113	97.89
Bifidobacterium	Bifidobacterium	94%	2.00E-113	99.56
Uncultured Bific	uncultured Bifid	100%	2.00E-113	97.89
Uncultured Bific	uncultured Bifid	100%	2.00E-113	97.89
Uncultured Bific	uncultured Bifid	100%	2.00E-113	97.89
Uncultured Bific	uncultured Bifid	100%	2.00E-113	97.89
Uncultured Bific	uncultured Bifid	100%	2.00E-113	97.89
Uncultured Bific	uncultured Bifid	100%	2.00E-113	97.89
Uncultured Bific	uncultured Bifid	100%	2.00E-113	97.89
Bifidobacterium	Bifidobacterium	100%	2.00E-113	97.89
Bifidobacterium	Bifidobacterium	100%	2.00E-113	97.89
Bifidobacterium	Bifidobacterium	100%	2.00E-113	97.89
Bifidobacterium	Bifidobacterium	100%	2.00E-113	97.89
Bifidobacterium	Bifidobacterium	100%	2.00E-113	97.89
Bifidobacterium	Bifidobacterium	100%	2.00E-113	97.89
Bifidobacterium	Bifidobacterium	100%	2.00E-113	97.89
Bifidobacterium	Bifidobacterium	100%	2.00E-113	97.89
Bifidobacterium	Bifidobacterium	100%	2.00E-113	97.89
Bifidobacterium	Bifidobacterium	100%	2.00E-113	97.89
Uncultured Bific	uncultured Bifid	100%	2.00E-113	97.89

Bifidobacterium	Bifidobacterium	100%	2.00E-113	97.89
Bifidobacterium	Bifidobacterium	100%	2.00E-113	97.89
Bifidobacterium	Bifidobacterium	100%	2.00E-113	97.89
Bifidobacterium	Bifidobacterium	100%	2.00E-113	97.89
Bifidobacterium	Bifidobacterium	100%	2.00E-113	97.89
Bifidobacterium	Bifidobacterium	100%	2.00E-113	97.89
Bifidobacterium	Bifidobacterium	100%	2.00E-113	97.89
Bifidobacterium	Bifidobacterium	100%	2.00E-113	97.89
Bifidobacterium	Bifidobacterium	100%	2.00E-113	97.89
Bifidobacterium	Bifidobacterium	100%	2.00E-113	97.05
Bifidobacterium	Bifidobacterium	94%	7.00E-113	99.55
Bifidobacterium	Bifidobacterium	100%	7.00E-113	96.62
Bifidobacterium	Bifidobacterium	99%	3.00E-112	97.87
Uncultured Bifi	uncultured Bifid	97%	3.00E-112	98.28
Bifidobacterium	Bifidobacterium	99%	3.00E-112	97.87
Bifidobacterium	Bifidobacterium	99%	3.00E-112	97.87
Bifidobacterium	Bifidobacterium	100%	3.00E-112	97.47
Bifidobacterium	Bifidobacterium	99%	3.00E-112	97.87
Bifidobacterium	Bifidobacterium	99%	3.00E-112	97.87
Bifidobacterium	Bifidobacterium	99%	3.00E-112	97.87
Bifidobacterium	Bifidobacterium	100%	3.00E-112	97.89
Bifidobacterium	Bifidobacterium	100%	3.00E-112	97.47
Uncultured Bifi	uncultured Bifid	99%	3.00E-112	97.87
Bifidobacterium	Bifidobacterium	99%	3.00E-112	97.87
B.infantis (CIP (	Bifidobacterium	100%	3.00E-112	97.05
Bifidobacterium	Bifidobacterium	100%	1.00E-111	97.47
Bifidobacterium	Bifidobacterium	100%	1.00E-111	97.47
Bifidobacterium	Bifidobacterium	100%	1.00E-111	97.47
Bifidobacterium	Bifidobacterium	100%	1.00E-111	97.47
Bifidobacterium	Bifidobacterium	100%	1.00E-111	97.47
Bifidobacterium	Bifidobacterium	100%	1.00E-111	97.47
Bifidobacterium	Bifidobacterium	100%	1.00E-111	97.47
Bifidobacterium	Bifidobacterium	100%	1.00E-111	97.47
Bifidobacterium	Bifidobacterium	100%	1.00E-111	97.47
Bifidobacterium	Bifidobacterium	100%	1.00E-111	97.47
Bifidobacterium	Bifidobacterium	100%	1.00E-111	97.47
Bifidobacterium	Bifidobacterium	100%	1.00E-111	97.47
Bifidobacterium	Bifidobacterium	100%	1.00E-111	97.47
Bifidobacterium	Bifidobacterium	100%	1.00E-111	97.47
Bifidobacterium	Bifidobacterium	100%	1.00E-111	97.47
Bifidobacterium	Bifidobacterium	100%	1.00E-111	97.47
Bifidobacterium	Bifidobacterium	100%	1.00E-111	97.47
Bifidobacterium	Bifidobacterium	100%	1.00E-111	97.47
Bifidobacterium	Bifidobacterium	100%	1.00E-111	97.47

Bifidobacterium	Bifidobacterium	100%	1.00E-111	97.47
Bifidobacterium	Bifidobacterium	100%	1.00E-111	97.47
Bifidobacterium	Bifidobacterium	100%	1.00E-111	97.47
Bifidobacterium	Bifidobacterium	100%	1.00E-111	97.47
Bifidobacterium	Bifidobacterium	100%	1.00E-111	97.47
Bifidobacterium	Bifidobacterium	100%	1.00E-111	97.47
Bifidobacterium	Bifidobacterium	100%	1.00E-111	97.47
Bifidobacterium	Bifidobacterium	100%	1.00E-111	97.47
Bifidobacterium	Bifidobacterium	100%	1.00E-111	97.47
Bifidobacterium	Bifidobacterium	100%	1.00E-111	97.47
Bifidobacterium	Bifidobacterium	100%	1.00E-111	97.47
Bifidobacterium	Bifidobacterium	100%	1.00E-111	97.47
Bifidobacterium	Bifidobacterium	100%	1.00E-111	97.47
Bifidobacterium	Bifidobacterium	100%	1.00E-111	97.47
Bifidobacterium	Bifidobacterium	100%	1.00E-111	97.47
Bifidobacterium	Bifidobacterium	100%	1.00E-111	97.47
Bifidobacterium	Bifidobacterium	100%	1.00E-111	97.47
Bifidobacterium	Bifidobacterium	100%	1.00E-111	97.47
Bifidobacterium	Bifidobacterium	100%	1.00E-111	97.47
Bifidobacterium	Bifidobacterium	100%	1.00E-111	97.47
Bifidobacterium	Bifidobacterium	100%	1.00E-111	97.47
Bifidobacterium	Bifidobacterium	100%	1.00E-111	97.47
Bifidobacterium	Bifidobacterium	100%	1.00E-111	97.47
Bifidobacterium	Bifidobacterium	100%	1.00E-111	97.47
Bifidobacterium	Bifidobacterium	100%	1.00E-111	97.47
Bifidobacterium	Bifidobacterium	100%	1.00E-111	97.47
Bifidobacterium	Bifidobacterium	100%	1.00E-111	97.47
Bifidobacterium	Bifidobacterium	100%	1.00E-111	97.47
Bifidobacterium	Bifidobacterium	100%	1.00E-111	97.47
Bifidobacterium	Bifidobacterium	100%	1.00E-111	97.47
Bifidobacterium	Bifidobacterium	100%	1.00E-111	97.47
Bifidobacterium	Bifidobacterium	100%	1.00E-111	97.47
Bifidobacterium	Bifidobacterium	100%	1.00E-111	97.47
Bifidobacterium	Bifidobacterium	100%	1.00E-111	97.47
Bifidobacterium	Bifidobacterium	100%	1.00E-111	97.47
Bifidobacterium	Bifidobacterium	100%	1.00E-111	97.47
Bifidobacterium	Bifidobacterium	100%	1.00E-111	97.47
Bifidobacterium	Bifidobacterium	100%	1.00E-111	97.47
Bifidobacterium	Bifidobacterium	100%	1.00E-111	97.47
Bifidobacterium	Bifidobacterium	100%	1.00E-111	97.47
Bifidobacterium	Bifidobacterium	100%	1.00E-111	97.47
Bifidobacterium	Bifidobacterium	100%	1.00E-111	97.47
Bifidobacterium	Bifidobacterium	100%	1.00E-111	97.47
Bifidobacterium	Bifidobacterium	100%	1.00E-111	97.47

Bifidobacterium	Bifidobacterium	100%	1.00E-111	97.47
Bifidobacterium	Bifidobacterium	100%	1.00E-111	97.47
Bifidobacterium	Bifidobacterium	100%	1.00E-111	97.47
Bifidobacterium	Bifidobacterium	100%	1.00E-111	97.47
Bifidobacterium	Bifidobacterium	100%	1.00E-111	97.47
Bifidobacterium	Bifidobacterium	100%	1.00E-111	97.47
Bifidobacterium	Bifidobacterium	100%	1.00E-111	97.47
Bifidobacterium	Bifidobacterium	100%	1.00E-111	97.47
Bifidobacterium	Bifidobacterium	100%	1.00E-111	97.47
Bifidobacterium	Bifidobacterium	100%	1.00E-111	97.47
Bifidobacterium	Bifidobacterium	100%	1.00E-111	97.47
Bifidobacterium	Bifidobacterium	100%	1.00E-111	97.47
Bifidobacterium	Bifidobacterium	100%	1.00E-111	97.47
Bifidobacterium	Bifidobacterium	100%	1.00E-111	97.47
Bifidobacterium	Bifidobacterium	100%	1.00E-111	97.47
Bifidobacterium	Bifidobacterium	100%	1.00E-111	97.47
Bifidobacterium	Bifidobacterium	100%	1.00E-111	97.47
Bifidobacterium	Bifidobacterium	100%	1.00E-111	97.47
Bifidobacterium	Bifidobacterium	100%	1.00E-111	97.47
Bifidobacterium	Bifidobacterium	100%	1.00E-111	97.47
Bifidobacterium	Bifidobacterium	100%	1.00E-111	97.47
Bifidobacterium	Bifidobacterium	100%	1.00E-111	97.47
Bifidobacterium	Bifidobacterium	100%	1.00E-111	97.47
Bifidobacterium	Bifidobacterium	100%	1.00E-111	97.47
Bifidobacterium	Bifidobacterium	100%	1.00E-111	97.47
Bifidobacterium	Bifidobacterium	100%	1.00E-111	97.47
Bifidobacterium	Bifidobacterium	100%	1.00E-111	97.47
Bifidobacterium	Bifidobacterium	100%	1.00E-111	97.47
Bifidobacterium	Bifidobacterium	100%	1.00E-111	97.47
Bifidobacterium	Bifidobacterium	100%	1.00E-111	97.47
Bifidobacterium	Bifidobacterium	100%	1.00E-111	97.47
Bifidobacterium	Bifidobacterium	100%	1.00E-111	97.47
Bifidobacterium	Bifidobacterium	100%	1.00E-111	97.47
Bifidobacterium	Bifidobacterium	100%	1.00E-111	97.47
Bifidobacterium	Bifidobacterium	100%	1.00E-111	97.47
Bifidobacterium	Bifidobacterium	100%	1.00E-111	97.47
Bifidobacterium	Bifidobacterium	100%	1.00E-111	97.47
Bifidobacterium	Bifidobacterium	100%	1.00E-111	97.47
Bifidobacterium	Bifidobacterium	100%	1.00E-111	97.47
Bifidobacterium	Bifidobacterium	100%	1.00E-111	97.47
Bifidobacterium	Bifidobacterium	100%	1.00E-111	97.47
Bifidobacterium	Bifidobacterium	100%	1.00E-111	97.47
Bifidobacterium	Bifidobacterium	100%	1.00E-111	97.47
Bifidobacterium	Bifidobacterium	100%	1.00E-111	97.47

Bifidobacterium	Bifidobacterium	100%	1.00E-111	97.47
Bifidobacterium	Bifidobacterium	100%	1.00E-111	97.47
Bifidobacterium	Bifidobacterium	100%	1.00E-111	97.47
Bifidobacterium	Bifidobacterium	100%	1.00E-111	97.47
Bifidobacterium	Bifidobacterium	100%	1.00E-111	97.47
Bifidobacterium	Bifidobacterium	100%	1.00E-111	97.47
Bifidobacterium	Bifidobacterium	100%	1.00E-111	97.47
Bifidobacterium	Bifidobacterium	100%	1.00E-111	97.47
Bifidobacterium	Bifidobacterium	100%	1.00E-111	97.47
Bifidobacterium	Bifidobacterium	100%	1.00E-111	97.47
Bifidobacterium	Bifidobacterium	100%	1.00E-111	97.47
Bifidobacterium	Bifidobacterium	100%	1.00E-111	97.47
Bifidobacterium	Bifidobacterium	100%	1.00E-111	97.47
Bifidobacterium	Bifidobacterium	100%	1.00E-111	97.47
Bifidobacterium	Bifidobacterium	100%	1.00E-111	97.47
Bifidobacterium	Bifidobacterium	100%	1.00E-111	97.47
Bifidobacterium	Bifidobacterium	100%	1.00E-111	97.47
Bifidobacterium	Bifidobacterium	100%	1.00E-111	97.47
Bifidobacterium	Bifidobacterium	100%	1.00E-111	97.47
Bifidobacterium	Bifidobacterium	100%	1.00E-111	97.47
Bifidobacterium	Bifidobacterium	100%	1.00E-111	97.47
Bifidobacterium	Bifidobacterium	100%	1.00E-111	97.47
Bifidobacterium	Bifidobacterium	100%	1.00E-111	97.47
Bifidobacterium	Bifidobacterium	100%	1.00E-111	97.47
Bifidobacterium	Bifidobacterium	100%	1.00E-111	97.47
Bifidobacterium	Bifidobacterium	100%	1.00E-111	97.47
Bifidobacterium	Bifidobacterium	100%	1.00E-111	97.47
Bifidobacterium	Bifidobacterium	100%	1.00E-111	97.47
Bifidobacterium	Bifidobacterium	100%	1.00E-111	97.47
Bifidobacterium	Bifidobacterium	100%	1.00E-111	97.47
Bifidobacterium	Bifidobacterium	100%	1.00E-111	97.47
Bifidobacterium	Bifidobacterium	100%	1.00E-111	97.47
Bifidobacterium	Bifidobacterium	100%	1.00E-111	97.47
Bifidobacterium	Bifidobacterium	100%	1.00E-111	97.47
Bifidobacterium	Bifidobacterium	100%	1.00E-111	97.47
Bifidobacterium	Bifidobacterium	100%	1.00E-111	97.47
Bifidobacterium	Bifidobacterium	100%	1.00E-111	97.47
Bifidobacterium	Bifidobacterium	100%	1.00E-111	97.47
Bifidobacterium	Bifidobacterium	100%	1.00E-111	97.47
Bifidobacterium	Bifidobacterium	100%	1.00E-111	97.47
Bifidobacterium	Bifidobacterium	100%	1.00E-111	97.47
Bifidobacterium	Bifidobacterium	100%	1.00E-111	97.47
Bifidobacterium	Bifidobacterium	100%	1.00E-111	97.47
Bifidobacterium	Bifidobacterium	100%	1.00E-111	97.47

Bifidobacterium	Bifidobacterium	100%	1.00E-111	97.47
Bifidobacterium	Bifidobacterium	100%	1.00E-111	97.47
Bifidobacterium	Bifidobacterium	100%	1.00E-111	97.47
Bifidobacterium	Bifidobacterium	100%	1.00E-111	97.47
Bifidobacterium	Bifidobacterium	100%	1.00E-111	97.47
Bifidobacterium	Bifidobacterium	100%	1.00E-111	97.47
Bifidobacterium	Bifidobacterium	100%	1.00E-111	97.47
Bifidobacterium	Bifidobacterium	100%	1.00E-111	97.47
Bifidobacterium	Bifidobacterium	100%	1.00E-111	97.47
Bifidobacterium	Bifidobacterium	100%	1.00E-111	97.47
Bifidobacterium	Bifidobacterium	100%	1.00E-111	97.47
Bifidobacterium	Bifidobacterium	100%	1.00E-111	97.47
Bifidobacterium	Bifidobacterium	100%	1.00E-111	97.47
Bifidobacterium	Bifidobacterium	100%	1.00E-111	97.47
Bifidobacterium	Bifidobacterium	100%	1.00E-111	97.47
Bifidobacterium	Bifidobacterium	100%	1.00E-111	97.47
Bifidobacterium	Bifidobacterium	100%	1.00E-111	97.47
Bifidobacterium	Bifidobacterium	100%	1.00E-111	97.47
Bifidobacterium	Bifidobacterium	100%	1.00E-111	97.47
Bifidobacterium	Bifidobacterium	100%	1.00E-111	97.47
Uncultured Bific	uncultured Bifid	100%	1.00E-111	97.47
Bifidobacterium	Bifidobacterium	100%	1.00E-111	97.47
Bifidobacterium	Bifidobacterium	100%	1.00E-111	97.47
Bifidobacterium	Bifidobacterium	100%	1.00E-111	97.47
Bifidobacterium	Bifidobacterium	100%	1.00E-111	97.47
Bifidobacterium	Bifidobacterium	100%	1.00E-111	97.47
Bifidobacterium	Bifidobacterium	100%	1.00E-111	97.47
Bifidobacterium	Bifidobacterium	100%	1.00E-111	97.47
Bifidobacterium	Bifidobacterium	100%	1.00E-111	97.47
Uncultured Bific	uncultured Bifid	100%	1.00E-111	97.47
Bifidobacterium	Bifidobacterium	100%	1.00E-111	97.47
Bifidobacterium	Bifidobacterium	100%	1.00E-111	97.47
Bifidobacterium	Bifidobacterium	100%	1.00E-111	97.47
Bifidobacterium	Bifidobacterium	100%	1.00E-111	97.47
Bifidobacterium	Bifidobacterium	100%	1.00E-111	97.47
Bifidobacterium	Bifidobacterium	100%	1.00E-111	97.47
Bifidobacterium	Bifidobacterium	100%	1.00E-111	97.47
Bifidobacterium	Bifidobacterium	100%	1.00E-111	97.47
Bifidobacterium	Bifidobacterium	100%	1.00E-111	97.47
Bifidobacterium	Bifidobacterium	100%	1.00E-111	97.47
Bifidobacterium	Bifidobacterium	100%	1.00E-111	96.62
Bifidobacterium	Bifidobacterium	100%	1.00E-111	97.47
Bifidobacterium	Bifidobacterium	100%	1.00E-111	97.47
Bifidobacterium	Bifidobacterium	100%	1.00E-111	97.47

Bifidobacterium	Bifidobacterium	100%	1.00E-111	97.47
Bifidobacterium	Bifidobacterium	100%	1.00E-111	97.47
Bifidobacterium	Bifidobacterium	100%	1.00E-111	97.47
Bifidobacterium	Bifidobacterium	100%	1.00E-111	97.47
Bifidobacterium	Bifidobacterium	100%	1.00E-111	97.48
Bifidobacterium	Bifidobacterium	100%	1.00E-111	97.47
Bifidobacterium	Bifidobacterium	100%	1.00E-111	97.47
Bifidobacterium	Bifidobacterium	100%	1.00E-111	97.47
Bifidobacterium	Bifidobacterium	100%	1.00E-111	97.47
Bifidobacterium	Bifidobacterium	100%	1.00E-111	97.47
Bifidobacterium	Bifidobacterium	100%	1.00E-111	97.47
Bifidobacterium	Bifidobacterium	100%	1.00E-111	97.47
Bifidobacterium	Bifidobacterium	100%	1.00E-111	97.47
Bifidobacterium	Bifidobacterium	93%	1.00E-111	99.55
Bifidobacterium	Bifidobacterium	100%	1.00E-111	97.47
Bifidobacterium	Bifidobacterium	97%	1.00E-111	98.28
Bifidobacterium	Bifidobacterium	100%	1.00E-111	97.47
Bifidobacterium	Bifidobacterium	100%	1.00E-111	97.47
Bifidobacterium	Bifidobacterium	100%	1.00E-111	97.47
Bifidobacterium	Bifidobacterium	100%	1.00E-111	97.47
Bifidobacterium	Bifidobacterium	100%	1.00E-111	97.47
Bifidobacterium	Bifidobacterium	100%	1.00E-111	97.47
Bifidobacterium	Bifidobacterium	100%	1.00E-111	97.47
Bifidobacterium	Bifidobacterium	100%	1.00E-111	97.47
Bifidobacterium	Bifidobacterium	100%	1.00E-111	97.47
Bifidobacterium	Bifidobacterium	100%	1.00E-111	97.47
Bifidobacterium	Bifidobacterium	100%	1.00E-111	97.47
Bifidobacterium	Bifidobacterium	100%	1.00E-111	97.47
Bifidobacterium	Bifidobacterium	100%	1.00E-111	97.47
Bifidobacterium	Bifidobacterium	100%	1.00E-111	97.47
Bifidobacterium	Bifidobacterium	100%	1.00E-111	97.47
Bifidobacterium	Bifidobacterium	100%	1.00E-111	97.47
Bifidobacterium	Bifidobacterium	100%	1.00E-111	97.47
Bifidobacterium	Bifidobacterium	100%	1.00E-111	97.47
Bifidobacterium	Bifidobacterium	100%	1.00E-111	97.47
Bifidobacterium	Bifidobacterium	100%	1.00E-111	97.47
Bifidobacterium	Bifidobacterium	100%	1.00E-111	97.47
Bifidobacterium	Bifidobacterium	100%	1.00E-111	97.47
Bifidobacterium	Bifidobacterium	100%	1.00E-111	97.47
Bifidobacterium	Bifidobacterium	100%	1.00E-111	97.47
Bifidobacterium	Bifidobacterium	100%	1.00E-111	97.47
Bifidobacterium	Bifidobacterium	100%	1.00E-111	97.47
Bifidobacterium	Bifidobacterium	100%	1.00E-111	97.47
Bifidobacterium	Bifidobacterium	100%	1.00E-111	97.47

Bifidobacterium	Bifidobacterium	100%	1.00E-111	97.47
Bifidobacterium	Bifidobacterium	100%	1.00E-111	97.47
Bifidobacterium	Bifidobacterium	100%	1.00E-111	97.47
Bifidobacterium	Bifidobacterium	100%	1.00E-111	97.47
Bifidobacterium	Bifidobacterium	100%	1.00E-111	97.47
Bifidobacterium	Bifidobacterium	100%	1.00E-111	97.47
Bifidobacterium	Bifidobacterium	100%	1.00E-111	97.47
Bifidobacterium	Bifidobacterium	100%	1.00E-111	97.47
Bifidobacterium	Bifidobacterium	100%	1.00E-111	97.47
Bifidobacterium	Bifidobacterium	100%	1.00E-111	97.47
Bifidobacterium	Bifidobacterium	100%	1.00E-111	97.47
Bifidobacterium	Bifidobacterium	100%	1.00E-111	97.47
Bifidobacterium	Bifidobacterium	100%	1.00E-111	97.47
Bifidobacterium	Bifidobacterium	100%	1.00E-111	97.47
Bifidobacterium	Bifidobacterium	100%	1.00E-111	97.47
Bifidobacterium	Bifidobacterium	100%	1.00E-111	97.47
Bifidobacterium	Bifidobacterium	100%	1.00E-111	97.47
Bifidobacterium	Bifidobacterium	100%	1.00E-111	97.47
Bifidobacterium	Bifidobacterium	100%	1.00E-111	97.47
Bifidobacterium	Bifidobacterium	100%	1.00E-111	97.47
Bifidobacterium	Bifidobacterium	100%	1.00E-111	97.47
Bifidobacterium	Bifidobacterium	100%	1.00E-111	97.47
Bifidobacterium	Bifidobacterium	100%	1.00E-111	97.47
Bifidobacterium	Bifidobacterium	100%	1.00E-111	97.47
Bifidobacterium	Bifidobacterium	100%	1.00E-111	97.47
Bifidobacterium	Bifidobacterium	100%	1.00E-111	97.47
Bifidobacterium	Bifidobacterium	100%	1.00E-111	97.47
Bifidobacterium	Bifidobacterium	100%	1.00E-111	97.47
Bifidobacterium	Bifidobacterium	100%	1.00E-111	97.47
Bifidobacterium	Bifidobacterium	100%	1.00E-111	97.47
Bifidobacterium	Bifidobacterium	100%	1.00E-111	97.47
Bifidobacterium	Bifidobacterium	100%	1.00E-111	97.47
Bifidobacterium	Bifidobacterium	100%	1.00E-111	97.47
Bifidobacterium	Bifidobacterium	100%	1.00E-111	97.47
Bifidobacterium	Bifidobacterium	100%	1.00E-111	97.47
Bifidobacterium	Bifidobacterium	100%	1.00E-111	97.47
Bifidobacterium	Bifidobacterium	100%	1.00E-111	97.47
Bifidobacterium	Bifidobacterium	100%	1.00E-111	97.47
Bifidobacterium	Bifidobacterium	100%	1.00E-111	97.47
Bifidobacterium	Bifidobacterium	100%	1.00E-111	97.47
Bifidobacterium	Bifidobacterium	100%	1.00E-111	97.47
Bifidobacterium	Bifidobacterium	100%	1.00E-111	97.47
Bifidobacterium	Bifidobacterium	100%	1.00E-111	97.47
Bifidobacterium	Bifidobacterium	100%	1.00E-111	97.47

Bifidobacterium	Bifidobacterium	100%	1.00E-111	97.47
Bifidobacterium	Bifidobacterium	100%	1.00E-111	97.47
Bifidobacterium	Bifidobacterium	100%	1.00E-111	97.47
Bifidobacterium	Bifidobacterium	100%	1.00E-111	97.47
Bifidobacterium	Bifidobacterium	100%	1.00E-111	97.47
Bifidobacterium	Bifidobacterium	100%	1.00E-111	97.47
Bifidobacterium	Bifidobacterium	100%	1.00E-111	97.47
Uncultured Bific	uncultured Bifid	100%	1.00E-111	97.47
Bifidobacterium	Bifidobacterium	100%	1.00E-111	97.47
Bifidobacterium	Bifidobacterium	100%	1.00E-111	97.47
Bifidobacterium	Bifidobacterium	100%	1.00E-111	97.47
Bifidobacterium	Bifidobacterium	100%	1.00E-111	97.47
Bifidobacterium	Bifidobacterium	100%	1.00E-111	97.47
Bifidobacterium	Bifidobacterium	100%	1.00E-111	97.47
Bifidobacterium	Bifidobacterium	100%	1.00E-111	97.48
Bifidobacterium	Bifidobacterium	100%	1.00E-111	97.47
Bifidobacterium	Bifidobacterium	100%	1.00E-111	97.47
Bifidobacterium	Bifidobacterium	100%	1.00E-111	97.47
Bifidobacterium	Bifidobacterium	100%	1.00E-111	97.47
Bifidobacterium	Bifidobacterium	100%	1.00E-111	97.47
Bifidobacterium	Bifidobacterium	100%	1.00E-111	97.47
Bifidobacterium	Bifidobacterium	100%	1.00E-111	97.47
Bifidobacterium	Bifidobacterium	100%	1.00E-111	97.47
Bifidobacterium	Bifidobacterium	100%	1.00E-111	97.47
Bifidobacterium	Bifidobacterium	100%	1.00E-111	97.47
Bifidobacterium	Bifidobacterium	100%	1.00E-111	97.47
Bifidobacterium	Bifidobacterium	100%	1.00E-111	97.47
Bifidobacterium	Bifidobacterium	100%	1.00E-111	97.47
Bifidobacterium	Bifidobacterium	100%	1.00E-111	97.47
Bifidobacterium	Bifidobacterium	100%	1.00E-111	97.47
Bifidobacterium	Bifidobacterium	100%	1.00E-111	97.47
Bifidobacterium	Bifidobacterium	100%	1.00E-111	97.47
Bifidobacterium	Bifidobacterium	100%	1.00E-111	97.47
Uncultured Bific	uncultured Bifid	100%	1.00E-111	97.47
Uncultured Bific	uncultured Bifid	100%	1.00E-111	97.47
Uncultured Bific	uncultured Bifid	100%	1.00E-111	97.47
Uncultured Bific	uncultured Bifid	100%	1.00E-111	97.47
Uncultured Bific	uncultured Bifid	100%	1.00E-111	97.47
Uncultured Bific	uncultured Bifid	100%	1.00E-111	97.47
Uncultured Bific	uncultured Bifid	100%	1.00E-111	97.47
Bifidobacterium	Bifidobacterium	100%	1.00E-111	97.47
Uncultured Bific	uncultured Bifid	100%	1.00E-111	97.47
Bifidobacterium	Bifidobacterium	100%	1.00E-111	97.47
Bifidobacterium	Bifidobacterium	100%	1.00E-111	97.47

Bifidobacterium	Bifidobacterium	100%	1.00E-111	97.47
Bifidobacterium	Bifidobacterium	100%	1.00E-111	97.47
Bifidobacterium	Bifidobacterium	100%	1.00E-111	96.62
Bifidobacterium	Bifidobacterium	100%	1.00E-111	97.47
Bifidobacterium	Bifidobacterium	100%	1.00E-111	97.47
Bifidobacterium	Bifidobacterium	100%	1.00E-111	97.47
Bifidobacterium	Bifidobacterium	100%	1.00E-111	97.47
Bifidobacterium	Bifidobacterium	100%	1.00E-111	97.47
Bifidobacterium	Bifidobacterium	100%	1.00E-111	97.47
Bifidobacterium	Bifidobacterium	100%	1.00E-111	97.47
Bifidobacterium	Bifidobacterium	100%	1.00E-111	97.47
B.lactis DNA 16	Bifidobacterium	100%	1.00E-111	97.47
Bifidobacterium	Bifidobacterium	100%	1.00E-111	97.47
Bifidobacterium	Bifidobacterium	99%	3.00E-111	97.45
Bifidobacterium	Bifidobacterium	100%	3.00E-111	96.2
Bifidobacterium	Bifidobacterium	96%	3.00E-111	98.27
Bifidobacterium	Bifidobacterium	98%	3.00E-111	97.85
Uncultured Bific	uncultured Bifid	99%	3.00E-111	97.46
Bifidobacterium	Bifidobacterium	99%	1.00E-110	97.45
Bifidobacterium	Bifidobacterium	99%	1.00E-110	97.45
Bifidobacterium	Bifidobacterium	99%	1.00E-110	97.45
Bifidobacterium	Bifidobacterium	99%	1.00E-110	97.45
Bifidobacterium	Bifidobacterium	99%	1.00E-110	97.45
Bifidobacterium	Bifidobacterium	99%	1.00E-110	97.45
Bifidobacterium	Bifidobacterium	99%	1.00E-110	97.45
Bifidobacterium	Bifidobacterium	99%	1.00E-110	97.45
Bifidobacterium	Bifidobacterium	99%	1.00E-110	97.45
Bifidobacterium	Bifidobacterium	99%	1.00E-110	97.45
Bifidobacterium	Bifidobacterium	99%	1.00E-110	97.45
Bifidobacterium	Bifidobacterium	99%	1.00E-110	97.45
Bifidobacterium	Bifidobacterium	99%	1.00E-110	97.45
Bifidobacterium	Bifidobacterium	99%	1.00E-110	97.45
Bifidobacterium	Bifidobacterium	99%	1.00E-110	97.45
Bifidobacterium	Bifidobacterium	99%	1.00E-110	97.45
Bifidobacterium	Bifidobacterium	99%	1.00E-110	97.45
Bifidobacterium	Bifidobacterium	99%	1.00E-110	97.45
Bifidobacterium	Bifidobacterium	99%	1.00E-110	97.45
Bifidobacterium	Bifidobacterium	99%	1.00E-110	97.45
Bifidobacterium	Bifidobacterium	99%	1.00E-110	97.45
Bifidobacterium	Bifidobacterium	99%	1.00E-110	97.45
Bifidobacterium	Bifidobacterium	99%	1.00E-110	97.45
Bifidobacterium	Bifidobacterium	99%	1.00E-110	97.45
Bifidobacterium	Bifidobacterium	99%	1.00E-110	97.45
Bifidobacterium	Bifidobacterium	99%	1.00E-110	97.45

Bifidobacterium	Bifidobacterium	99%	1.00E-110	97.45
Bifidobacterium	Bifidobacterium	99%	1.00E-110	97.45
Bifidobacterium	Bifidobacterium	99%	1.00E-110	97.45
Bifidobacterium	Bifidobacterium	99%	1.00E-110	97.45
Bifidobacterium	Bifidobacterium	99%	1.00E-110	97.45
Bifidobacterium	Bifidobacterium	99%	1.00E-110	97.45
Bifidobacterium	Bifidobacterium	99%	1.00E-110	97.45
Bifidobacterium	Bifidobacterium	99%	1.00E-110	97.45
Bifidobacterium	Bifidobacterium	99%	1.00E-110	97.45
Bifidobacterium	Bifidobacterium	99%	1.00E-110	97.45
Bifidobacterium	Bifidobacterium	99%	1.00E-110	97.45
Bifidobacterium	Bifidobacterium	99%	1.00E-110	97.45
Bifidobacterium	Bifidobacterium	99%	1.00E-110	97.45
Bifidobacterium	Bifidobacterium	99%	1.00E-110	97.45
Bifidobacterium	Bifidobacterium	99%	1.00E-110	97.45
Bifidobacterium	Bifidobacterium	99%	1.00E-110	97.45
Bifidobacterium	Bifidobacterium	99%	1.00E-110	97.45
Bifidobacterium	Bifidobacterium	99%	1.00E-110	97.45
Bifidobacterium	Bifidobacterium	99%	1.00E-110	97.45
Bifidobacterium	Bifidobacterium	99%	1.00E-110	97.45
Bifidobacterium	Bifidobacterium	99%	1.00E-110	97.45
Bifidobacterium	Bifidobacterium	99%	1.00E-110	97.45
Bifidobacterium	Bifidobacterium	99%	1.00E-110	97.45
Bifidobacterium	Bifidobacterium	99%	1.00E-110	97.45
Bifidobacterium	Bifidobacterium	99%	1.00E-110	97.45
Bifidobacterium	Bifidobacterium	99%	1.00E-110	97.45
Bifidobacterium	Bifidobacterium	99%	1.00E-110	97.45
Bifidobacterium	Bifidobacterium	99%	1.00E-110	97.45
Bifidobacterium	Bifidobacterium	99%	1.00E-110	97.45
Bifidobacterium	Bifidobacterium	99%	1.00E-110	97.45
Bifidobacterium	Bifidobacterium	99%	1.00E-110	97.45
Bifidobacterium	Bifidobacterium	99%	1.00E-110	97.45
Bifidobacterium	Bifidobacterium	99%	1.00E-110	97.45
Bifidobacterium	Bifidobacterium	99%	1.00E-110	97.45
Bifidobacterium	Bifidobacterium	99%	1.00E-110	97.45
Bifidobacterium	Bifidobacterium	99%	1.00E-110	97.45
Bifidobacterium	Bifidobacterium	99%	1.00E-110	97.45
Bifidobacterium	Bifidobacterium	99%	1.00E-110	97.45
Bifidobacterium	Bifidobacterium	99%	1.00E-110	97.45
Bifidobacterium	Bifidobacterium	99%	1.00E-110	97.45
Bifidobacterium	Bifidobacterium	99%	1.00E-110	97.45
Bifidobacterium	Bifidobacterium	99%	1.00E-110	97.45
Bifidobacterium	Bifidobacterium	99%	1.00E-110	97.45
Bifidobacterium	Bifidobacterium	99%	1.00E-110	97.45

Bifidobacterium	Bifidobacterium	99%	1.00E-110	97.45
Bifidobacterium	Bifidobacterium	99%	1.00E-110	97.45
Bifidobacterium	Bifidobacterium	99%	1.00E-110	97.45
Bifidobacterium	Bifidobacterium	99%	1.00E-110	97.45
Bifidobacterium	Bifidobacterium	99%	1.00E-110	97.45
Bifidobacterium	Bifidobacterium	99%	1.00E-110	97.45
Bifidobacterium	Bifidobacterium	99%	1.00E-110	97.45
Bifidobacterium	Bifidobacterium	99%	1.00E-110	97.45
Bifidobacterium	Bifidobacterium	99%	1.00E-110	97.45
Bifidobacterium	Bifidobacterium	99%	1.00E-110	97.45
Bifidobacterium	Bifidobacterium	99%	1.00E-110	97.45
Bifidobacterium	Bifidobacterium	99%	1.00E-110	97.45
Uncultured Bific	uncultured Bifid	99%	1.00E-110	97.45
Bifidobacterium	Bifidobacterium	99%	1.00E-110	97.45
Bifidobacterium	Bifidobacterium	99%	1.00E-110	97.45
Bifidobacterium	Bifidobacterium	99%	1.00E-110	97.45
Bifidobacterium	Bifidobacterium	99%	1.00E-110	97.45
Bifidobacterium	Bifidobacterium	99%	1.00E-110	97.45
Bifidobacterium	Bifidobacterium	99%	1.00E-110	97.45
Bifidobacterium	Bifidobacterium	99%	1.00E-110	97.46
Bifidobacterium	Bifidobacterium	99%	1.00E-110	97.45
Bifidobacterium	Bifidobacterium	99%	1.00E-110	97.45
Bifidobacterium	Bifidobacterium	99%	1.00E-110	97.45
Bifidobacterium	Bifidobacterium	99%	1.00E-110	97.45
Bifidobacterium	Bifidobacterium	99%	1.00E-110	97.45
Uncultured Bific	uncultured Bifid	99%	1.00E-110	97.45
Bifidobacterium	Bifidobacterium	99%	1.00E-110	97.45
Bifidobacterium	Bifidobacterium	99%	1.00E-110	97.45
Bifidobacterium	Bifidobacterium	99%	1.00E-110	97.45
Bifidobacterium	Bifidobacterium	99%	1.00E-110	97.45
Bifidobacterium	Bifidobacterium	99%	1.00E-110	97.45
Bifidobacterium	Bifidobacterium	99%	1.00E-110	97.45
Bifidobacterium	Bifidobacterium	99%	1.00E-110	97.45
Bifidobacterium	Bifidobacterium	99%	1.00E-110	97.45
Bifidobacterium	Bifidobacterium	99%	1.00E-110	97.45
Uncultured Bific	uncultured Bifid	99%	1.00E-110	97.45
Uncultured Bific	uncultured Bifid	99%	1.00E-110	97.45
Bifidobacterium	Bifidobacterium	99%	1.00E-110	97.45
Bifidobacterium	Bifidobacterium	99%	1.00E-110	97.46
Bifidobacterium	Bifidobacterium	99%	1.00E-110	97.45
Bifidobacterium	Bifidobacterium	99%	1.00E-110	97.45
Bifidobacterium	Bifidobacterium	99%	1.00E-110	97.45
Bifidobacterium	Bifidobacterium	99%	1.00E-110	97.45
Bifidobacterium	Bifidobacterium	99%	1.00E-110	97.45

Bifidobacterium	Bifidobacterium	99%	1.00E-110	97.45
Bifidobacterium	Bifidobacterium	99%	1.00E-110	97.45
Bifidobacterium	Bifidobacterium	99%	1.00E-110	97.45
Bifidobacterium	Bifidobacterium	99%	1.00E-110	97.45
Bifidobacterium	Bifidobacterium	99%	1.00E-110	97.45
Bifidobacterium	Bifidobacterium	99%	1.00E-110	97.45
Bifidobacterium	Bifidobacterium	99%	1.00E-110	97.45
Bifidobacterium	Bifidobacterium	99%	1.00E-110	97.45
Bifidobacterium	Bifidobacterium	99%	1.00E-110	97.45
Bifidobacterium	Bifidobacterium	99%	1.00E-110	97.45
Bifidobacterium	Bifidobacterium	99%	1.00E-110	97.45
Bifidobacterium	Bifidobacterium	99%	1.00E-110	97.45
Bifidobacterium	Bifidobacterium	99%	1.00E-110	97.45
Bifidobacterium	Bifidobacterium	99%	1.00E-110	97.45
Bifidobacterium	Bifidobacterium	99%	1.00E-110	97.45
Bifidobacterium	Bifidobacterium	99%	1.00E-110	97.45
Bifidobacterium	Bifidobacterium	99%	1.00E-110	97.45
Bifidobacterium	Bifidobacterium	99%	1.00E-110	97.45
Bifidobacterium	Bifidobacterium	99%	1.00E-110	97.45
Bifidobacterium	Bifidobacterium	99%	1.00E-110	97.45
Bifidobacterium	Bifidobacterium	99%	1.00E-110	97.45
Bifidobacterium	Bifidobacterium	99%	1.00E-110	97.45
Bifidobacterium	Bifidobacterium	99%	1.00E-110	97.45
Bifidobacterium	Bifidobacterium	100%	1.00E-110	97.05
Uncultured Bifid	uncultured Bifid	99%	1.00E-110	97.45
Uncultured Bifid	uncultured Bifid	99%	1.00E-110	97.45
Bifidobacterium	Bifidobacterium	99%	1.00E-110	97.45
Bifidobacterium	Bifidobacterium	99%	1.00E-110	97.45
Bifidobacterium	Bifidobacterium	99%	1.00E-110	97.45
Bifidobacterium	Bifidobacterium	99%	1.00E-110	97.45
Bifidobacterium	Bifidobacterium	99%	1.00E-110	97.45
Bifidobacterium	Bifidobacterium	99%	1.00E-110	97.45
Bifidobacterium	Bifidobacterium	99%	1.00E-110	97.45
Bifidobacterium	Bifidobacterium	99%	1.00E-110	97.45
Bifidobacterium	Bifidobacterium	99%	1.00E-110	97.45
Bifidobacterium	Bifidobacterium	99%	1.00E-110	97.45
Bifidobacterium	Bifidobacterium	99%	1.00E-110	97.45
Bifidobacterium	Bifidobacterium	99%	1.00E-110	97.45
Bifidobacterium	Bifidobacterium	99%	1.00E-110	97.45
Bifidobacterium	Bifidobacterium	99%	1.00E-110	97.45
Bifidobacterium	Bifidobacterium	99%	1.00E-110	97.45
Bifidobacterium	Bifidobacterium	99%	1.00E-110	97.45
Bifidobacterium	Bifidobacterium	99%	1.00E-110	97.45
Bifidobacterium	Bifidobacterium	99%	1.00E-110	97.45

Bifidobacterium	Bifidobacterium	99%	1.00E-110	97.45
Bifidobacterium	Bifidobacterium	99%	1.00E-110	97.45
Bifidobacterium	Bifidobacterium	99%	1.00E-110	97.45
Bifidobacterium	Bifidobacterium	99%	1.00E-110	97.45
Bifidobacterium	Bifidobacterium	99%	1.00E-110	97.45
Bifidobacterium	Bifidobacterium	99%	1.00E-110	97.45
Bifidobacterium	Bifidobacterium	99%	1.00E-110	97.45
Bifidobacterium	Bifidobacterium	99%	1.00E-110	97.45
Bifidobacterium	Bifidobacterium	99%	1.00E-110	97.45
Bifidobacterium	Bifidobacterium	99%	1.00E-110	97.45
Bifidobacterium	Bifidobacterium	99%	1.00E-110	97.45
Bifidobacterium	Bifidobacterium	99%	1.00E-110	97.45
Bifidobacterium	Bifidobacterium	99%	1.00E-110	97.45
Bifidobacterium	Bifidobacterium	99%	1.00E-110	97.45
Bifidobacterium	Bifidobacterium	99%	1.00E-110	97.45
Bifidobacterium	Bifidobacterium	99%	1.00E-110	97.45
Bifidobacterium	Bifidobacterium	99%	1.00E-110	97.45
Bifidobacterium	Bifidobacterium	99%	1.00E-110	97.45
Bifidobacterium	Bifidobacterium	99%	1.00E-110	97.45
Bifidobacterium	Bifidobacterium	99%	1.00E-110	97.45
Bifidobacterium	Bifidobacterium	99%	1.00E-110	97.45
Bifidobacterium	Bifidobacterium	92%	1.00E-110	99.55
Bifidobacterium	Bifidobacterium	99%	1.00E-110	97.45
Bifidobacterium	Bifidobacterium	99%	1.00E-110	97.45
Bifidobacterium	Bifidobacterium	99%	1.00E-110	97.45
Bifidobacterium	Bifidobacterium	99%	1.00E-110	97.45
Bifidobacterium	Bifidobacterium	99%	1.00E-110	97.45
Bifidobacterium	Bifidobacterium	99%	1.00E-110	97.45
Bifidobacterium	Bifidobacterium	99%	1.00E-110	97.45
Bifidobacterium	Bifidobacterium	99%	1.00E-110	97.45
Bifidobacterium	Bifidobacterium	99%	1.00E-110	97.45
Bifidobacterium	Bifidobacterium	99%	1.00E-110	97.45
Bifidobacterium	Bifidobacterium	99%	1.00E-110	97.45
Bifidobacterium	Bifidobacterium	99%	1.00E-110	97.45
Bifidobacterium	Bifidobacterium	99%	1.00E-110	97.45
Bifidobacterium	Bifidobacterium	100%	1.00E-110	97.05
Bifidobacterium	Bifidobacterium	100%	1.00E-110	97.05
Bifidobacterium	Bifidobacterium	99%	1.00E-110	97.45
Bifidobacterium	Bifidobacterium	99%	1.00E-110	97.45
Bifidobacterium	Bifidobacterium	99%	1.00E-110	97.45
Bifidobacterium	Bifidobacterium	99%	1.00E-110	97.45
Bifidobacterium	Bifidobacterium	99%	1.00E-110	97.45
Bifidobacterium	Bifidobacterium	99%	1.00E-110	97.45
Bifidobacterium	Bifidobacterium	99%	1.00E-110	97.45

Bifidobacterium	Bifidobacterium	99%	1.00E-110	97.45
Bifidobacterium	Bifidobacterium	99%	1.00E-110	97.45
Bifidobacterium	Bifidobacterium	100%	1.00E-110	97.05
Uncultured Bifid	uncultured Bifid	99%	1.00E-110	97.45
Uncultured Bifid	uncultured Bifid	99%	1.00E-110	97.45
Bifidobacterium	Bifidobacterium	99%	1.00E-110	97.45
Bifidobacterium	Bifidobacterium	99%	1.00E-110	97.45
Bifidobacterium	Bifidobacterium	99%	1.00E-110	97.45
Bifidobacterium	Bifidobacterium	99%	1.00E-110	97.45
16S rRNA [Bifid	Bifidobacterium	99%	1.00E-110	97.45
Bifidobacterium	Bifidobacterium	99%	1.00E-110	97.45
Bifidobacterium	Bifidobacterium	100%	4.00E-110	97.05
Bifidobacterium	Bifidobacterium	100%	4.00E-110	97.06
Bifidobacterium	Bifidobacterium	100%	4.00E-110	97.05
Bifidobacterium	Bifidobacterium	100%	4.00E-110	97.05
Bifidobacterium	Bifidobacterium	100%	4.00E-110	97.05
Bifidobacterium	Bifidobacterium	100%	4.00E-110	97.05
Bifidobacterium	Bifidobacterium	100%	4.00E-110	97.05
Bifidobacterium	Bifidobacterium	100%	4.00E-110	97.05
Bifidobacterium	Bifidobacterium	100%	4.00E-110	97.05
Bifidobacterium	Bifidobacterium	100%	4.00E-110	97.05
Bifidobacterium	Bifidobacterium	100%	4.00E-110	97.05
Bifidobacterium	Bifidobacterium	100%	4.00E-110	97.05
Bifidobacterium	Bifidobacterium	100%	4.00E-110	97.05
Bifidobacterium	Bifidobacterium	100%	4.00E-110	97.05
Bifidobacterium	Bifidobacterium	100%	4.00E-110	97.05
Bifidobacterium	Bifidobacterium	100%	4.00E-110	97.05
Bifidobacterium	Bifidobacterium	100%	4.00E-110	97.06
Bifidobacterium	Bifidobacterium	100%	4.00E-110	97.05
Bifidobacterium	Bifidobacterium	100%	4.00E-110	97.05
Bifidobacterium	Bifidobacterium	100%	4.00E-110	97.06
Bifidobacterium	Bifidobacterium	100%	4.00E-110	97.05
Bifidobacterium	Bifidobacterium	100%	4.00E-110	97.05
Bifidobacterium	Bifidobacterium	100%	4.00E-110	97.05
Bifidobacterium	Bifidobacterium	100%	4.00E-110	97.05
Bifidobacterium	Bifidobacterium	100%	4.00E-110	97.05
Bifidobacterium	Bifidobacterium	100%	4.00E-110	97.05
Bifidobacterium	Bifidobacterium	100%	4.00E-110	97.05
Bifidobacterium	Bifidobacterium	100%	4.00E-110	97.05
Bifidobacterium	Bifidobacterium	100%	4.00E-110	97.05
Bifidobacterium	Bifidobacterium	100%	4.00E-110	97.05
Bifidobacterium	Bifidobacterium	100%	4.00E-110	97.05
Bifidobacterium	Bifidobacterium	100%	4.00E-110	97.05
Bifidobacterium	Bifidobacterium	100%	4.00E-110	97.05

Bifidobacterium	Bifidobacterium	100%	4.00E-110	97.05
Bifidobacterium	Bifidobacterium	100%	4.00E-110	97.05
Bifidobacterium	Bifidobacterium	100%	4.00E-110	97.05
Uncultured Bific	uncultured Bifid	100%	4.00E-110	97.05
Bifidobacterium	Bifidobacterium	100%	4.00E-110	97.05
Bifidobacterium	Bifidobacterium	100%	4.00E-110	97.05
Bifidobacterium	Bifidobacterium	100%	4.00E-110	97.05
Bifidobacterium	Bifidobacterium	100%	4.00E-110	97.05
Bifidobacterium	Bifidobacterium	100%	4.00E-110	97.05
Bifidobacterium	Bifidobacterium	100%	4.00E-110	97.05
Bifidobacterium	Bifidobacterium	100%	4.00E-110	97.05
Bifidobacterium	Bifidobacterium	100%	4.00E-110	97.05
Uncultured Bific	uncultured Bifid	100%	4.00E-110	97.05
Bifidobacterium	Bifidobacterium	100%	4.00E-110	97.05
Bifidobacterium	Bifidobacterium	100%	4.00E-110	97.05
Bifidobacterium	Bifidobacterium	100%	4.00E-110	97.05
Bifidobacterium	Bifidobacterium	100%	4.00E-110	97.05
Bifidobacterium	Bifidobacterium	100%	4.00E-110	97.05
Bifidobacterium	Bifidobacterium	100%	4.00E-110	97.05
Bifidobacterium	Bifidobacterium	100%	4.00E-110	97.06
Bifidobacterium	Bifidobacterium	100%	4.00E-110	97.05
Bifidobacterium	Bifidobacterium	100%	4.00E-110	97.05
Bifidobacterium	Bifidobacterium	100%	4.00E-110	97.05
Bifidobacterium	Bifidobacterium	100%	4.00E-110	97.05
Uncultured Bific	uncultured Bifid	92%	4.00E-110	99.54
Bifidobacterium	Bifidobacterium	100%	4.00E-110	97.05
Uncultured Bific	uncultured Bifid	100%	4.00E-110	97.05
Uncultured Bific	uncultured Bifid	100%	4.00E-110	97.05
Uncultured Bific	uncultured Bifid	100%	4.00E-110	97.05
Uncultured Bific	uncultured Bifid	100%	4.00E-110	97.06
Bifidobacterium	Bifidobacterium	100%	4.00E-110	97.05
Bifidobacterium	Bifidobacterium	100%	4.00E-110	97.05
Bifidobacterium	Bifidobacterium	100%	4.00E-110	97.05
Bifidobacterium	Bifidobacterium	100%	4.00E-110	97.05
Bifidobacterium	Bifidobacterium	94%	2.00E-109	98.67
Bifidobacterium	Bifidobacterium	90%	2.00E-109	100
Bifidobacterium	Bifidobacterium	99%	2.00E-109	97.02
Uncultured Bific	uncultured Bifid	99%	2.00E-109	97.03
Bifidobacterium	Bifidobacterium	100%	2.00E-109	97.05
Bifidobacterium	Bifidobacterium	91%	2.00E-109	99.54
Bifidobacterium	Bifidobacterium	100%	2.00E-109	97.05
Bifidobacterium	Bifidobacterium	94%	2.00E-109	98.65
Bifidobacterium	Bifidobacterium	99%	6.00E-109	97.02
Uncultured Bific	uncultured Bifid	97%	6.00E-109	97.41

Bifidobacterium	Bifidobacterium	99%	6.00E-109	97.02
Bifidobacterium	Bifidobacterium	99%	6.00E-109	97.02
Bifidobacterium	Bifidobacterium	99%	6.00E-109	97.02
Bifidobacterium	Bifidobacterium	99%	6.00E-109	97.02
Bifidobacterium	Bifidobacterium	99%	6.00E-109	97.02
Bifidobacterium	Bifidobacterium	99%	6.00E-109	97.03
Bifidobacterium	Bifidobacterium	99%	6.00E-109	97.02
Bifidobacterium	Bifidobacterium	99%	6.00E-109	97.02
Bifidobacterium	Bifidobacterium	97%	6.00E-109	97.41
Bifidobacterium	Bifidobacterium	99%	6.00E-109	97.02
Bifidobacterium	Bifidobacterium	99%	6.00E-109	97.02
Bifidobacterium	Bifidobacterium	100%	6.00E-109	96.2
Bifidobacterium	Bifidobacterium	91%	6.00E-109	99.54
Bifidobacterium	Bifidobacterium	99%	6.00E-109	97.02
Bifidobacterium	Bifidobacterium	100%	6.00E-109	97.05
Bifidobacterium	Bifidobacterium	100%	2.00E-108	96.62
Bifidobacterium	Bifidobacterium	100%	2.00E-108	96.62
Bifidobacterium	Bifidobacterium	100%	2.00E-108	96.62
Bifidobacterium	Bifidobacterium	100%	2.00E-108	96.62
Bifidobacterium	Bifidobacterium	100%	2.00E-108	96.62
Bifidobacterium	Bifidobacterium	100%	2.00E-108	96.62
Bifidobacterium	Bifidobacterium	100%	2.00E-108	96.62
Bifidobacterium	Bifidobacterium	100%	2.00E-108	96.62
Uncultured Bific	uncultured Bifid	100%	2.00E-108	96.62
Bifidobacterium	Bifidobacterium	100%	2.00E-108	96.62
Bifidobacterium	Bifidobacterium	100%	2.00E-108	96.62
Bifidobacterium	Bifidobacterium	100%	2.00E-108	96.62
Bifidobacterium	Bifidobacterium	100%	2.00E-108	96.64
Bifidobacterium	Bifidobacterium	100%	2.00E-108	96.62
Bifidobacterium	Bifidobacterium	100%	2.00E-108	96.62
Bifidobacterium	Bifidobacterium	100%	2.00E-108	96.62
Uncultured Bific	uncultured Bifid	100%	2.00E-108	96.62
Bifidobacterium	Bifidobacterium	100%	2.00E-108	96.62
Bifidobacterium	Bifidobacterium	100%	2.00E-108	96.62
Bifidobacterium	Bifidobacterium	100%	2.00E-108	96.62
Bifidobacterium	Bifidobacterium	100%	2.00E-108	96.62
Bifidobacterium	Bifidobacterium	100%	2.00E-108	96.62
Bifidobacterium	Bifidobacterium	100%	2.00E-108	96.62
Bifidobacterium	Bifidobacterium	100%	2.00E-108	96.62
Bifidobacterium	Bifidobacterium	100%	2.00E-108	96.62
Bifidobacterium	Bifidobacterium	100%	2.00E-108	96.62
Bifidobacterium	Bifidobacterium	100%	2.00E-108	96.62
Bifidobacterium	Bifidobacterium	100%	2.00E-108	96.62
Bifidobacterium	Bifidobacterium	100%	2.00E-108	96.62

Bifidobacterium	Bifidobacterium	100%	2.00E-108	96.62
Bifidobacterium	Bifidobacterium	100%	2.00E-108	96.62
Bifidobacterium	Bifidobacterium	100%	2.00E-108	96.62
Bifidobacterium	Bifidobacterium	100%	2.00E-108	96.62
Bifidobacterium	Bifidobacterium	100%	2.00E-108	96.62
Bifidobacterium	Bifidobacterium	100%	2.00E-108	96.62
Bifidobacterium	Bifidobacterium	100%	2.00E-108	96.62
Bifidobacterium	Bifidobacterium	100%	2.00E-108	96.62
Bifidobacterium	Bifidobacterium	100%	2.00E-108	96.62
Bifidobacterium	Bifidobacterium	100%	2.00E-108	96.62
Bifidobacterium	Bifidobacterium	100%	2.00E-108	96.62
Bifidobacterium	Bifidobacterium	100%	2.00E-108	96.64
Bifidobacterium	Bifidobacterium	100%	2.00E-108	96.62
Bifidobacterium	Bifidobacterium	100%	2.00E-108	96.62
Bifidobacterium	Bifidobacterium	100%	2.00E-108	96.62
Bifidobacterium	Bifidobacterium	100%	2.00E-108	96.62
Bifidobacterium	Bifidobacterium	100%	2.00E-108	96.62
Bifidobacterium	Bifidobacterium	100%	2.00E-108	96.62
Bifidobacterium	Bifidobacterium	100%	2.00E-108	96.62
Bifidobacterium	Bifidobacterium	100%	2.00E-108	96.62
Bifidobacterium	Bifidobacterium	100%	2.00E-108	96.62
Uncultured Bific	uncultured Bifid	100%	2.00E-108	96.62
Uncultured Bific	uncultured Bifid	100%	2.00E-108	96.62
Bifidobacterium	Bifidobacterium	100%	2.00E-108	96.64
Bifidobacterium	Bifidobacterium	100%	2.00E-108	96.62
Bifidobacterium	Bifidobacterium	100%	2.00E-108	96.62
Bifidobacterium	Bifidobacterium	100%	2.00E-108	96.62
Uncultured Bific	uncultured Bifid	100%	2.00E-108	96.62
Bifidobacterium	Bifidobacterium	100%	2.00E-108	96.62
Uncultured Bific	uncultured Bifid	100%	2.00E-108	96.62
Bifidobacterium	Bifidobacterium	100%	2.00E-108	96.62
Bifidobacterium	Bifidobacterium	100%	2.00E-108	96.62
Bifidobacterium	Bifidobacterium	100%	2.00E-108	96.62
Bifidobacterium	Bifidobacterium	100%	2.00E-108	96.62
Bifidobacterium	Bifidobacterium	100%	2.00E-108	96.62
Bifidobacterium	Bifidobacterium	100%	2.00E-108	96.62
Bifidobacterium	Bifidobacterium	100%	2.00E-108	96.62
Bifidobacterium	Bifidobacterium	100%	2.00E-108	96.62
Bifidobacterium	Bifidobacterium	100%	2.00E-108	96.62
Bifidobacterium	Bifidobacterium	100%	2.00E-108	96.62
Bifidobacterium	Bifidobacterium	100%	2.00E-108	96.62
Bifidobacterium	Bifidobacterium	100%	2.00E-108	96.62
Bifidobacterium	Bifidobacterium	100%	2.00E-108	96.62
Bifidobacterium	Bifidobacterium	100%	2.00E-108	96.62

Bifidobacterium	Bifidobacterium	100%	2.00E-108	96.62
Bifidobacterium	Bifidobacterium	100%	2.00E-108	96.62
Bifidobacterium	Bifidobacterium	100%	2.00E-108	96.62
Bifidobacterium	Bifidobacterium	100%	2.00E-108	96.62
Bifidobacterium	Bifidobacterium	100%	2.00E-108	96.62
Bifidobacterium	Bifidobacterium	100%	2.00E-108	96.62
Bifidobacterium	Bifidobacterium	100%	2.00E-108	96.62
Bifidobacterium	Bifidobacterium	100%	2.00E-108	96.62
Bifidobacterium	Bifidobacterium	100%	2.00E-108	96.62
Bifidobacterium	Bifidobacterium	100%	2.00E-108	96.62
Bifidobacterium	Bifidobacterium	100%	2.00E-108	96.62
Bifidobacterium	Bifidobacterium	100%	2.00E-108	96.62
Bifidobacterium	Bifidobacterium	100%	2.00E-108	96.62
Bifidobacterium	Bifidobacterium	100%	2.00E-108	96.62
Bifidobacterium	Bifidobacterium	100%	2.00E-108	96.62
Bifidobacterium	Bifidobacterium	100%	2.00E-108	96.62
Bifidobacterium	Bifidobacterium	100%	2.00E-108	96.62
Bifidobacterium	Bifidobacterium	100%	2.00E-108	96.62
Bifidobacterium	Bifidobacterium	100%	2.00E-108	96.62
Bifidobacterium	Bifidobacterium	100%	2.00E-108	96.62
Bifidobacterium	Bifidobacterium	100%	2.00E-108	96.62
Bifidobacterium	Bifidobacterium	100%	2.00E-108	96.62
Bifidobacterium	Bifidobacterium	100%	2.00E-108	96.62
Bifidobacterium	Bifidobacterium	100%	2.00E-108	96.62
Bifidobacterium	Bifidobacterium	100%	2.00E-108	96.62
Bifidobacterium	Bifidobacterium	100%	2.00E-108	96.62
Bifidobacterium	Bifidobacterium	100%	2.00E-108	96.62
Bifidobacterium	Bifidobacterium	100%	2.00E-108	96.62
Bifidobacterium	Bifidobacterium	100%	2.00E-108	96.62
Bifidobacterium	Bifidobacterium	100%	2.00E-108	96.62
Bifidobacterium	Bifidobacterium	100%	2.00E-108	96.62
Bifidobacterium	Bifidobacterium	100%	2.00E-108	96.62
Bifidobacterium	Bifidobacterium	100%	2.00E-108	96.62
Bifidobacterium	Bifidobacterium	100%	2.00E-108	96.62
Bifidobacterium	Bifidobacterium	100%	2.00E-108	96.62
Bifidobacterium	Bifidobacterium	100%	2.00E-108	96.62
Bifidobacterium	Bifidobacterium	100%	2.00E-108	96.62
Bifidobacterium	Bifidobacterium	100%	2.00E-108	96.62
Bifidobacterium	Bifidobacterium	100%	2.00E-108	96.62
Bifidobacterium	Bifidobacterium	100%	2.00E-108	96.62
Uncultured Bific	uncultured Bifid	100%	2.00E-108	96.62
Uncultured Bific	uncultured Bifid	100%	2.00E-108	96.62
Uncultured Bific	uncultured Bifid	100%	2.00E-108	96.62
Uncultured Bific	uncultured Bifid	100%	2.00E-108	96.62

Bifidobacterium	Bifidobacterium	100%	2.00E-108	96.62
Bifidobacterium	Bifidobacterium	100%	2.00E-108	96.62
Bifidobacterium	Bifidobacterium	100%	2.00E-108	96.62
Bifidobacterium	Bifidobacterium	100%	2.00E-108	96.62
Bifidobacterium	Bifidobacterium	100%	2.00E-108	96.62
Bifidobacterium	Bifidobacterium	100%	2.00E-108	96.62
Bifidobacterium	Bifidobacterium	100%	2.00E-108	96.62
Bifidobacterium	Bifidobacterium	100%	2.00E-108	96.62
Bifidobacterium	Bifidobacterium	100%	2.00E-108	96.62
Bifidobacterium	Bifidobacterium	100%	2.00E-108	96.62
Bifidobacterium	Bifidobacterium	100%	2.00E-108	96.62
Bifidobacterium	Bifidobacterium	100%	2.00E-108	96.62
Bifidobacterium	Bifidobacterium	100%	2.00E-108	96.62
Bifidobacterium	Bifidobacterium	100%	2.00E-108	96.62
Bifidobacterium	Bifidobacterium	100%	2.00E-108	96.62
Bifidobacterium	Bifidobacterium	100%	2.00E-108	96.62
Bifidobacterium	Bifidobacterium	100%	2.00E-108	96.62
Bifidobacterium	Bifidobacterium	100%	2.00E-108	96.62
Bifidobacterium	Bifidobacterium	100%	2.00E-108	96.62
Bifidobacterium	Bifidobacterium	100%	2.00E-108	96.64
Bifidobacterium	Bifidobacterium	100%	2.00E-108	96.62
Bifidobacterium	Bifidobacterium	100%	2.00E-108	96.62
Bifidobacterium	Bifidobacterium	100%	2.00E-108	96.62
Bifidobacterium	Bifidobacterium	100%	2.00E-108	96.62
Bifidobacterium	Bifidobacterium	100%	2.00E-108	96.62
Bifidobacterium	Bifidobacterium	100%	2.00E-108	96.62
Bifidobacterium	Bifidobacterium	100%	2.00E-108	96.62
Bifidobacterium	Bifidobacterium	100%	2.00E-108	96.62
Bifidobacterium	Bifidobacterium	100%	2.00E-108	96.62
Bifidobacterium	Bifidobacterium	100%	2.00E-108	96.62
Bifidobacterium	Bifidobacterium	91%	2.00E-108	99.54
Bifidobacterium	Bifidobacterium	91%	2.00E-108	99.54
Bifidobacterium	Bifidobacterium	100%	2.00E-108	96.62
Bifidobacterium	Bifidobacterium	100%	2.00E-108	96.62
Bifidobacterium	Bifidobacterium	100%	2.00E-108	96.62
Bifidobacterium	Bifidobacterium	100%	2.00E-108	96.62
Bifidobacterium	Bifidobacterium	100%	2.00E-108	96.62
Bifidobacterium	Bifidobacterium	100%	2.00E-108	96.62
Bifidobacterium	Bifidobacterium	100%	2.00E-108	96.62
Bifidobacterium	Bifidobacterium	100%	2.00E-108	96.62
Bifidobacterium	Bifidobacterium	100%	2.00E-108	96.62
Bifidobacterium	Bifidobacterium	100%	2.00E-108	96.62
Bifidobacterium	Bifidobacterium	100%	2.00E-108	96.62
Bifidobacterium	Bifidobacterium	100%	2.00E-108	96.62

Bifidobacterium	Bifidobacterium	100%	2.00E-108	96.62
Bifidobacterium	Bifidobacterium	100%	2.00E-108	96.62
Bifidobacterium	Bifidobacterium	100%	2.00E-108	96.62
Bifidobacterium	Bifidobacterium	100%	2.00E-108	96.64
Bifidobacterium	Bifidobacterium	100%	2.00E-108	96.64
Bifidobacterium	Bifidobacterium	100%	2.00E-108	96.62
Bifidobacterium	Bifidobacterium	100%	2.00E-108	96.62
Bifidobacterium	Bifidobacterium	100%	2.00E-108	96.62
Bifidobacterium	Bifidobacterium	100%	2.00E-108	96.62
Bifidobacterium	Bifidobacterium	100%	2.00E-108	96.62
Bifidobacterium	Bifidobacterium	100%	2.00E-108	96.62
Bifidobacterium	Bifidobacterium	100%	2.00E-108	96.62
Bifidobacterium	Bifidobacterium	100%	2.00E-108	96.62
Bifidobacterium	Bifidobacterium	100%	2.00E-108	96.62
Bifidobacterium	Bifidobacterium	100%	2.00E-108	96.62
Bifidobacterium	Bifidobacterium	100%	2.00E-108	96.62
Bifidobacterium	Bifidobacterium	100%	2.00E-108	96.62
Bifidobacterium	Bifidobacterium	100%	2.00E-108	96.62
Bifidobacterium	Bifidobacterium	100%	2.00E-108	96.62
Bifidobacterium	Bifidobacterium	100%	2.00E-108	96.62
Uncultured Bific	uncultured Bifid	100%	2.00E-108	96.62
Uncultured Bific	uncultured Bifid	100%	2.00E-108	96.62
Uncultured Bific	uncultured Bifid	100%	2.00E-108	96.62
Uncultured Bific	uncultured Bifid	100%	2.00E-108	96.62
Uncultured Bific	uncultured Bifid	100%	2.00E-108	96.62
Uncultured Bific	uncultured Bifid	100%	2.00E-108	96.62
Uncultured Bific	uncultured Bifid	100%	2.00E-108	96.62
Uncultured Bific	uncultured Bifid	100%	2.00E-108	96.62
Uncultured Bific	uncultured Bifid	100%	2.00E-108	96.62
Uncultured Bific	uncultured Bifid	100%	2.00E-108	96.62
Uncultured Bific	uncultured Bifid	100%	2.00E-108	96.62
Uncultured Bific	uncultured Bifid	100%	2.00E-108	96.62
Uncultured Bific	uncultured Bifid	100%	2.00E-108	96.62
Uncultured Bific	uncultured Bifid	100%	2.00E-108	96.62
Uncultured Bific	uncultured Bifid	100%	2.00E-108	96.62
Uncultured Bific	uncultured Bifid	100%	2.00E-108	96.62
Uncultured Bific	uncultured Bifid	100%	2.00E-108	96.62
Uncultured Bific	uncultured Bifid	100%	2.00E-108	96.62
Uncultured Bific	uncultured Bifid	100%	2.00E-108	96.62
Uncultured Bific	uncultured Bifid	100%	2.00E-108	96.62
Uncultured Bific	uncultured Bifid	100%	2.00E-108	96.62
Uncultured Bific	uncultured Bifid	100%	2.00E-108	96.62
Uncultured Bific	uncultured Bifid	100%	2.00E-108	96.62
Bifidobacterium	Bifidobacterium	100%	2.00E-108	96.62

Bifidobacterium	Bifidobacterium	92%	2.00E-108	99.09
Uncultured Bific	uncultured Bifid	100%	2.00E-108	96.62
Uncultured Bific	uncultured Bifid	100%	2.00E-108	96.62
Bifidobacterium	Bifidobacterium	100%	2.00E-108	96.62
Bifidobacterium	Bifidobacterium	100%	2.00E-108	96.62
Bifidobacterium	Bifidobacterium	100%	2.00E-108	96.62
Bifidobacterium	Bifidobacterium	100%	2.00E-108	96.62
Bifidobacterium	Bifidobacterium	100%	2.00E-108	96.62
Bifidobacterium	Bifidobacterium	100%	2.00E-108	96.62
Bifidobacterium	Bifidobacterium	100%	2.00E-108	96.62
Bifidobacterium	Bifidobacterium	100%	2.00E-108	96.62
Bifidobacterium	Bifidobacterium	100%	2.00E-108	96.62
Bifidobacterium	Bifidobacterium	100%	2.00E-108	96.62
Bifidobacterium	Bifidobacterium	100%	2.00E-108	96.62
Bifidobacterium	Bifidobacterium	100%	2.00E-108	96.62
Bifidobacterium	Bifidobacterium	99%	2.00E-108	97.02
Bifidobacterium	Bifidobacterium	100%	2.00E-108	96.62
Bifidobacterium	Bifidobacterium	100%	2.00E-108	96.62
Bifidobacterium	Bifidobacterium	100%	2.00E-108	96.62
Uncultured Bific	uncultured Bifid	97%	7.00E-108	97.39
Uncultured Bific	uncultured Bifid	99%	7.00E-108	96.61
Bifidobacterium	Bifidobacterium	89%	7.00E-108	100
Bifidobacterium	Bifidobacterium	98%	7.00E-108	97.01
Bifidobacterium	Bifidobacterium	100%	7.00E-108	95.78
Bifidobacterium	Bifidobacterium	100%	7.00E-108	95.78
Bifidobacterium	Bifidobacterium	100%	3.00E-107	96.2
Bifidobacterium	Bifidobacterium	99%	3.00E-107	96.6
Bifidobacterium	Bifidobacterium	100%	3.00E-107	96.2
Uncultured Bific	uncultured Bifid	91%	3.00E-107	98.62
Bifidobacterium	Bifidobacterium	99%	3.00E-107	96.6
Bifidobacterium	Bifidobacterium	99%	3.00E-107	96.6
Bifidobacterium	Bifidobacterium	99%	3.00E-107	96.6
Bifidobacterium	Bifidobacterium	99%	3.00E-107	96.6
Bifidobacterium	Bifidobacterium	100%	3.00E-107	96.2
Bifidobacterium	Bifidobacterium	90%	3.00E-107	99.53
Uncultured Bific	uncultured Bifid	99%	3.00E-107	96.6
Uncultured Bific	uncultured Bifid	99%	3.00E-107	96.6
Bifidobacterium	Bifidobacterium	89%	3.00E-107	100
Bifidobacterium	Bifidobacterium	89%	3.00E-107	100
Bifidobacterium	Bifidobacterium	91%	3.00E-107	99.08
Bifidobacterium	Bifidobacterium	100%	1.00E-106	96.2
Bifidobacterium	Bifidobacterium	100%	1.00E-106	96.2
Bifidobacterium	Bifidobacterium	100%	1.00E-106	96.2
Bifidobacterium	Bifidobacterium	100%	1.00E-106	96.2

Bifidobacterium	Bifidobacterium	100%	1.00E-106	96.2
Bifidobacterium	Bifidobacterium	100%	1.00E-106	96.2
Uncultured Bific	uncultured Bifid	100%	1.00E-106	96.2
Bifidobacterium	Bifidobacterium	100%	1.00E-106	96.22
Bifidobacterium	Bifidobacterium	100%	1.00E-106	96.22
Bifidobacterium	Bifidobacterium	100%	1.00E-106	96.2
Bifidobacterium	Bifidobacterium	100%	1.00E-106	96.2
Uncultured Bific	uncultured Bifid	100%	1.00E-106	96.2
Bifidobacterium	Bifidobacterium	99%	1.00E-106	97.02
Uncultured Bific	uncultured Bifid	100%	1.00E-106	96.2
Bifidobacterium	Bifidobacterium	100%	1.00E-106	96.2
Bifidobacterium	Bifidobacterium	100%	1.00E-106	96.2
Bifidobacterium	Bifidobacterium	100%	1.00E-106	96.2
Bifidobacterium	Bifidobacterium	100%	1.00E-106	96.2
Bifidobacterium	Bifidobacterium	100%	1.00E-106	96.2
Bifidobacterium	Bifidobacterium	100%	1.00E-106	96.2
Bifidobacterium	Bifidobacterium	100%	1.00E-106	96.2
Bifidobacterium	Bifidobacterium	100%	1.00E-106	96.2
Bifidobacterium	Bifidobacterium	100%	1.00E-106	96.2
Uncultured Bific	uncultured Bifid	100%	1.00E-106	96.2
Uncultured Bific	uncultured Bifid	89%	1.00E-106	99.53
Uncultured Bific	uncultured Bifid	98%	1.00E-106	96.58
Bifidobacterium	Bifidobacterium	97%	1.00E-106	96.55
Uncultured Bific	uncultured Bifid	100%	1.00E-106	96.2
Bifidobacterium	Bifidobacterium	100%	1.00E-106	96.2
Bifidobacterium	Bifidobacterium	100%	1.00E-106	96.2
Bifidobacterium	Bifidobacterium	100%	1.00E-106	96.2
Bifidobacterium	Bifidobacterium	100%	1.00E-106	96.2
Uncultured Bific	uncultured Bifid	100%	1.00E-106	96.2
Uncultured Bific	uncultured Bifid	100%	1.00E-106	96.22
Uncultured Bific	uncultured Bifid	100%	1.00E-106	96.22
Uncultured Bific	uncultured Bifid	100%	1.00E-106	96.22
Uncultured Bific	uncultured Bifid	100%	1.00E-106	96.22
Uncultured Bific	uncultured Bifid	100%	1.00E-106	96.2
Bifidobacterium	Bifidobacterium	100%	1.00E-106	96.2
Uncultured Bific	uncultured Bifid	100%	1.00E-106	96.2
Uncultured Bific	uncultured Bifid	100%	1.00E-106	96.2
Uncultured Bific	uncultured Bifid	100%	1.00E-106	96.2
Uncultured Bific	uncultured Bifid	100%	1.00E-106	96.2
Bifidobacterium	Bifidobacterium	100%	1.00E-106	96.2
B.bifidum small	Bifidobacterium	97%	1.00E-106	96.55
Bifidobacterium	Bifidobacterium	100%	3.00E-106	95.87
Bifidobacterium	Bifidobacterium	97%	3.00E-106	95.26
Bifidobacterium	Bifidobacterium	100%	3.00E-106	95.36

Bifidobacterium	Bifidobacterium	100%	3.00E-106	95.36
Uncultured Bific	uncultured Bifid	100%	3.00E-106	96.2
Bifidobacterium	Bifidobacterium	100%	3.00E-106	96.2
Bifidobacterium	Bifidobacterium	95%	3.00E-106	97.36
Uncultured Bific	uncultured Bifid	100%	3.00E-106	96.2
Uncultured Bific	uncultured Bifid	100%	3.00E-106	96.2
Bifidobacterium	Bifidobacterium	88%	3.00E-106	100
Bifidobacterium	Bifidobacterium	100%	1.00E-105	95.83
Bifidobacterium	Bifidobacterium	99%	1.00E-105	96.17
Bifidobacterium	Bifidobacterium	99%	1.00E-105	96.17
Bifidobacterium	Bifidobacterium	99%	1.00E-105	96.17
Bifidobacterium	Bifidobacterium	99%	1.00E-105	96.17
Bifidobacterium	Bifidobacterium	99%	1.00E-105	96.17
Bifidobacterium	Bifidobacterium	99%	1.00E-105	96.17
Bifidobacterium	Bifidobacterium	99%	1.00E-105	96.17
Bifidobacterium	Bifidobacterium	100%	1.00E-105	95.78
Bifidobacterium	Bifidobacterium	100%	1.00E-105	95.82
Bifidobacterium	Bifidobacterium	87%	1.00E-105	100
Uncultured Bific	uncultured Bifid	99%	1.00E-105	96.2
Bifidobacterium	Bifidobacterium	89%	1.00E-105	99.53
Bifidobacterium	Bifidobacterium	89%	1.00E-105	99.53
Bifidobacterium	Bifidobacterium	100%	1.00E-105	95.82
Bifidobacterium	Bifidobacterium	100%	4.00E-105	95.78
Bifidobacterium	Bifidobacterium	90%	4.00E-105	99.07
Bifidobacterium	Bifidobacterium	100%	4.00E-105	95.78
Bifidobacterium	Bifidobacterium	100%	4.00E-105	95.78
Bifidobacterium	Bifidobacterium	100%	4.00E-105	95.78
Bifidobacterium	Bifidobacterium	100%	4.00E-105	95.78
Bifidobacterium	Bifidobacterium	100%	4.00E-105	95.78
Bifidobacterium	Bifidobacterium	100%	4.00E-105	95.78
Bifidobacterium	Bifidobacterium	100%	4.00E-105	95.78
Bifidobacterium	Bifidobacterium	100%	4.00E-105	95.78
Bifidobacterium	Bifidobacterium	100%	4.00E-105	95.18
Bifidobacterium	Bifidobacterium	100%	4.00E-105	95.8
Bifidobacterium	Bifidobacterium	100%	4.00E-105	95.78
Bifidobacterium	Bifidobacterium	100%	4.00E-105	95.78
Bifidobacterium	Bifidobacterium	90%	4.00E-105	98.62
Bifidobacterium	Bifidobacterium	89%	4.00E-105	99.06
Uncultured Bific	uncultured Bifid	98%	4.00E-105	96.15
Uncultured Bific	uncultured Bifid	100%	4.00E-105	95.78
Uncultured Bific	uncultured Bifid	100%	4.00E-105	95.78
Uncultured Bific	uncultured Bifid	100%	4.00E-105	95.78
Uncultured Bific	uncultured Bifid	100%	4.00E-105	95.78
Bifidobacterium	Bifidobacterium	100%	4.00E-105	95.78

Uncultured Bific	uncultured Bifid	100%	4.00E-105	95.78
Bifidobacterium	Bifidobacterium	99%	2.00E-104	96.17
Bifidobacterium	Bifidobacterium	100%	2.00E-104	95.44
Bifidobacterium	Bifidobacterium	100%	2.00E-104	95.44
Bifidobacterium	Bifidobacterium	100%	2.00E-104	95.45
Bifidobacterium	Bifidobacterium	100%	2.00E-104	95.8
Bifidobacterium	Bifidobacterium	89%	2.00E-104	99.06
Bifidobacterium	Bifidobacterium	89%	2.00E-104	99.06
Bifidobacterium	Bifidobacterium	86%	2.00E-104	100
Bifidobacterium	Bifidobacterium	86%	2.00E-104	100
Bifidobacterium	Bifidobacterium	99%	6.00E-104	95.74
Bifidobacterium	Bifidobacterium	99%	6.00E-104	95.74
Bifidobacterium	Bifidobacterium	100%	6.00E-104	95.42
Bifidobacterium	Bifidobacterium	99%	6.00E-104	95.74
Bifidobacterium	Bifidobacterium	99%	6.00E-104	95.74
Bifidobacterium	Bifidobacterium	99%	6.00E-104	95.74
Bifidobacterium	Bifidobacterium	99%	6.00E-104	95.74
Bifidobacterium	Bifidobacterium	99%	6.00E-104	95.74
Bifidobacterium	Bifidobacterium	99%	6.00E-104	95.74
Bifidobacterium	Bifidobacterium	99%	6.00E-104	95.74
Bifidobacterium	Bifidobacterium	99%	6.00E-104	95.74
Bifidobacterium	Bifidobacterium	99%	6.00E-104	95.74
Bifidobacterium	Bifidobacterium	99%	6.00E-104	95.74
Bifidobacterium	Bifidobacterium	99%	6.00E-104	95.74
Bifidobacterium	Bifidobacterium	99%	6.00E-104	95.74
Bifidobacterium	Bifidobacterium	99%	6.00E-104	95.74
Bifidobacterium	Bifidobacterium	99%	6.00E-104	95.74
Bifidobacterium	Bifidobacterium	100%	6.00E-104	95.4
Bifidobacterium	Bifidobacterium	100%	6.00E-104	95.42
Bifidobacterium	Bifidobacterium	87%	6.00E-104	99.52
Bifidobacterium	Bifidobacterium	86%	6.00E-104	100
Bifidobacterium	Bifidobacterium	86%	6.00E-104	100
Bifidobacterium	Bifidobacterium	90%	6.00E-104	98.17
Bifidobacterium	Bifidobacterium	100%	6.00E-104	95.78
Bifidobacterium	Bifidobacterium	91%	6.00E-104	98.17
Bifidobacterium	Bifidobacterium	90%	2.00E-103	98.6
Bifidobacterium	Bifidobacterium	100%	2.00E-103	95.78
Bifidobacterium	Bifidobacterium	99%	2.00E-103	95.32
Bifidobacterium	Bifidobacterium	100%	2.00E-103	95.36
Uncultured Bific	uncultured Bifid	98%	2.00E-103	95.73
Bifidobacterium	Bifidobacterium	100%	2.00E-103	95.36
Bifidobacterium	Bifidobacterium	100%	2.00E-103	94.51
Uncultured Bific	uncultured Bifid	100%	2.00E-103	95.36
Bifidobacterium	Bifidobacterium	87%	2.00E-103	99.52

Bifidobacterium	Bifidobacterium	86%	2.00E-103	100
Bifidobacterium	Bifidobacterium	86%	2.00E-103	100
Uncultured Bific	uncultured Bifid	100%	2.00E-103	95.36
Bifidobacterium	Bifidobacterium	100%	2.00E-103	95.36
Bifidobacterium	Bifidobacterium	100%	2.00E-103	93.67
Bifidobacterium	Bifidobacterium	100%	2.00E-103	94.51
Bifidobacterium	Bifidobacterium	86%	7.00E-103	99.51
Bifidobacterium	Bifidobacterium	85%	7.00E-103	100
Bifidobacterium	Bifidobacterium	99%	3.00E-102	95.32
Bifidobacterium	Bifidobacterium	99%	3.00E-102	95.32
Bifidobacterium	Bifidobacterium	99%	3.00E-102	95.32
Bifidobacterium	Bifidobacterium	99%	3.00E-102	95.32
Bifidobacterium	Bifidobacterium	99%	3.00E-102	94.89
Bifidobacterium	Bifidobacterium	99%	3.00E-102	95.32
Bifidobacterium	Bifidobacterium	99%	3.00E-102	95.32
Bifidobacterium	Bifidobacterium	99%	3.00E-102	95.32
Bifidobacterium	Bifidobacterium	99%	3.00E-102	95.32
Uncultured Bific	uncultured Bifid	99%	3.00E-102	95.32
Uncultured Bific	uncultured Bifid	99%	3.00E-102	95.32
Uncultured Bific	uncultured Bifid	99%	3.00E-102	95.32
Bifidobacterium	Bifidobacterium	86%	3.00E-102	99.51
Bifidobacterium	Bifidobacterium	85%	3.00E-102	100
Bifidobacterium	Bifidobacterium	99%	3.00E-102	95.32
Bifidobacterium	Bifidobacterium	99%	3.00E-102	95.32
Bifidobacterium	Bifidobacterium	100%	1.00E-101	94.94
Bifidobacterium	Bifidobacterium	90%	1.00E-101	98.13
Bifidobacterium	Bifidobacterium	90%	1.00E-101	98.13
Bifidobacterium	Bifidobacterium	90%	1.00E-101	98.13
Bifidobacterium	Bifidobacterium	100%	1.00E-101	94.94
Bifidobacterium	Bifidobacterium	100%	1.00E-101	94.94
Bifidobacterium	Bifidobacterium	100%	1.00E-101	94.94
Uncultured Bific	uncultured Bifid	100%	1.00E-101	94.94
Bifidobacterium	Bifidobacterium	99%	1.00E-101	94.69
Bifidobacterium	Bifidobacterium	84%	1.00E-101	100
Bifidobacterium	Bifidobacterium	84%	1.00E-101	100
Bifidobacterium	Bifidobacterium	84%	1.00E-101	100
Uncultured Bific	uncultured Bifid	100%	1.00E-101	94.94
Bifidobacterium	Bifidobacterium	100%	1.00E-101	94.94
Uncultured Bific	uncultured Bifid	100%	1.00E-101	93.83
Bifidobacterium	Bifidobacterium	100%	1.00E-101	93.67
Uncultured Bific	uncultured Bifid	98%	3.00E-101	95.3
Uncultured Bific	uncultured Bifid	98%	3.00E-101	94.96
Bifidobacterium	Bifidobacterium	85%	3.00E-101	99.51
Uncultured Bific	uncultured Bifid	99%	1.00E-100	94.92

Uncultured Bific	uncultured Bifid	99%	1.00E-100	94.89
Bifidobacterium	Bifidobacterium	97%	1.00E-100	95.26
Uncultured Bific	uncultured Bifid	99%	1.00E-100	94.89
Bifidobacterium	Bifidobacterium	100%	1.00E-100	94.51
Bifidobacterium	Bifidobacterium	99%	1.00E-100	94.89
Bifidobacterium	Bifidobacterium	99%	1.00E-100	94.89
Bifidobacterium	Bifidobacterium	90%	5.00E-100	97.66
Bifidobacterium	Bifidobacterium	90%	5.00E-100	97.66
Bifidobacterium	Bifidobacterium	90%	5.00E-100	97.66
Bifidobacterium	Bifidobacterium	90%	5.00E-100	97.66
Bifidobacterium	Bifidobacterium	90%	5.00E-100	97.66
Bifidobacterium	Bifidobacterium	90%	5.00E-100	97.66
Bifidobacterium	Bifidobacterium	90%	5.00E-100	97.66
Bifidobacterium	Bifidobacterium	90%	5.00E-100	97.66
Bifidobacterium	Bifidobacterium	100%	5.00E-100	94.51
Bifidobacterium	Bifidobacterium	90%	5.00E-100	97.66
Uncultured Bific	uncultured Bifid	100%	5.00E-100	94.51
Bifidobacterium	Bifidobacterium	100%	5.00E-100	94.56
Bifidobacterium	Bifidobacterium	100%	5.00E-100	94.51
Bifidobacterium	Bifidobacterium	99%	5.00E-100	94.04
Bifidobacterium	Bifidobacterium	100%	5.00E-100	94.51
Uncultured Bific	uncultured Bifid	100%	5.00E-100	94.51
Bifidobacterium	Bifidobacterium	91%	5.00E-100	97.22
Bifidobacterium	Bifidobacterium	100%	5.00E-100	94.51
Bifidobacterium	Bifidobacterium	100%	5.00E-100	94.51
Bifidobacterium	Bifidobacterium	100%	5.00E-100	94.51
Bifidobacterium	Bifidobacterium	100%	5.00E-100	94.51
Bifidobacterium	Bifidobacterium	100%	5.00E-100	94.51
Bifidobacterium	Bifidobacterium	84%	5.00E-100	99.5
Bifidobacterium	Bifidobacterium	89%	5.00E-100	97.65
Uncultured Bific	uncultured Bifid	100%	5.00E-100	94.51
Uncultured Bific	uncultured Bifid	100%	5.00E-100	94.51
Uncultured Bific	uncultured Bifid	100%	5.00E-100	94.54
Bifidobacterium	Bifidobacterium	100%	5.00E-100	94.51
Bifidobacterium	Bifidobacterium	100%	5.00E-100	94.51
Bifidobacterium	Bifidobacterium	100%	5.00E-100	94.51
Bifidobacterium	Bifidobacterium	100%	5.00E-100	94.51
Bifidobacterium	Bifidobacterium	100%	5.00E-100	94.51
Bifidobacterium	Bifidobacterium	100%	5.00E-100	94.51
Bifidobacterium	Bifidobacterium	89%	2.00E-99	97.65
Bifidobacterium	Bifidobacterium	88%	2.00E-99	98.09
Bifidobacterium	Bifidobacterium	84%	2.00E-99	99.5
Bifidobacterium	Bifidobacterium	84%	2.00E-99	99.5
Uncultured Bific	uncultured Bifid	99%	2.00E-99	94.49

Uncultured Bific	uncultured Bifid	97%	2.00E-99	95.22
Bifidobacterium	Bifidobacterium	91%	6.00E-99	96.77
Bifidobacterium	Bifidobacterium	100%	6.00E-99	93.93
Bifidobacterium	Bifidobacterium	100%	6.00E-99	93.93
Bifidobacterium	Bifidobacterium	82%	6.00E-99	100
Bifidobacterium	Bifidobacterium	82%	6.00E-99	100
Bifidobacterium	Bifidobacterium	85%	6.00E-99	99.01
Bifidobacterium	Bifidobacterium	90%	2.00E-98	97.2
Bifidobacterium	Bifidobacterium	87%	2.00E-98	98.07
Uncultured Bific	uncultured Bifid	98%	2.00E-98	94.47
Bifidobacterium	Bifidobacterium	100%	2.00E-98	94.12
Uncultured Bific	uncultured Bifid	89%	2.00E-98	97.2
Bifidobacterium	Bifidobacterium	82%	2.00E-98	100
Bifidobacterium	Bifidobacterium	82%	2.00E-98	100
Uncultured Bific	uncultured Bifid	100%	2.00E-98	94.09
Uncultured Bific	uncultured Bifid	100%	2.00E-98	94.12
Bifidobacterium	Bifidobacterium	100%	2.00E-98	94.09
Bifidobacterium	Bifidobacterium	100%	2.00E-98	94.09
Bifidobacterium	Bifidobacterium	100%	2.00E-98	94.09
Bifidobacterium	Bifidobacterium	88%	8.00E-98	97.62
Bifidobacterium	Bifidobacterium	83%	8.00E-98	99.49
Bifidobacterium	Bifidobacterium	83%	8.00E-98	99.49
Bifidobacterium	Bifidobacterium	84%	8.00E-98	99
Bifidobacterium	Bifidobacterium	85%	8.00E-98	98.52
Uncultured Bific	uncultured Bifid	99%	3.00E-97	94.07
Bifidobacterium	Bifidobacterium	99%	3.00E-97	94.07
Uncultured Bific	uncultured Bifid	99%	3.00E-97	94.07
Bifidobacterium	Bifidobacterium	100%	3.00E-97	93.5
Bifidobacterium	Bifidobacterium	82%	3.00E-97	99.49
Bifidobacterium	Bifidobacterium	82%	3.00E-97	99.49
Bifidobacterium	Bifidobacterium	82%	3.00E-97	99.49
Bifidobacterium	Bifidobacterium	90%	3.00E-97	96.74
Bifidobacterium	Bifidobacterium	99%	3.00E-97	93.62
Bifidobacterium	Bifidobacterium	81%	3.00E-97	100
Bifidobacterium	Bifidobacterium	86%	3.00E-97	98.05
Bifidobacterium	Bifidobacterium	85%	3.00E-97	98.51
Bifidobacterium	Bifidobacterium	81%	1.00E-96	100
Bifidobacterium	Bifidobacterium	100%	1.00E-96	93.67
Uncultured Bific	uncultured Bifid	100%	1.00E-96	93.67
Bifidobacterium	Bifidobacterium	82%	1.00E-96	99.49
Bifidobacterium	Bifidobacterium	88%	1.00E-96	97.14
Bifidobacterium	Bifidobacterium	82%	1.00E-96	99.49
Bifidobacterium	Bifidobacterium	82%	1.00E-96	99.49
Bifidobacterium	Bifidobacterium	97%	1.00E-96	93.64

Bifidobacterium	Bifidobacterium	100%	1.00E-96	93.67
Bifidobacterium	Bifidobacterium	84%	1.00E-96	98.51
Bifidobacterium	Bifidobacterium	84%	1.00E-96	98.51
Bifidobacterium	Bifidobacterium	88%	1.00E-96	97.14
Uncultured Bifid	uncultured Bifid	100%	1.00E-96	93.67
Bifidobacterium	Bifidobacterium	100%	1.00E-96	93.67
Bifidobacterium	Bifidobacterium	90%	4.00E-96	96.28
Bifidobacterium	Bifidobacterium	81%	4.00E-96	99.48
Uncultured Bifid	uncultured Bifid	98%	4.00E-96	94.02
Uncultured Bifid	uncultured Bifid	98%	4.00E-96	93.99
Uncultured Bifid	uncultured Bifid	83%	4.00E-96	98.99
Bifidobacterium	Bifidobacterium	81%	4.00E-96	99.48
Bifidobacterium	Bifidobacterium	86%	4.00E-96	97.57
Bifidobacterium	Bifidobacterium	81%	4.00E-96	99.48
Bifidobacterium	Bifidobacterium	80%	4.00E-96	100
Bifidobacterium	Bifidobacterium	99%	1.00E-95	93.62
Uncultured Bifid	uncultured Bifid	81%	1.00E-95	99.48
Bifidobacterium	Bifidobacterium	99%	1.00E-95	93.62
Uncultured Bifid	uncultured Bifid	100%	1.00E-95	93.06
Bifidobacterium	Bifidobacterium	81%	1.00E-95	99.48
Bifidobacterium	Bifidobacterium	86%	1.00E-95	97.57
Bifidobacterium	Bifidobacterium	87%	1.00E-95	97.12
Bifidobacterium	Bifidobacterium	86%	1.00E-95	97.56
Uncultured Bifid	uncultured Bifid	99%	1.00E-95	93.64
Bifidobacterium	Bifidobacterium	86%	5.00E-95	97.12
Uncultured Bifid	uncultured Bifid	98%	5.00E-95	93.59
Bifidobacterium	Bifidobacterium	83%	5.00E-95	98.48
Bifidobacterium	Bifidobacterium	83%	5.00E-95	98.48
Bifidobacterium	Bifidobacterium	84%	5.00E-95	98.01
Uncultured Bifid	uncultured Bifid	100%	5.00E-95	93.28
Bifidobacterium	Bifidobacterium	80%	2.00E-94	99.48
Bifidobacterium	Bifidobacterium	80%	2.00E-94	99.48
Bifidobacterium	Bifidobacterium	85%	2.00E-94	97.54
Uncultured Bifid	uncultured Bifid	83%	6.00E-94	97.99
Bifidobacterium	Bifidobacterium	80%	6.00E-94	99.48
Bifidobacterium	Bifidobacterium	99%	2.00E-93	94.04
Uncultured Bifid	uncultured Bifid	100%	2.00E-93	92.86
Bifidobacterium	Bifidobacterium	84%	2.00E-93	97.51
Bifidobacterium	Bifidobacterium	86%	2.00E-93	97.07
Bifidobacterium	Bifidobacterium	99%	8.00E-93	92.8
Bifidobacterium	Bifidobacterium	99%	8.00E-93	93.62
Bifidobacterium	Bifidobacterium	99%	8.00E-93	93.62
Bifidobacterium	Bifidobacterium	79%	8.00E-93	99.47
Bifidobacterium	Bifidobacterium	81%	8.00E-93	98.45

Bifidobacterium	Bifidobacterium	84%	8.00E-93	97.5
Bifidobacterium	Bifidobacterium	80%	8.00E-93	98.95
Uncultured Bific	uncultured Bifid	99%	3.00E-92	92.83
Bifidobacterium	Bifidobacterium	78%	3.00E-92	99.47
Bifidobacterium	Bifidobacterium	77%	1.00E-91	100
Bifidobacterium	Bifidobacterium	92%	1.00E-91	94.52
Bifidobacterium	Bifidobacterium	94%	1.00E-91	94.17
Uncultured Bific	uncultured Bifid	98%	1.00E-91	92.77
Uncultured Bific	uncultured Bifid	100%	1.00E-91	92.41
Bifidobacterium	Bifidobacterium	83%	1.00E-91	97.47
Uncultured Bific	uncultured Bifid	100%	1.00E-91	92.44
Uncultured Bific	uncultured Bifid	76%	4.00E-91	100
Uncultured Bific	uncultured Bifid	76%	4.00E-91	100
Uncultured Bific	uncultured Bifid	76%	4.00E-91	100
Uncultured Bific	uncultured Bifid	76%	4.00E-91	100
Uncultured Bific	uncultured Bifid	76%	4.00E-91	100
Uncultured Bific	uncultured Bifid	76%	4.00E-91	100
Uncultured Bific	uncultured Bifid	76%	4.00E-91	100
Uncultured Bific	uncultured Bifid	76%	4.00E-91	100
Uncultured Bific	uncultured Bifid	76%	4.00E-91	100
Uncultured Bific	uncultured Bifid	76%	4.00E-91	100
Uncultured Bific	uncultured Bifid	76%	4.00E-91	100
Uncultured Bific	uncultured Bifid	76%	4.00E-91	100
Uncultured Bific	uncultured Bifid	76%	4.00E-91	100
Uncultured Bific	uncultured Bifid	76%	4.00E-91	100
Uncultured Bific	uncultured Bifid	76%	4.00E-91	100
Uncultured Bific	uncultured Bifid	76%	4.00E-91	100
Uncultured Bific	uncultured Bifid	76%	4.00E-91	100
Uncultured Bific	uncultured Bifid	76%	4.00E-91	100
Uncultured Bific	uncultured Bifid	76%	4.00E-91	100
Uncultured Bific	uncultured Bifid	76%	4.00E-91	100
Uncultured Bific	uncultured Bifid	76%	4.00E-91	100
Uncultured Bific	uncultured Bifid	76%	4.00E-91	100
Uncultured Bific	uncultured Bifid	76%	4.00E-91	100
Uncultured Bific	uncultured Bifid	76%	4.00E-91	100
Uncultured Bific	uncultured Bifid	76%	4.00E-91	100
Uncultured Bific	uncultured Bifid	76%	4.00E-91	100
Uncultured Bific	uncultured Bifid	76%	4.00E-91	100
Uncultured Bific	uncultured Bifid	76%	4.00E-91	100
Uncultured Bific	uncultured Bifid	76%	4.00E-91	100
Uncultured Bific	uncultured Bifid	76%	4.00E-91	100
Uncultured Bific	uncultured Bifid	76%	4.00E-91	100
Uncultured Bific	uncultured Bifid	76%	4.00E-91	100

Uncultured Bifid	uncultured Bifid	76%	4.00E-91	100
Uncultured Bifid	uncultured Bifid	76%	4.00E-91	100
Uncultured Bifid	uncultured Bifid	76%	4.00E-91	100
Uncultured Bifid	uncultured Bifid	76%	4.00E-91	100
Uncultured Bifid	uncultured Bifid	76%	4.00E-91	100
Uncultured Bifid	uncultured Bifid	76%	4.00E-91	100
Uncultured Bifid	uncultured Bifid	76%	4.00E-91	100
Uncultured Bifid	uncultured Bifid	76%	4.00E-91	100
Uncultured Bifid	uncultured Bifid	76%	4.00E-91	100
Uncultured Bifid	uncultured Bifid	76%	4.00E-91	100
Bifidobacterium	Bifidobacterium	78%	4.00E-91	99.46
Bifidobacterium	Bifidobacterium	80%	4.00E-91	98.43
Bifidobacterium	Bifidobacterium	76%	1.00E-90	100
Bifidobacterium	Bifidobacterium	76%	1.00E-90	100
Bifidobacterium	Bifidobacterium	76%	1.00E-90	100
Bifidobacterium	Bifidobacterium	76%	1.00E-90	100
Bifidobacterium	Bifidobacterium	76%	1.00E-90	100
Bifidobacterium	Bifidobacterium	76%	1.00E-90	100
Bifidobacterium	Bifidobacterium	76%	1.00E-90	100
Bifidobacterium	Bifidobacterium	76%	1.00E-90	100
Bifidobacterium	Bifidobacterium	76%	1.00E-90	100
Bifidobacterium	Bifidobacterium	76%	1.00E-90	100
Bifidobacterium	Bifidobacterium	76%	1.00E-90	100
Uncultured Bifid	uncultured Bifid	99%	1.00E-90	93.19
Bifidobacterium	Bifidobacterium	75%	5.00E-90	100
Bifidobacterium	Bifidobacterium	75%	5.00E-90	100
Bifidobacterium	Bifidobacterium	75%	5.00E-90	100
Bifidobacterium	Bifidobacterium	75%	5.00E-90	100
Bifidobacterium	Bifidobacterium	75%	5.00E-90	100
Bifidobacterium	Bifidobacterium	75%	5.00E-90	100
Bifidobacterium	Bifidobacterium	75%	5.00E-90	100
Bifidobacterium	Bifidobacterium	75%	5.00E-90	100
Bifidobacterium	Bifidobacterium	75%	5.00E-90	100
Bifidobacterium	Bifidobacterium	75%	5.00E-90	100
Bifidobacterium	Bifidobacterium	75%	5.00E-90	100
Bifidobacterium	Bifidobacterium	75%	5.00E-90	100
Bifidobacterium	Bifidobacterium	75%	5.00E-90	100
Bifidobacterium	Bifidobacterium	75%	5.00E-90	100
Bifidobacterium	Bifidobacterium	75%	5.00E-90	100
Bifidobacterium	Bifidobacterium	75%	5.00E-90	100
Bifidobacterium	Bifidobacterium	75%	5.00E-90	100
Bifidobacterium	Bifidobacterium	75%	5.00E-90	100
Bifidobacterium	Bifidobacterium	75%	5.00E-90	100
Bifidobacterium	Bifidobacterium	75%	5.00E-90	100

Bifidobacterium	Bifidobacterium	75%	5.00E-90	100
Bifidobacterium	Bifidobacterium	75%	5.00E-90	100
Bifidobacterium	Bifidobacterium	75%	5.00E-90	100
Bifidobacterium	Bifidobacterium	75%	5.00E-90	100
Bifidobacterium	Bifidobacterium	75%	5.00E-90	100
Bifidobacterium	Bifidobacterium	75%	5.00E-90	100
Bifidobacterium	Bifidobacterium	75%	5.00E-90	100
Bifidobacterium	Bifidobacterium	75%	5.00E-90	100
Bifidobacterium	Bifidobacterium	75%	5.00E-90	100
Bifidobacterium	Bifidobacterium	75%	5.00E-90	100
Bifidobacterium	Bifidobacterium	75%	5.00E-90	100
Bifidobacterium	Bifidobacterium	75%	5.00E-90	100
Bifidobacterium	Bifidobacterium	75%	5.00E-90	100
Bifidobacterium	Bifidobacterium	75%	5.00E-90	100
Bifidobacterium	Bifidobacterium	75%	5.00E-90	100
Bifidobacterium	Bifidobacterium	75%	5.00E-90	100
Bifidobacterium	Bifidobacterium	75%	5.00E-90	100
Bifidobacterium	Bifidobacterium	75%	5.00E-90	100
Bifidobacterium	Bifidobacterium	75%	5.00E-90	100
Bifidobacterium	Bifidobacterium	75%	5.00E-90	100
Bifidobacterium	Bifidobacterium	75%	5.00E-90	100
Bifidobacterium	Bifidobacterium	75%	5.00E-90	100
Bifidobacterium	Bifidobacterium	75%	5.00E-90	100
Bifidobacterium	Bifidobacterium	75%	5.00E-90	100
Bifidobacterium	Bifidobacterium	75%	5.00E-90	100
Bifidobacterium	Bifidobacterium	75%	5.00E-90	100
Bifidobacterium	Bifidobacterium	75%	5.00E-90	100
Bifidobacterium	Bifidobacterium	75%	5.00E-90	100
Bifidobacterium	Bifidobacterium	75%	5.00E-90	100
Bifidobacterium	Bifidobacterium	75%	5.00E-90	100
Bifidobacterium	Bifidobacterium	75%	5.00E-90	100
Bifidobacterium	Bifidobacterium	75%	5.00E-90	100
Bifidobacterium	Bifidobacterium	75%	5.00E-90	100
Bifidobacterium	Bifidobacterium	75%	5.00E-90	100
Bifidobacterium	Bifidobacterium	75%	5.00E-90	100
Bifidobacterium	Bifidobacterium	75%	5.00E-90	100
Bifidobacterium	Bifidobacterium	75%	5.00E-90	100
Bifidobacterium	Bifidobacterium	75%	5.00E-90	100
Bifidobacterium	Bifidobacterium	75%	5.00E-90	100
Bifidobacterium	Bifidobacterium	75%	5.00E-90	100
Bifidobacterium	Bifidobacterium	75%	5.00E-90	100
Bifidobacterium	Bifidobacterium	75%	5.00E-90	100
Bifidobacterium	Bifidobacterium	75%	5.00E-90	100
Bifidobacterium	Bifidobacterium	75%	5.00E-90	100

Bifidobacterium	Bifidobacterium	75%	5.00E-90	100
Bifidobacterium	Bifidobacterium	75%	5.00E-90	100
Bifidobacterium	Bifidobacterium	75%	5.00E-90	100
Bifidobacterium	Bifidobacterium	75%	5.00E-90	100
Bifidobacterium	Bifidobacterium	75%	5.00E-90	100
Bifidobacterium	Bifidobacterium	75%	5.00E-90	100
Bifidobacterium	Bifidobacterium	75%	5.00E-90	100
Bifidobacterium	Bifidobacterium	75%	5.00E-90	100
Bifidobacterium	Bifidobacterium	75%	5.00E-90	100
Bifidobacterium	Bifidobacterium	75%	5.00E-90	100
Bifidobacterium	Bifidobacterium	75%	5.00E-90	100
Bifidobacterium	Bifidobacterium	75%	5.00E-90	100
Bifidobacterium	Bifidobacterium	75%	5.00E-90	100
Bifidobacterium	Bifidobacterium	75%	5.00E-90	100
Bifidobacterium	Bifidobacterium	75%	5.00E-90	100
Bifidobacterium	Bifidobacterium	75%	5.00E-90	100
Bifidobacterium	Bifidobacterium	75%	5.00E-90	100
Bifidobacterium	Bifidobacterium	75%	5.00E-90	100
Bifidobacterium	Bifidobacterium	75%	5.00E-90	100
Bifidobacterium	Bifidobacterium	75%	5.00E-90	100
Bifidobacterium	Bifidobacterium	75%	5.00E-90	100
Bifidobacterium	Bifidobacterium	75%	5.00E-90	100
Bifidobacterium	Bifidobacterium	75%	5.00E-90	100
Bifidobacterium	Bifidobacterium	75%	5.00E-90	100
Bifidobacterium	Bifidobacterium	75%	5.00E-90	100
Bifidobacterium	Bifidobacterium	75%	5.00E-90	100
Bifidobacterium	Bifidobacterium	75%	5.00E-90	100
Bifidobacterium	Bifidobacterium	75%	5.00E-90	100
Bifidobacterium	Bifidobacterium	75%	5.00E-90	100
Bifidobacterium	Bifidobacterium	75%	5.00E-90	100
Bifidobacterium	Bifidobacterium	75%	5.00E-90	100
Bifidobacterium	Bifidobacterium	75%	5.00E-90	100
Bifidobacterium	Bifidobacterium	75%	5.00E-90	100
Bifidobacterium	Bifidobacterium	75%	5.00E-90	100
Bifidobacterium	Bifidobacterium	75%	5.00E-90	100
Bifidobacterium	Bifidobacterium	75%	5.00E-90	100
Bifidobacterium	Bifidobacterium	75%	5.00E-90	100
Bifidobacterium	Bifidobacterium	75%	5.00E-90	100
Bifidobacterium	Bifidobacterium	75%	5.00E-90	100
Bifidobacterium	Bifidobacterium	75%	5.00E-90	100
Bifidobacterium	Bifidobacterium	75%	5.00E-90	100
Bifidobacterium	Bifidobacterium	75%	5.00E-90	100
Bifidobacterium	Bifidobacterium	75%	5.00E-90	100
Bifidobacterium	Bifidobacterium	75%	5.00E-90	100

Bifidobacterium	Bifidobacterium	75%	5.00E-90	100
Bifidobacterium	Bifidobacterium	75%	5.00E-90	100
Bifidobacterium	Bifidobacterium	75%	5.00E-90	100
Bifidobacterium	Bifidobacterium	75%	5.00E-90	100
Bifidobacterium	Bifidobacterium	75%	5.00E-90	100
Bifidobacterium	Bifidobacterium	75%	5.00E-90	100
Bifidobacterium	Bifidobacterium	75%	5.00E-90	100
Bifidobacterium	Bifidobacterium	75%	5.00E-90	100
Bifidobacterium	Bifidobacterium	75%	5.00E-90	100
Bifidobacterium	Bifidobacterium	75%	5.00E-90	100
Bifidobacterium	Bifidobacterium	75%	5.00E-90	100
Bifidobacterium	Bifidobacterium	75%	5.00E-90	100
Bifidobacterium	Bifidobacterium	75%	5.00E-90	100
Bifidobacterium	Bifidobacterium	75%	5.00E-90	100
Bifidobacterium	Bifidobacterium	75%	5.00E-90	100
Bifidobacterium	Bifidobacterium	75%	5.00E-90	100
Bifidobacterium	Bifidobacterium	75%	5.00E-90	100
Bifidobacterium	Bifidobacterium	75%	5.00E-90	100
Bifidobacterium	Bifidobacterium	75%	5.00E-90	100
Bifidobacterium	Bifidobacterium	75%	5.00E-90	100
Bifidobacterium	Bifidobacterium	75%	5.00E-90	100
Bifidobacterium	Bifidobacterium	75%	5.00E-90	100
Bifidobacterium	Bifidobacterium	75%	5.00E-90	100
Bifidobacterium	Bifidobacterium	75%	5.00E-90	100
Bifidobacterium	Bifidobacterium	75%	5.00E-90	100
Uncultured Bific	uncultured Bifid	100%	5.00E-90	91.98
Uncultured Bific	uncultured Bifid	75%	5.00E-90	100
Bifidobacterium	Bifidobacterium	75%	5.00E-90	100
Bifidobacterium	Bifidobacterium	75%	5.00E-90	100
Bifidobacterium	Bifidobacterium	75%	5.00E-90	100
Bifidobacterium	Bifidobacterium	75%	5.00E-90	100
Bifidobacterium	Bifidobacterium	75%	5.00E-90	100
Bifidobacterium	Bifidobacterium	75%	5.00E-90	100
Bifidobacterium	Bifidobacterium	75%	5.00E-90	100
Bifidobacterium	Bifidobacterium	75%	5.00E-90	100
Bifidobacterium	Bifidobacterium	75%	5.00E-90	100
Bifidobacterium	Bifidobacterium	75%	5.00E-90	100
Bifidobacterium	Bifidobacterium	75%	5.00E-90	100
Bifidobacterium	Bifidobacterium	75%	5.00E-90	100
Bifidobacterium	Bifidobacterium	75%	5.00E-90	100
Bifidobacterium	Bifidobacterium	75%	5.00E-90	100
Bifidobacterium	Bifidobacterium	75%	5.00E-90	100
Bifidobacterium	Bifidobacterium	75%	5.00E-90	100
Bifidobacterium	Bifidobacterium	75%	5.00E-90	100

Bifidobacterium	Bifidobacterium	75%	5.00E-90	100
Bifidobacterium	Bifidobacterium	75%	5.00E-90	100
Bifidobacterium	Bifidobacterium	75%	5.00E-90	100
Bifidobacterium	Bifidobacterium	75%	5.00E-90	100
Bifidobacterium	Bifidobacterium	75%	5.00E-90	100
Bifidobacterium	Bifidobacterium	75%	5.00E-90	100
Bifidobacterium	Bifidobacterium	75%	5.00E-90	100
Bifidobacterium	Bifidobacterium	75%	5.00E-90	100
Bifidobacterium	Bifidobacterium	75%	5.00E-90	100
Bifidobacterium	Bifidobacterium	75%	5.00E-90	100
Bifidobacterium	Bifidobacterium	75%	5.00E-90	100
Bifidobacterium	Bifidobacterium	75%	5.00E-90	100
Bifidobacterium	Bifidobacterium	75%	5.00E-90	100
Bifidobacterium	Bifidobacterium	75%	5.00E-90	100
Bifidobacterium	Bifidobacterium	75%	5.00E-90	100
Bifidobacterium	Bifidobacterium	75%	5.00E-90	100
Bifidobacterium	Bifidobacterium	75%	5.00E-90	100
Bifidobacterium	Bifidobacterium	75%	5.00E-90	100
Bifidobacterium	Bifidobacterium	75%	5.00E-90	100
Bifidobacterium	Bifidobacterium	75%	5.00E-90	100
Bifidobacterium	Bifidobacterium	75%	5.00E-90	100
Bifidobacterium	Bifidobacterium	75%	5.00E-90	100
Bifidobacterium	Bifidobacterium	75%	5.00E-90	100
Bifidobacterium	Bifidobacterium	75%	5.00E-90	100
Bifidobacterium	Bifidobacterium	75%	5.00E-90	100
Bifidobacterium	Bifidobacterium	75%	5.00E-90	100
Bifidobacterium	Bifidobacterium	75%	5.00E-90	100
Bifidobacterium	Bifidobacterium	75%	5.00E-90	100
Bifidobacterium	Bifidobacterium	75%	5.00E-90	100
Bifidobacterium	Bifidobacterium	75%	5.00E-90	100
Bifidobacterium	Bifidobacterium	75%	5.00E-90	100
Bifidobacterium	Bifidobacterium	75%	5.00E-90	100
Bifidobacterium	Bifidobacterium	75%	5.00E-90	100
Bifidobacterium	Bifidobacterium	75%	5.00E-90	100
Bifidobacterium	Bifidobacterium	75%	5.00E-90	100
Bifidobacterium	Bifidobacterium	75%	5.00E-90	100
Bifidobacterium	Bifidobacterium	75%	5.00E-90	100
Bifidobacterium	Bifidobacterium	75%	5.00E-90	100
Bifidobacterium	Bifidobacterium	75%	5.00E-90	100
Bifidobacterium	Bifidobacterium	75%	5.00E-90	100
Bifidobacterium	Bifidobacterium	75%	5.00E-90	100
Bifidobacterium	Bifidobacterium	75%	5.00E-90	100
Bifidobacterium	Bifidobacterium	75%	5.00E-90	100
Bifidobacterium	Bifidobacterium	75%	5.00E-90	100

Bifidobacterium	Bifidobacterium	75%	5.00E-90	100
Bifidobacterium	Bifidobacterium	75%	5.00E-90	100
Bifidobacterium	Bifidobacterium	75%	5.00E-90	100
Bifidobacterium	Bifidobacterium	75%	5.00E-90	100
Bifidobacterium	Bifidobacterium	75%	5.00E-90	100
Bifidobacterium	Bifidobacterium	75%	5.00E-90	100
Bifidobacterium	Bifidobacterium	75%	5.00E-90	100
Bifidobacterium	Bifidobacterium	75%	5.00E-90	100
Bifidobacterium	Bifidobacterium	75%	5.00E-90	100
Bifidobacterium	Bifidobacterium	75%	5.00E-90	100
Bifidobacterium	Bifidobacterium	75%	5.00E-90	100
Bifidobacterium	Bifidobacterium	75%	5.00E-90	100
Bifidobacterium	Bifidobacterium	75%	5.00E-90	100
Bifidobacterium	Bifidobacterium	75%	5.00E-90	100
Bifidobacterium	Bifidobacterium	75%	5.00E-90	100
Bifidobacterium	Bifidobacterium	75%	5.00E-90	100
Bifidobacterium	Bifidobacterium	75%	5.00E-90	100
Bifidobacterium	Bifidobacterium	75%	5.00E-90	100
Bifidobacterium	Bifidobacterium	75%	5.00E-90	100
Bifidobacterium	Bifidobacterium	75%	5.00E-90	100
Bifidobacterium	Bifidobacterium	75%	5.00E-90	100
Bifidobacterium	Bifidobacterium	75%	5.00E-90	100
Bifidobacterium	Bifidobacterium	75%	5.00E-90	100
Bifidobacterium	Bifidobacterium	75%	5.00E-90	100
Bifidobacterium	Bifidobacterium	75%	5.00E-90	100
Bifidobacterium	Bifidobacterium	75%	5.00E-90	100
Bifidobacterium	Bifidobacterium	75%	5.00E-90	100
Bifidobacterium	Bifidobacterium	75%	5.00E-90	100
Bifidobacterium	Bifidobacterium	75%	5.00E-90	100
Bifidobacterium	Bifidobacterium	75%	5.00E-90	100
Bifidobacterium	Bifidobacterium	75%	5.00E-90	100
Bifidobacterium	Bifidobacterium	75%	5.00E-90	100
Bifidobacterium	Bifidobacterium	75%	5.00E-90	100
Bifidobacterium	Bifidobacterium	75%	5.00E-90	100
Bifidobacterium	Bifidobacterium	75%	5.00E-90	100
Bifidobacterium	Bifidobacterium	75%	5.00E-90	100
Bifidobacterium	Bifidobacterium	75%	5.00E-90	100
Bifidobacterium	Bifidobacterium	75%	5.00E-90	100
Bifidobacterium	Bifidobacterium	75%	5.00E-90	100
Bifidobacterium	Bifidobacterium	75%	5.00E-90	100
Bifidobacterium	Bifidobacterium	75%	5.00E-90	100
Bifidobacterium	Bifidobacterium	75%	5.00E-90	100
Bifidobacterium	Bifidobacterium	75%	5.00E-90	100
Bifidobacterium	Bifidobacterium	75%	5.00E-90	100

Bifidobacterium	Bifidobacterium	75%	5.00E-90	100
Bifidobacterium	Bifidobacterium	75%	5.00E-90	100
Bifidobacterium	Bifidobacterium	75%	5.00E-90	100
Bifidobacterium	Bifidobacterium	75%	5.00E-90	100
Bifidobacterium	Bifidobacterium	75%	5.00E-90	100
Bifidobacterium	Bifidobacterium	75%	5.00E-90	100
Uncultured Bific	uncultured Bifid	75%	5.00E-90	100
Uncultured Bific	uncultured Bifid	75%	5.00E-90	100
Bifidobacterium	Bifidobacterium	82%	5.00E-90	97.44
Bifidobacterium	Bifidobacterium	75%	2.00E-89	100
Bifidobacterium	Bifidobacterium	75%	2.00E-89	100
Bifidobacterium	Bifidobacterium	75%	2.00E-89	100
Bifidobacterium	Bifidobacterium	75%	2.00E-89	100
Bifidobacterium	Bifidobacterium	75%	2.00E-89	100
Bifidobacterium	Bifidobacterium	75%	2.00E-89	100
Bifidobacterium	Bifidobacterium	75%	2.00E-89	100
Bifidobacterium	Bifidobacterium	75%	2.00E-89	100
Bifidobacterium	Bifidobacterium	75%	2.00E-89	100
Bifidobacterium	Bifidobacterium	75%	2.00E-89	100
Bifidobacterium	Bifidobacterium	75%	2.00E-89	100
Bifidobacterium	Bifidobacterium	75%	2.00E-89	100
Bifidobacterium	Bifidobacterium	75%	2.00E-89	100
Bifidobacterium	Bifidobacterium	75%	2.00E-89	100
Bifidobacterium	Bifidobacterium	75%	2.00E-89	100
Bifidobacterium	Bifidobacterium	75%	2.00E-89	100
Bifidobacterium	Bifidobacterium	75%	2.00E-89	100
Bifidobacterium	Bifidobacterium	75%	2.00E-89	100
Bifidobacterium	Bifidobacterium	75%	2.00E-89	100
Uncultured Bific	uncultured Bifid	75%	2.00E-89	100
Uncultured Bific	uncultured Bifid	75%	2.00E-89	100
Uncultured Bific	uncultured Bifid	75%	2.00E-89	100
Uncultured Bific	uncultured Bifid	75%	2.00E-89	100
Uncultured Bific	uncultured Bifid	75%	2.00E-89	100
Uncultured Bific	uncultured Bifid	75%	2.00E-89	100
Uncultured Bific	uncultured Bifid	75%	2.00E-89	100
Uncultured Bific	uncultured Bifid	75%	2.00E-89	100
Uncultured Bific	uncultured Bifid	75%	2.00E-89	100
Uncultured Bific	uncultured Bifid	75%	2.00E-89	100
Uncultured Bific	uncultured Bifid	75%	2.00E-89	100
Uncultured Bific	uncultured Bifid	75%	2.00E-89	100
Uncultured Bific	uncultured Bifid	75%	2.00E-89	100
Uncultured Bific	uncultured Bifid	75%	2.00E-89	100
Uncultured Bific	uncultured Bifid	75%	2.00E-89	100
Uncultured Bific	uncultured Bifid	75%	2.00E-89	100

Uncultured Bific	uncultured Bifid	75%	2.00E-89	100
Uncultured Bific	uncultured Bifid	75%	2.00E-89	100
Uncultured Bific	uncultured Bifid	75%	2.00E-89	100
Uncultured Bific	uncultured Bifid	75%	2.00E-89	100
Uncultured Bific	uncultured Bifid	75%	2.00E-89	100
Uncultured Bific	uncultured Bifid	75%	2.00E-89	100
Uncultured Bific	uncultured Bifid	75%	2.00E-89	100
Uncultured Bific	uncultured Bifid	75%	2.00E-89	100
Uncultured Bific	uncultured Bifid	75%	2.00E-89	100
Uncultured Bific	uncultured Bifid	75%	2.00E-89	100
Uncultured Bific	uncultured Bifid	75%	2.00E-89	100
Uncultured Bific	uncultured Bifid	75%	2.00E-89	100
Uncultured Bific	uncultured Bifid	75%	2.00E-89	100
Uncultured Bific	uncultured Bifid	75%	2.00E-89	100
Uncultured Bific	uncultured Bifid	75%	2.00E-89	100
Uncultured Bific	uncultured Bifid	75%	2.00E-89	100
Uncultured Bific	uncultured Bifid	75%	2.00E-89	100
Bifidobacterium	Bifidobacterium	75%	2.00E-89	100
Bifidobacterium	Bifidobacterium	75%	2.00E-89	100
Bifidobacterium	Bifidobacterium	75%	2.00E-89	100
Bifidobacterium	Bifidobacterium	75%	2.00E-89	100
Bifidobacterium	Bifidobacterium	75%	2.00E-89	100
Bifidobacterium	Bifidobacterium	75%	2.00E-89	100
Bifidobacterium	Bifidobacterium	75%	2.00E-89	100
Bifidobacterium	Bifidobacterium	75%	2.00E-89	100
Bifidobacterium	Bifidobacterium	75%	2.00E-89	100
Bifidobacterium	Bifidobacterium	75%	2.00E-89	100
Bifidobacterium	Bifidobacterium	75%	2.00E-89	100
Uncultured Bific	uncultured Bifid	75%	2.00E-89	100
Uncultured Bific	uncultured Bifid	75%	2.00E-89	100
Uncultured Bific	uncultured Bifid	76%	2.00E-89	99.45
Uncultured Bific	uncultured Bifid	75%	2.00E-89	100
Uncultured Bific	uncultured Bifid	75%	2.00E-89	100
Uncultured Bific	uncultured Bifid	75%	2.00E-89	100
Uncultured Bific	uncultured Bifid	75%	2.00E-89	100
Uncultured Bific	uncultured Bifid	75%	2.00E-89	100
Uncultured Bific	uncultured Bifid	76%	2.00E-89	99.45
Uncultured Bific	uncultured Bifid	76%	2.00E-89	99.45
Uncultured Bific	uncultured Bifid	75%	2.00E-89	100
Uncultured Bific	uncultured Bifid	75%	2.00E-89	100
Uncultured Bific	uncultured Bifid	76%	2.00E-89	99.45
Uncultured Bific	uncultured Bifid	76%	2.00E-89	99.45
Uncultured Bific	uncultured Bifid	76%	2.00E-89	99.45
Uncultured Bific	uncultured Bifid	76%	2.00E-89	99.45

Uncultured Bifid	uncultured Bifid	76%	2.00E-89	99.45
Uncultured Bifid	uncultured Bifid	76%	2.00E-89	99.45
Uncultured Bifid	uncultured Bifid	75%	2.00E-89	100
Uncultured Bifid	uncultured Bifid	75%	2.00E-89	100
Uncultured Bifid	uncultured Bifid	75%	2.00E-89	100
Uncultured Bifid	uncultured Bifid	75%	2.00E-89	100
Uncultured Bifid	uncultured Bifid	75%	2.00E-89	100
Bifidobacterium	Bifidobacterium	84%	2.00E-89	96.5
Uncultured Bifid	uncultured Bifid	75%	2.00E-89	100
Bifidobacterium	Bifidobacterium	76%	6.00E-89	99.45
Bifidobacterium	Bifidobacterium	76%	6.00E-89	99.45
Bifidobacterium	Bifidobacterium	76%	6.00E-89	99.45
Bifidobacterium	Bifidobacterium	76%	6.00E-89	99.45
Bifidobacterium	Bifidobacterium	76%	6.00E-89	99.45
Bifidobacterium	Bifidobacterium	76%	6.00E-89	99.45
Bifidobacterium	Bifidobacterium	76%	6.00E-89	99.45
Bifidobacterium	Bifidobacterium	76%	6.00E-89	99.45
Bifidobacterium	Bifidobacterium	76%	6.00E-89	99.45
Bifidobacterium	Bifidobacterium	76%	6.00E-89	99.45
Bifidobacterium	Bifidobacterium	76%	6.00E-89	99.45
Bifidobacterium	Bifidobacterium	76%	6.00E-89	99.45
Bifidobacterium	Bifidobacterium	76%	6.00E-89	99.45
Bifidobacterium	Bifidobacterium	76%	6.00E-89	99.45
Bifidobacterium	Bifidobacterium	76%	6.00E-89	99.45
Bifidobacterium	Bifidobacterium	86%	6.00E-89	95.69
Bifidobacterium	Bifidobacterium	75%	6.00E-89	100
Uncultured Bifid	uncultured Bifid	76%	6.00E-89	99.45
Bifidobacterium	Bifidobacterium	81%	6.00E-89	97.41
Bifidobacterium	Bifidobacterium	75%	6.00E-89	100
Bifidobacterium	Bifidobacterium	76%	6.00E-89	99.45
Bifidobacterium	Bifidobacterium	76%	6.00E-89	99.45
Bifidobacterium	Bifidobacterium	76%	6.00E-89	99.45
Bifidobacterium	Bifidobacterium	76%	6.00E-89	99.45
Bifidobacterium	Bifidobacterium	76%	6.00E-89	99.45
Uncultured Bifid	uncultured Bifid	75%	6.00E-89	100
Uncultured Bifid	uncultured Bifid	75%	6.00E-89	100
Uncultured Bifid	uncultured Bifid	76%	6.00E-89	99.45
Uncultured Bifid	uncultured Bifid	75%	6.00E-89	100
Uncultured Bifid	uncultured Bifid	75%	6.00E-89	100
Uncultured Bifid	uncultured Bifid	76%	6.00E-89	99.45
Uncultured Bifid	uncultured Bifid	76%	6.00E-89	99.45
Uncultured Bifid	uncultured Bifid	76%	6.00E-89	99.45
Uncultured Bifid	uncultured Bifid	76%	6.00E-89	99.45
Uncultured Bifid	uncultured Bifid	76%	6.00E-89	99.45

Bifidobacterium	Bifidobacterium	81%	6.00E-89	97.41
Bifidobacterium	Bifidobacterium	75%	2.00E-88	99.44
Bifidobacterium	Bifidobacterium	75%	2.00E-88	99.44
Bifidobacterium	Bifidobacterium	75%	2.00E-88	99.44
Bifidobacterium	Bifidobacterium	75%	2.00E-88	99.45
Bifidobacterium	Bifidobacterium	75%	2.00E-88	99.44
Bifidobacterium	Bifidobacterium	75%	2.00E-88	99.45
Bifidobacterium	Bifidobacterium	75%	2.00E-88	99.44
Bifidobacterium	Bifidobacterium	75%	2.00E-88	99.45
Bifidobacterium	Bifidobacterium	75%	2.00E-88	99.45
Bifidobacterium	Bifidobacterium	75%	2.00E-88	99.44
Bifidobacterium	Bifidobacterium	75%	2.00E-88	99.45
Bifidobacterium	Bifidobacterium	75%	2.00E-88	99.44
Bifidobacterium	Bifidobacterium	75%	2.00E-88	99.44
Bifidobacterium	Bifidobacterium	75%	2.00E-88	99.44
Bifidobacterium	Bifidobacterium	75%	2.00E-88	99.44
Bifidobacterium	Bifidobacterium	75%	2.00E-88	99.44
Bifidobacterium	Bifidobacterium	75%	2.00E-88	99.44
Bifidobacterium	Bifidobacterium	75%	2.00E-88	99.44
Bifidobacterium	Bifidobacterium	75%	2.00E-88	99.44
Bifidobacterium	Bifidobacterium	75%	2.00E-88	99.44
Bifidobacterium	Bifidobacterium	75%	2.00E-88	99.44
Bifidobacterium	Bifidobacterium	75%	2.00E-88	99.44
Bifidobacterium	Bifidobacterium	75%	2.00E-88	99.44
Bifidobacterium	Bifidobacterium	75%	2.00E-88	99.44
Bifidobacterium	Bifidobacterium	75%	2.00E-88	99.44
Bifidobacterium	Bifidobacterium	75%	2.00E-88	99.44
Bifidobacterium	Bifidobacterium	75%	2.00E-88	99.44
Bifidobacterium	Bifidobacterium	75%	2.00E-88	99.45
Bifidobacterium	Bifidobacterium	75%	2.00E-88	99.44
Bifidobacterium	Bifidobacterium	75%	2.00E-88	99.44
Bifidobacterium	Bifidobacterium	75%	2.00E-88	99.44
Bifidobacterium	Bifidobacterium	75%	2.00E-88	99.44
Bifidobacterium	Bifidobacterium	75%	2.00E-88	99.44
Bifidobacterium	Bifidobacterium	75%	2.00E-88	99.44
Bifidobacterium	Bifidobacterium	75%	2.00E-88	99.44
Bifidobacterium	Bifidobacterium	75%	2.00E-88	99.45
Bifidobacterium	Bifidobacterium	75%	2.00E-88	99.44
Bifidobacterium	Bifidobacterium	75%	2.00E-88	99.44
Bifidobacterium	Bifidobacterium	75%	2.00E-88	99.44
Bifidobacterium	Bifidobacterium	75%	2.00E-88	99.44
Bifidobacterium	Bifidobacterium	74%	2.00E-88	100
Bifidobacterium	Bifidobacterium	75%	2.00E-88	99.44
Bifidobacterium	Bifidobacterium	75%	2.00E-88	99.44

Bifidobacterium	Bifidobacterium	75%	2.00E-88	99.44
Bifidobacterium	Bifidobacterium	75%	2.00E-88	99.44
Bifidobacterium	Bifidobacterium	75%	2.00E-88	99.44
Bifidobacterium	Bifidobacterium	75%	2.00E-88	99.44
Bifidobacterium	Bifidobacterium	75%	2.00E-88	99.44
Bifidobacterium	Bifidobacterium	75%	2.00E-88	99.44
Bifidobacterium	Bifidobacterium	75%	2.00E-88	99.44
Bifidobacterium	Bifidobacterium	75%	2.00E-88	99.44
Bifidobacterium	Bifidobacterium	75%	2.00E-88	99.45
Bifidobacterium	Bifidobacterium	75%	2.00E-88	99.44
Bifidobacterium	Bifidobacterium	75%	2.00E-88	99.44
Bifidobacterium	Bifidobacterium	75%	2.00E-88	99.44
Bifidobacterium	Bifidobacterium	75%	2.00E-88	99.44
Bifidobacterium	Bifidobacterium	75%	2.00E-88	99.44
Bifidobacterium	Bifidobacterium	75%	2.00E-88	99.44
Bifidobacterium	Bifidobacterium	75%	2.00E-88	99.44
Bifidobacterium	Bifidobacterium	75%	2.00E-88	99.45
Bifidobacterium	Bifidobacterium	75%	2.00E-88	99.44
Bifidobacterium	Bifidobacterium	75%	2.00E-88	99.44
Bifidobacterium	Bifidobacterium	75%	2.00E-88	99.44
Bifidobacterium	Bifidobacterium	75%	2.00E-88	99.44
Bifidobacterium	Bifidobacterium	75%	2.00E-88	99.44
Bifidobacterium	Bifidobacterium	75%	2.00E-88	99.44
Bifidobacterium	Bifidobacterium	75%	2.00E-88	99.44
Bifidobacterium	Bifidobacterium	75%	2.00E-88	99.44
Bifidobacterium	Bifidobacterium	75%	2.00E-88	99.44
Bifidobacterium	Bifidobacterium	75%	2.00E-88	99.44
Bifidobacterium	Bifidobacterium	75%	2.00E-88	99.44
Bifidobacterium	Bifidobacterium	75%	2.00E-88	99.44
Bifidobacterium	Bifidobacterium	74%	2.00E-88	100
Bifidobacterium	Bifidobacterium	75%	2.00E-88	99.44
Bifidobacterium	Bifidobacterium	75%	2.00E-88	99.45
Bifidobacterium	Bifidobacterium	75%	2.00E-88	99.45
Bifidobacterium	Bifidobacterium	75%	2.00E-88	99.44
Bifidobacterium	Bifidobacterium	75%	2.00E-88	99.44
Bifidobacterium	Bifidobacterium	75%	2.00E-88	99.44
Bifidobacterium	Bifidobacterium	75%	2.00E-88	99.45
Bifidobacterium	Bifidobacterium	75%	2.00E-88	99.44
Bifidobacterium	Bifidobacterium	75%	2.00E-88	99.45
Bifidobacterium	Bifidobacterium	75%	2.00E-88	99.45
Bifidobacterium	Bifidobacterium	75%	2.00E-88	99.45
Bifidobacterium	Bifidobacterium	75%	2.00E-88	99.44
Bifidobacterium	Bifidobacterium	75%	2.00E-88	99.45
Bifidobacterium	Bifidobacterium	75%	2.00E-88	99.44

Bifidobacterium	Bifidobacterium	75%	2.00E-88	99.44
Bifidobacterium	Bifidobacterium	75%	2.00E-88	99.45
Bifidobacterium	Bifidobacterium	75%	2.00E-88	99.44
Bifidobacterium	Bifidobacterium	75%	2.00E-88	99.44
Bifidobacterium	Bifidobacterium	75%	2.00E-88	99.44
Bifidobacterium	Bifidobacterium	75%	2.00E-88	99.44
Bifidobacterium	Bifidobacterium	75%	2.00E-88	99.44
Bifidobacterium	Bifidobacterium	75%	2.00E-88	99.45
Bifidobacterium	Bifidobacterium	75%	2.00E-88	99.44
Bifidobacterium	Bifidobacterium	75%	2.00E-88	99.44
Bifidobacterium	Bifidobacterium	75%	2.00E-88	99.44
Bifidobacterium	Bifidobacterium	75%	2.00E-88	99.44
Bifidobacterium	Bifidobacterium	75%	2.00E-88	99.44
Bifidobacterium	Bifidobacterium	75%	2.00E-88	99.44
Bifidobacterium	Bifidobacterium	75%	2.00E-88	99.45
Bifidobacterium	Bifidobacterium	75%	2.00E-88	99.44
Bifidobacterium	Bifidobacterium	75%	2.00E-88	99.44
Bifidobacterium	Bifidobacterium	75%	2.00E-88	99.44
Bifidobacterium	Bifidobacterium	75%	2.00E-88	99.45
Bifidobacterium	Bifidobacterium	75%	2.00E-88	99.44
Bifidobacterium	Bifidobacterium	75%	2.00E-88	99.45
Bifidobacterium	Bifidobacterium	75%	2.00E-88	99.44
Bifidobacterium	Bifidobacterium	75%	2.00E-88	99.44
Bifidobacterium	Bifidobacterium	75%	2.00E-88	99.44
Bifidobacterium	Bifidobacterium	75%	2.00E-88	99.45
Bifidobacterium	Bifidobacterium	75%	2.00E-88	99.45
Bifidobacterium	Bifidobacterium	75%	2.00E-88	99.45
Bifidobacterium	Bifidobacterium	75%	2.00E-88	99.44
Bifidobacterium	Bifidobacterium	75%	2.00E-88	99.44
Bifidobacterium	Bifidobacterium	75%	2.00E-88	99.45
Bifidobacterium	Bifidobacterium	75%	2.00E-88	99.44
Bifidobacterium	Bifidobacterium	75%	2.00E-88	99.45
Bifidobacterium	Bifidobacterium	75%	2.00E-88	99.44
Bifidobacterium	Bifidobacterium	75%	2.00E-88	99.44
Bifidobacterium	Bifidobacterium	75%	2.00E-88	99.44
Bifidobacterium	Bifidobacterium	75%	2.00E-88	99.44
Bifidobacterium	Bifidobacterium	75%	2.00E-88	99.44
Bifidobacterium	Bifidobacterium	75%	2.00E-88	99.44
Bifidobacterium	Bifidobacterium	75%	2.00E-88	99.44
Bifidobacterium	Bifidobacterium	75%	2.00E-88	99.44
Bifidobacterium	Bifidobacterium	75%	2.00E-88	99.45
Bifidobacterium	Bifidobacterium	75%	2.00E-88	99.45
Bifidobacterium	Bifidobacterium	75%	2.00E-88	99.44
Bifidobacterium	Bifidobacterium	75%	2.00E-88	99.44

Bifidobacterium	Bifidobacterium	75%	2.00E-88	99.44
Bifidobacterium	Bifidobacterium	75%	2.00E-88	99.45
Bifidobacterium	Bifidobacterium	75%	2.00E-88	99.44
Bifidobacterium	Bifidobacterium	75%	2.00E-88	99.45
Bifidobacterium	Bifidobacterium	75%	2.00E-88	99.44
Bifidobacterium	Bifidobacterium	75%	2.00E-88	99.44
Bifidobacterium	Bifidobacterium	75%	2.00E-88	99.45
Bifidobacterium	Bifidobacterium	75%	2.00E-88	99.45
Bifidobacterium	Bifidobacterium	75%	2.00E-88	99.44
Bifidobacterium	Bifidobacterium	75%	2.00E-88	99.44
Bifidobacterium	Bifidobacterium	75%	2.00E-88	99.45
Bifidobacterium	Bifidobacterium	75%	2.00E-88	99.45
Bifidobacterium	Bifidobacterium	75%	2.00E-88	99.45
Bifidobacterium	Bifidobacterium	75%	2.00E-88	99.45
Bifidobacterium	Bifidobacterium	75%	2.00E-88	99.44
Bifidobacterium	Bifidobacterium	75%	2.00E-88	99.45
Bifidobacterium	Bifidobacterium	75%	2.00E-88	99.44
Bifidobacterium	Bifidobacterium	74%	2.00E-88	100
Uncultured Bifid	uncultured Bifid	98%	2.00E-88	91.95
Uncultured Bifid	uncultured Bifid	98%	2.00E-88	91.88
Bifidobacterium	Bifidobacterium	75%	2.00E-88	99.45
Bifidobacterium	Bifidobacterium	75%	2.00E-88	99.44
Bifidobacterium	Bifidobacterium	75%	2.00E-88	99.45
Uncultured Bifid	uncultured Bifid	75%	2.00E-88	99.44
Uncultured Bifid	uncultured Bifid	74%	2.00E-88	100
Uncultured Bifid	uncultured Bifid	75%	2.00E-88	99.44
Uncultured Bifid	uncultured Bifid	75%	2.00E-88	99.44
Uncultured Bifid	uncultured Bifid	75%	2.00E-88	99.44
Uncultured Bifid	uncultured Bifid	75%	2.00E-88	99.44
Uncultured Bifid	uncultured Bifid	75%	2.00E-88	99.44
Uncultured Bifid	uncultured Bifid	75%	2.00E-88	99.44
Uncultured Bifid	uncultured Bifid	75%	2.00E-88	99.44
Uncultured Bifid	uncultured Bifid	75%	2.00E-88	99.44
Uncultured Bifid	uncultured Bifid	75%	2.00E-88	99.44
Uncultured Bifid	uncultured Bifid	75%	2.00E-88	99.44
Uncultured Bifid	uncultured Bifid	75%	2.00E-88	99.44
Uncultured Bifid	uncultured Bifid	75%	2.00E-88	99.44
Uncultured Bifid	uncultured Bifid	75%	2.00E-88	99.44
Uncultured Bifid	uncultured Bifid	75%	2.00E-88	99.44
Uncultured Bifid	uncultured Bifid	75%	2.00E-88	99.44
Uncultured Bifid	uncultured Bifid	75%	2.00E-88	99.44
Uncultured Bifid	uncultured Bifid	75%	2.00E-88	99.44
Uncultured Bifid	uncultured Bifid	75%	2.00E-88	99.44
Uncultured Bifid	uncultured Bifid	75%	2.00E-88	99.44

Uncultured Bific	uncultured Bifid	75%	2.00E-88	99.44
Uncultured Bific	uncultured Bifid	75%	2.00E-88	99.44
Uncultured Bific	uncultured Bifid	75%	2.00E-88	99.44
Uncultured Bific	uncultured Bifid	75%	2.00E-88	99.44
Uncultured Bific	uncultured Bifid	75%	2.00E-88	99.44
Uncultured Bific	uncultured Bifid	75%	2.00E-88	99.44
Uncultured Bific	uncultured Bifid	75%	2.00E-88	99.44
Uncultured Bific	uncultured Bifid	75%	2.00E-88	99.44
Bifidobacterium	Bifidobacterium	75%	2.00E-88	99.45
Bifidobacterium	Bifidobacterium	75%	2.00E-88	99.44
Bifidobacterium	Bifidobacterium	75%	2.00E-88	99.45
Bifidobacterium	Bifidobacterium	75%	2.00E-88	99.45
Bifidobacterium	Bifidobacterium	75%	2.00E-88	99.45
Bifidobacterium	Bifidobacterium	75%	2.00E-88	99.44
Bifidobacterium	Bifidobacterium	75%	2.00E-88	99.45
Bifidobacterium	Bifidobacterium	75%	2.00E-88	99.45
Bifidobacterium	Bifidobacterium	75%	2.00E-88	99.45
Bifidobacterium	Bifidobacterium	75%	2.00E-88	99.45
Bifidobacterium	Bifidobacterium	75%	2.00E-88	99.44
Bifidobacterium	Bifidobacterium	75%	2.00E-88	99.45
Bifidobacterium	Bifidobacterium	75%	2.00E-88	99.45
Bifidobacterium	Bifidobacterium	75%	2.00E-88	99.45
Bifidobacterium	Bifidobacterium	75%	2.00E-88	99.45
Bifidobacterium	Bifidobacterium	75%	2.00E-88	99.45
Bifidobacterium	Bifidobacterium	75%	2.00E-88	99.45
Bifidobacterium	Bifidobacterium	75%	2.00E-88	99.44
Bifidobacterium	Bifidobacterium	75%	2.00E-88	99.45
Bifidobacterium	Bifidobacterium	75%	2.00E-88	99.45
Bifidobacterium	Bifidobacterium	75%	2.00E-88	99.45
Bifidobacterium	Bifidobacterium	75%	2.00E-88	99.45
Bifidobacterium	Bifidobacterium	75%	2.00E-88	99.45
Bifidobacterium	Bifidobacterium	75%	2.00E-88	99.44
Bifidobacterium	Bifidobacterium	75%	2.00E-88	99.45
Bifidobacterium	Bifidobacterium	75%	2.00E-88	99.44
Bifidobacterium	Bifidobacterium	75%	2.00E-88	99.45
Bifidobacterium	Bifidobacterium	75%	2.00E-88	99.45
Bifidobacterium	Bifidobacterium	75%	2.00E-88	99.44
Bifidobacterium	Bifidobacterium	75%	2.00E-88	99.44
Bifidobacterium	Bifidobacterium	75%	2.00E-88	99.45
Bifidobacterium	Bifidobacterium	75%	2.00E-88	99.45
Bifidobacterium	Bifidobacterium	75%	2.00E-88	99.45
Bifidobacterium	Bifidobacterium	75%	2.00E-88	99.45
Bifidobacterium	Bifidobacterium	75%	2.00E-88	99.45
Bifidobacterium	Bifidobacterium	75%	2.00E-88	99.45

Bifidobacterium	Bifidobacterium	75%	2.00E-88	99.45
Bifidobacterium	Bifidobacterium	75%	2.00E-88	99.45
Bifidobacterium	Bifidobacterium	75%	2.00E-88	99.45
Bifidobacterium	Bifidobacterium	75%	2.00E-88	99.45
Bifidobacterium	Bifidobacterium	75%	2.00E-88	99.44
Bifidobacterium	Bifidobacterium	75%	2.00E-88	99.44
Bifidobacterium	Bifidobacterium	75%	2.00E-88	99.45
Bifidobacterium	Bifidobacterium	75%	2.00E-88	99.44
Bifidobacterium	Bifidobacterium	75%	2.00E-88	99.44
Bifidobacterium	Bifidobacterium	75%	2.00E-88	99.45
Bifidobacterium	Bifidobacterium	75%	2.00E-88	99.45
Bifidobacterium	Bifidobacterium	75%	2.00E-88	99.45
Bifidobacterium	Bifidobacterium	75%	2.00E-88	99.45
Bifidobacterium	Bifidobacterium	75%	2.00E-88	99.45
Bifidobacterium	Bifidobacterium	75%	2.00E-88	99.45
Bifidobacterium	Bifidobacterium	75%	2.00E-88	99.45
Bifidobacterium	Bifidobacterium	75%	2.00E-88	99.45
Bifidobacterium	Bifidobacterium	75%	2.00E-88	99.44
Bifidobacterium	Bifidobacterium	75%	2.00E-88	99.44
Bifidobacterium	Bifidobacterium	74%	2.00E-88	100
Bifidobacterium	Bifidobacterium	75%	2.00E-88	99.45
Bifidobacterium	Bifidobacterium	75%	2.00E-88	99.45
Bifidobacterium	Bifidobacterium	75%	2.00E-88	99.45
Bifidobacterium	Bifidobacterium	75%	2.00E-88	99.44
Bifidobacterium	Bifidobacterium	75%	2.00E-88	99.45
Bifidobacterium	Bifidobacterium	75%	2.00E-88	99.45
Bifidobacterium	Bifidobacterium	75%	2.00E-88	99.45
Bifidobacterium	Bifidobacterium	75%	2.00E-88	99.45
Bifidobacterium	Bifidobacterium	75%	2.00E-88	99.45
Bifidobacterium	Bifidobacterium	75%	2.00E-88	99.45
Bifidobacterium	Bifidobacterium	75%	2.00E-88	99.45
Bifidobacterium	Bifidobacterium	75%	2.00E-88	99.45
Bifidobacterium	Bifidobacterium	75%	2.00E-88	99.45
Bifidobacterium	Bifidobacterium	75%	2.00E-88	99.45
Bifidobacterium	Bifidobacterium	75%	2.00E-88	99.45
Bifidobacterium	Bifidobacterium	75%	2.00E-88	99.45
Bifidobacterium	Bifidobacterium	75%	2.00E-88	99.45
Bifidobacterium	Bifidobacterium	75%	2.00E-88	99.44
Bifidobacterium	Bifidobacterium	75%	2.00E-88	99.45
Bifidobacterium	Bifidobacterium	75%	2.00E-88	99.45
Bifidobacterium	Bifidobacterium	75%	2.00E-88	99.44
Bifidobacterium	Bifidobacterium	75%	2.00E-88	99.45
Bifidobacterium	Bifidobacterium	75%	2.00E-88	99.45
Bifidobacterium	Bifidobacterium	75%	2.00E-88	99.44

Bifidobacterium	Bifidobacterium	75%	2.00E-88	99.44
Bifidobacterium	Bifidobacterium	75%	2.00E-88	99.44
Bifidobacterium	Bifidobacterium	75%	2.00E-88	99.44
Bifidobacterium	Bifidobacterium	75%	2.00E-88	99.44
Bifidobacterium	Bifidobacterium	75%	2.00E-88	99.45
Bifidobacterium	Bifidobacterium	75%	2.00E-88	99.45
Bifidobacterium	Bifidobacterium	75%	2.00E-88	99.45
Bifidobacterium	Bifidobacterium	75%	2.00E-88	99.45
Bifidobacterium	Bifidobacterium	75%	2.00E-88	99.45
Bifidobacterium	Bifidobacterium	75%	2.00E-88	99.45
Bifidobacterium	Bifidobacterium	75%	2.00E-88	99.45
Bifidobacterium	Bifidobacterium	75%	2.00E-88	99.44
Bifidobacterium	Bifidobacterium	75%	2.00E-88	99.45
Bifidobacterium	Bifidobacterium	75%	2.00E-88	99.44
Bifidobacterium	Bifidobacterium	75%	2.00E-88	99.44
Bifidobacterium	Bifidobacterium	75%	2.00E-88	99.44
Bifidobacterium	Bifidobacterium	75%	2.00E-88	99.45
Bifidobacterium	Bifidobacterium	100%	2.00E-88	91.32
Bifidobacterium	Bifidobacterium	75%	2.00E-88	99.45
Bifidobacterium	Bifidobacterium	75%	2.00E-88	99.45
Bifidobacterium	Bifidobacterium	75%	2.00E-88	99.45
Bifidobacterium	Bifidobacterium	75%	2.00E-88	99.45
Bifidobacterium	Bifidobacterium	75%	2.00E-88	99.45
Bifidobacterium	Bifidobacterium	75%	2.00E-88	99.44
Bifidobacterium	Bifidobacterium	75%	2.00E-88	99.44
Bifidobacterium	Bifidobacterium	75%	2.00E-88	99.44
Bifidobacterium	Bifidobacterium	75%	2.00E-88	99.45
Bifidobacterium	Bifidobacterium	75%	2.00E-88	99.44
Bifidobacterium	Bifidobacterium	75%	2.00E-88	99.44
Bifidobacterium	Bifidobacterium	75%	2.00E-88	99.44
Bifidobacterium	Bifidobacterium	75%	2.00E-88	99.45
Bifidobacterium	Bifidobacterium	75%	2.00E-88	99.44
Bifidobacterium	Bifidobacterium	75%	2.00E-88	99.45
Bifidobacterium	Bifidobacterium	75%	2.00E-88	99.45
Bifidobacterium	Bifidobacterium	75%	2.00E-88	99.44
Bifidobacterium	Bifidobacterium	75%	2.00E-88	99.44
Bifidobacterium	Bifidobacterium	75%	2.00E-88	99.44
Bifidobacterium	Bifidobacterium	75%	2.00E-88	99.45
Bifidobacterium	Bifidobacterium	75%	2.00E-88	99.44
Bifidobacterium	Bifidobacterium	75%	2.00E-88	99.44
Bifidobacterium	Bifidobacterium	75%	2.00E-88	99.45
Bifidobacterium	Bifidobacterium	75%	2.00E-88	99.44
Bifidobacterium	Bifidobacterium	75%	2.00E-88	99.44
Bifidobacterium	Bifidobacterium	75%	2.00E-88	99.45

Bifidobacterium	Bifidobacterium	75%	2.00E-88	99.44
Bifidobacterium	Bifidobacterium	75%	2.00E-88	99.44
Bifidobacterium	Bifidobacterium	75%	2.00E-88	99.44
Bifidobacterium	Bifidobacterium	75%	2.00E-88	99.44
Bifidobacterium	Bifidobacterium	75%	2.00E-88	99.44
Bifidobacterium	Bifidobacterium	75%	2.00E-88	99.44
Bifidobacterium	Bifidobacterium	75%	2.00E-88	99.45
Bifidobacterium	Bifidobacterium	75%	2.00E-88	99.45
Bifidobacterium	Bifidobacterium	75%	2.00E-88	99.45
Bifidobacterium	Bifidobacterium	75%	2.00E-88	99.44
Bifidobacterium	Bifidobacterium	75%	2.00E-88	99.44
Bifidobacterium	Bifidobacterium	75%	2.00E-88	99.44
Bifidobacterium	Bifidobacterium	75%	2.00E-88	99.45
Bifidobacterium	Bifidobacterium	75%	2.00E-88	99.45
Bifidobacterium	Bifidobacterium	75%	2.00E-88	99.44
Bifidobacterium	Bifidobacterium	75%	2.00E-88	99.45
Bifidobacterium	Bifidobacterium	75%	2.00E-88	99.45
Bifidobacterium	Bifidobacterium	75%	2.00E-88	99.44
Bifidobacterium	Bifidobacterium	75%	2.00E-88	99.45
Bifidobacterium	Bifidobacterium	75%	2.00E-88	99.45
Bifidobacterium	Bifidobacterium	75%	2.00E-88	99.44
Bifidobacterium	Bifidobacterium	75%	2.00E-88	99.45
Bifidobacterium	Bifidobacterium	75%	2.00E-88	99.44
Bifidobacterium	Bifidobacterium	75%	2.00E-88	99.44
Bifidobacterium	Bifidobacterium	75%	2.00E-88	99.44
Bifidobacterium	Bifidobacterium	75%	2.00E-88	99.44
Bifidobacterium	Bifidobacterium	75%	2.00E-88	99.44
Bifidobacterium	Bifidobacterium	75%	2.00E-88	99.45
Bifidobacterium	Bifidobacterium	75%	2.00E-88	99.45
Bifidobacterium	Bifidobacterium	75%	2.00E-88	99.45
Bifidobacterium	Bifidobacterium	75%	2.00E-88	99.44
Bifidobacterium	Bifidobacterium	75%	2.00E-88	99.45
Bifidobacterium	Bifidobacterium	75%	2.00E-88	99.44
Bifidobacterium	Bifidobacterium	75%	2.00E-88	99.45
Bifidobacterium	Bifidobacterium	75%	2.00E-88	99.44
Bifidobacterium	Bifidobacterium	75%	2.00E-88	99.45
Bifidobacterium	Bifidobacterium	75%	2.00E-88	99.44
Bifidobacterium	Bifidobacterium	75%	2.00E-88	99.44
Bifidobacterium	Bifidobacterium	75%	2.00E-88	99.45
Bifidobacterium	Bifidobacterium	75%	2.00E-88	99.45
Bifidobacterium	Bifidobacterium	75%	2.00E-88	99.44
Bifidobacterium	Bifidobacterium	81%	2.00E-88	97.4
Bifidobacterium	Bifidobacterium	75%	8.00E-88	99.44
Bifidobacterium	Bifidobacterium	76%	8.00E-88	98.91

Bifidobacterium	Bifidobacterium	75%	8.00E-88	99.44
Bifidobacterium	Bifidobacterium	75%	8.00E-88	99.44
Bifidobacterium	Bifidobacterium	75%	8.00E-88	99.44
Bifidobacterium	Bifidobacterium	75%	8.00E-88	99.44
Bifidobacterium	Bifidobacterium	75%	8.00E-88	99.44
Bifidobacterium	Bifidobacterium	75%	8.00E-88	99.44
Bifidobacterium	Bifidobacterium	75%	8.00E-88	99.44
Bifidobacterium	Bifidobacterium	75%	8.00E-88	99.44
Bifidobacterium	Bifidobacterium	75%	8.00E-88	99.44
Bifidobacterium	Bifidobacterium	74%	8.00E-88	100
Bifidobacterium	Bifidobacterium	74%	8.00E-88	100
Bifidobacterium	Bifidobacterium	75%	8.00E-88	99.44
Bifidobacterium	Bifidobacterium	75%	8.00E-88	99.44
Bifidobacterium	Bifidobacterium	74%	8.00E-88	100
Bifidobacterium	Bifidobacterium	75%	8.00E-88	99.44
Bifidobacterium	Bifidobacterium	75%	8.00E-88	99.44
Bifidobacterium	Bifidobacterium	75%	8.00E-88	99.44
Bifidobacterium	Bifidobacterium	75%	8.00E-88	99.44
Bifidobacterium	Bifidobacterium	75%	8.00E-88	99.44
Bifidobacterium	Bifidobacterium	74%	8.00E-88	100
Bifidobacterium	Bifidobacterium	74%	8.00E-88	100
Bifidobacterium	Bifidobacterium	74%	8.00E-88	100
Uncultured Bifid	uncultured Bifid	75%	8.00E-88	99.44
Uncultured Bifid	uncultured Bifid	75%	8.00E-88	99.44
Uncultured Bifid	uncultured Bifid	75%	8.00E-88	99.44
Uncultured Bifid	uncultured Bifid	75%	8.00E-88	99.44
Uncultured Bifid	uncultured Bifid	75%	8.00E-88	99.44
Uncultured Bifid	uncultured Bifid	75%	8.00E-88	99.44
Uncultured Bifid	uncultured Bifid	75%	8.00E-88	99.44
Uncultured Bifid	uncultured Bifid	75%	8.00E-88	99.44
Uncultured Bifid	uncultured Bifid	75%	8.00E-88	99.44
Uncultured Bifid	uncultured Bifid	75%	8.00E-88	99.44
Uncultured Bifid	uncultured Bifid	75%	8.00E-88	99.44
Uncultured Bifid	uncultured Bifid	75%	8.00E-88	99.44
Uncultured Bifid	uncultured Bifid	75%	8.00E-88	99.44
Uncultured Bifid	uncultured Bifid	75%	8.00E-88	99.44
Uncultured Bifid	uncultured Bifid	75%	8.00E-88	99.44
Uncultured Bifid	uncultured Bifid	75%	8.00E-88	99.44
Uncultured Bifid	uncultured Bifid	75%	8.00E-88	99.44
Uncultured Bifid	uncultured Bifid	75%	8.00E-88	99.44
Uncultured Bifid	uncultured Bifid	75%	8.00E-88	99.44
Uncultured Bifid	uncultured Bifid	75%	8.00E-88	99.44
Uncultured Bifid	uncultured Bifid	75%	8.00E-88	99.44
Uncultured Bifid	uncultured Bifid	75%	8.00E-88	99.44

Uncultured Bific	uncultured Bifid	75%	8.00E-88	99.44
Uncultured Bific	uncultured Bifid	75%	8.00E-88	99.44
Uncultured Bific	uncultured Bifid	75%	8.00E-88	99.44
Uncultured Bific	uncultured Bifid	75%	8.00E-88	99.44
Uncultured Bific	uncultured Bifid	75%	8.00E-88	99.44
Uncultured Bific	uncultured Bifid	75%	8.00E-88	99.44
Uncultured Bific	uncultured Bifid	75%	8.00E-88	99.44
Uncultured Bific	uncultured Bifid	75%	8.00E-88	99.44
Uncultured Bific	uncultured Bifid	75%	8.00E-88	99.44
Uncultured Bific	uncultured Bifid	75%	8.00E-88	99.44
Uncultured Bific	uncultured Bifid	75%	8.00E-88	99.44
Uncultured Bific	uncultured Bifid	75%	8.00E-88	99.44
Uncultured Bific	uncultured Bifid	75%	8.00E-88	99.44
Uncultured Bific	uncultured Bifid	75%	8.00E-88	99.44
Uncultured Bific	uncultured Bifid	75%	8.00E-88	99.44
Bifidobacterium	Bifidobacterium	75%	8.00E-88	99.44
Bifidobacterium	Bifidobacterium	75%	8.00E-88	99.44
Bifidobacterium	Bifidobacterium	75%	8.00E-88	99.44
Bifidobacterium	Bifidobacterium	74%	8.00E-88	100
Bifidobacterium	Bifidobacterium	75%	8.00E-88	99.44
Bifidobacterium	Bifidobacterium	75%	8.00E-88	99.44
Bifidobacterium	Bifidobacterium	75%	8.00E-88	99.44
Bifidobacterium	Bifidobacterium	75%	8.00E-88	99.44
Bifidobacterium	Bifidobacterium	75%	8.00E-88	99.44
Bifidobacterium	Bifidobacterium	75%	8.00E-88	99.44
Bifidobacterium	Bifidobacterium	75%	8.00E-88	99.44
Bifidobacterium	Bifidobacterium	75%	8.00E-88	99.44
Bifidobacterium	Bifidobacterium	75%	8.00E-88	99.44
Bifidobacterium	Bifidobacterium	75%	8.00E-88	99.44
Bifidobacterium	Bifidobacterium	75%	8.00E-88	99.44
Bifidobacterium	Bifidobacterium	75%	8.00E-88	99.44
Bifidobacterium	Bifidobacterium	75%	8.00E-88	99.44
Bifidobacterium	Bifidobacterium	75%	8.00E-88	99.44
Bifidobacterium	Bifidobacterium	75%	8.00E-88	99.44
Bifidobacterium	Bifidobacterium	75%	8.00E-88	99.44
Bifidobacterium	Bifidobacterium	75%	8.00E-88	99.44
Bifidobacterium	Bifidobacterium	75%	8.00E-88	99.44
Bifidobacterium	Bifidobacterium	75%	8.00E-88	99.44
Bifidobacterium	Bifidobacterium	75%	8.00E-88	99.44
Bifidobacterium	Bifidobacterium	75%	8.00E-88	99.44
Bifidobacterium	Bifidobacterium	74%	8.00E-88	100
Bifidobacterium	Bifidobacterium	75%	8.00E-88	99.44
Bifidobacterium	Bifidobacterium	75%	8.00E-88	99.44

Bifidobacterium	Bifidobacterium	75%	8.00E-88	99.44
Bifidobacterium	Bifidobacterium	74%	8.00E-88	100
Bifidobacterium	Bifidobacterium	75%	8.00E-88	99.44
Bifidobacterium	Bifidobacterium	75%	8.00E-88	99.44
Bifidobacterium	Bifidobacterium	75%	8.00E-88	99.44
Bifidobacterium	Bifidobacterium	74%	8.00E-88	100
Bifidobacterium	Bifidobacterium	75%	8.00E-88	99.44
Bifidobacterium	Bifidobacterium	75%	8.00E-88	99.44
Bifidobacterium	Bifidobacterium	75%	8.00E-88	99.44
Bifidobacterium	Bifidobacterium	75%	8.00E-88	99.44
Bifidobacterium	Bifidobacterium	75%	8.00E-88	99.44
Bifidobacterium	Bifidobacterium	75%	8.00E-88	99.44
Bifidobacterium	Bifidobacterium	75%	8.00E-88	99.44
Bifidobacterium	Bifidobacterium	75%	8.00E-88	99.44
Bifidobacterium	Bifidobacterium	75%	8.00E-88	99.44
Uncultured Bific	uncultured Bifid	75%	8.00E-88	99.44
Uncultured Bific	uncultured Bifid	75%	8.00E-88	99.44
Uncultured Bific	uncultured Bifid	75%	8.00E-88	99.44
Uncultured Bific	uncultured Bifid	75%	8.00E-88	99.44
Uncultured Bific	uncultured Bifid	75%	8.00E-88	99.44
Uncultured Bific	uncultured Bifid	75%	8.00E-88	99.44
Uncultured Bific	uncultured Bifid	75%	8.00E-88	99.44
Uncultured Bific	uncultured Bifid	75%	8.00E-88	99.44
Uncultured Bific	uncultured Bifid	75%	8.00E-88	99.44
Bifidobacterium	Bifidobacterium	80%	8.00E-88	97.38
Bifidobacterium	Bifidobacterium	80%	8.00E-88	97.38
Bifidobacterium	Bifidobacterium	78%	8.00E-88	98.38
Uncultured Bific	uncultured Bifid	75%	8.00E-88	99.44
Uncultured Bific	uncultured Bifid	75%	8.00E-88	99.44
Bifidobacterium	Bifidobacterium	76%	3.00E-87	98.9
Bifidobacterium	Bifidobacterium	76%	3.00E-87	98.9
Bifidobacterium	Bifidobacterium	76%	3.00E-87	98.9
Bifidobacterium	Bifidobacterium	75%	3.00E-87	99.44
Bifidobacterium	Bifidobacterium	76%	3.00E-87	98.9
Bifidobacterium	Bifidobacterium	73%	3.00E-87	100
Bifidobacterium	Bifidobacterium	73%	3.00E-87	100
Bifidobacterium	Bifidobacterium	76%	3.00E-87	98.9
Bifidobacterium	Bifidobacterium	76%	3.00E-87	98.9
Bifidobacterium	Bifidobacterium	76%	3.00E-87	98.9
Bifidobacterium	Bifidobacterium	76%	3.00E-87	98.9
Bifidobacterium	Bifidobacterium	76%	3.00E-87	98.9
Bifidobacterium	Bifidobacterium	76%	3.00E-87	98.9
Bifidobacterium	Bifidobacterium	75%	3.00E-87	98.9
Bifidobacterium	Bifidobacterium	75%	3.00E-87	98.9

Bifidobacterium	Bifidobacterium	76%	3.00E-87	98.9
Bifidobacterium	Bifidobacterium	75%	3.00E-87	98.9
Bifidobacterium	Bifidobacterium	75%	3.00E-87	98.9
Bifidobacterium	Bifidobacterium	75%	3.00E-87	98.9
Bifidobacterium	Bifidobacterium	76%	3.00E-87	98.9
Bifidobacterium	Bifidobacterium	76%	3.00E-87	98.9
Bifidobacterium	Bifidobacterium	76%	3.00E-87	98.9
Bifidobacterium	Bifidobacterium	75%	3.00E-87	99.44
Bifidobacterium	Bifidobacterium	76%	3.00E-87	98.9
Bifidobacterium	Bifidobacterium	76%	3.00E-87	98.9
Bifidobacterium	Bifidobacterium	76%	3.00E-87	98.9
Bifidobacterium	Bifidobacterium	76%	3.00E-87	98.9
Bifidobacterium	Bifidobacterium	75%	3.00E-87	98.9
Uncultured Bifi	uncultured Bifid	99%	3.00E-87	91.53
Uncultured Bifi	uncultured Bifid	76%	3.00E-87	98.9
Uncultured Bifi	uncultured Bifid	76%	3.00E-87	98.9
Bifidobacterium	Bifidobacterium	75%	3.00E-87	98.9
Bifidobacterium	Bifidobacterium	76%	3.00E-87	98.9
Bifidobacterium	Bifidobacterium	75%	3.00E-87	98.9
Bifidobacterium	Bifidobacterium	75%	3.00E-87	98.9
Bifidobacterium	Bifidobacterium	75%	3.00E-87	98.9
Bifidobacterium	Bifidobacterium	75%	3.00E-87	98.9
Bifidobacterium	Bifidobacterium	75%	3.00E-87	98.9
Bifidobacterium	Bifidobacterium	75%	3.00E-87	98.9
Bifidobacterium	Bifidobacterium	75%	3.00E-87	98.9
Bifidobacterium	Bifidobacterium	76%	3.00E-87	98.9
Bifidobacterium	Bifidobacterium	75%	3.00E-87	98.9
Bifidobacterium	Bifidobacterium	75%	3.00E-87	98.9
Uncultured Bifi	uncultured Bifid	75%	3.00E-87	99.44
Bifidobacterium	Bifidobacterium	75%	1.00E-86	98.9
Bifidobacterium	Bifidobacterium	75%	1.00E-86	98.9
Bifidobacterium	Bifidobacterium	75%	1.00E-86	98.9
Bifidobacterium	Bifidobacterium	75%	1.00E-86	98.9
Bifidobacterium	Bifidobacterium	75%	1.00E-86	98.9
Bifidobacterium	Bifidobacterium	75%	1.00E-86	98.9
Bifidobacterium	Bifidobacterium	75%	1.00E-86	98.89
Bifidobacterium	Bifidobacterium	75%	1.00E-86	98.9
Bifidobacterium	Bifidobacterium	75%	1.00E-86	98.9
Bifidobacterium	Bifidobacterium	75%	1.00E-86	98.89
Bifidobacterium	Bifidobacterium	75%	1.00E-86	98.9
Bifidobacterium	Bifidobacterium	75%	1.00E-86	98.9
Bifidobacterium	Bifidobacterium	75%	1.00E-86	98.9
Bifidobacterium	Bifidobacterium	75%	1.00E-86	98.9
Bifidobacterium	Bifidobacterium	75%	1.00E-86	98.9

Bifidobacterium	Bifidobacterium	75%	1.00E-86	98.89
Bifidobacterium	Bifidobacterium	75%	1.00E-86	98.89
Bifidobacterium	Bifidobacterium	75%	1.00E-86	98.89
Bifidobacterium	Bifidobacterium	75%	1.00E-86	98.89
Bifidobacterium	Bifidobacterium	75%	1.00E-86	98.89
Bifidobacterium	Bifidobacterium	75%	1.00E-86	98.89
Bifidobacterium	Bifidobacterium	75%	1.00E-86	98.9
Bifidobacterium	Bifidobacterium	75%	1.00E-86	98.89
Bifidobacterium	Bifidobacterium	75%	1.00E-86	98.9
Bifidobacterium	Bifidobacterium	75%	1.00E-86	98.9
Bifidobacterium	Bifidobacterium	75%	1.00E-86	98.89
Bifidobacterium	Bifidobacterium	75%	1.00E-86	98.89
Bifidobacterium	Bifidobacterium	75%	1.00E-86	98.9
Bifidobacterium	Bifidobacterium	75%	1.00E-86	98.89
Bifidobacterium	Bifidobacterium	75%	1.00E-86	98.89
Bifidobacterium	Bifidobacterium	75%	1.00E-86	98.89
Bifidobacterium	Bifidobacterium	75%	1.00E-86	98.9
Bifidobacterium	Bifidobacterium	75%	1.00E-86	98.89
Bifidobacterium	Bifidobacterium	75%	1.00E-86	98.89
Bifidobacterium	Bifidobacterium	75%	1.00E-86	98.9
Bifidobacterium	Bifidobacterium	75%	1.00E-86	98.89
Bifidobacterium	Bifidobacterium	75%	1.00E-86	98.89
Bifidobacterium	Bifidobacterium	75%	1.00E-86	98.89
Bifidobacterium	Bifidobacterium	75%	1.00E-86	98.89
Bifidobacterium	Bifidobacterium	75%	1.00E-86	98.89
Bifidobacterium	Bifidobacterium	75%	1.00E-86	98.89
Bifidobacterium	Bifidobacterium	75%	1.00E-86	98.89
Bifidobacterium	Bifidobacterium	75%	1.00E-86	98.9
Bifidobacterium	Bifidobacterium	75%	1.00E-86	98.9
Bifidobacterium	Bifidobacterium	75%	1.00E-86	98.9
Bifidobacterium	Bifidobacterium	75%	1.00E-86	98.9
Bifidobacterium	Bifidobacterium	75%	1.00E-86	98.89
Bifidobacterium	Bifidobacterium	75%	1.00E-86	98.9
Bifidobacterium	Bifidobacterium	75%	1.00E-86	98.9
Bifidobacterium	Bifidobacterium	75%	1.00E-86	98.89
Bifidobacterium	Bifidobacterium	75%	1.00E-86	98.89
Bifidobacterium	Bifidobacterium	75%	1.00E-86	98.9
Bifidobacterium	Bifidobacterium	74%	1.00E-86	99.44
Bifidobacterium	Bifidobacterium	75%	1.00E-86	98.9
Bifidobacterium	Bifidobacterium	75%	1.00E-86	98.9
Bifidobacterium	Bifidobacterium	75%	1.00E-86	98.89
Bifidobacterium	Bifidobacterium	75%	1.00E-86	98.89
Bifidobacterium	Bifidobacterium	75%	1.00E-86	98.9
Bifidobacterium	Bifidobacterium	75%	1.00E-86	98.9

Bifidobacterium	Bifidobacterium	75%	1.00E-86	98.9
Bifidobacterium	Bifidobacterium	75%	1.00E-86	98.9
Bifidobacterium	Bifidobacterium	75%	1.00E-86	98.9
Bifidobacterium	Bifidobacterium	75%	1.00E-86	98.9
Bifidobacterium	Bifidobacterium	75%	1.00E-86	98.9
Bifidobacterium	Bifidobacterium	75%	1.00E-86	98.9
Bifidobacterium	Bifidobacterium	75%	1.00E-86	98.89
Bifidobacterium	Bifidobacterium	75%	1.00E-86	98.89
Bifidobacterium	Bifidobacterium	75%	1.00E-86	98.9
Bifidobacterium	Bifidobacterium	75%	1.00E-86	98.9
Bifidobacterium	Bifidobacterium	75%	1.00E-86	98.9
Bifidobacterium	Bifidobacterium	75%	1.00E-86	98.89
Bifidobacterium	Bifidobacterium	75%	1.00E-86	98.9
Bifidobacterium	Bifidobacterium	75%	1.00E-86	98.9
Bifidobacterium	Bifidobacterium	75%	1.00E-86	98.9
Bifidobacterium	Bifidobacterium	75%	1.00E-86	98.9
Bifidobacterium	Bifidobacterium	75%	1.00E-86	98.9
Bifidobacterium	Bifidobacterium	75%	1.00E-86	98.9
Bifidobacterium	Bifidobacterium	75%	1.00E-86	98.89
Bifidobacterium	Bifidobacterium	75%	1.00E-86	98.89
Bifidobacterium	Bifidobacterium	75%	1.00E-86	98.89
Bifidobacterium	Bifidobacterium	75%	1.00E-86	98.89
Bifidobacterium	Bifidobacterium	75%	1.00E-86	98.9
Bifidobacterium	Bifidobacterium	75%	1.00E-86	98.89
Bifidobacterium	Bifidobacterium	75%	1.00E-86	98.9
Bifidobacterium	Bifidobacterium	75%	1.00E-86	98.89
Bifidobacterium	Bifidobacterium	75%	1.00E-86	98.89
Bifidobacterium	Bifidobacterium	75%	1.00E-86	98.9
Bifidobacterium	Bifidobacterium	75%	1.00E-86	98.9
Bifidobacterium	Bifidobacterium	75%	1.00E-86	98.89
Bifidobacterium	Bifidobacterium	75%	1.00E-86	98.89
Bifidobacterium	Bifidobacterium	75%	1.00E-86	98.89
Bifidobacterium	Bifidobacterium	75%	1.00E-86	98.89
Bifidobacterium	Bifidobacterium	75%	1.00E-86	98.9
Bifidobacterium	Bifidobacterium	75%	1.00E-86	98.89
Bifidobacterium	Bifidobacterium	75%	1.00E-86	98.89
Bifidobacterium	Bifidobacterium	75%	1.00E-86	98.9
Bifidobacterium	Bifidobacterium	75%	1.00E-86	98.89
Bifidobacterium	Bifidobacterium	75%	1.00E-86	98.9
Bifidobacterium	Bifidobacterium	75%	1.00E-86	98.89
Bifidobacterium	Bifidobacterium	75%	1.00E-86	98.89
Bifidobacterium	Bifidobacterium	75%	1.00E-86	98.89
Bifidobacterium	Bifidobacterium	75%	1.00E-86	98.89
Bifidobacterium	Bifidobacterium	75%	1.00E-86	98.89

Bifidobacterium	Bifidobacterium	75%	1.00E-86	98.9
Bifidobacterium	Bifidobacterium	75%	1.00E-86	98.9
Bifidobacterium	Bifidobacterium	75%	1.00E-86	98.9
Bifidobacterium	Bifidobacterium	75%	1.00E-86	98.89
Bifidobacterium	Bifidobacterium	75%	1.00E-86	98.89
Bifidobacterium	Bifidobacterium	75%	1.00E-86	98.89
Bifidobacterium	Bifidobacterium	75%	1.00E-86	98.89
Bifidobacterium	Bifidobacterium	75%	1.00E-86	98.89
Bifidobacterium	Bifidobacterium	75%	1.00E-86	98.9
Bifidobacterium	Bifidobacterium	75%	1.00E-86	98.9
Bifidobacterium	Bifidobacterium	75%	1.00E-86	98.89
Bifidobacterium	Bifidobacterium	75%	1.00E-86	98.9
Bifidobacterium	Bifidobacterium	75%	1.00E-86	98.9
Bifidobacterium	Bifidobacterium	75%	1.00E-86	98.9
Bifidobacterium	Bifidobacterium	75%	1.00E-86	98.9
Bifidobacterium	Bifidobacterium	75%	1.00E-86	98.9
Bifidobacterium	Bifidobacterium	75%	1.00E-86	98.9
Bifidobacterium	Bifidobacterium	75%	1.00E-86	98.9
Bifidobacterium	Bifidobacterium	75%	1.00E-86	98.9
Bifidobacterium	Bifidobacterium	75%	1.00E-86	98.9
Bifidobacterium	Bifidobacterium	75%	1.00E-86	98.89
Bifidobacterium	Bifidobacterium	75%	1.00E-86	98.9
Bifidobacterium	Bifidobacterium	75%	1.00E-86	98.9
Bifidobacterium	Bifidobacterium	75%	1.00E-86	98.9
Bifidobacterium	Bifidobacterium	75%	1.00E-86	98.89
Bifidobacterium	Bifidobacterium	75%	1.00E-86	98.89
Bifidobacterium	Bifidobacterium	75%	1.00E-86	98.9
Bifidobacterium	Bifidobacterium	75%	1.00E-86	98.9
Bifidobacterium	Bifidobacterium	75%	1.00E-86	98.9
Bifidobacterium	Bifidobacterium	75%	1.00E-86	98.9
Bifidobacterium	Bifidobacterium	75%	1.00E-86	98.9
Bifidobacterium	Bifidobacterium	75%	1.00E-86	98.9
Bifidobacterium	Bifidobacterium	75%	1.00E-86	98.9
Bifidobacterium	Bifidobacterium	75%	1.00E-86	98.9
Bifidobacterium	Bifidobacterium	75%	1.00E-86	98.9
Bifidobacterium	Bifidobacterium	75%	1.00E-86	98.9
Bifidobacterium	Bifidobacterium	75%	1.00E-86	98.9
Bifidobacterium	Bifidobacterium	75%	1.00E-86	98.89
Bifidobacterium	Bifidobacterium	75%	1.00E-86	98.89
Bifidobacterium	Bifidobacterium	75%	1.00E-86	98.9
Bifidobacterium	Bifidobacterium	75%	1.00E-86	98.89
Bifidobacterium	Bifidobacterium	75%	1.00E-86	98.89
Bifidobacterium	Bifidobacterium	75%	1.00E-86	98.9
Bifidobacterium	Bifidobacterium	75%	1.00E-86	98.9

Bifidobacterium	Bifidobacterium	75%	1.00E-86	98.9
Bifidobacterium	Bifidobacterium	75%	1.00E-86	98.9
Bifidobacterium	Bifidobacterium	75%	1.00E-86	98.9
Bifidobacterium	Bifidobacterium	75%	1.00E-86	98.9
Bifidobacterium	Bifidobacterium	75%	1.00E-86	98.9
Bifidobacterium	Bifidobacterium	75%	1.00E-86	98.9
Bifidobacterium	Bifidobacterium	75%	1.00E-86	98.89
Bifidobacterium	Bifidobacterium	75%	1.00E-86	98.89
Bifidobacterium	Bifidobacterium	75%	1.00E-86	98.89
Bifidobacterium	Bifidobacterium	75%	1.00E-86	98.9
Bifidobacterium	Bifidobacterium	75%	1.00E-86	98.9
Bifidobacterium	Bifidobacterium	75%	1.00E-86	98.9
Bifidobacterium	Bifidobacterium	75%	1.00E-86	98.9
Bifidobacterium	Bifidobacterium	75%	1.00E-86	98.9
Bifidobacterium	Bifidobacterium	75%	1.00E-86	98.9
Bifidobacterium	Bifidobacterium	75%	1.00E-86	98.9
Bifidobacterium	Bifidobacterium	75%	1.00E-86	98.9
Bifidobacterium	Bifidobacterium	75%	1.00E-86	98.9
Bifidobacterium	Bifidobacterium	75%	1.00E-86	98.9
Bifidobacterium	Bifidobacterium	75%	1.00E-86	98.9
Bifidobacterium	Bifidobacterium	75%	1.00E-86	98.9
Bifidobacterium	Bifidobacterium	75%	1.00E-86	98.9
Bifidobacterium	Bifidobacterium	75%	1.00E-86	98.89
Bifidobacterium	Bifidobacterium	75%	1.00E-86	98.89
Bifidobacterium	Bifidobacterium	75%	1.00E-86	98.89
Bifidobacterium	Bifidobacterium	75%	1.00E-86	98.9
Bifidobacterium	Bifidobacterium	75%	1.00E-86	98.89
Bifidobacterium	Bifidobacterium	75%	1.00E-86	98.89
Bifidobacterium	Bifidobacterium	75%	1.00E-86	98.9
Bifidobacterium	Bifidobacterium	75%	1.00E-86	98.89
Bifidobacterium	Bifidobacterium	75%	1.00E-86	98.9
Bifidobacterium	Bifidobacterium	75%	1.00E-86	98.89
Bifidobacterium	Bifidobacterium	75%	1.00E-86	98.89
Bifidobacterium	Bifidobacterium	75%	1.00E-86	98.89
Bifidobacterium	Bifidobacterium	75%	1.00E-86	98.9
Bifidobacterium	Bifidobacterium	75%	1.00E-86	98.89
Bifidobacterium	Bifidobacterium	100%	1.00E-86	91.14
Bifidobacterium	Bifidobacterium	75%	1.00E-86	98.89
Bifidobacterium	Bifidobacterium	75%	1.00E-86	98.89
Bifidobacterium	Bifidobacterium	75%	1.00E-86	98.9
Bifidobacterium	Bifidobacterium	75%	1.00E-86	98.89
Bifidobacterium	Bifidobacterium	75%	1.00E-86	98.9
Bifidobacterium	Bifidobacterium	75%	1.00E-86	98.9
Bifidobacterium	Bifidobacterium	75%	1.00E-86	98.9

Bifidobacterium	Bifidobacterium	75%	1.00E-86	98.9
Bifidobacterium	Bifidobacterium	75%	1.00E-86	98.9
Bifidobacterium	Bifidobacterium	75%	1.00E-86	98.9
Bifidobacterium	Bifidobacterium	75%	1.00E-86	98.9
Bifidobacterium	Bifidobacterium	75%	1.00E-86	98.9
Bifidobacterium	Bifidobacterium	75%	1.00E-86	98.9
Bifidobacterium	Bifidobacterium	75%	1.00E-86	98.9
Bifidobacterium	Bifidobacterium	75%	1.00E-86	98.9
Bifidobacterium	Bifidobacterium	75%	1.00E-86	98.9
Bifidobacterium	Bifidobacterium	75%	1.00E-86	98.9
Bifidobacterium	Bifidobacterium	75%	1.00E-86	98.9
Bifidobacterium	Bifidobacterium	74%	1.00E-86	99.44
Bifidobacterium	Bifidobacterium	74%	1.00E-86	99.44
Bifidobacterium	Bifidobacterium	73%	1.00E-86	100
Bifidobacterium	Bifidobacterium	75%	1.00E-86	98.9
Bifidobacterium	Bifidobacterium	75%	1.00E-86	98.89
Uncultured Bifid	uncultured Bifid	98%	1.00E-86	91.49
Bifidobacterium	Bifidobacterium	75%	1.00E-86	98.89
Uncultured Bifid	uncultured Bifid	75%	1.00E-86	98.89
Uncultured Bifid	uncultured Bifid	75%	1.00E-86	98.89
Uncultured Bifid	uncultured Bifid	75%	1.00E-86	98.89
Uncultured Bifid	uncultured Bifid	75%	1.00E-86	98.89
Uncultured Bifid	uncultured Bifid	75%	1.00E-86	98.9
Uncultured Bifid	uncultured Bifid	75%	1.00E-86	98.9
Uncultured Bifid	uncultured Bifid	75%	1.00E-86	98.89
Uncultured Bifid	uncultured Bifid	75%	1.00E-86	98.89
Uncultured Bifid	uncultured Bifid	75%	1.00E-86	98.9
Uncultured Bifid	uncultured Bifid	75%	1.00E-86	98.9
Uncultured Bifid	uncultured Bifid	75%	1.00E-86	98.9
Uncultured Bifid	uncultured Bifid	75%	1.00E-86	98.9
Uncultured Bifid	uncultured Bifid	75%	1.00E-86	98.89
Uncultured Bifid	uncultured Bifid	75%	1.00E-86	98.9
Uncultured Bifid	uncultured Bifid	76%	1.00E-86	98.9
Uncultured Bifid	uncultured Bifid	75%	1.00E-86	98.89
Bifidobacterium	Bifidobacterium	75%	1.00E-86	98.9
Bifidobacterium	Bifidobacterium	75%	1.00E-86	98.89
Bifidobacterium	Bifidobacterium	73%	1.00E-86	100
Bifidobacterium	Bifidobacterium	74%	1.00E-86	99.44
Bifidobacterium	Bifidobacterium	75%	1.00E-86	98.9
Bifidobacterium	Bifidobacterium	75%	1.00E-86	98.9
Bifidobacterium	Bifidobacterium	75%	1.00E-86	98.89
Bifidobacterium	Bifidobacterium	75%	1.00E-86	98.9
Bifidobacterium	Bifidobacterium	75%	1.00E-86	98.9
Bifidobacterium	Bifidobacterium	74%	1.00E-86	99.44

Bifidobacterium	Bifidobacterium	75%	1.00E-86	98.9
Bifidobacterium	Bifidobacterium	75%	1.00E-86	98.89
Bifidobacterium	Bifidobacterium	75%	1.00E-86	98.9
Bifidobacterium	Bifidobacterium	75%	1.00E-86	98.89
Bifidobacterium	Bifidobacterium	75%	1.00E-86	98.9
Bifidobacterium	Bifidobacterium	75%	1.00E-86	98.9
Bifidobacterium	Bifidobacterium	75%	1.00E-86	98.89
Bifidobacterium	Bifidobacterium	75%	1.00E-86	98.89
Bifidobacterium	Bifidobacterium	75%	1.00E-86	98.89
Bifidobacterium	Bifidobacterium	75%	1.00E-86	98.89
Bifidobacterium	Bifidobacterium	75%	1.00E-86	98.89
Bifidobacterium	Bifidobacterium	75%	1.00E-86	98.89
Bifidobacterium	Bifidobacterium	75%	1.00E-86	98.89
Bifidobacterium	Bifidobacterium	75%	1.00E-86	98.9
Bifidobacterium	Bifidobacterium	75%	1.00E-86	98.9
Bifidobacterium	Bifidobacterium	73%	1.00E-86	100
Bifidobacterium	Bifidobacterium	75%	1.00E-86	98.9
Bifidobacterium	Bifidobacterium	75%	1.00E-86	98.9
Bifidobacterium	Bifidobacterium	74%	1.00E-86	99.44
Bifidobacterium	Bifidobacterium	75%	1.00E-86	98.9
Bifidobacterium	Bifidobacterium	74%	1.00E-86	99.44
Bifidobacterium	Bifidobacterium	75%	1.00E-86	98.89
Bifidobacterium	Bifidobacterium	75%	1.00E-86	98.9
Bifidobacterium	Bifidobacterium	75%	1.00E-86	98.9
Bifidobacterium	Bifidobacterium	75%	1.00E-86	98.9
Bifidobacterium	Bifidobacterium	75%	1.00E-86	98.89
Bifidobacterium	Bifidobacterium	75%	1.00E-86	98.9
Bifidobacterium	Bifidobacterium	75%	1.00E-86	98.9
Bifidobacterium	Bifidobacterium	74%	1.00E-86	99.44
Bifidobacterium	Bifidobacterium	75%	1.00E-86	98.9
Bifidobacterium	Bifidobacterium	75%	1.00E-86	98.89
Bifidobacterium	Bifidobacterium	75%	1.00E-86	98.9
Bifidobacterium	Bifidobacterium	75%	1.00E-86	98.9
Bifidobacterium	Bifidobacterium	75%	1.00E-86	98.9
Bifidobacterium	Bifidobacterium	75%	1.00E-86	98.89
Bifidobacterium	Bifidobacterium	75%	1.00E-86	98.9
Bifidobacterium	Bifidobacterium	75%	1.00E-86	98.9
Bifidobacterium	Bifidobacterium	75%	1.00E-86	98.9
Bifidobacterium	Bifidobacterium	75%	1.00E-86	98.9
Bifidobacterium	Bifidobacterium	75%	1.00E-86	98.89
Bifidobacterium	Bifidobacterium	75%	1.00E-86	98.89
Bifidobacterium	Bifidobacterium	75%	1.00E-86	98.9
Bifidobacterium	Bifidobacterium	75%	1.00E-86	98.9
Bifidobacterium	Bifidobacterium	74%	1.00E-86	99.44

Bifidobacterium	Bifidobacterium	75%	1.00E-86	98.89
Bifidobacterium	Bifidobacterium	75%	1.00E-86	98.9
Bifidobacterium	Bifidobacterium	75%	1.00E-86	98.9
Bifidobacterium	Bifidobacterium	75%	1.00E-86	98.9
Bifidobacterium	Bifidobacterium	75%	1.00E-86	98.9
Bifidobacterium	Bifidobacterium	75%	1.00E-86	98.9
Bifidobacterium	Bifidobacterium	75%	1.00E-86	98.9
Bifidobacterium	Bifidobacterium	75%	1.00E-86	98.89
Bifidobacterium	Bifidobacterium	75%	1.00E-86	98.89
Bifidobacterium	Bifidobacterium	75%	1.00E-86	98.89
Bifidobacterium	Bifidobacterium	75%	1.00E-86	98.89
Bifidobacterium	Bifidobacterium	75%	1.00E-86	98.89
Bifidobacterium	Bifidobacterium	74%	1.00E-86	99.44
Bifidobacterium	Bifidobacterium	75%	1.00E-86	98.9
Bifidobacterium	Bifidobacterium	75%	1.00E-86	98.89
Bifidobacterium	Bifidobacterium	75%	1.00E-86	98.89
Bifidobacterium	Bifidobacterium	75%	1.00E-86	98.89
Bifidobacterium	Bifidobacterium	75%	1.00E-86	98.89
Bifidobacterium	Bifidobacterium	75%	1.00E-86	98.89
Bifidobacterium	Bifidobacterium	75%	1.00E-86	98.9
Bifidobacterium	Bifidobacterium	75%	1.00E-86	98.89
Bifidobacterium	Bifidobacterium	75%	1.00E-86	98.89
Bifidobacterium	Bifidobacterium	75%	1.00E-86	98.89
Bifidobacterium	Bifidobacterium	75%	1.00E-86	98.89
Bifidobacterium	Bifidobacterium	75%	1.00E-86	98.89
Bifidobacterium	Bifidobacterium	75%	1.00E-86	98.89
Bifidobacterium	Bifidobacterium	75%	1.00E-86	98.89
Bifidobacterium	Bifidobacterium	75%	1.00E-86	98.89
Bifidobacterium	Bifidobacterium	75%	1.00E-86	98.89
Bifidobacterium	Bifidobacterium	74%	1.00E-86	99.44
Bifidobacterium	Bifidobacterium	75%	1.00E-86	98.89
Bifidobacterium	Bifidobacterium	75%	1.00E-86	98.89
Bifidobacterium	Bifidobacterium	74%	1.00E-86	99.44
Bifidobacterium	Bifidobacterium	75%	1.00E-86	98.9
Bifidobacterium	Bifidobacterium	75%	1.00E-86	98.9
Bifidobacterium	Bifidobacterium	75%	1.00E-86	98.89
Bifidobacterium	Bifidobacterium	75%	1.00E-86	98.89
Bifidobacterium	Bifidobacterium	75%	1.00E-86	98.89
Bifidobacterium	Bifidobacterium	75%	1.00E-86	98.9
Bifidobacterium	Bifidobacterium	75%	1.00E-86	98.9
Bifidobacterium	Bifidobacterium	75%	1.00E-86	98.9
Bifidobacterium	Bifidobacterium	75%	1.00E-86	98.9
Bifidobacterium	Bifidobacterium	75%	1.00E-86	98.9
Bifidobacterium	Bifidobacterium	75%	1.00E-86	98.9

Bifidobacterium	Bifidobacterium	75%	1.00E-86	98.9
Bifidobacterium	Bifidobacterium	75%	1.00E-86	98.9
Bifidobacterium	Bifidobacterium	75%	1.00E-86	98.9
Bifidobacterium	Bifidobacterium	75%	1.00E-86	98.9
Bifidobacterium	Bifidobacterium	75%	1.00E-86	98.89
Bifidobacterium	Bifidobacterium	75%	1.00E-86	98.9
Bifidobacterium	Bifidobacterium	75%	1.00E-86	98.89
Bifidobacterium	Bifidobacterium	75%	1.00E-86	98.89
Bifidobacterium	Bifidobacterium	75%	1.00E-86	98.89
Bifidobacterium	Bifidobacterium	75%	1.00E-86	98.89
Bifidobacterium	Bifidobacterium	75%	1.00E-86	98.9
Bifidobacterium	Bifidobacterium	75%	1.00E-86	98.9
Bifidobacterium	Bifidobacterium	75%	1.00E-86	98.9
Bifidobacterium	Bifidobacterium	75%	1.00E-86	98.9
Bifidobacterium	Bifidobacterium	75%	1.00E-86	98.9
Bifidobacterium	Bifidobacterium	75%	1.00E-86	98.9
Bifidobacterium	Bifidobacterium	75%	1.00E-86	98.89
Bifidobacterium	Bifidobacterium	75%	1.00E-86	98.9
Bifidobacterium	Bifidobacterium	75%	1.00E-86	98.9
Bifidobacterium	Bifidobacterium	75%	1.00E-86	98.9
Bifidobacterium	Bifidobacterium	75%	1.00E-86	98.9
Bifidobacterium	Bifidobacterium	75%	1.00E-86	98.9
Bifidobacterium	Bifidobacterium	75%	1.00E-86	98.89
Bifidobacterium	Bifidobacterium	75%	1.00E-86	98.9
Bifidobacterium	Bifidobacterium	75%	1.00E-86	98.89
Bifidobacterium	Bifidobacterium	75%	1.00E-86	98.89
Bifidobacterium	Bifidobacterium	75%	1.00E-86	98.89
Bifidobacterium	Bifidobacterium	75%	1.00E-86	98.89
Bifidobacterium	Bifidobacterium	75%	1.00E-86	98.89
Bifidobacterium	Bifidobacterium	75%	1.00E-86	98.89
Bifidobacterium	Bifidobacterium	75%	1.00E-86	98.89
Bifidobacterium	Bifidobacterium	75%	1.00E-86	98.89
Bifidobacterium	Bifidobacterium	75%	1.00E-86	98.89
Bifidobacterium	Bifidobacterium	75%	1.00E-86	98.89
Bifidobacterium	Bifidobacterium	75%	1.00E-86	98.9
Bifidobacterium	Bifidobacterium	75%	1.00E-86	98.9
Bifidobacterium	Bifidobacterium	75%	1.00E-86	98.89
Bifidobacterium	Bifidobacterium	75%	1.00E-86	98.89
Bifidobacterium	Bifidobacterium	75%	1.00E-86	98.9
Bifidobacterium	Bifidobacterium	75%	1.00E-86	98.9
Bifidobacterium	Bifidobacterium	75%	1.00E-86	98.9
Bifidobacterium	Bifidobacterium	75%	1.00E-86	98.9
Bifidobacterium	Bifidobacterium	75%	1.00E-86	98.9
Bifidobacterium	Bifidobacterium	75%	1.00E-86	98.9

Bifidobacterium	Bifidobacterium	75%	1.00E-86	98.89
Bifidobacterium	Bifidobacterium	75%	1.00E-86	98.9
Bifidobacterium	Bifidobacterium	75%	1.00E-86	98.9
Bifidobacterium	Bifidobacterium	75%	1.00E-86	98.9
Bifidobacterium	Bifidobacterium	75%	1.00E-86	98.9
Bifidobacterium	Bifidobacterium	75%	1.00E-86	98.9
Bifidobacterium	Bifidobacterium	75%	1.00E-86	98.89
Bifidobacterium	Bifidobacterium	75%	1.00E-86	98.89
Bifidobacterium	Bifidobacterium	75%	1.00E-86	98.9
Bifidobacterium	Bifidobacterium	75%	1.00E-86	98.89
Bifidobacterium	Bifidobacterium	75%	1.00E-86	98.89
Bifidobacterium	Bifidobacterium	75%	1.00E-86	98.89
Bifidobacterium	Bifidobacterium	75%	1.00E-86	98.89
Bifidobacterium	Bifidobacterium	75%	1.00E-86	98.9
Bifidobacterium	Bifidobacterium	75%	1.00E-86	98.89
Bifidobacterium	Bifidobacterium	75%	1.00E-86	98.89
Bifidobacterium	Bifidobacterium	75%	1.00E-86	98.89
Bifidobacterium	Bifidobacterium	75%	1.00E-86	98.89
Bifidobacterium	Bifidobacterium	75%	1.00E-86	98.9
Bifidobacterium	Bifidobacterium	75%	1.00E-86	98.89
Bifidobacterium	Bifidobacterium	75%	1.00E-86	98.9
Bifidobacterium	Bifidobacterium	75%	1.00E-86	98.9
Bifidobacterium	Bifidobacterium	74%	1.00E-86	99.44
Bifidobacterium	Bifidobacterium	75%	1.00E-86	98.89
Bifidobacterium	Bifidobacterium	75%	1.00E-86	98.89
Bifidobacterium	Bifidobacterium	75%	1.00E-86	98.89
Bifidobacterium	Bifidobacterium	75%	1.00E-86	98.89
Bifidobacterium	Bifidobacterium	75%	1.00E-86	98.89
Bifidobacterium	Bifidobacterium	75%	1.00E-86	98.9
Bifidobacterium	Bifidobacterium	75%	1.00E-86	98.9
Bifidobacterium	Bifidobacterium	75%	1.00E-86	98.89
Bifidobacterium	Bifidobacterium	75%	1.00E-86	98.9
Bifidobacterium	Bifidobacterium	75%	1.00E-86	98.9
Bifidobacterium	Bifidobacterium	75%	1.00E-86	98.89
Bifidobacterium	Bifidobacterium	75%	1.00E-86	98.9
Bifidobacterium	Bifidobacterium	75%	1.00E-86	98.89
Bifidobacterium	Bifidobacterium	75%	1.00E-86	98.9
Bifidobacterium	Bifidobacterium	75%	1.00E-86	98.9
Bifidobacterium	Bifidobacterium	75%	1.00E-86	98.89
Bifidobacterium	Bifidobacterium	75%	1.00E-86	98.89
Bifidobacterium	Bifidobacterium	75%	1.00E-86	98.89
Bifidobacterium	Bifidobacterium	75%	1.00E-86	98.89
Bifidobacterium	Bifidobacterium	75%	1.00E-86	98.89
Bifidobacterium	Bifidobacterium	75%	1.00E-86	98.9

Bifidobacterium	Bifidobacterium	75%	1.00E-86	98.89
Bifidobacterium	Bifidobacterium	75%	1.00E-86	98.89
Bifidobacterium	Bifidobacterium	75%	1.00E-86	98.9
Bifidobacterium	Bifidobacterium	75%	1.00E-86	98.9
Bifidobacterium	Bifidobacterium	75%	1.00E-86	98.9
Bifidobacterium	Bifidobacterium	75%	1.00E-86	98.9
Bifidobacterium	Bifidobacterium	75%	1.00E-86	98.9
Bifidobacterium	Bifidobacterium	75%	1.00E-86	98.89
Bifidobacterium	Bifidobacterium	75%	1.00E-86	98.89
Bifidobacterium	Bifidobacterium	75%	1.00E-86	98.89
Bifidobacterium	Bifidobacterium	75%	1.00E-86	98.9
Bifidobacterium	Bifidobacterium	75%	1.00E-86	98.9
Bifidobacterium	Bifidobacterium	82%	1.00E-86	96.41
Bifidobacterium	Bifidobacterium	75%	4.00E-86	98.89
Bifidobacterium	Bifidobacterium	75%	4.00E-86	98.89
Bifidobacterium	Bifidobacterium	75%	4.00E-86	98.89
Bifidobacterium	Bifidobacterium	75%	4.00E-86	98.89
Bifidobacterium	Bifidobacterium	75%	4.00E-86	98.89
Bifidobacterium	Bifidobacterium	75%	4.00E-86	98.89
Bifidobacterium	Bifidobacterium	75%	4.00E-86	98.88
Bifidobacterium	Bifidobacterium	75%	4.00E-86	98.88
Bifidobacterium	Bifidobacterium	75%	4.00E-86	98.88
Bifidobacterium	Bifidobacterium	75%	4.00E-86	98.88
Bifidobacterium	Bifidobacterium	75%	4.00E-86	98.88
Bifidobacterium	Bifidobacterium	75%	4.00E-86	98.89
Bifidobacterium	Bifidobacterium	75%	4.00E-86	98.88
Bifidobacterium	Bifidobacterium	75%	4.00E-86	98.88
Bifidobacterium	Bifidobacterium	75%	4.00E-86	98.88
Bifidobacterium	Bifidobacterium	75%	4.00E-86	98.88
Bifidobacterium	Bifidobacterium	75%	4.00E-86	98.88
Bifidobacterium	Bifidobacterium	75%	4.00E-86	98.88
Bifidobacterium	Bifidobacterium	75%	4.00E-86	98.88
Bifidobacterium	Bifidobacterium	75%	4.00E-86	98.88
Bifidobacterium	Bifidobacterium	75%	4.00E-86	98.89
Bifidobacterium	Bifidobacterium	75%	4.00E-86	98.89
Bifidobacterium	Bifidobacterium	75%	4.00E-86	98.89
Bifidobacterium	Bifidobacterium	75%	4.00E-86	98.89
Bifidobacterium	Bifidobacterium	75%	4.00E-86	98.89
Bifidobacterium	Bifidobacterium	75%	4.00E-86	98.89
Bifidobacterium	Bifidobacterium	76%	4.00E-86	98.35
Bifidobacterium	Bifidobacterium	75%	4.00E-86	98.89
Bifidobacterium	Bifidobacterium	76%	4.00E-86	98.36
Bifidobacterium	Bifidobacterium	75%	4.00E-86	98.89
Bifidobacterium	Bifidobacterium	75%	4.00E-86	98.88

Bifidobacterium	Bifidobacterium	75%	4.00E-86	98.88
Bifidobacterium	Bifidobacterium	75%	4.00E-86	98.88
Bifidobacterium	Bifidobacterium	75%	4.00E-86	98.89
Bifidobacterium	Bifidobacterium	75%	4.00E-86	98.89
Bifidobacterium	Bifidobacterium	74%	4.00E-86	99.44
Bifidobacterium	Bifidobacterium	74%	4.00E-86	99.44
Bifidobacterium	Bifidobacterium	75%	4.00E-86	98.89
Bifidobacterium	Bifidobacterium	72%	4.00E-86	100
Bifidobacterium	Bifidobacterium	74%	4.00E-86	99.44
Bifidobacterium	Bifidobacterium	75%	4.00E-86	98.89
Uncultured Bifi	uncultured Bifid	75%	4.00E-86	98.88
Uncultured Bifi	uncultured Bifid	75%	4.00E-86	98.88
Uncultured Bifi	uncultured Bifid	75%	4.00E-86	98.88
Uncultured Bifi	uncultured Bifid	75%	4.00E-86	98.88
Uncultured Bifi	uncultured Bifid	75%	4.00E-86	98.88
Uncultured Bifi	uncultured Bifid	75%	4.00E-86	98.88
Uncultured Bifi	uncultured Bifid	75%	4.00E-86	98.89
Uncultured Bifi	uncultured Bifid	75%	4.00E-86	98.88
Uncultured Bifi	uncultured Bifid	75%	4.00E-86	98.89
Uncultured Bifi	uncultured Bifid	75%	4.00E-86	98.89
Uncultured Bifi	uncultured Bifid	75%	4.00E-86	98.88
Uncultured Bifi	uncultured Bifid	75%	4.00E-86	98.88
Uncultured Bifi	uncultured Bifid	75%	4.00E-86	98.89
Uncultured Bifi	uncultured Bifid	75%	4.00E-86	98.88
Uncultured Bifi	uncultured Bifid	75%	4.00E-86	98.88
Uncultured Bifi	uncultured Bifid	75%	4.00E-86	98.88
Uncultured Bifi	uncultured Bifid	75%	4.00E-86	98.88
Uncultured Bifi	uncultured Bifid	75%	4.00E-86	98.88
Uncultured Bifi	uncultured Bifid	75%	4.00E-86	98.88
Uncultured Bifi	uncultured Bifid	75%	4.00E-86	98.88
Uncultured Bifi	uncultured Bifid	75%	4.00E-86	98.88
Uncultured Bifi	uncultured Bifid	75%	4.00E-86	98.88
Uncultured Bifi	uncultured Bifid	75%	4.00E-86	98.88
Uncultured Bifi	uncultured Bifid	75%	4.00E-86	98.88
Uncultured Bifi	uncultured Bifid	75%	4.00E-86	98.89
Uncultured Bifi	uncultured Bifid	75%	4.00E-86	98.89
Uncultured Bifi	uncultured Bifid	75%	4.00E-86	98.89
Bifidobacterium	Bifidobacterium	72%	4.00E-86	100
Bifidobacterium	Bifidobacterium	72%	4.00E-86	100
Bifidobacterium	Bifidobacterium	75%	4.00E-86	98.89
Bifidobacterium	Bifidobacterium	72%	4.00E-86	100
Bifidobacterium	Bifidobacterium	74%	4.00E-86	99.44
Bifidobacterium	Bifidobacterium	75%	4.00E-86	98.89
Bifidobacterium	Bifidobacterium	75%	4.00E-86	98.89

Bifidobacterium	Bifidobacterium	74%	4.00E-86	99.43
Bifidobacterium	Bifidobacterium	75%	4.00E-86	98.88
Bifidobacterium	Bifidobacterium	75%	4.00E-86	98.88
Bifidobacterium	Bifidobacterium	75%	4.00E-86	98.89
Bifidobacterium	Bifidobacterium	75%	4.00E-86	98.88
Bifidobacterium	Bifidobacterium	75%	4.00E-86	98.89
Bifidobacterium	Bifidobacterium	75%	4.00E-86	98.88
Bifidobacterium	Bifidobacterium	75%	4.00E-86	98.88
Bifidobacterium	Bifidobacterium	74%	4.00E-86	99.44
Bifidobacterium	Bifidobacterium	75%	4.00E-86	98.89
Bifidobacterium	Bifidobacterium	75%	4.00E-86	98.89
Bifidobacterium	Bifidobacterium	75%	4.00E-86	98.89
Bifidobacterium	Bifidobacterium	75%	4.00E-86	98.89
Bifidobacterium	Bifidobacterium	74%	4.00E-86	99.43
Bifidobacterium	Bifidobacterium	75%	4.00E-86	98.89
Bifidobacterium	Bifidobacterium	75%	4.00E-86	98.89
Bifidobacterium	Bifidobacterium	75%	4.00E-86	98.89
Bifidobacterium	Bifidobacterium	75%	4.00E-86	98.89
Bifidobacterium	Bifidobacterium	75%	4.00E-86	98.89
Bifidobacterium	Bifidobacterium	74%	4.00E-86	99.43
Bifidobacterium	Bifidobacterium	75%	4.00E-86	98.89
Bifidobacterium	Bifidobacterium	74%	4.00E-86	99.43
Bifidobacterium	Bifidobacterium	75%	4.00E-86	98.88
Bifidobacterium	Bifidobacterium	75%	4.00E-86	98.89
Bifidobacterium	Bifidobacterium	75%	4.00E-86	98.89
Bifidobacterium	Bifidobacterium	75%	4.00E-86	98.89
Bifidobacterium	Bifidobacterium	75%	4.00E-86	98.89
Bifidobacterium	Bifidobacterium	75%	4.00E-86	98.88
Bifidobacterium	Bifidobacterium	74%	4.00E-86	99.44
