# Supplementary material for: Neonatal gut Bifidobacterium associates with indole-3-lactic acid levels in blood and risk of ADHD at age 10
Source: Mol Psychiatry. 2026 Feb 11;31(6):3544–57. doi: 10.1038/s41380-026-03480-z (PMC13190319; doi:10.1038/s41380-026-03480-z)
Supplement: Supplementary file 3 — Supplementary Methods [file 41380_2026_3480_MOESM3_ESM.pdf]

Preprocessing - iPsych Twin Study Cohort

Thermo .raw files were exported to the .mzML format using ProteoWizard's MSConvert10 and subsequently preprocessed using the Ion Identity Network workflow in MZmine[1,2] (version 3.9.0). Mass lists were created by considering mass spectra with retention times of 0.4-14 minutes and retaining MS1 intensities above 1E4 and MS2 intensities above 5E2. The chromatogram was built through the ADAP chromatogram builder by using the following parameters, minimum group size of scans: 5, group intensity threshold: 5E4, minimum highest intensity: 1.5E5, and *m/z* tolerance: 0.002 *m/z* or 5 ppm. The chromatogram was smoothed with a filter width of 5. The local minimum search algorithm was used for deconvolution with parameters set to, chromatographic threshold: 85%, minimum RT range (min): 0.05, minimum relative height: 0%, minimum absolute height: 5.0E4, min ratio of peak top/edge: 2.2, peak duration range (min): 0.01-0.5. The peaks were deisotoped by using the isotopic peak grouper function, with parameters set to, *m/z* tolerance: 0.002 *m/z* or 5 ppm, retention time tolerance: 0.15 min, monotonic shape: on, maximum charge: 2, representative isotope: most intense. Peaks from all samples were aligned, by using the join aligner function with parameters set to, *m/z* tolerance: 0.002 *m/z* or 5 ppm, retention time tolerance: 0.15 min, weight for *m/z*: 75, weight for retention time: 25. Rows were then filtered using the duplicate peak filter with the new average filter mode and *m/z* tolerance set to 0.001 *m/z* or 5 ppm and RT tolerance 0.03 min. Gap-filling was performed using the same mz and RT range gap filler, with a *m/z* tolerance of 0.002 *m/z* or 5ppm and a RT tolerance of 0.03 minutes. The metaCorrelate function was used to find correlating peak shapes with parameters set to, RT tolerance: 0.1 min, min height: 5.0E4, noise level: 1.5E5, min samples in all: 2 (abs), min samples in group: 0 (abs), min %-intensity overlap: 60%, exclude estimated features (gap-filled): on. Parameters for the correlation grouping were set as follows, min data points: 5, min data points on edge: 2, measure: Pearson, min feature shape correlation: 85%. Ion identity networking parameters were set to, *m/z* tolerance: 0.002 *m/z* or 5 ppm, check: one feature, min height: 1.5E5 with ion identity library parameters set to, MS mode: positive, maximum charge: 2, maximum molecules/cluster: 2, adducts: M+H, M+Na, M+K, modifications: M-H2O, M-NH3. Further ion identity networks were added with *m/z* tolerance: 0.002 *m/z* or 5 ppm, min height: 1.5E5 and ion identity library parameters set to, MS mode: positive, maximum charge: 2, maximum molecules/cluster: 6, adducts: M+H, M+Na, modifications: M-H2O, M-2H2O, M-3H2O, M-4H2O, M-5H2O and *m/z* tolerance: 0.002 *m/z* or 5 ppm, min height: 1.5E5, and annotation refinement on with parameters set to, delete smaller networks: link threshold: 4, delete networks without monomer: on, and ion identity library parameters set to MS mode: positive, maximum charge: 2,

maximum molecules/cluster: 2, adducts: M+H, M+Na, M+K, modifications: M-H2O, M-NH3. Finally, two feature tables were exported in the .csv format. One feature table containing all extracted mass spectral features and another feature table filtered for mass spectral features with associated fragmentation spectra (MS2). An aggregated list of MS2 fragmentation spectra was exported in the .mgf format and submitted to ion identity feature-based mass spectral molecular networking through the Global Natural Products Social Molecular Networking Platform (GNPS)[4,5].
